# Landscape of genomic diversity and trait discovery in soybean

**DOI:** 10.1038/srep23598

**Published:** 2016-03-31

**Authors:** Babu Valliyodan, Dan Qiu, Gunvant Patil, Peng Zeng, Jiaying Huang, Lu Dai, Chengxuan Chen, Yanjun Li, Trupti Joshi, Li Song, Tri D. Vuong, Theresa A. Musket, Dong Xu, J. Grover Shannon, Cheng Shifeng, Xin Liu, Henry T. Nguyen

**Affiliations:** 1 Division of Plant Sciences and National Center for Soybean Biotechnology (NCSB), University of Missouri, Columbia 65211, USA; 2 Beijing Genomics Institute-Shenzhen, Shenzhen, 518083, China; 3 Department of Molecular Microbiology and Immunology and Medical Research Office, School of Medicine, University of Missouri, Columbia, 65212; 4 Department of Computer Science, Informatics Institute and Christopher S. Bond Life Sciences Center, University of Missouri, Columbia, 65211, USA; 5 Division of Plant Sciences and NCSB, University of Missouri-Fisher Delta Research Center, Portageville, MO, 63873, USA

## Abstract

Cultivated soybean [*Glycine max* (L.) Merr.] is a primary source of vegetable oil and protein. We report a landscape analysis of genome-wide genetic variation and an association study of major domestication and agronomic traits in soybean. A total of 106 soybean genomes representing wild, landraces, and elite lines were re-sequenced at an average of 17x depth with a 97.5% coverage. Over 10 million high-quality SNPs were discovered, and 35.34% of these have not been previously reported. Additionally, 159 putative domestication sweeps were identified, which includes 54.34 Mbp (4.9%) and 4,414 genes; 146 regions were involved in artificial selection during domestication. A genome-wide association study of major traits including oil and protein content, salinity, and domestication traits resulted in the discovery of novel alleles. Genomic information from this study provides a valuable resource for understanding soybean genome structure and evolution, and can also facilitate trait dissection leading to sequencing-based molecular breeding.

Legumes account for 27% of the world’s primary crop production, and the legume seeds are an essential source for food and feed and also provide nitrogen fixation through symbioses with microorganisms^1^. Soybean (*Glycine max* (L.) Merr.), a leguminous crop of major economic importance, is a main source of oil and protein^2^,^3^. The domestication history of cultivated soybean traces back to around 5,000 years ago in China^4^. Soybean was introduced to the United States (US) in the year 1765^5^, and at present soybean is the second-most planted field crop in the US. The US is currently the largest producer of soybean (34% of the global production), followed by Brazil and Argentina. The genetic diversity in soybean presumably declined to a low level due to man-made genetic bottlenecks, including selection for high yielding lines in modern plant breeding programs^6^,^7^,^8^. Additionally, the cleistogamous characteristics of soybean may have a strong influence on genomic homogeneity and reduced genomic variation, and this characteristic might become more sensitive during domestication practices^9^.

As a commercial cash crop in both the developing and developed world, the genomic information of soybean is essential for discovering traits for crop improvement. This information will help to further investigate the genetic bottle-neck that causes a change in allele frequencies and causes low genetic diversity and high linkage disequilibrium (LD), eliminating rare alleles in the selected populations/lines. In 2010, the first cultivated soybean genome was sequenced^10^. That was followed by *Glycine soja*, the undomesticated ancestor of *Glycine max*^11^. This wild soybean genome represents a 97.65% coverage of the published *G. max* genome, and the comparative genomic analysis shows significant differences between the genomic compositions of the 2 soybean lines^12^,^13^.

Advances in the next generation sequencing (NGS) technologies have made crop genome sequencing easier and more cost effective, and as a result, several crop genomes were sequenced, for example, rice (*Oryza sativa*)^14^, maize (*Zea maize*)^15^, cucumber (*Cucumis sativus*)^16^, sorghum (*Sorghum bicolor*)^17^, and common bean (*Phaseolus vulgaris*)^18^. Another key application of NGS technologies is in the re-sequencing of crop genomes at a desirable genomic equivalent depth to capture the domestication signature and any rare allelic variations^8^,^19^,^20^,^21^. Only a few re-sequencing reports are available for soybean genomes^8^,^13^,^22^,^23^,^25^. At the same time, more pan genomes, mainly from the wild soybean groups, are being sequenced^22^. The wild annual species *G. soja*, has been collected from a wide geographical distribution of a multitude of ecological and environmental conditions. Along with the wild species, soybean landraces also have an extensive geographical distribution. Adaptation to a wide variety of environmental condition makes these lines a valuable (untapped) reservoir for adaptive traits and associated genes that can be used in soybean breeding programs for various agronomic traits including stress tolerance and yield improvement.

Here, we present genomic sequences of 106 soybean accessions representing a wide variety of geographic origins, crop management techniques, and desirable traits used in the soybean research program for crop improvement. Re-sequencing was performed at a deeper level (17× average depth) than any previous reports on soybean. Comparison of genomic sequences has revealed approximately 35.34% new SNPs that have not been previously reported^9^,^13^,^25^. Our findings indicate that soybean offers unique and potential genomic resources among legume crops. Genomic comparisons of wild, landrace, and elite soybeans shows a higher percentage of putative domestication sweeps that were involved in artificial selection during domestication from landraces to elite cultivars. The soybean sequences and the corresponding data sets will be an unmatched resource for understanding genetic diversity, as well as a community tool for biological discovery and genetic improvement of soybean.

## Results and Discussion

### Landscape of genomic diversity and evaluation of variants

We selected a total of 106 soybean accessions representing different countries of origin and domestication. The collection includes seven wild, 43 landraces, and 56 elite soybean cultivars associated with different traits as well as maturity groups for whole genome re-sequencing using the Illumina platform (Supplementary Table 1). Most of these accessions were used as parents in the development of genetic mapping populations to study major agronomic and quality traits and were also used in breeding programs. Re-sequencing of the 106 soybean accessions yielded a total of 2,133 billion 90-bp length paired-end reads and a high-quality raw data of approximately 2 Tb (tera bases). The sequence reads were aligned to the soybean reference genome cultivar Williams 82, which was approximately 0.9 Gb^10^ in size using SOAP2^24^. The mapping rate in different accessions varied from 95% to 99%, averaging a 97.48% coverage and the average final effective mapping depth achieved was approximately 17x depth per accession (Supplementary Table 2). SNP calling was conducted using SOAPsnp followed by the S filtering step. A total of 10,417,285 SNPs were identified of which 9.107 million SNPs (>87%) have a rate of missing data lower than 10% (Supplementary Fig. 1), which suggests high data quality.

We analyzed the SNP position relevant to the gene annotation, and also identified non-synonymous SNPs. The majority of SNPs (82.6%) were located in intergenic regions, with only a small portion (3.83%) being located in the coding sequences (CDS) (6% of the genome); the remainder were present in the 5′ and 3′ un-translated regions (UTR) and introns (Supplementary Table 3). Lower diversity levels were found in the coding regions when compared to the introns and UTR regions. Among the coding regions, there were 161,432 synonymous SNP and 237, 560 non-synonymous SNPs, with a non-synonymous to synonymous substitution ratio of 1.47. Earlier reports in soybean^9^,^22^,^25^ also show a smaller number of synonymous SNPs compared to non-synonymous SNP substitutions. When compared to other plant species genome re-sequencing studies, the soybean non-synonymous SNP to synonymous SNP substitution ratio in wild, landraces, and elite germplasm is higher than that in other plants such as sorghum^17^ (1.0), rice^26^ (1.2) and Arabidopsis^27^ (0.83).

Total indels (equal to or less than 5 bp) were detected using the SOAPindel pipeline. A total of 745,814 indels were identified in all germplasm lines (Supplementary Table 4). Approximately 79% of the indels were located in the intergenic regions, and only 10,312 (1.38%) indels/insertions were located in the coding region. The remaining indels are present in the 5′ and 3′ UTRs and introns. Approximately 36% of indels were 1 bp shorter, and approximately 27% of insertions were 1 bp longer (Supplementary Fig. 2). Importantly, we found indels causing a frame shift in 3,464 genes, and this genetic variation data will provide a valuable resource for future investigations.

Large-effect SNPs are mutations that affect gene splicing and polypeptide chain initiation and termination. These large-effect SNPs have a major impact on gene function, and a total of 12,029 large-effect SNPs in 7,632 genes were identified, including 6,236 premature stop SNPs, 1,072 stop to non-stop code SNPs, 1,000 start to non-start code SNPs, and 3,721 splice sites SNPs (Supplementary Table 5a). This represents approximately 14% of the soybean genes that carry this mutation. This percentage is higher than previously reported in soybean^9^ and is also higher than the percentage in other plant species such as sorghum^17^ and Arabidopsis^22^. Functional classification analysis has identified 1,695 genes with large-effect SNPs belongs to biological process terms (1,061 genes), cellular component terms (69 genes), and molecular function terms (997 genes) (Supplementary Fig. 3a, 3b and Supplementary Table 5b). We also calculated the proportion of gene numbers from certain gene categories to the gene numbers in the whole genome. Surprisingly, the proportions for large-effect SNPs for these gene categories are nearly the same as the number for all genes in the whole genome, which indicates that the mutation of large-effect SNPs occurred randomly across the entire genome.

We detected gene content variation by assembling the unmapped sequences and annotating them to identify the presence and absence variations (PAVs) in different soybean lines. In total, 7,902 PAVs gene fragments longer than 1,000 bps were identified, and approximately 994 PAVs in the coding sequence have homology to plant proteins (Supplementary Table 6). We have identified 133 PAVs under the DNA metabolic process category and among them 38 PAVs are with transposable elements related function (Supplementary Table 6). This shows that majority of PAVs under “biological_process” are enriched for DNA metabolic process and that could be related to essential cellular functions. (Supplementary Fig. 4). Additionally, we have identified 146 genes that were absent in both landraces and elite germplasms and were present only in wild species. A majority of these genes are associated with metabolism and protein modification functions, including a small number of transcription factors. PAVs of specific categories of genes in wild, landraces, and elite germplasm clearly suggest the effect of a variety of selective forces on germplasm based on their different growing habitats and domestication events.

### Phylogeny and population structure of the 106 soybean lines

**Figure 1:**
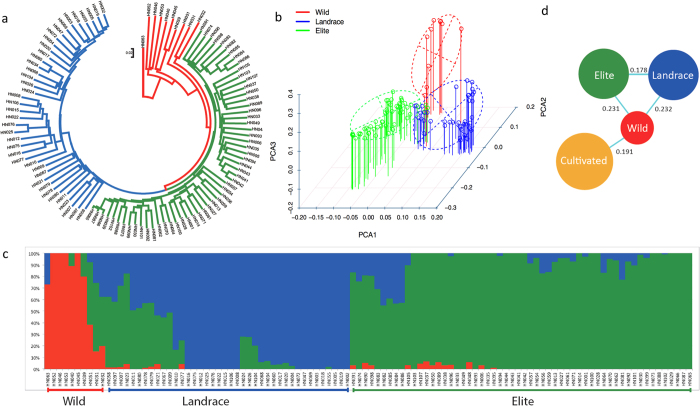
Phylogeny and population structure of soybean lines. (**a**) Phylogenetic tree constructed using SNP data. (**b**) Principal component analysis (PCA) of the 106 soybean lines. The PCA analysis was conducted using 10,417,285 SNPs. (**c**) Bayesian clustering (STRUCTURE, K = 3) of soybean accessions. (**d**) Summary of population divergence represent measures of nucleotide diversity for the group, and values between pairs indicate the population divergence (*Fst*).

To investigate the phylogenetic relationship and population structure of the selected 106 soybean lines, we generated the population structure using 79,632 4-fold degenerate-site SNPs without missing genotypes using STRUCTURE^28^,^45^ (version 2.3.1), based on allele frequencies (Fig. 1c). The 4-fold degenerate-site SNPs without amino acid changes should be under less selective pressure, thereby providing more reliability of the population structure and demography. In the STRUCTURE analysis, the different subgroups demonstrated variation in population structure, suggesting the presence of 3 groups within the 106 lines, which exactly followed our wild, landrace, and elite groups (Supplementary Fig. 5, Supplementary Table 7).

The phylogenetic relationships among 106 soybean germplasm lines were analyzed using both 4-fold degenerate-site SNPs, and all 10,417,285 SNPs were obtained from the sequence comparisons. The results of 2 SNP data sets show almost the same structure as in Fig. 1a. All of the wild soybean lines were clustered in the phylogenetic tree (Fig. 1a), and 3 soybean lines close to this group were also included. The landraces and elite lines formed separate clusters in the tree. This phylogenetic tree supports the hypothesis that the wild, landraces, and elite soybeans originated from a common ancestor. In addition, we performed a principal component analysis (PCA) using the 10,417,285 SNPs, and these 3 groups are clearly distinguished (Fig. 1b), with a compact individual clustering of a majority of landrace and elite lines separately from wild soybeans. Interestingly, all of the lines in the landrace group have a black/brown/green seed-coat color, with the exception of 3 yellow seed-coat lines [PI 507354 (HN011), PI 567357 (HN079), and PI 567383(HN080)]. A majority of the yellow seed-coat lines (48 lines) were grouped into the elite group, which indicates that the seed coat color is the most dramatically modified element under the domestication and artificial selection process. The allelic variation for the *I* locus was examined to evaluate the difference in seed coat color because the dominant forms (*I* and *i*^*i*^) of the *I* locus inhibit pigmentation of the seed coat in a spatial manner resulting in a completely yellow seed (I allele) or a yellow seed with pigmented hilum (*i*^*i*^ allele)^29^. We examined a 109.7 kb region (Gm08: 8378515–8488261) containing a cluster of chalcone synthase (CHS) genes involved in the anthocyanin pathway. We found all 3 alleles (*I, i*^*i*^, and *i*) in the form of haplogroups for seed color. The second haplogroup further divided into 2a (majority with buff hilum) and 2b (majority with black hilum). The allelic variation present in the *CHS* genes *I* locus is a major contributor for seed coat color.

All of the US cultivars were grouped into the elite group, and most of Chinese cultivars were grouped into the landrace group, which confirms the fact that the cultivated soybean was domesticated in China and then introduced to the United States. Individuals from the same geographical region tended to cluster together, which reflected isolation by distance during evolution and/or parallel selections in similar ecological habitats accompanied by gene flow.

**Figure 2:**
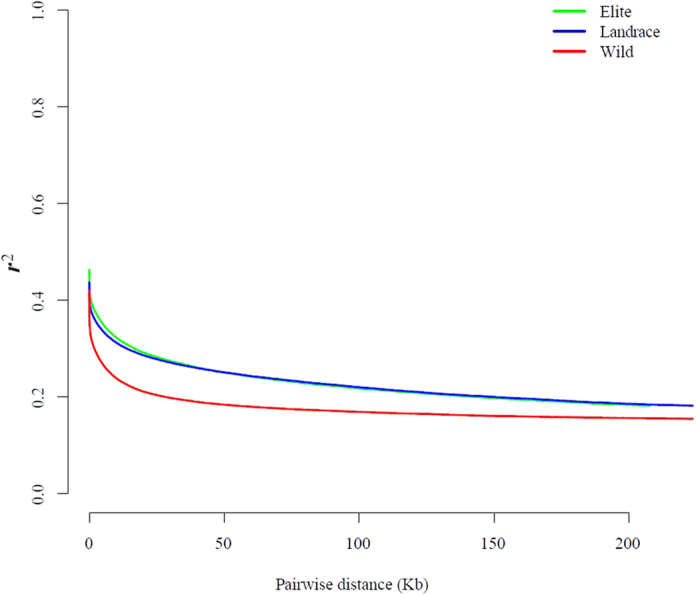
LD decay of wild, landrace and elite soybeans. LD decay determined by squared correlations of allele frequencies (*r*^*2*^) against distance between polymorphic sites in elite (green), landrace (blue) and wild (red) soybeans.

Earlier reports suggested that there would be a high LD in the soybean genomes^6^,^9^. We used Haploview 4.2 to calculate correlation coefficient values (*r*^*2*^) of alleles to measure the LD level in the 3 populations. The wild, landraces and elite germplasms showed a high LD level (Fig. 2 and Supplementary Table 8) and dropped to half of its maximum values as expected.

### Genetic divergence between wild, landrace, and elite cultivated soybeans

The genetic diversity analysis using the whole-genome SNPs, θ_π_ and θ_w_ (Table 1) showed a lower level of genetic diversity in cultivated soybeans compared to wild soybeans, and the genetic diversity decreased from wild to landraces, to elite cultivars (θ_π,_ Landrace: 1.78 × 10^−3^; Elite: 1.60 × 10^−3^; wild soybean: 2.79 × 10^−3^). The total number of SNPs and the number of non-synonymous SNPs was 15% higher in wild soybeans than in the landrace and elite groups. Also the number of large effect SNPs was nearly the same between landraces and elite groups (Supplementary Table 5a). In addition, relatively lower diversity (θπ) was observed in the CDS region with compared to intron and UTR regions (Supplementary Fig. 7).

Approximately 57% of the total SNPs (4,656,477) and almost 57% of the non-synonymous SNPs (100,821) from wild soybeans were shared with the landrace and elite groups, which indicated that approximately 43% of the SNPs were distinct between the wild and other 2 groups (Supplementary Fig. 6). This observation confirms that the cultivated soybean population had expanded after domestication and the wild soybean habitat was reduced (Table 1). Approximately 1,579, 3,139 and 3,184 PAVs were identified in the wild, landrace, and elite groups, respectively (Supplementary Table 6). We observed a lower number of PAVs in the case of wild accessions, and this might be due to the lower number of lines compared to the landrace and cultivated lines. The PAVs in wild, landrace and elite lines represent high-resolution structural diversity, and this can be further dissected to reveal the genetic basis of a major phenotypic transformation that was associated with the domestication of the soybean.

The number of SNPs, which was different between the landraces and elite cultivars (532, 573), was about one-quarter (27%) of the total number of SNPs that occurred during the domestication process (Supplementary Fig. 6). Similar patterns were also observed in the non-synonymous SNPs (approximately 23%) between these 2 groups. These observations agree with the fact that the impact of intensive selection by modern soybean breeding on the reduction of genetic diversity was less severe than that of selection by the domestication process^13^. This finding suggests that the wild soybean gene pool is a very valuable resource, and tapping into this genomic resource will have a significant impact on the development of improved soybean cultivars.

### Domestication in the soybean genome

**Figure 3:**
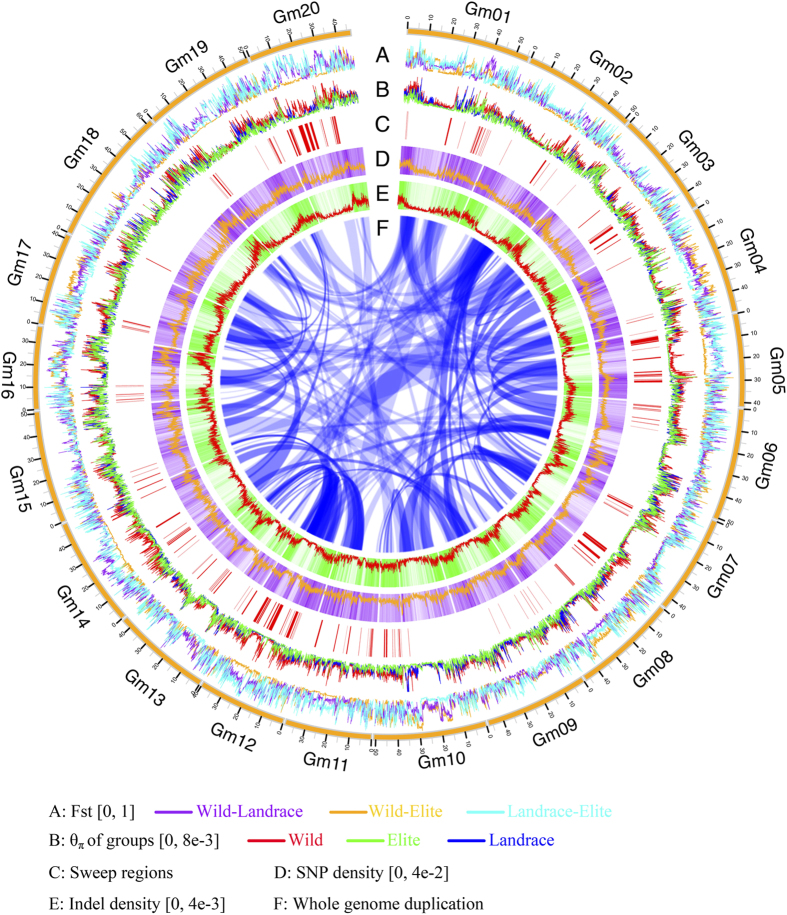
Summary of resequencing data of 106 soybean germplasm.

**Figure 4:**
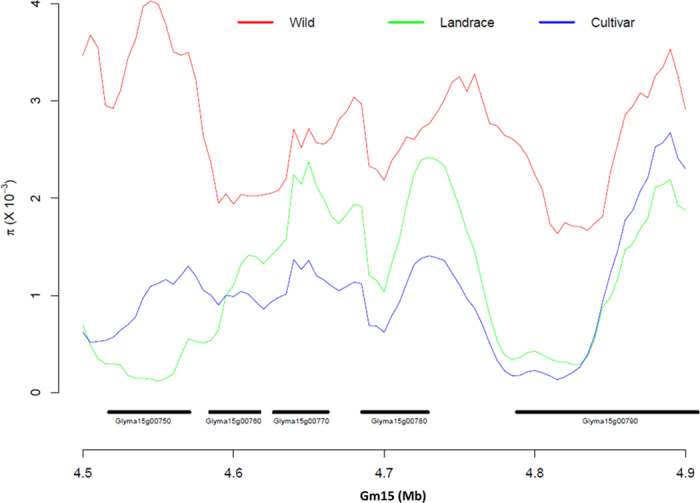
Genetic diversity (π) of a domestication region. Diversity pattern in Gm15 for *G. soja*, landrace and elite cultivars associated with yield and flower number related QTLs^48^ (4.5–4.9 Mb). Previously this region was identified as a large QTL (3.1–6.7 MB) and contained 47 genes. In the present study this region narrow down to 4.5–4.9 Mb and contains 5 genes, in which *Glyma15g00750* showed relatively lower π in cultivated soybean, and functionally this gene is annotated to bHLH/Circadian-protein, suggesting that this gene might be involved in yield-related traits in cultivated soybean.

The divergence index value (*Fst*) between wild, landrace, and elite soybean was calculated. This measurement helped to localize genomic regions with a higher degree of diversification between wild-landrace, wild-elite and landrace-elite soybeans (Fig. 3; Supplementary Fig. 9). These large *Fst* value regions may be harboring domestication-related loci. We analyzed genomic region with extreme pattern of diversity (π) (for example Chr.15, Fig. 4) that was associated with domestication related QTL, such as flower number and yield. The diversity in this genomic region was lower in elite and landrace when compared to wild (Fig. 4).

We combined the landrace and elite groups as a single cultivated gene pool to identify the domestication sweeps. The regions and genes under domestication sweeps should have significantly lower diversity in cultivated soybeans compared to wild soybeans. The ratio of genetic diversity by comparing the wild group to cultivated groups (π_w_/π_c_) in 50-kb sliding windows with a step size of 5 kb was used to identify regions with significantly lower levels of polymorphisms in the cultivated groups. We have identified 159 putative domestication sweeps, which include 54.34 Mbp (5.7%) and 4,414 genes.

Soybean QTL information available in Soybase (www.soybase.org) was gathered to develop a gene list containing 15,502 genes associated with 96 QTL regions for the most important soybean agronomic traits. These genes were used for the diversity analysis. Approximately 22 sweep regions (496 genes underlying 26 QTL) were overlapped in the 15,502 gene regions (Supplementary Table 9). We identified the region that had the highest π_w_/π_c_ value. Interestingly, 20 genes with the highest π_w_/π_c_ values from the 27.82 Mbp to 28.55 Mbp region in chromosome Gm01 (Supplementary Fig. 8) were located in the QTL region for seed weight^30^,^31^. Moreover, 44 genes that have the second highest π_w_/π_c_ value from the 22.31 Mbp to 24.26 Mbp region in Gm12 were reported in the QTL region for soybean yield^32^. Overall, these sweep regions will provide key information for future soybean breeding and selection.

### Artificial selection in the soybean genome

We applied same analysis method of the sweep regions in the artificial selection process between the landrace and elite groups. The regions and genes under artificial selection should have a significantly lower diversity in elite compared to landrace soybeans. Additionally, 146 regions (32.16 Mbp, 2.9% and 2,542 genes) were identified. Approximately 15 artificial selection regions (270 genes) were associated with a QTL region of major traits when compared to the list of 15,502 genes (Supplementary Table 10).

### Analysis of specific traits

#### Maturity

The maturity of plants depends upon the complex and coordinated regulation of photoreceptors, floral meristem, and flowering time genes as well as geographical distribution^35^,^36^,^37^. We examined the natural variation in 106 PI lines for known maturity (E1–E4)^35^,^36^ and plant architecture^37^ (Dt) genes (Supplementary Table 11). The functional analysis of mutant alleles for maturity genes showed an early flowering time phenotype^34^. The haplogroup analysis of maturity and plant architecture genes showed a strong correlation, and distinct clusters were associated with growth habits and geographic origin (Supplementary Fig. 10 A–E). The analysis of E1–E4 genes (gene plus 3.5 kb upstream/downstream regions) found that the lower maturity group (MG) lines (MG-0, −I, −II) retain one or more mutant allele (Supplementary Table 12). Interestingly, Fiskeby-III originated from Sweden, belonged to MG-0, and showed an entire gene deletion for the E1 locus, leading to the *e1-nl* allele. In addition, this line showed all of the major mutant alleles for other maturity (E2 and E3) and Dt genes (Supplementary Fig. 10 A–E). Assessment of maturity and Dt gene for each line is presented in Supplementary Fig. 10 and Supplementary Table 12.

#### Acyl-lipid metabolism

We have selected a subset of soybean lines based on their contrasting total seed oil content, and they showed a wide range of oil content from 5–24%. Lines, based on oil content, were categorized as low oil lines (5 to 16.9%), moderate oil lines (17 to 19.9%), and high oil lines (20 to 24%), comprising 26, 38, and 37 lines respectively. All the US PIs from elite backgrounds contain more than 19% oil. In contract, most of the wild relatives contain less than 14% oil, except for 2 lines (Supplementary Table 13). Candidate genes involved in acyl lipid metabolism were selected from diverse data sets such as the soybean acyl-lipid dataset^38^, the Meta-QTL dataset, and the RNAseq dataset. We focused our subsequent analysis to identify the allelic variation in acyl-lipid genes and found a wide variation in non-syn-SNPs, indels, and CNVs. A total of 1,686 non-syn-SNPs were identified; out of those, 150 were associated with high and low oil lines that were significantly enriched for biological process of wax biosynthesis (WBT), fatty acid synthesis (FAS), and tri acyl glycerol biosynthesis and degradation (TAG_FAD, TAGB). To better define the allelic variation, we further narrowed down the search in 15 PIs lines with high and low oil content. We also found 40 SNPs in the candidate genes leading to premature stop codons (large effect SNPs). Analysis of the copy number variation suggested that approximately 23% of the predicted acyl-lipid genes in soybean had CNVs (Supplementary Table 14). The comparative analysis between high and low oil lines showed an increased copy number of lipid transfer protein (LPT; *Glyma16g31780, Glyma16g31840*, and *Glyma16g31540*) in high oil lines, whereas the negative regulators (ABC transporter, *Glyma03g36310*; Lipase 3, and *Glyma13g04561*) of lipid biosynthesis genes showed more copies in low oil lines (Supplementary Fig. 11a). The differential variation in SNP and copy number for lipid biosynthesis and lipid degradation genes between high oil and low oil lines might be associated with the differences in oil content within the PI lines. Recently, Wang *et al*.^39^ compared the sesame (55% oil) acyl-lipid genes with soybean and concluded that in families encoding lipid transfer proteins (LTP), midchain alkane hydroxylase was expanded by tandem duplication in sesame, whereas soybean has fewer numbers of those gene families.

#### Protein content

Similar to acyl-lipid, the allelic variation in seed protein related genes was investigated. Seed protein related genes were selected from the known QTL that is associated with protein content reported in Soybase.org and from the molecular dissection of QTL I^40^ and GWAS studies^41^. We found 42 SNPs in 21 genes that were associated with protein content. The genomic block on Gm20 (24.5 to 32.9 Mbp, 8.1 Mbp) is defined as a major protein QTL for protein content^40^,^41^, and we found that the copy number variations in candidate genes (HSP, *Glyma20g19680*; ammonium transporter, *Glyma20g21030*; ethylene receptor, and *Glyma20g21780*) were associated with protein content (Supplementary Table 15; Supplementary Fig. 11b). The 2.4 Mbp genomic region (within 8.1 Mbp) defined in the GWAS study^41^ was further studied to determine haplotype variation, and we found 3 major clusters that were significantly associated with protein content.

#### Salinity tolerance genes

Recently Qi *et al*.^23^ identified the salt tolerance gene *CHX1* (ion transporter) in wild soybean using a combination of WGS approaches, and showed *CHX1* gene is associated with salt tolerance. The *GmCHX1* gene (*Glyma03g32900*) possesses an approximately 3.3 kb Ty1/copia retrotransposon in the third exon, leading to a sensitive phenotype, and another variant without a retrotransposon leads to a tolerant phenotype. In the present investigation, we studied the CHX1 locus to verify the allelic variation in the 106 soybean genomes. Alignment of 13 kb consensus genomic region in the whole genome sequences of 106 soybean lines resulted in 3 major groups G1, G2, and G3. The groups G2 and G3 were reported by Qi *et al*.^23^, and one new variant, G1, was discovered in the present study (Supplementary Fig. 12). The presence of the Ty1/copia retrotransposon in the G2 lines confirmed that they carry the same allele as the sensitive lines (C08 and W82); however, the absence of the retrotransposon in G3 confirmed that they carry same allele as the tolerant group (W05). The tolerant group G3 includes previously known salt tolerant lines such as S-100^42^, FiskebyIII and the PI 483463^43^. Interestingly, lines belonging to G1, including cv. Hutcheson (Supplementary Table 1, PI 518664), do not retain the Ty1/copia retrotransposon; however, those lines carry 12 SNPs in the 1 Kb upstream promoter region, and deletions in exon 3 and part of 1^st^ and 2^nd^ intron result in their inclusion in the sensitive group. These variants can be confirmed by phenotyping and association with allele specific marker assays. The tolerant line, S-100, is the ancestor of popular cultivars in the US germplasm^42^, and this suggests that the tolerant gene from S-100 could be the main source of salt tolerance in the US soybean cultivars for late maturity groups (IV–VI), and cv. FiskebyIII for the early maturity group. Overall, the structural variation in *CHX1* gene reconfirms that *GmCHX1* is the major gene controlling salt tolerance in soybean via ion homeostasis.

## Summary

Soybean yield improvement is changing at a slower pace and this major challenge needs to be addressed to meet the global demand for food, feed and fiber. Natural genetic variation analysis of soybean germplasm using the genome sequence information of individuals will greatly help to identify genes and rare alleles that are associated with major traits and components for soybean improvement. In the present investigation, we provided a significant genomic variation data set for wild, landrace, and elite soybean lines. Deeper sequencing, high-quality data, and millions of SNPs in the representative soybean lines provide an unprecedented opportunity for us to understand the landscape of soybean diversity and domestication. We identified a number of candidate genes and genetic loci that may have been selected as hot spots of domestication in soybean. Our population structural analysis and phylogenetic analysis support the common hypothesis that soybean was domesticated in the China subcontinent and then introduced to the US and other parts of the world.

Our findings showed that the SNP variation and PAV between the wild and elite lines were 43% and 50%, respectively; and this finding explains the diversification of elite lines after domestication based on climatic zones, plant architecture, and agronomic traits. The genes identified in the domestication sweep regions were also correlated with the previously identified traits. We also studied agronomically important traits, such as oil and protein content, maturity, plant architecture, seed coat color, and salinity, and provide genomic variation information that can be used for developing trait specific genetic markers. Genetic and genomic resources generated in this study will provide a strong foundation for trait discovery, next generation breeding tools, and genomic selection strategies for the development of improved soybean cultivars.

## Methods

### Sequencing and mapping

We extracted genomic DNA from 106 soybean lines, constructed paired-end (PE) sequencing libraries with insert sizes of approximately 500 bp, and generated approximately 2 Tb of clean data after filtering low quality reads and duplicate reads. The soybean reference genome and annotation were downloaded from ftp://ftp.jgi-psf.org/pub/JGI_data/phytozome/v7.0/Gmax. We joined the unanchored scaffolds to a pseudo-chromosome “GmU” then PE reads of each line were independently mapped to the reference genome using the program SOAP2 (http://soap.genomics.org.cn/soapaligner.html). The parameters were set as “-m 100 -x 888 -s 35 -l 32 -v 3 -p 4”, 3 mismatches were allowed in a single read while mapping to the genome. The output of mappings were split into chromosomes, and sorted by the ninth column i.e., location of first bp on the reference. Approximately 95 to 99% of the reads were mapped, which covered 97.48% of the genome at a 17× depth. All of the mapping results were used to detect variation.

### Variation detection

#### SNP

Consensus sequence for the genome of each line was assembled based on Bayes’ theorem using the program SOAPsnp 1.04 (http://soap.genomics.org.cn/soapsnp.html) with parameters “-L 100 -u -F 1”. Based on the maximum-likelihood estimation of site frequency, we used GLF multi to generate the mapping depth, allele, allele frequency and quality of each site using the consensus sequence information from 106 lines. Sites were filtered by “>50x and < 3000x” in depth, “>15” in quality and “<1.5” in copy-number. SOAPsnp outputs were used to obtain accurate genotype SNPs of the filtered sites. We generated a final SNP set containing 10,417,285 SNPs after removing the sites with the heterozygote allele ratio larger than 0.88, or the missing allele ratio larger than 0.88.

#### Indel

All of the reads were mapped to the reference using a parameter “–g 5”, which allowed a less than 5 bp gap within the hit of one single read to detect small insertions and deletions. Indels (1–5bp) were called by the SOAPindel 1.09 (http://soap.genomics.org.cn/soapindel.html) by “-m 1 -p 0.01 -k 5 -c 3 -h 0.5”.

#### PAV

For PAV detection, the unmapped reads were collected and assembled using SOAPdenovoV2.04 (parameters pregraph −K 63 −R; contig −R), and filtered for scaffolds with lengths less than 1 kb. Retained scaffolds were then annotated using the mapping protein to genome method based on all available proteins, and the gene set was generated using *genewise*. Those genes with paralogs in the soybean gene sets with >80% identity and >80% coverage at the nucleotide level were filtered out by BLAST. Finally, we identified 2,399 genes as PAV genes, and these genes were used to conduct functional annotation.

#### CNV

Copy number variations were detected according to the depth distribution of each line, having a minimum length of >2 kb, with a mean depth of less than half of the sequence depth, or larger than double of the sequence depth. The initial and final minimum probability to merge the adjacent breakpoints were set to 0.5 and 0.8, respectively.

### Population analysis

#### Structure

The program STRUCTURE 2.3.1^44^ was used to determine the population structure of the 106 soybean lines assuming different numbers of clusters (K = 2, …6). To obtain a best K value, we selected 1,000 4-fold degenerate SNPs randomly to calculate the distribution of ‘delta K’^45^ and found the mostly like number of clusters of: K = 3 (Supplementary Fig. 5).The population structure is shown in Fig. 1c.

#### Phylogenetic tree

Using a subset of 79,632 4-fold degenerate SNPs, a phylogenetic tree was constructed using neighbor (Version: EMBOSS:6.6.0.0) based on the Neighbor-joining method^46^.

#### Principal component analysis

PCA of the SNPs was performed using the software EIGENSOFT^47^, with the population structure determined by the STRUCTURE program.

#### SNP diversity and F_ST_

The average pairwise divergence within a population (*θ*_*π*_), the Watterson’s estimator (*θ*_*w*_) and Tajima’s D were estimated for the whole genome of 3 soybean populations using a sliding windows of 50 kb. In each window, these parameters were calculated using the Bio::PopGen package of PERL and *F*_*ST*_ was calculated to measure the differentiation between the two populations.

#### Linkage Disequilibrium decay

We used Haploview. 4.2 to calculate correlation coefficient values (r^2^) of alleles to measure the LD level in 3 populations. The parameters were set as: -maxdistance 250 -minMAF 0.1 -hwcutoff 0.001 -dprime -memory 2,096. The average r^2^ value was calculated for each length of distance and LD decay figures were drawn using an R script.

#### Domestication sweeps

We combined the landrace group and elite group into a single cultivated gene pool to detect the regions and genes underlying domestication sweeps. These regions should have lower diversity in the cultivated group compared to the wild group. Thus, by comparing the wild group to the cultivated group (π_w_/π_c_) in 50-kb sliding windows, the ratio of genetic diversity was used to identify regions with lower levels of polymorphisms in cultivated groups. After removing the windows lower than 0.002 π_w_, windows in the top 5% of π_w_/π_c_ values were considered as candidate regions with significantly lower diversity in cultivated lines, and windows with a distance of ≤50 kb were merged into a single selected locus.

## Additional Information

**Accession code**: The sequencing data have been deposited in the NCBI Short Read Archive under the accession code SRP062245.

**How to cite this article**: Valliyodan, B. *et al*. Landscape of genomic diversity and trait discovery in soybean. *Sci. Rep.* **6**, 23598; doi: 10.1038/srep23598 (2016).

## Supplementary Material

Supplementary Information

## Figures and Tables

**Table 1:** Summary of SNP statistics in soybean genotypes and the distribution of SNPs in whole genome and genic regions.

	Total	θ_π_ (10^−3^)	θ_w_ (10^−3^)	Intergenic	Intron	5′-UTR	3′-UTR	Exon			
								Total	Nonsynonymous*	Synonymous	Nonsyn/Syn
**Wild**	8,106,944	2.79	2.34	6,688,631	891,196	86,445	134,627	306,045	177,729	128,485	1.38
**Landrace**	7,022,002	1.78	1.49	5,812,993	753,093	76,649	112,834	266,433	158,476	108,082	1.47
**Elite**	7,148,434	1.6	1.4	5,921,685	764,306	77,156	113,474	271,813	162,697	109,271	1.49
**Landrace & Elite**	8,430,864	1.9	1.49	6,989,257	898,983	90,548	133,771	318,305	191,266	127,215	1.50

^*^SNPs located in overlapping region of different transcripts were annotated independently. Some SNPs are synonymous SNPs in one transcript, and concurrently non-synonymous SNPs in another overlapping transcript, and vice versa. Thus, the sum of synonymous and non-synonymous SNPs is more than the number of SNPs in the CDS regions.

## References

[b1] Gepts P. . Legumes as a model plant family. Genomics for food and feed report of the cross-legume advances through genomics conference. Plant Physiol. 137, 1228–1235 (2005). 15824285 10.1104/pp.105.060871 PMC1088316

[b2] Wilcox J. R. World distribution and trade of soybean. In: Boerma H.R. & Specht J.E., ed., Soybeans: Improvement, Production and Uses, p 1–14, American Society of Agronomy, Madison, WI, USA, (2004).

[b3] Valliyodan B. & Nguyen H.T. Biological mechanisms that influence soy protein concentration and composition. In: Wilson R. F. ed., Designing Soybeans for the 21st Century Markets. AOCS, IL, USA, (2012).

[b4] Hymowitz T. On the domestication of soybean. Econ. Bot. 24, 408–421 (1970).

[b5] Hymowitz T. & Harlan J.R. Introduction of soybean to North America by Samuel Bowen in 1765. Econ. Bot. 37, 371–379 (1983).

[b6] Hyten D.L. . Highly variable patterns of linkage disequilibrium in multiple soybean populations. Genetics 175, 1937–1944 (2007). 17287533 10.1534/genetics.106.069740 PMC1855121

[b7] Hyten D.L. . Impacts of genetic bottlenecks on soybean genome diversity. Proc. Natl. Acad. Sci. USA 103, 16666–16671 (2006). 17068128 10.1073/pnas.0604379103 PMC1624862

[b8] Rincker K. . Genetic improvement of US soybean in maturity groups II, III, and IV. Crop Sci. 54, 1–14 (2014).

[b9] Lam H.M. . Resequencing of 31 wild and cultivated soybean genomes identifies patterns of genetic diversity and selection. Nat. Genet. 42, 1053–1059 (2010). 21076406 10.1038/ng.715

[b10] Schmutz J. . Genome sequence of the palaeopolyploid soybean. Nature 463, 178–183 (2010). 20075913 10.1038/nature08670

[b11] Kim M.Y. . Whole-genome sequencing and intensive analysis of the undomesticated soybean (*Glycine soja* Sieb. and Zucc.) genome. Proc. Natl. Acad. Sci. USA 107, 22032–22037 (2010). 21131573 10.1073/pnas.1009526107 PMC3009785

[b12] Joshi T. . Genomic differences between cultivated soybean, G. max and its wild relative G. soja. BMC Genomics 14, S1–S5 (2013). 10.1186/1471-2164-14-S1-S5 PMC3549820 23368680

[b13] Li Y.H. . Molecular footprints of domestication and improvement in soybean revealed by whole genome re-sequencing. BMC Genomics 14, 579 (2013). 23984715 10.1186/1471-2164-14-579 PMC3844514

[b14] International Rice Genome Sequencing Project. The map-based sequence of the rice genome. Nature 436, 793–800 (2005). 16100779 10.1038/nature03895

[b15] Schnable P.S. . The B73 maize genome: Complexity, diversity, and dynamics. Science 326, 1112–1115 (2009). 19965430 10.1126/science.1178534

[b16] Huang S. . The genome of the cucumber, Cucumis sativus L. Nat Genet. 41, 1275–1281 (2009). 19881527 10.1038/ng.475

[b17] Paterson A.H. . The Sorghum bicolor genome and the diversification of grasses. Nature 457, 551–556 (2009). 19189423 10.1038/nature07723

[b18] Schmutz J. . A reference genome for common bean and genome wide analysis of dual domestications. Nat Genet. 46, 707–713 (2014). 24908249 10.1038/ng.3008 PMC7048698

[b19] Lim J.H. . Quantitative trait locus mapping and candidate gene analysis for plant architecture traits using whole genome re-sequencing in rice. Mol Cell 37, 149–160 (2014). 10.14348/molcells.2014.2336 PMC3935628 24599000

[b20] Wang Z.H. . Genome wide variation in an introgression line of rice-Zizania revealed by whole-genome re-sequencing. PLoS One. 8, e74479 (2013). 24058573 10.1371/journal.pone.0074479 PMC3776793

[b21] Mace E.S. . Whole-genome sequencing reveals untapped genetic potential in Africa’s indigenous cereal crop sorghum. Nat Commun. 4, 2320 (2013). 23982223 10.1038/ncomms3320 PMC3759062

[b22] Li Y.H. . De novo assembly of soybean wild relatives for pan-genome analysis of diversity and agronomic traits. Nat Biotechnol. 32, 1045–52 (2014). 25218520 10.1038/nbt.2979

[b23] Qi X. . Identification of a novel salt tolerance gene in wild soybean by whole-genome sequencing. Nat. Commun. 5, 4340 (2014). 25004933 10.1038/ncomms5340 PMC4104456

[b24] Liu C.M. . SOAP3: ultra-fast GPU-based parallel alignment tool for short reads. Bioinformatics 28, 878–879 (2012). 22285832 10.1093/bioinformatics/bts061

[b25] Zhou Z. . Resequencing 302 wild and cultivated accessions identifies genes related to domestication and improvement in soybean. Nat Biotechnol. 33, 408–414 (2015). 25643055 10.1038/nbt.3096

[b26] McNally K. L. . Genome wide SNP variation reveals relationships among landraces and modern varieties of rice. Proc. Natl Acad. Sci. USA 106, 12273–12278 (2009). 19597147 10.1073/pnas.0900992106 PMC2718348

[b27] Clark R. M. . Common sequence polymorphisms shaping genetic diversity in Arabidopsis thaliana. Science 317, 338–342 (2007). 17641193 10.1126/science.1138632

[b28] Pritchard J.K. . Inference of Population structure using multilocus genotype data. Genetics 155, 945–959 (2000). 10835412 10.1093/genetics/155.2.945 PMC1461096

[b29] Tuteja J. H. . Endogenous, tissue-specific short interfering RNAs silence the Chalcone Synthase gene family in Glycine max seed coats. Plant Cell 21, 3063–3077 (2009). 19820189 10.1105/tpc.109.069856 PMC2782299

[b30] Sun Y. N. . Multi-environment mapping and meta-analysis of 100-seed weight in soybean. Mol. Biol. Rep. 39, 9435–9443 (2012). 22740134 10.1007/s11033-012-1808-4

[b31] Pathan S.M. . Genetic mapping and confirmation of quantitative trait loci for seed protein and oil contents and seed weight in soybean. Crop Sci. 53, 765–774 (2013).

[b32] Chung J. . The Seed Protein, Oil, and Yield QTL on Soybean Linkage Group I. Crop Sci. 43, 1053–1067 (2003).

[b33] Langewisch T. . Major soybean maturity gene haplotypes revealed by SNPViz analysis of 72 sequenced soybean genomes. PloS One 9, 4 (2014). 10.1371/journal.pone.0094150 PMC3984090 24727730

[b34] Xia Z. . Positional cloning and characterization reveal the molecular basis for soybean maturity locus E1that regulates photoperiodic flowering. Proc. Natl Acad. Sci. USA 109, 2155–2164 (2012). 10.1073/pnas.1117982109 PMC3420212 22619331

[b35] Liu B, . Genetic redundancy in soybean photoresponses associated with duplication of the phytochrome A gene. Genetics 180, 995–1007 (2008). 18780733 10.1534/genetics.108.092742 PMC2567397

[b36] Watanabe S. . Map-based cloning of the gene associated with the soybean maturity locus E3. Genetics 182, 1251–1262 (2009). 19474204 10.1534/genetics.108.098772 PMC2728863

[b37] Tian Z. . Artificial selection for determinate growth habit in soybean. Proc. Natl Acad. Sci. USA 107, 8563–8568 (2010). 20421496 10.1073/pnas.1000088107 PMC2889302

[b38] Yu J. . SFGD: a comprehensive platform for mining functional information from soybean transcriptome data and its use in identifying acyl-lipid metabolism pathways. BMC Genomics, 15, 271 (2014). 24712981 10.1186/1471-2164-15-271 PMC4051163

[b39] Wang L. . Genome sequencing of the high oil crop sesame provides insight into oil biosynthesis. Genome Biology 15, 39 (2014). 10.1186/gb-2014-15-2-r39 PMC4053841 24576357

[b40] Bolon Y.T. . Complementary genetic and genomic approaches help characterize the linkage group I seed protein QTL in soybean. BMC Plant Biol. 10, 41 (2010). 20199683 10.1186/1471-2229-10-41 PMC2848761

[b41] Hwang E.Y. . A genome-wide association study of seed protein and oil content in soybean. BMC Genomics 15, 1 (2014). 24382143 10.1186/1471-2164-15-1 PMC3890527

[b42] Lee G.J. . A major QTL conditioning salt tolerance in S-100 and descendent cultivars. Theor Appl Genet 109, 610–1619 (2004). 10.1007/s00122-004-1783-9 15365627

[b43] Lee J.D. . Inheritance of salt tolerance in wild soybean (*Glycine soja* Sieb. and Zucc.) accession PI 483463. J. Heredity 100, 798–801 (2009). 10.1093/jhered/esp027 19451208

[b44] Pritchard J.K. . Inference of population structure using multilocus genotype data. Genetics 155, 945–959 (2000). 10835412 10.1093/genetics/155.2.945 PMC1461096

[b45] Evanno C. . Detecting the number of clusters of individuals using the software STRUCTURE: a simulation study. Mol. Ecol. 14, 2611–2620 (2005). 15969739 10.1111/j.1365-294X.2005.02553.x

[b46] Price A.L. . Principal components analysis corrects for stratification in genome-wide association studies. Nature Genet. 38, 904–909 (2006). 16862161 10.1038/ng1847

[b47] Patterson N. . Population structure and eigenanalysis. PLoS Genet. e190 (2006). 10.1371/journal.pgen.0020190 PMC1713260 17194218

[b48] Zhang D., Cheng H., Wang H., Zhang H., Liu C. & Yu D. Identification of genomic regions determining flower and pod numbers development in soybean (Glycine max L.). J. Genet. Genomics 37, 545–556 (2010). 20816387 10.1016/S1673-8527(09)60074-6

