# Supplementary material for: Landscape of genomic diversity and trait discovery in soybean
Source: Sci Rep. 2016 Mar 31;6:23598. doi: 10.1038/srep23598 (PMC4814817; doi:10.1038/srep23598)

Landscape of genomic diversity and trait discovery in soybean

Babu Valliyodan[1*], Dan Qiu[1*], Gunvant Patil[1*], Peng Zeng[2*], Jiaying Huang[2*], Lu Dai[2*], Chengxuan Chen[2*], Yanjun Li[2], Trupti Joshi[3,4], Li Song[1], Tri D. Vuong[1], Theresa A. Musket[1], Dong Xu[3], J. Grover Shannon[5], Cheng Shifeng[2], Xin Liu[2], and Henry T. Nguyen[1]

[1]Division of Plant Sciences and National Center for Soybean Biotechnology (NCSB), University of Missouri, Columbia 65211, USA; [2]Beijing Genomics Institute-Shenzhen, Shenzhen, 518083, China; [3]Department of Molecular Microbiology and Immunology and Medical Research Office, School of Medicine, University of Missouri, Columbia, 65212; [4]Department of Computer Science, Informatics Institute and Christopher S. Bond Life Sciences Center, University of Missouri, Columbia, 65211, USA, [5]Division of Plant Sciences and NCSB, University of Missouri-Fisher Delta Research Center, Portageville, MO, 63873, USA.

[*]These authors contributed equally to this work.

Corresponding authors:

Xin Liu

Beijing Genomics Institute-Shenzhen, Shenzhen, 518083, China

Email: liuxin@genomics.cn; Phone: +86-186-6629-0227

&

Henry T. Nguyen

Division of Plant Sciences and National Center for Soybean Biotechnology (NCSB), University of Missouri, Columbia MO, 65211, U.S.A

Email: nguyenhenry@missouri.edu; Phone: +1-573-882-5494

Supplementary Tables:

Supplementary Table 1. List of the 106 soybean germplasm lines sequenced with sequence identifier.

Supplementary Table 2. Summary of soybean accession sequence coverage and mean depth of resequencing.

Supplementary Table 3. Distribution of SNPs in different chromosomes of 106 soybean accessions.

Supplementary Table 4. Distribution of indels within various soybean genomic regions.

Supplementary Table 5 a. Distribution of large effect SNPs in three soybean gene pools. **b.** GO enrichment cluster of genes with large effect SNPs.

Supplementary Table 6. Region of Present Absent Variation (PAV) in three gene pools (wild, landrace and elite) and gene list.

Supplementary Table 7. Population structure analysis of soybean germplasm.

Supplementary Table 8. Distribution of LD blocks in three soybean gene pools.

Supplementary Table 9. Gene list (496 genes) from the sweep region harboring the QTL associated with major traits.

Supplementary Table 10. Gene list (270 genes) of the artificial selection region harboring QTL associated with major traits.

Supplementary Table 11. Candidate genes associated with maturity and growth habit in soybean.

Supplementary Table 12. **Allelic variation for maturity genes in soybean**. a: Allelic variation for maturity and determinate/indeterminate growth loci. b: Details of major maturity genes used for haplogroup analysis.

Supplementary Table 13. Total oil and protein content in 106 soybean lines.

Supplementary Table 14. Copy number variation on genes associated with oil traits in soybean.

Supplementary Table 15. Copy number variations in total protein trait related genes.

Supplementary Figures:

Supplementary Figure 1. Missing rate of SNPs for 106 soybean germplasm sequences. Proportion of 106 individuals with missing genotype at each SNP loci was calculated. Distribution of the missing genotype proportions was shown in this figure. Majority of the SNPs had low missing rate.

Supplementary Figure 2. Distribution rate of indels. Distribution rate of small insertion and deletions (indels) in different genomic regions. All identified indels are shorter than or equal to 5 bp.

Supplementary Figure 3. Gene ontology (GO) analysis of large effect SNPs genes within biological process, cellular component and molecular function. a. Gene ontology (GO) of each Large effect SNPs genes were obtained by the annotation of gene functions using GO database. The blue bar is the ratio of certain category of genes number in the whole genome to the whole category gene numbers. **b.** GO enrichment analysis of large effect SNPs genes were calculated by Cytoscape 2.8 using the plugins BiNGO-2.44.

Supplementary Figure 4. Gene ontology (GO) analysis of Present/Absent Variation (PAV) genes within biological process, cellular component and molecular function. Gene ontology (GO) of each PAV genes were obtained by the annotation of gene functions using GO database.

Supplementary Figure 5. Bayesian clustering analysis of all individual (STRUCTURE, K=3; K=4; K=5; and K=6).

Supplementary Figure 6. Comparison of (a) Total SNPs and (b) non-synonymous SNPs in the three soybean gene pools (wild, landrace and elite cultivar).

Supplementary Figure 7. Comparison of the diversity (θπ) levels across genic and non-genic regions. Coding regions displayed lower diversity levels relative to intron and UTR regions. The genic regions include both coding and non-coding regions of the genes (mRNA, CDS, UTR and intron), in comparison to the genic and non-genic regions genome-wide.

Supplementary Figure 8. Distribution of Genomic diversity (θ_π) for wild (red), landrace (blue), elite soybeans (green) and cultivated (orange) in 20 soybean chromosomes.

Supplementary Figure 9. Frequency distribution of Genomic diversity (θ_π) and Divergence index value (*Fst*). Both *Fst* and θ_π between wild, landrace and elite soybean was calculated.

Supplementary Figure 10a-e. Haplotype analysis of Maturity genes. A- *E1*, B-*E2*, C-*E3*, D-*E4*, E-*Dt1*. SNPviz software was used to draw pictorial clusters. Base position identical to reference (W82) are white, black – different, gray-missing data.

Supplementary Figure 11a. **Diversity and allelic variation of lipid biosynthesis genes**. Example showing the allelic variation of lipid biosynthesis related genes associated with a QTL controlling oil content. (a) The *Fst* between wild, landrace and elite soybean; (b) Predicted gene models in the genomic block on Chromosome 13 (4-89-11.49 Mb); (c) Comparative analysis of amino acid polymorphism in the gene *Glyma13g04571.1*, a negative regulator of lipid biosynthesis.

Supplementary Figure 11b. **Diversity and allelic variation of genes associated with protein content.** Example showing the allelic variation of seed protein related genes associated with a QTL controlling protein content. (a) The *Fst* between wild, landrace and elite soybean; (b) The genomic block on chromosome 20 (24.5 to 32.9 Mbp, 8.1 Mbp) is defined as major protein QTL for protein content.

Predicted gene models in this genomic region; (c) Comparative analysis of amino acid polymorphism in the gene HSP, *Glyma20g19680*; Ammonium transporter, *Glyma20g21030*; ethylene receptor, *Glyma20g21780* associated with protein content.

Supplementary Figure 12a-b. Gene structure variation of the salt tolerance gene *CHX1* in 106 soybean lines.

a. Phylogenetic clustering of *CHX1* locus (gene + 2 kb promoter region) preceded three major clades. Samples were grouped by their sequence identity to the reference (W 82). W05 and C08 were used for comparing salt-tolerant and salt sensitive genotypes, respectively[23]. Known indicator lines are marked with arrows. **b.** *CHX1* gene architecture in 106 lines. Dotted lines indicating the exon position. Red lines indicating that this region is deleted in group 1 (G1). G2 and G1 group indicates the sensitive while G3 indicates salt tolerant group. However, no deletion was tested in the exon at the G1 group.

Supplementary Table 1. List of the 106 soybean germplasm lines sequenced with sequence identifier.

Genotypes	Sequence identifier	Country of Origin	MG	Determinate	Flower Color	Pubescence Color	Seed Color	Hilum Color
PI 518664 (Hutcheson)	HN001	U.S.	V	Determinate	White	Gray	Yellow	Buff
PI 548402 (Peking)	HN002	China	IV	Determinate	White	Tawny	Black	Black
PI 089772	HN003	China	IV	Non-determinate	Purpler	Tawny	Black	Black
PI 090763	HN004	China	IV	Non-determinate	Purple	Light tawny	Black	Black
PI 404166	HN005	China	III	Non-determinate	Purple	Tawny	Black	Black
PI 407788A	HN006	South Korea	IV	Determinate	Purple	Gray	Yellow	Buff
PI 424298	HN007	South Korea	IV	Non-determinate	Purple	Tawny	Black	Black
PI 437655	HN008	China	III	Non-determinate	Purple	Light tawny	Black	Black
PI 495017C	HN009	China	IV	Non-determinate	Purple	Tawny	Green	Black
PI 468915	HN010	China	II	Non-determinate	Purple	Tawny	Black	Black
PI 507354	HN011	Japan	I	Determinate	Purple	Gray	Yellow	Yellow
PI 567305	HN012	China	IV	Non-determinate	Purple	Tawny	Black	Black
S05-11482	HN013	U.S.	IV,V	Determinate	White	Tawny	N/A	Black
PI 548667 (Essex)	HN014	U.S.	V	Determinate	Purple	Gray	Yellow	Imperfect black
PI 437654	HN015	China	III	Non-determinate	Purple	Tawny	Black	Black
PI 567387	HN016	China	IV	Non-determinate	Purple	Light tawny	Greenish-brown	Brown
PI 437725	HN017	China	IV	Semi-determinate	Purple	Tawny	Black	Black
PI 437690	HN018	China	III	Semi-determinate	White	Light tawny	Black	Black
PI 548402	HN019	China	IV	Determinate	White	Tawny	Black	Black
PI 088788	HN020	China	III	Non-determinate	White	Tawny	Black	Black
PI 209332	HN021	Japan	IV	Non-determinate	Purple	Tawny	Black	Black
PI 404198B	HN022	China	IV	Non-determinate	Purple	Tawny	Black	Black
PI 424608A	HN023	South Korea	IV	Non-determinate	Purple	Tawny	Black	Black
PI 548316	HN024	China	III	Non-determinate	White	Near gray	Black	Black
PI 567516C	HN025	China	IV	Non-determinate	Purple	Light tawny	Greenish-brown	Greenish-brown
PI 612611	HN026	North Korea	III	Non-determinate	Purple	Light tawny	Brown	Reddish-brown
S10-11227	HN027	U.S.	N/A	Non-determinate	White	Tawny	Yellow	Brown
Holladay (S-100)	HN028	U.S.	V	Determinate	Purple	Gray	Yellow	Imperfect black
IA3023	HN029	U.S.	III	N/A	White	Light tawny	Yellow	Black
Maverick	HN030	U.S.	III	Non-determinate	Purple	Gray	Yellow	Buff
PI 079691-4	HN031	China	III	Non-determinate	Purple	Light tawny	Brown	Black
PI 086006	HN032	Japan	III	Non-determinate	Purple	Tawny	Reddish-brown	Reddish-brown
PI 087617	HN033	North Korea	III	Non-determinate	Purple	Tawny	Yellow	Tan
PI 087631-1	HN034	Japan	III	Non-determinate	Purple	Tawny	Light-green	Black

PI 196175	HN035	South Korea	V	Determinate	Purple	Tawny	Yellow	Brown w/ black
PI 200508	HN037	Japan	I	Determinate	Purple	Gray	Yellow	Buff
PI 248515	HN038	Japan	IV	Determinate	White	Gray	Yellow	Yellow
PI 366121	HN039	Japan	IV	N/A	Purple	Tawny	Black	Black
PI 378702	HN040	Japan	IV	N/A	Purple	Tawny	Black	Black
PI 398593	HN041	South Korea	V	Determinate	Purple	Tawny	Yellow	Brown
PI 398595	HN042	South Korea	V	Determinate	Purple	Tawny	Yellow	Brown
PI 398610	HN043	South Korea	V	Semi-determinate	Purple	Gray	Yellow	Buff
PI 398614	HN044	South Korea	V	Semi-determinate	Purple	Gray	Yellow	Buff
PI 407162 (soja)	HN045	South Korea	IV	N/A	Purple	Tawny	Black	Black
PI 407184	HN046	South Korea	IV	N/A	Purple	Tawny	Black	Black
PI 407729	HN047	China	IV	Non-determinate	White	Light tawny	Black	Black
PI 407965	HN048	South Korea	V	Non-determinate	Purple	Gray	Yellow	Buff
PI 408105A	HN049	South Korea	IV	Determinate	Purple	Tawny	Reddish-brown	Reddish-brown
PI 416937	HN050	Japan	VI	Determinate	Purple	Gray	Yellow	Yellow
PI 424078	HN051	South Korea	III	Non-determinate	Purple	Tawny	Black	Black
PI 424079	HN052	South Korea	IV	N/A	Purple	Tawny	Black	Black
PI 424088	HN053	South Korea	IV	N/A	Purple	Tawny	Black	Black
PI 437169B	HN054	Russian Federation	II	Non-determinate	Purple	Gray	Yellow	Imperfect black
PI 437679	HN055	China	IV	Semi-determinate	Purple	Tawny	Black	Black
PI 437863A	HN056	China	II	Non-determinate	White	Gray	Yellow	Light buff
PI 438258	HN057	China	II	Non-determinate	Purple	Tawny	Yellow	Brown
PI 458515	HN058	China	IV	Semi-determinate	White	Tawny	Black	Black
PI 464920B	HN059	China	III	Determinate	White	Gray	Yellow	Light buff
PI 467312	HN060	China	II	Non-determinate	Purple	Tawny	Greenish-brown	Brown
PI 471938	HN061	Nepal	V	Non-determinate	Purple	Gray	Yellow	Yellow
PI 475783B	HN062	China	II	Semi-determinate	Purple	Gray	Yellow	Yellow
PI 483463 (soja)	HN063	China	II	Non-determinate	Purple	Tawny	Black	Black
PI 518751	HN064	Former Serbia	II	Non-determinate	Purple	Gray	Yellow	Imperfect black
PI 542044	HN065	U.S.	III	Non-determinate	White	Tawny	Yellow	Black
PI 547862	HN066	U.S.	III	Non-determinate	White	Tawny	Yellow	Black
PI 548317	HN067	China	III	Non-determinate	Purple	Gray	Green	Buff
PI 548349	HN068	North Korea	III	Non-determinate	Purple	Light tawny	Brown	Brown
PI 548415	HN069	China	IV	Non-determinate	Purple	Near gray	Black	Black
PI 548511	HN070	U.S.	II	Non-determinate	Purple	Gray	Yellow	Imperfect black
PI 548657 (Jackson)	HN071	U.S.	VII	Determinate	White	Gray	Yellow	Buff
PI 549031	HN072	China	III	Non-determinate	Purple	Light tawny	Black	Black
PI 552538 (Dunbar)	HN073	U.S.	III	Non-determinate	Purple	Gray	Yellow	Imperfect black

PI 561271	HN074	China	V	Non-determinate	White	Gray	Green	Buff
PI 567230	HN075	China	V	Non-determinate	Purple	Light tawny	Black	Black
PI 567336B	HN076	China	IV	Non-determinate	Purple	Tawny	Black	Black
PI 567343	HN077	China	V	Non-determinate	Purple	Light tawny	Brown	Brown
PI 567354	HN078	China	IV	Non-determinate	White	Tawny	Brown	Brown
PI 567357	HN079	China	III	Non-determinate	White	Gray	Yellow	Buff
PI 567383	HN080	China	V	Semi-determinate	White	Gray	Yellow	Buff
PI 567519	HN081	China	III	Non-determinate	White	Gray	Yellow	Dark buff
PI 567611	HN082	China	IV	Non-determinate	White	Gray	Yellow	Buff
PI 567651	HN083	China	IV	Semi-determinate	White	Gray	Yellow	Buff
PI 567690	HN084	China	III	Semi-determinate	Purple	Gray	Yellow	Buff
PI 567719	HN085	China	IV	Non-determinate	White	Gray	Yellow	Buff
PI 567731	HN086	China	III	Semi-determinate	Dark purple	Gray	Yellow	Buff
PI 591539	HN087	U.S.	III	Non-determinate	White	Tawny	Yellow	Black
PI 593258 (Macon)	HN088	N/A	III	Non-determinate	White	Tawny	Yellow	Black
PI 594012	HN089	South Korea	IV, V	Determinate	White	Tawny	Black	Black
PI 594512A	HN090	China	VII	Determinate	Purple	Tawny	Reddish-brown	Reddish-brown
PI 594599	HN091	China	IV	Semi-determinate	Purple	Tawny	Black	Black
PI 597387 (Pana)	HN092	U.S.	III	Non-determinate	Purple	Gray	Yellow	Buff
PI 603154	HN093	North Korea	V	Determinate	White	Gray	Yellow	Buff
PI 603170	HN094	North Korea	IV	Determinate	Purple	Tawny	Reddish-brown	Reddish-brown
PI 603175	HN095	North Korea	IV	Determinate	Purple	Tawny	Reddish-brown	Reddish-brown
PI 603176A	HN096	North Korea	IV	Non-determinate	Purple	Tawny	Black	Black
PI 603497	HN097	China	III	Determinate	Purple	Tawny	Black	Black
PI 605869A	HN098	Vietnam	V	Non-determinate	Purple	Tawny	Green	Black
PI 639740 (LD00-3309)	HN099	U.S.	IV	Non-determinate	Purple	Tawny	Yellow	Black
PI 647086	HN100	U.S.	VIII	Determinate	Purple	Gray	Yellow	Imperfect black
PI 658519 (LD00-2817)	HN101	N/A	VII	Non-determinate	Purple	Tawny	Reddish-brown	Reddish-brown
S07-5049	HN102	U.S.	IV	Non-determinate	Purple	Light tawny	Yellow	Buff
V71-370	HN103	U.S.	VI	N/A	N/A	N/A	Yellow	N/A
FC 31721	HN104	N/A	VI	Non-determinate	Purple	Tawny	Brown	Brown
PI 438471 (Fiskeby III)	HN105	Sweden	0	Determinate	Purple	Tawny	Yellow	Brown
PI 417091	HN106	Japan	II	Non-determinate	Purple	Tawny	Black	Black
PI 417015	HN107	Japan	III	Determinate	Light purple	Gray	Yellow	Buff

Color coded Text

**Red**	**Glycine soja**	**7**
**Blue**	**Landrace**	**43**
**Green**	**Elite lines/Breeding parents**	**56**
**Flowering type**		
Determinate	**60**	
Indeterminate	**28**	
Semi-determinate	**12**	
**Country of origin:**		
China	46	
South Korea	16	
North Korea	7	
Japan	11	
USA	17	
Vietnam	1	(These can be combined as Others- 5)
Sweden	1	"
Russia +Serbia	2	"
Nepal	1	"

Supplementary Table 2. Summary of soybean accession sequence coverage and mean depth of resequencing.

Genotypes	ID	Mean Depth	Coverage(%)	Total Reads	Total Bases
PI_518664	HN001	14.8	97.24	169012338	15211110420
Peking	HN002	15.68	97.07	182862604	16457634360
PI_089772	HN003	16.3	96.79	190153268	17113794120
PI_090763	HN004	14.28	96.57	167923632	15113126880
PI_404166	HN005	16.58	97.31	193155444	17383989960
PI_407788A	HN006	15.5	97.19	178857216	16097149440
PI_424298	HN007	16.18	97.27	190922894	17183060460
PI_437655	HN008	13.37	96.86	162161226	14594510340
PI_495017C	HN009	15.6	97.38	182789762	16451078580
PI_468915	HN010	12.51	95.55	144058760	12965288400
PI_507354	HN011	13.29	97.24	153640902	13827681180
PI_567305	HN012	13.39	95.91	155405290	13986476100
S05-11482	HN013	15.76	97.66	185390372	16685133480
PI_548667	HN014	13.64	97.21	161696054	14552644860
PI_437654	HN015	15.77	96.66	186339704	16770573360
PI_567387	HN016	20.48	97.26	243020574	21871851660
PI_437725	HN017	19.8	96.41	235192378	21167314020
PI_437690	HN018	14.15	96.87	167942518	15114826620
PI_548402	HN019	18.37	97.43	215669112	19410220080
PI_088788	HN020	12	96.35	141883428	12769508520
PI_209332	HN021	15.82	97.07	182081550	16387339500
PI_404198B	HN022	14.84	96.64	171861964	15467576760
PI_424608A	HN023	12.58	96.99	145279868	13075188120
PI_548316	HN024	17.68	97.28	203063302	18275697180
PI_567516C	HN025	13.22	95.92	157577702	14181993180
PI_612611	HN026	14.9	97.08	177478250	15973042500
S10-11227	HN027	17.17	97.51	195287660	17575889400
Holladay	HN028	17.6	97.74	203654054	18328864860
IA3023	HN029	16.27	98.5	186734870	16806138300
Maverick	HN030	16.34	98.16	186590730	16793165700
PI_079691-4	HN031	15.08	96.82	178065582	16025902380
PI_086006	HN032	19.54	97.55	234928518	21143566620
PI_087617	HN033	19.64	97.92	227710262	20493923580
PI_087631-1	HN034	16.36	97.31	194753982	17527858380
PI_196175	HN035	18.59	97.59	215692734	19412346060
PI_200508	HN037	14.84	97.55	179642206	16167798540
PI_248515	HN038	12.67	97.32	155145388	13963084920
PI_366121	HN039	16.97	97.61	213554280	19219885200
PI_378702	HN040	14.29	96.46	179746598	16177193820
PI_398593	HN041	18.63	97.7	213815782	19243420380

PI_398595	HN042	19.64	97.7	229236844	20631315960
PI_398610	HN043	19.95	97.68	232987084	20968837560
PI_398614	HN044	16.93	97.56	196015226	17641370340
PI_407162	HN045	19.51	97.02	228079008	20527110720
PI_407184	HN046	18.81	96.38	224516646	20206498140
PI_407729	HN047	17.84	97.23	207398246	18665842140
PI_407965	HN048	20.48	97.66	239711488	21574033920
PI_408105A	HN049	17.38	97.81	200239264	18021533760
PI_416937	HN050	19.48	97.73	223204656	20088419040
PI_424078	HN051	18.29	97.11	216599770	19493979300
PI_424079	HN052	18.4	96.78	218858586	19697272740
PI_424088	HN053	17.93	96.7	212540282	19128625380
PI_437169B	HN054	19.19	97.85	219653914	19768852260
PI_437679	HN055	17.68	97.32	206104066	18549365940
PI_437863A	HN056	19.3	97.93	224912934	20242164060
PI_438258	HN057	17.98	98.22	206996246	18629662140
PI_458515	HN058	14.3	98.41	172032796	15482951640
PI_464920B	HN059	20.7	98.03	244836236	22035261240
PI_467312	HN060	17.78	97.5	213275758	19194818220
PI_471938	HN061	16.82	97.72	199250662	17932559580
PI_475783B	HN062	15.31	98.01	178533828	16068044520
PI_483463	HN063	14.96	95.76	183277998	16495019820
PI_518751	HN064	14.69	97.22	171088604	15397974360
PI_542044	HN065	22.72	99.3	253465324	22811879160
PI_547862	HN066	20.43	99.22	232670066	20940305940
PI_548317	HN067	18.84	97.63	218281110	19645299900
PI_548349	HN068	20.39	97.75	240767152	21669043680
PI_548415	HN069	16.23	97.24	191324332	17219189880
PI_548511	HN070	16.39	97.98	194321730	17488955700
PI_548657	HN071	16.94	97.76	199866892	17988020280
PI_549031	HN072	15.95	97.3	189097166	17018744940
PI_552538	HN073	17.52	98.2	204243824	18381944160
PI_561271	HN074	17.65	97.73	210015330	18901379700
PI_567230	HN075	17.4	97.11	205061116	18455500440
PI_567336B	HN076	17.94	97.18	209908592	18891773280
PI_567343	HN077	14.6	96.96	169266356	15233972040
PI_567354	HN078	17.61	97.56	202046178	18184156020
PI_567357	HN079	15.85	97.34	181795242	16361571780
PI_567383	HN080	18.44	97.66	216112624	19450136160
PI_567519	HN081	17.33	98.29	198156232	17834060880
PI_567611	HN082	18.14	97.64	212037010	19083330900
PI_567651	HN083	15.03	97.37	176228940	15860604600
PI_567690	HN084	17.16	97.61	200696298	18062666820
PI_567719	HN085	15.11	97.76	205652200	18508698000
PI_567731	HN086	15.6	97.44	182659124	16439321160
PI_591539	HN087	19.09	99.28	214446256	19300163040

PI_593258	HN088	17.8	98.42	203637960	18327416400
PI_594012	HN089	18.38	97.76	214102900	19269261000
PI_594512A	HN090	20.49	97.79	235461772	21191559480
PI_594599	HN091	14.87	97.81	182991332	16469219880
PI_597387	HN092	17.35	98.37	210656058	18959045220
PI_603154	HN093	17.62	97.92	202119518	18190756620
PI_603170	HN094	17.44	97.72	203885070	18349656300
PI_603175	HN095	24.6	97.76	285733800	25716042000
PI_603176A	HN096	18.92	97.89	218750198	19687517820
PI_603497	HN097	15.56	97.57	193730402	17435736180
PI_605869A	HN098	15.96	97.79	196814124	17713271160
PI_639740	HN099	13.69	98.31	168515406	15166386540
PI_647086	HN100	18.2	98.07	208002580	18720232200
PI_658519	HN101	18.71	98.31	214508272	19305744480
S07-5049	HN102	20.88	98.61	238623482	21476113380
V71-370	HN103	19.09	97.8	220099746	19808977140
FC_31721	HN104	20.2	97.07	234212784	21079150560
PI_438471	HN105	24.01	97.83	279508308	25155747720
PI_417091	HN106	22.45	97.38	263451824	23710664160
PI_417015	HN107	16.22	96.68	188059874	16925388660
Total		17.16	97.48	21331077404	1.92E+12

Supplementary Table 3. Distribution of SNPs in different chromosomes of 106 soybean accessions.

	Total	Intergenic	Intron	5'-UTR	3'-UTR	Exon		
						Total	Nonsynonymous*	Synonymous
**Gm01**	543355	473103	43488	4078	6798	15888	9496	6405
**Gm02**	478949	390621	54810	5832	8770	18916	11098	7830
**Gm03**	578158	483219	60010	4998	8583	21348	12852	8499
**Gm04**	516441	436810	51665	4981	7390	15595	9190	6416
**Gm05**	376452	304637	44664	4807	7483	14861	8610	6264
**Gm06**	570418	464902	65534	6495	9853	23634	14259	9394
**Gm07**	462355	365293	60758	5915	9060	21329	12667	8678
**Gm08**	471167	356903	72178	7084	11048	23954	13792	10179
**Gm09**	485670	398083	55900	5632	7560	18495	10932	7582
**Gm10**	524201	435637	56166	5289	8227	18882	10984	7909
**Gm11**	388464	316025	45194	4658	7143	15444	8978	6477
**Gm12**	414629	343005	43916	4754	7102	15852	9296	6562
**Gm13**	511424	395165	71999	7595	11499	25166	14719	10455
**Gm14**	554802	471356	52577	4704	7554	18611	11382	7239
**Gm15**	656251	556855	62862	5627	8722	22185	13763	8429
**Gm16**	499810	397009	61411	5926	8919	26545	16396	10162
**Gm17**	447863	370578	47419	5104	8191	16571	9533	7047
**Gm18**	824220	696508	78750	7557	11287	30118	18357	11780
**Gm19**	528172	447899	51137	4823	7410	16903	10060	6848
**Gm20**	492594	411288	51407	5004	7653	17242	10359	6889
**GmU**	91890	88416	1749	160	339	1226	837	388
**Total**	10417285	8603312	1133594	111023	170591	398765	237560	161432

*SNPs located in overlap regions of different transcripts were annotated independently. Some SNPs are synonymous SNPs in one transcript, and at the same time non-synonymous SNPs in another overlapping transcript and vice versa. Thus the sum of synonymous and non-synonymous SNPs is more than the number of SNPs in the CDS regions

Supplementary Table 4. Distribution of indels within various soybean genomic regions

Chromosome	Indel	Intergenic	Intron	5'-UTR	3'-UTR	Exon
**Gm01**	34228	28276	4251	572	760	369
**Gm02**	37312	29072	5753	861	1080	546
**Gm03**	40449	32243	5949	718	1006	533
**Gm04**	35377	28392	4896	730	903	456
**Gm05**	27109	20944	4280	636	873	376
**Gm06**	41817	32895	6358	810	1150	604
**Gm07**	35429	27220	5801	793	1053	562
**Gm08**	39755	29356	7476	975	1323	625
**Gm09**	36419	28252	5902	812	975	478
**Gm10**	37908	29949	5795	730	906	528
**Gm11**	28171	21798	4433	652	830	458
**Gm12**	27958	21913	4179	590	872	404
**Gm13**	43732	33342	7307	1041	1362	680
**Gm14**	34416	27758	4759	637	823	439
**Gm15**	44067	35834	5969	738	923	603
**Gm16**	37233	28943	5958	786	974	572
**Gm17**	34747	27591	4916	751	1011	478
**Gm18**	57177	46373	7907	1000	1219	678
**Gm19**	35836	28914	4902	658	889	473
**Gm20**	32670	25824	4831	665	932	418
**GmU**	4004	3761	159	14	38	32
**Total**	745814	588650	111781	15169	19902	10312

Supplementary Table 5 a. Distribution of large effect SNPs in three soybean gene pools.

Group	Premature stop	Stop to non Stop code	Start to non start code	Splice sites	Total
Wild	5271	963	886	3100	10220
Landrace	3897	757	622	2460	7736
Elite	4104	733	698	2612	8147
Cultivated	5254	928	845	3210	10237
Total	6236	1072	1000	3721	12029

Cultivated = Landrace + Elite

Supplementary Table 5 b. GO enrichment cluster of genes with large effect SNPs[1,2].

GO-ID	Description	cluster freq	total freq	P-value	Genes in the test set
GO:032559	adenyl ribonucleotide binding	342/1695	3915/27682	1.39E-12	Glyma13G41790,Glyma04G04510,Glyma04G09210,Glyma20G26160,Glyma14G36660,Glyma13G36600,Glyma18G00700,Glyma19G39680,Glyma12G06530,Glyma07G08120,Glyma01G03410,Glyma09G30430,Glyma12G34330,Glyma10G30060,Glyma02G03070,Glyma13G26000,Glyma11G15520,Glyma18G50510,Glyma13G18690,Glyma16G08560,Glyma12G08900,Glyma16G25010,Glyma0028S00210,Glyma17G34190,Glyma13G05430,Glyma18G01960,Glyma06G40690,Glyma09G03190,Glyma17G08540,Glyma17G01730,Glyma20G16170,Glyma11G20180,Glyma13G25420,Glyma07G07550,Glyma19G31720,Glyma13G23840,Glyma06G39990,Glyma13G17440,Glyma04G04380,Glyma05G34180,Glyma02G45800,Glyma08G03240,Glyma16G34030,Glyma17G10420,Glyma14G33400,Glyma02G11110,Glyma06G17560,Glyma18G07080,Glyma13G21820,Glyma07G32200,Glyma13G35790,Glyma19G39580,Glyma06G41880,Glyma06G45590,Glyma19G41210,Glyma06G15900,Glyma18G48600,Glyma07G07110,Glyma05G33240,Glyma04G05920,Glyma13G21030,Glyma01G07970,Glyma06G21140,Glyma07G01890,Glyma02G47230,Glyma18G09670,Glyma16G24940,Glyma16G04910,Glyma15G36110,Glyma20G23010,Glyma18G11590,Glyma15G10370,Glyma04G41060,Glyma08G09050,Glyma08G46130,Glyma05G32070,Glyma09G24410,Glyma18G50450,Glyma06G41430,Glyma14G02740,Glyma08G41510,Glyma18G40680,Glyma10G07500,Glyma05G27850,Glyma05G30650,Glyma11G34210,Glyma17G34410,Glyma15G20000,Glyma04G01440,Glyma15G37390,Glyma11G19120,Glyma16G21050,Glyma06G08480,Glyma09G33120,Glyma18G09290,Glyma09G19730,Glyma14G04930,Glyma11G37910,Glyma20G37930,Glyma11G09060,Glyma18G16060,Glyma13G16540,Glyma09G34860,Glyma20G27860,Glyma18G15150,Glyma08G39070,Glyma08G12540,Glyma12G32880,Glyma03G05640,Glyma01G10100,Glyma14G06570,Glyma03G02310,Glyma15G02520,Glyma01G27440,Glyma11G04310,Glyma18G06990,Glyma03G30310,Glyma15G37320,Glyma15G13290,Glyma16G32640,Glyma12G17340,Glyma13G40550,Glyma13G06620,Glyma17G38210,Glyma15G34810,Glyma07G09260,Glyma09G08370,Glyma13G25970,Glyma06G21910,Glyma07G11880,Glyma08G13040,Glyma16G32700,Glyma13G38950,Glyma08G13150,Glyma18G45440,Glyma17G33470,Glyma06G47650,Glyma07G00670,Glyma20G38380,Glyma06G20360,Glyma20G34060,Glyma20G38610,Glyma16G02290,Glyma14G12710,Glyma01G42240,Glyma16G22370,Glyma02G09080,Glyma05G30800,Glyma16G27250,Glyma08G40030,Glyma18G52480,Glyma17G02080,Glyma03G33590,Glyma16G33590,Glyma03G36310,Glyma14G07750,Glyma02G40850,Glyma16G02340,Glyma07G00340,Glyma10G38450.2,Glyma11G09070,Glyma04G43190,Glyma19G31950,Glyma18G44800,Glyma11G02150,Glyma18G50540,Glyma01G42770,Glyma13G43480,Glyma05G21420,Glyma17G09700,Glyma16G29040,Glyma18G09980,Glyma02G06700,Glyma10G41990,Glyma12G16450,Glyma03G32530,Glyma14G00380,Glyma20G06780,Glyma01G41150,Glyma04G36240,Glyma14G10790,Glyma20G29060,Glyma09G35140,Glyma16G32830,Glyma07G16260,Glyma08G44620,Glyma08G40920,Glyma06G20200,Glyma20G27790,Glyma09G05550,Glyma09G28260,Glyma09G02930,Glyma20G27800,Glyma19G38970,Glyma17G34060,Glyma15G37290,Glyma09G37580,Glyma20G35970,Glyma12G03140,Glyma03G04780,Glyma01G39380,Glyma10G38810,Glyma18G45200,Glyma18G53590,Glyma17G16070,Glyma02G46070,Glyma01G00960,Glyma07G30260,Glyma09G40150,Glyma08G06730,Glyma18G08220,Glyma02G46670,Glyma14G05060,Glyma02G03760,Glyma08G06520,Glyma16G03780,Glyma09G38730,Glyma03G29230,Glyma16G07060,Glyma07G39300,Glyma05G02470,Glyma18G38470,Glyma06G40780,Glyma10G32800,Glyma04G27730,Glyma06G47870,Glyma11G19880,Glyma18G47590,Glyma07G04560,Glyma06G40110,Glyma06G08880,Glyma19G44690,Glyma13G02620,Glyma04G06610,Glyma18G50650,Glyma09G30070,Glyma10G08040,Glyma20G30100,Glyma18G49060,Glyma08G18760,Glyma14G03040,Glyma16G33950,Glyma01G34780,Glyma18G47140,Glyma06G18700,Glyma20G27720,Glyma13G42950,Glyma18G50440,Glyma06G40710,Glyma06G40920,Glyma05G23260,Glyma18G46930,Glyma13G10530,Glyma05G01460,Glyma14G36960,Glyma18G51960,Glyma13G10040,Glyma17G04470,Glyma15G17430,Glyma09G13540,Glyma06G40740,Glyma15G42030,Glyma06G02010,Glyma09G26710,Glyma09G05710,Glyma16G10080,Glyma07G31140,Glyma15G00670,Glyma17G09120,Glyma03G00540,Glyma18G04440,Glyma06G40980,Glyma03G05400,Glyma18G08870,Glyma07G12130,Glyma02G48100,Glyma12G34890,Glyma15G00340,Glyma10G32990,Glyma12G33340,Glyma06G40350,Glyma15G00950,Glyma18G10610,Glyma07G10460,Glyma14G29130,Glyma08G15360,Glyma15G37140,Glyma11G27200,Glyma13G10200,Glyma19G35270,Glyma11G05140,Glyma06G40670,Glyma04G01110,Glyma09G40650,Glyma18G51950,Glyma05G03440,Glyma15G04850,Glyma16G32710,Glyma16G10760,Glyma19G00650,Glyma18G42700,Glyma07G09530,Glyma04G34140,Glyma10G35610,Glym

					a10G02020,Glyma06G05910,Glyma03G32160,Glyma13G23610,Glyma13G26250,Glyma05G30030,Glyma13G00370,Glyma19G43290,Glyma03G37760,Glyma20G27660,Glyma17G30980,Glyma08G13060,Glyma17G34170,Glyma12G17450,Glyma12G17360,Glyma19G34250,Glyma04G39670,Glyma03G42000,Glyma10G04480,Glyma13G06490,Glyma11G34490,Glyma02G14310,Glyma20G27510,Glyma05G30260,Glyma10G35310,Glyma08G23150,Glyma09G37650,Glyma07G02940,Glyma14G38650,Glyma18G10470,Glyma12G36440,Glyma15G04280,Glyma08G08000,Glyma15G04350,Glyma19G04140,Glyma09G08180,Glyma08G05720,Glyma08G43170
GO:005524	ATP binding	341/1695	3913/27682	2.08E-12	Glyma13G41790,Glyma04G04510,Glyma04G09210,Glyma20G26160,Glyma14G36660,Glyma13G36600,Glyma18G00700,Glyma19G39680,Glyma12G06530,Glyma07G08120,Glyma01G03410,Glyma09G30430,Glyma12G34330,Glyma10G30060,Glyma02G03070,Glyma13G26000,Glyma11G15520,Glyma18G50510,Glyma13G18690,Glyma16G08560,Glyma12G08900,Glyma16G25010,Glyma0028S00210,Glyma17G34190,Glyma13G05430,Glyma18G01960,Glyma06G40690,Glyma09G03190,Glyma17G08540,Glyma17G01730,Glyma20G16170,Glyma11G20180,Glyma13G25420,Glyma07G07550,Glyma19G31720,Glyma13G23840,Glyma06G39990,Glyma13G17440,Glyma04G04380,Glyma05G34180,Glyma02G45800,Glyma08G03240,Glyma16G34030,Glyma17G10420,Glyma14G33400,Glyma02G11110,Glyma06G17560,Glyma18G07080,Glyma13G21820,Glyma07G32200,Glyma13G35790,Glyma19G39580,Glyma06G41880,Glyma06G45590,Glyma19G41210,Glyma06G15900,Glyma18G48600,Glyma07G07110,Glyma05G33240,Glyma04G05920,Glyma13G21030,Glyma01G07970,Glyma06G21140,Glyma07G01890,Glyma02G47230,Glyma18G09670,Glyma16G24940,Glyma15G36110,Glyma20G23010,Glyma18G11590,Glyma15G10370,Glyma04G41060,Glyma08G09050,Glyma08G46130,Glyma05G32070,Glyma09G24410,Glyma18G50450,Glyma06G41430,Glyma14G02740,Glyma08G41510,Glyma18G40680,Glyma10G07500,Glyma05G27850,Glyma05G30650,Glyma11G34210,Glyma17G34410,Glyma15G20000,Glyma04G01440,Glyma15G37390,Glyma11G19120,Glyma16G21050,Glyma06G08480,Glyma09G33120,Glyma18G09290,Glyma09G19730,Glyma14G04930,Glyma11G37910,Glyma20G37930,Glyma11G09060,Glyma18G16060,Glyma13G16540,Glyma09G34860,Glyma20G27860,Glyma18G15150,Glyma08G39070,Glyma08G12540,Glyma12G32880,Glyma03G05640,Glyma01G10100,Glyma14G06570,Glyma03G02310,Glyma15G02520,Glyma01G27440,Glyma11G04310,Glyma18G06990,Glyma03G30310,Glyma15G37320,Glyma15G13290,Glyma16G32640,Glyma12G17340,Glyma13G40550,Glyma13G06620,Glyma17G38210,Glyma15G34810,Glyma07G09260,Glyma09G08370,Glyma13G25970,Glyma06G21910,Glyma07G11880,Glyma08G13040,Glyma16G32700,Glyma13G38950,Glyma08G13150,Glyma18G45440,Glyma17G33470,Glyma06G47650,Glyma07G00670,Glyma20G38380,Glyma06G20360,Glyma20G34060,Glyma20G38610,Glyma16G02290,Glyma14G12710,Glyma01G42240,Glyma16G22370,Glyma02G09080,Glyma05G30800,Glyma16G27250,Glyma08G40030,Glyma18G52480,Glyma17G02080,Glyma03G33590,Glyma16G33590,Glyma03G36310,Glyma14G07750,Glyma02G40850,Glyma16G02340,Glyma07G00340,Glyma10G38450.2,Glyma11G09070,Glyma04G43190,Glyma19G31950,Glyma18G44800,Glyma11G02150,Glyma18G50540,Glyma01G42770,Glyma13G43480,Glyma05G21420,Glyma17G09700,Glyma16G29040,Glyma18G09980,Glyma02G06700,Glyma10G41990,Glyma12G16450,Glyma03G32530,Glyma14G00380,Glyma20G06780,Glyma01G41150,Glyma04G36240,Glyma14G10790,Glyma20G29060,Glyma09G35140,Glyma16G32830,Glyma07G16260,Glyma08G44620,Glyma08G40920,Glyma06G20200,Glyma20G27790,Glyma09G05550,Glyma09G28260,Glyma09G02930,Glyma20G27800,Glyma19G38970,Glyma17G34060,Glyma15G37290,Glyma09G37580,Glyma20G35970,Glyma12G03140,Glyma03G04780,Glyma01G39380,Glyma10G38810,Glyma18G45200,Glyma18G53590,Glyma17G16070,Glyma02G46070,Glyma01G00960,Glyma07G30260,Glyma09G40150,Glyma08G06730,Glyma18G08220,Glyma02G46670,Glyma14G05060,Glyma02G03760,Glyma08G06520,Glyma16G03780,Glyma09G38730,Glyma03G29230,Glyma16G07060,Glyma07G39300,Glyma05G02470,Glyma18G38470,Glyma06G40780,Glyma10G32800,Glyma04G27730,Glyma06G47870,Glyma11G19880,Glyma18G47590,Glyma07G04560,Glyma06G40110,Glyma06G08880,Glyma19G44690,Glyma13G02620,Glyma04G06610,Glyma18G50650,Glyma09G30070,Glyma10G08040,Glyma20G30100,Glyma18G49060,Glyma08G18760,Glyma14G03040,Glyma16G33950,Glyma01G34780,Glyma18G47140,Glyma06G18700,Glyma20G27720,Glyma13G42950,Glyma18G50440,Glyma06G40710,Glyma06G40920,Glyma05G23260,Glyma18G46930,Glyma13G10530,Glyma05G01460,Glyma14G36960,Glyma18G51960,Glyma13G10040,Glyma17G04470,Glyma15G17430,Glyma09G13540,Glyma06G40740,Glyma15G42030,Glyma06G02010,Glyma09G26710,Glyma09G05710,Glyma16G10080,Glyma07G31140,Glyma15G00670,Glyma17G09120,Glyma03G00540,Glyma18G04440,Glyma06G40980,Glyma03G05400,Glyma18G08870,Glyma07G12130,Glyma02G48100,Glyma12G34890,Glyma15G00340,Glyma10G32990,Glyma12G33340,Glyma06G40350,Glyma15G00950,Glyma18G10610,Glyma07G10460,Glyma14G29130,Glyma08G15360,Glyma15G37140,Glyma11G27200,Glyma13G10200,Glyma19G35270,Glyma11G05140,Glyma06G40670,Glyma04G01110,Glyma09G40650,Glyma18G51950,Glyma05G03440,Glyma15G04850,Glyma16G32710,Glyma16G10760,Glyma19G00650,Glyma18G42700,Glyma07G09530,Glyma04G34140,Glyma10G35610,Glyma10G02020,Glyma06G05910,Glyma03G32160,Glyma13G23610,Glyma13G26250,Glyma05G30030,Glyma13G00370,Glyma19G43290,Glyma03G37760,Glyma20G27660,Glyma17G30980,Glyma08G13060,Glyma17G34170,Glyma12G17450,Glyma12G17360,Glyma19G34250,Glyma04G39670,Glyma03G42000,Glyma10G04480,Glyma13G06490,Glyma11G34490,Glyma02G14310,Glyma20G27510,Glyma05G30260,Glyma10G35310,Glyma08G23150,Glyma09G37650,Glyma07G02940,Glyma14G38650,Glyma18G10470,Glyma12G36440,Glyma15G04280,Glyma08G08000,Glyma15G04350,Glyma19G04140,Glyma09G08180,Glyma08G05720,Glyma08G43170

GO:001882	nucleoside binding	356/1695	4166/27682	7.70E-12	Glyma13G41790,Glyma04G04510,Glyma04G09210,Glyma20G26160,Glyma14G36660,Glyma13G36600,Glyma18G00700,Glyma17G08610,Glyma19G39680,Glyma12G06530,Glyma07G08120,Glyma01G03410,Glyma09G30430,Glyma12G34330,Glyma10G30060,Glyma02G03070,Glyma13G26000,Glyma15G14020,Glyma11G15520,Glyma18G50510,Glyma13G18690,Glyma16G08560,Glyma12G08900,Glyma16G25010,Glyma0028S00210,Glyma17G34190,Glyma13G05430,Glyma18G01960,Glyma06G40690,Glyma09G03190,Glyma17G08540,Glyma17G01730,Glyma20G16170,Glyma11G20180,Glyma13G25420,Glyma07G07550,Glyma19G31720,Glyma13G23840,Glyma06G39990,Glyma13G17440,Glyma04G04380,Glyma05G34180,Glyma02G45800,Glyma08G03240,Glyma16G34030,Glyma17G10420,Glyma14G33400,Glyma02G11110,Glyma06G17560,Glyma18G07080,Glyma08G08500,Glyma13G21820,Glyma07G32200,Glyma13G35790,Glyma19G39580,Glyma06G41880,Glyma06G45590,Glyma19G41210,Glyma06G15900,Glyma18G48600,Glyma07G07110,Glyma05G33240,Glyma04G05920,Glyma01G07970,Glyma13G21030,Glyma06G21140,Glyma07G01890,Glyma02G47230,Glyma18G09670,Glyma16G24940,Glyma16G04910,Glyma15G36110,Glyma20G23010,Glyma18G11590,Glyma15G10370,Glyma15G14080,Glyma04G41060,Glyma08G09050,Glyma08G46130,Glyma05G32070,Glyma09G24410,Glyma18G50450,Glyma14G02740,Glyma06G41430,Glyma08G41510,Glyma18G40680,Glyma10G07500,Glyma05G27850,Glyma15G23150,Glyma05G30650,Glyma11G34210,Glyma17G34410,Glyma15G20000,Glyma04G01440,Glyma15G37390,Glyma11G19120,Glyma16G21050,Glyma06G08480,Glyma09G33120,Glyma18G09290,Glyma09G19730,Glyma14G04930,Glyma11G37910,Glyma20G37930,Glyma11G09060,Glyma18G16060,Glyma13G16540,Glyma09G34860,Glyma20G27860,Glyma18G15150,Glyma08G39070,Glyma08G12540,Glyma12G32880,Glyma03G05640,Glyma01G10100,Glyma14G06570,Glyma03G02310,Glyma15G02520,Glyma01G27440,Glyma11G04310,Glyma18G06990,Glyma03G30310,Glyma15G37320,Glyma15G13290,Glyma16G32640,Glyma12G17340,Glyma13G40550,Glyma13G06620,Glyma17G38210,Glyma15G34810,Glyma06G21910,Glyma07G09260,Glyma09G08370,Glyma13G25970,Glyma07G11880,Glyma08G13040,Glyma16G32700,Glyma13G38950,Glyma08G13150,Glyma18G45440,Glyma17G33470,Glyma06G47650,Glyma07G00670,Glyma20G38380,Glyma06G20360,Glyma20G34060,Glyma20G38610,Glyma16G02290,Glyma14G12710,Glyma01G42240,Glyma16G22370,Glyma02G09080,Glyma05G30800,Glyma16G27250,Glyma08G40030,Glyma18G52480,Glyma17G02080,Glyma03G33590,Glyma16G33590,Glyma03G36310,Glyma14G07750,Glyma02G40850,Glyma16G02340,Glyma10G38450.2,Glyma07G00340,Glyma11G09070,Glyma04G43190,Glyma19G31950,Glyma18G44800,Glyma15G14200,Glyma11G02150,Glyma18G50540,Glyma01G42770,Glyma05G21420,Glyma13G43480,Glyma17G09700,Glyma16G29040,Glyma18G09980,Glyma01G43190,Glyma02G06700,Glyma10G41990,Glyma12G16450,Glyma03G32530,Glyma14G00380,Glyma20G06780,Glyma01G41150,Glyma04G36240,Glyma14G10790,Glyma19G34960,Glyma20G29060,Glyma09G35140,Glyma11G15530,Glyma16G32830,Glyma07G16260,Glyma08G44620,Glyma08G40920,Glyma06G20200,Glyma20G27790,Glyma09G05550,Glyma09G28260,Glyma09G02930,Glyma19G38970,Glyma20G27800,Glyma17G34060,Glyma15G37290,Glyma09G37580,Glyma20G35970,Glyma12G03140,Glyma03G04780,Glyma01G39380,Glyma10G38810,Glyma18G45200,Glyma18G53590,Glyma17G16070,Glyma02G46070,Glyma01G00960,Glyma07G30260,Glyma09G40150,Glyma08G06730,Glyma08G08550,Glyma18G08220,Glyma02G46670,Glyma14G05060,Glyma02G03760,Glyma08G06520,Glyma16G03780,Glyma09G38730,Glyma16G07060,Glyma03G29230,Glyma07G39300,Glyma05G02470,Glyma18G38470,Glyma06G40780,Glyma10G32800,Glyma04G27730,Glyma06G47870,Glyma11G19880,Glyma18G47590,Glyma07G04560,Glyma06G40110,Glyma06G08880,Glyma19G44690,Glyma13G02620,Glyma04G06610,Glyma18G50650,Glyma09G30070,Glyma10G08040,Glyma06G45340,Glyma20G30100,Glyma18G49060,Glyma08G18760,Glyma14G03040,Glyma16G33950,Glyma01G34780,Glyma18G47140,Glyma06G18700,Glyma20G27720,Glyma13G42950,Glyma18G50440,Glyma06G40710,Glyma06G40920,Glyma05G23260,Glyma18G46930,Glyma13G10530,Glyma05G01460,Glyma14G36960,Glyma18G51960,Glyma13G10040,Glyma17G04470,Glyma15G17430,Glyma09G13540,Glyma06G40740,Glyma15G42030,Glyma06G02010,Glyma09G26710,Glyma15G14030,Glyma09G05710,Glyma16G10080,Glyma07G31140,Glyma15G00670,Glyma17G09120,Glyma03G00540,Glyma18G04440,Glyma06G40980,Glyma03G05400,Glyma18G08870,Glyma07G12130,Glyma02G48100,Glyma12G34890,Glyma15G00340,Glyma10G32990,Glyma12G33340,Glyma06G40350,Glyma15G00950,Glyma18G10610,Glyma07G10460,Glyma14G29130,Glyma08G15360,Glyma15G37140,Glyma11G27200,Glyma13G10200,Glyma19G35270,Glyma11G05140,Glyma06G40670,Glyma04G01110,Glyma09G40650,Glyma18G51950,Glyma05G03440,Glyma15G04850,Glyma16G32710,Glyma16G10760,Glyma19G00650,Glyma18G42700,Glyma07G09530,Glyma04G34140,Glyma10G35610,Glyma10G02020,Glyma06G05910,Glyma03G32160,Glyma13G23610,Glyma13G26250,Glyma05G30030,Glyma13G00370,Glyma19G43290,Glyma03G37760,Glyma20G27660,Glyma17G30980,Glyma08G13060,Glyma17G34170,Glyma15G14210,Glyma12G17450,Glyma12G17360,Glyma19G34250,Glyma04G39670,Glyma03G42000,Glyma08G09540,Glyma10G04480,Glyma13G06490,Glyma11G34490,Glyma02G14310,Glyma20G27510,Glyma05G30260,Glyma10G35310,Glyma08G23150,Glyma09G37650,Glyma07G02940,Glyma14G38650,Glyma18G10470,Glyma12G36440,Glyma15G04280,Glyma08G08000,Glyma15G04350,Glyma19G04140,Glyma09G08180,Glyma08G05720,Glyma08G43170

GO:030554	adenyl nucleotide binding	355/1695	4155/27682	8.51E-12	Glyma13G41790,Glyma04G04510,Glyma04G09210,Glyma20G26160,Glyma14G36660,Glyma13G36600,Glyma18G00700,Glyma17G08610,Glyma19G39680,Glyma12G06530,Glyma07G08120,Glyma01G03410,Glyma09G30430,Glyma12G34330,Glyma10G30060,Glyma02G03070,Glyma13G26000,Glyma15G14020,Glyma11G15520,Glyma18G50510,Glyma13G18690,Glyma16G08560,Glyma12G08900,Glyma16G25010,Glyma0028S00210,Glyma17G34190,Glyma13G05430,Glyma18G01960,Glyma06G40690,Glyma09G03190,Glyma17G08540,Glyma17G01730,Glyma20G16170,Glyma11G20180,Glyma13G25420,Glyma07G07550,Glyma19G31720,Glyma13G23840,Glyma06G39990,Glyma13G17440,Glyma04G04380,Glyma05G34180,Glyma02G45800,Glyma08G03240,Glyma16G34030,Glyma17G10420,Glyma14G33400,Glyma02G11110,Glyma06G17560,Glyma18G07080,Glyma08G08500,Glyma13G21820,Glyma07G32200,Glyma13G35790,Glyma19G39580,Glyma06G41880,Glyma06G45590,Glyma19G41210,Glyma06G15900,Glyma18G48600,Glyma07G07110,Glyma05G33240,Glyma04G05920,Glyma13G21030,Glyma01G07970,Glyma06G21140,Glyma07G01890,Glyma02G47230,Glyma18G09670,Glyma16G24940,Glyma16G04910,Glyma15G36110,Glyma20G23010,Glyma18G11590,Glyma15G10370,Glyma15G14080,Glyma04G41060,Glyma08G09050,Glyma08G46130,Glyma05G32070,Glyma09G24410,Glyma18G50450,Glyma14G02740,Glyma06G41430,Glyma08G41510,Glyma18G40680,Glyma10G07500,Glyma05G27850,Glyma15G23150,Glyma05G30650,Glyma11G34210,Glyma17G34410,Glyma15G20000,Glyma04G01440,Glyma15G37390,Glyma11G19120,Glyma16G21050,Glyma06G08480,Glyma09G33120,Glyma18G09290,Glyma09G19730,Glyma14G04930,Glyma11G37910,Glyma20G37930,Glyma11G09060,Glyma18G16060,Glyma13G16540,Glyma09G34860,Glyma20G27860,Glyma18G15150,Glyma08G39070,Glyma08G12540,Glyma12G32880,Glyma03G05640,Glyma01G10100,Glyma14G06570,Glyma03G02310,Glyma15G02520,Glyma01G27440,Glyma11G04310,Glyma18G06990,Glyma03G30310,Glyma15G37320,Glyma15G13290,Glyma16G32640,Glyma12G17340,Glyma13G40550,Glyma13G06620,Glyma17G38210,Glyma15G34810,Glyma06G21910,Glyma07G09260,Glyma09G08370,Glyma13G25970,Glyma07G11880,Glyma08G13040,Glyma16G32700,Glyma13G38950,Glyma08G13150,Glyma18G45440,Glyma17G33470,Glyma06G47650,Glyma07G00670,Glyma20G38380,Glyma06G20360,Glyma20G34060,Glyma20G38610,Glyma16G02290,Glyma14G12710,Glyma01G42240,Glyma16G22370,Glyma02G09080,Glyma05G30800,Glyma16G27250,Glyma08G40030,Glyma18G52480,Glyma17G02080,Glyma03G33590,Glyma16G33590,Glyma03G36310,Glyma14G07750,Glyma02G40850,Glyma16G02340,Glyma10G38450.2,Glyma07G00340,Glyma11G09070,Glyma04G43190,Glyma19G31950,Glyma18G44800,Glyma15G14200,Glyma11G02150,Glyma18G50540,Glyma01G42770,Glyma05G21420,Glyma13G43480,Glyma17G09700,Glyma16G29040,Glyma18G09980,Glyma01G43190,Glyma02G06700,Glyma10G41990,Glyma12G16450,Glyma03G32530,Glyma14G00380,Glyma20G06780,Glyma01G41150,Glyma04G36240,Glyma14G10790,Glyma19G34960,Glyma20G29060,Glyma09G35140,Glyma16G32830,Glyma07G16260,Glyma08G44620,Glyma08G40920,Glyma06G20200,Glyma20G27790,Glyma09G05550,Glyma09G28260,Glyma09G02930,Glyma19G38970,Glyma20G27800,Glyma17G34060,Glyma15G37290,Glyma09G37580,Glyma20G35970,Glyma12G03140,Glyma03G04780,Glyma01G39380,Glyma10G38810,Glyma18G45200,Glyma18G53590,Glyma17G16070,Glyma02G46070,Glyma01G00960,Glyma07G30260,Glyma09G40150,Glyma08G06730,Glyma08G08550,Glyma18G08220,Glyma02G46670,Glyma14G05060,Glyma02G03760,Glyma08G06520,Glyma16G03780,Glyma09G38730,Glyma16G07060,Glyma03G29230,Glyma07G39300,Glyma05G02470,Glyma18G38470,Glyma06G40780,Glyma10G32800,Glyma04G27730,Glyma06G47870,Glyma11G19880,Glyma18G47590,Glyma07G04560,Glyma06G40110,Glyma06G08880,Glyma19G44690,Glyma13G02620,Glyma04G06610,Glyma18G50650,Glyma09G30070,Glyma10G08040,Glyma06G45340,Glyma20G30100,Glyma18G49060,Glyma08G18760,Glyma14G03040,Glyma16G33950,Glyma01G34780,Glyma18G47140,Glyma06G18700,Glyma20G27720,Glyma13G42950,Glyma18G50440,Glyma06G40710,Glyma06G40920,Glyma05G23260,Glyma18G46930,Glyma13G10530,Glyma05G01460,Glyma14G36960,Glyma18G51960,Glyma13G10040,Glyma17G04470,Glyma15G17430,Glyma09G13540,Glyma06G40740,Glyma15G42030,Glyma06G02010,Glyma09G26710,Glyma15G14030,Glyma09G05710,Glyma16G10080,Glyma07G31140,Glyma15G00670,Glyma17G09120,Glyma03G00540,Glyma18G04440,Glyma06G40980,Glyma03G05400,Glyma18G08870,Glyma07G12130,Glyma02G48100,Glyma12G34890,Glyma15G00340,Glyma10G32990,Glyma12G33340,Glyma06G40350,Glyma15G00950,Glyma18G10610,Glyma07G10460,Glyma14G29130,Glyma08G15360,Glyma15G37140,Glyma11G27200,Glyma13G10200,Glyma19G35270,Glyma11G05140,Glyma06G40670,Glyma04G01110,Glyma09G40650,Glyma18G51950,Glyma05G03440,Glyma15G04850,Glyma16G32710,Glyma16G10760,Glyma19G00650,Glyma18G42700,Glyma07G09530,Glyma04G34140,Glyma10G35610,Glyma10G02020,Glyma06G05910,Glyma03G32160,Glyma13G23610,Glyma13G26250,Glyma05G30030,Glyma13G00370,Glyma19G43290,Glyma03G37760,Glyma20G27660,Glyma17G30980,Glyma08G13060,Glyma17G34170,Glyma15G14210,Glyma12G17450,Glyma12G17360,Glyma19G34250,Glyma04G39670,Glyma03G42000,Glyma08G09540,Glyma10G04480,Glyma13G06490,Glyma11G34490,Glyma02G14310,Glyma20G27510,Glyma05G30260,Glyma10G35310,Glyma08G23150,Glyma09G37650,Glyma07G02940,Glyma14G38650,Glyma18G10470,Glyma12G36440,Glyma15G04280,Glyma08G08000,Glyma15G04350,Glyma19G04140,Glyma09G08180,Glyma08G05720,Glyma08G43170

GO:001883	purine nucleoside binding	355/1695	4155/27682	8.51E-12	Glyma13G41790,Glyma04G04510,Glyma04G09210,Glyma20G26160,Glyma14G36660,Glyma13G36600,Glyma18G00700,Glyma17G08610,Glyma19G39680,Glyma12G06530,Glyma07G08120,Glyma01G03410,Glyma09G30430,Glyma12G34330,Glyma10G30060,Glyma02G03070,Glyma13G26000,Glyma15G14020,Glyma11G15520,Glyma18G50510,Glyma13G18690,Glyma16G08560,Glyma12G08900,Glyma16G25010,Glyma0028S00210,Glyma17G34190,Glyma13G05430,Glyma18G01960,Glyma06G40690,Glyma09G03190,Glyma17G08540,Glyma17G01730,Glyma20G16170,Glyma11G20180,Glyma13G25420,Glyma07G07550,Glyma19G31720,Glyma13G23840,Glyma06G39990,Glyma13G17440,Glyma04G04380,Glyma05G34180,Glyma02G45800,Glyma08G03240,Glyma16G34030,Glyma17G10420,Glyma14G33400,Glyma02G11110,Glyma06G17560,Glyma18G07080,Glyma08G08500,Glyma13G21820,Glyma07G32200,Glyma13G35790,Glyma19G39580,Glyma06G41880,Glyma06G45590,Glyma19G41210,Glyma06G15900,Glyma18G48600,Glyma07G07110,Glyma05G33240,Glyma04G05920,Glyma13G21030,Glyma01G07970,Glyma06G21140,Glyma07G01890,Glyma02G47230,Glyma18G09670,Glyma16G24940,Glyma16G04910,Glyma15G36110,Glyma20G23010,Glyma18G11590,Glyma15G10370,Glyma15G14080,Glyma04G41060,Glyma08G09050,Glyma08G46130,Glyma05G32070,Glyma09G24410,Glyma18G50450,Glyma14G02740,Glyma06G41430,Glyma08G41510,Glyma18G40680,Glyma10G07500,Glyma05G27850,Glyma15G23150,Glyma05G30650,Glyma11G34210,Glyma17G34410,Glyma15G20000,Glyma04G01440,Glyma15G37390,Glyma11G19120,Glyma16G21050,Glyma06G08480,Glyma09G33120,Glyma18G09290,Glyma09G19730,Glyma14G04930,Glyma11G37910,Glyma20G37930,Glyma11G09060,Glyma18G16060,Glyma13G16540,Glyma09G34860,Glyma20G27860,Glyma18G15150,Glyma08G39070,Glyma08G12540,Glyma12G32880,Glyma03G05640,Glyma01G10100,Glyma14G06570,Glyma03G02310,Glyma15G02520,Glyma01G27440,Glyma11G04310,Glyma18G06990,Glyma03G30310,Glyma15G37320,Glyma15G13290,Glyma16G32640,Glyma12G17340,Glyma13G40550,Glyma13G06620,Glyma17G38210,Glyma15G34810,Glyma06G21910,Glyma07G09260,Glyma09G08370,Glyma13G25970,Glyma07G11880,Glyma08G13040,Glyma16G32700,Glyma13G38950,Glyma08G13150,Glyma18G45440,Glyma17G33470,Glyma06G47650,Glyma07G00670,Glyma20G38380,Glyma06G20360,Glyma20G34060,Glyma20G38610,Glyma16G02290,Glyma14G12710,Glyma01G42240,Glyma16G22370,Glyma02G09080,Glyma05G30800,Glyma16G27250,Glyma08G40030,Glyma18G52480,Glyma17G02080,Glyma03G33590,Glyma16G33590,Glyma03G36310,Glyma14G07750,Glyma02G40850,Glyma16G02340,Glyma10G38450.2,Glyma07G00340,Glyma11G09070,Glyma04G43190,Glyma19G31950,Glyma18G44800,Glyma15G14200,Glyma11G02150,Glyma18G50540,Glyma01G42770,Glyma05G21420,Glyma13G43480,Glyma17G09700,Glyma16G29040,Glyma18G09980,Glyma01G43190,Glyma02G06700,Glyma10G41990,Glyma12G16450,Glyma03G32530,Glyma14G00380,Glyma20G06780,Glyma01G41150,Glyma04G36240,Glyma14G10790,Glyma19G34960,Glyma20G29060,Glyma09G35140,Glyma16G32830,Glyma07G16260,Glyma08G44620,Glyma08G40920,Glyma06G20200,Glyma20G27790,Glyma09G05550,Glyma09G28260,Glyma09G02930,Glyma19G38970,Glyma20G27800,Glyma17G34060,Glyma15G37290,Glyma09G37580,Glyma20G35970,Glyma12G03140,Glyma03G04780,Glyma01G39380,Glyma10G38810,Glyma18G45200,Glyma18G53590,Glyma17G16070,Glyma02G46070,Glyma01G00960,Glyma07G30260,Glyma09G40150,Glyma08G06730,Glyma08G08550,Glyma18G08220,Glyma02G46670,Glyma14G05060,Glyma02G03760,Glyma08G06520,Glyma16G03780,Glyma09G38730,Glyma16G07060,Glyma03G29230,Glyma07G39300,Glyma05G02470,Glyma18G38470,Glyma06G40780,Glyma10G32800,Glyma04G27730,Glyma06G47870,Glyma11G19880,Glyma18G47590,Glyma07G04560,Glyma06G40110,Glyma06G08880,Glyma19G44690,Glyma13G02620,Glyma04G06610,Glyma18G50650,Glyma09G30070,Glyma10G08040,Glyma06G45340,Glyma20G30100,Glyma18G49060,Glyma08G18760,Glyma14G03040,Glyma16G33950,Glyma01G34780,Glyma18G47140,Glyma06G18700,Glyma20G27720,Glyma13G42950,Glyma18G50440,Glyma06G40710,Glyma06G40920,Glyma05G23260,Glyma18G46930,Glyma13G10530,Glyma05G01460,Glyma14G36960,Glyma18G51960,Glyma13G10040,Glyma17G04470,Glyma15G17430,Glyma09G13540,Glyma06G40740,Glyma15G42030,Glyma06G02010,Glyma09G26710,Glyma15G14030,Glyma09G05710,Glyma16G10080,Glyma07G31140,Glyma15G00670,Glyma17G09120,Glyma03G00540,Glyma18G04440,Glyma06G40980,Glyma03G05400,Glyma18G08870,Glyma07G12130,Glyma02G48100,Glyma12G34890,Glyma15G00340,Glyma10G32990,Glyma12G33340,Glyma06G40350,Glyma15G00950,Glyma18G10610,Glyma07G10460,Glyma14G29130,Glyma08G15360,Glyma15G37140,Glyma11G27200,Glyma13G10200,Glyma19G35270,Glyma11G05140,Glyma06G40670,Glyma04G01110,Glyma09G40650,Glyma18G51950,Glyma05G03440,Glyma15G04850,Glyma16G32710,Glyma16G10760,Glyma19G00650,Glyma18G42700,Glyma07G09530,Glyma04G34140,Glyma10G35610,Glyma10G02020,Glyma06G05910,Glyma03G32160,Glyma13G23610,Glyma13G26250,Glyma05G30030,Glyma13G00370,Glyma19G43290,Glyma03G37760,Glyma20G27660,Glyma17G30980,Glyma08G13060,Glyma17G34170,Glyma15G14210,Glyma12G17450,Glyma12G17360,Glyma19G34250,Glyma04G39670,Glyma03G42000,Glyma08G09540,Glyma10G04480,Glyma13G06490,Glyma11G34490,Glyma02G14310,Glyma20G27510,Glyma05G30260,Glyma10G35310,Glyma08G23150,Glyma09G37650,Glyma07G02940,Glyma14G38650,Glyma18G10470,Glyma12G36440,Glyma15G04280,Glyma08G08000,Glyma15G04350,Glyma19G04140,Glyma09G08180,Glyma08G05720,Glyma08G43170

GO:000166	nucleotide binding	399/1695	4791/27682	9.81E-12	Glyma13G41790,Glyma04G04510,Glyma18G00700,Glyma17G08610,Glyma04G38360,Glyma16G09890,Glyma06G07410,Glyma08G06570,Glyma02G03070,Glyma13G26000,Glyma06G16700,Glyma11G15520,Glyma12G08900,Glyma0028S00210,Glyma05G34540,Glyma17G08540,Glyma17G01730,Glyma20G16170,Glyma13G25420,Glyma19G31720,Glyma14G36420,Glyma13G23840,Glyma04G04380,Glyma05G34180,Glyma08G03240,Glyma14G33400,Glyma14G05020,Glyma06G17560,Glyma18G07080,Glyma13G21820,Glyma06G41880,Glyma13G35790,Glyma06G45590,Glyma18G48600,Glyma05G33240,Glyma04G05920,Glyma13G21030,Glyma01G07970,Glyma02G47230,Glyma18G09670,Glyma16G04910,Glyma16G24940,Glyma15G36110,Glyma20G23010,Glyma15G10370,Glyma18G11590,Glyma11G13120,Glyma08G46130,Glyma18G50450,Glyma04G07370,Glyma06G41430,Glyma18G40680,Glyma02G38280,Glyma05G30650,Glyma15G37390,Glyma11G19120,Glyma06G08480,Glyma11G37910,Glyma11G09060,Glyma09G34860,Glyma20G27860,Glyma08G12540,Glyma01G10100,Glyma14G06570,Glyma15G02520,Glyma11G04310,Glyma15G37320,Glyma16G32640,Glyma12G17340,Glyma13G40550,Glyma09G08370,Glyma07G09260,Glyma06G21910,Glyma14G40380,Glyma13G38950,Glyma18G45440,Glyma08G13150,Glyma17G33470,Glyma07G00670,Glyma06G20360,Glyma20G38610,Glyma16G02290,Glyma14G12710,Glyma16G22370,Glyma02G09080,Glyma08G40030,Glyma18G52480,Glyma03G33590,Glyma02G16630,Glyma03G36310,Glyma02G40850,Glyma16G02340,Glyma10G38450.2,Glyma11G09070,Glyma04G43190,Glyma19G31950,Glyma18G44800,Glyma15G14200,Glyma01G42770,Glyma05G21420,Glyma14G00380,Glyma03G32530,Glyma19G34960,Glyma20G29060,Glyma13G25570,Glyma08G40920,Glyma09G05550,Glyma09G28260,Glyma19G38970,Glyma09G37580,Glyma12G03140,Glyma03G04780,Glyma01G39380,Glyma10G38810,Glyma02G46070,Glyma07G30260,Glyma08G06730,Glyma08G08550,Glyma02G46670,Glyma14G05060,Glyma08G06520,Glyma16G03780,Glyma09G38730,Glyma16G07060,Glyma10G32800,Glyma04G27730,Glyma07G04560,Glyma18G47590,Glyma06G08880,Glyma06G40110,Glyma13G02620,Glyma19G44690,Glyma15G07040,Glyma18G50650,Glyma06G45340,Glyma10G08040,Glyma08G18760,Glyma11G04350,Glyma16G33950,Glyma14G03040,Glyma01G34780,Glyma06G18700,Glyma17G01310,Glyma20G27720,Glyma18G50440,Glyma13G36900,Glyma18G46930,Glyma19G38110,Glyma13G10530,Glyma05G01460,Glyma14G36960,Glyma12G34500,Glyma18G51960,Glyma17G04470,Glyma13G10040,Glyma15G17430,Glyma15G42030,Glyma09G26710,Glyma15G14030,Glyma09G05710,Glyma18G04440,Glyma06G40980,Glyma03G05400,Glyma07G30750,Glyma07G12130,Glyma12G33340,Glyma15G00950,Glyma13G32300,Glyma15G37140,Glyma11G27200,Glyma13G10200,Glyma06G40670,Glyma04G01110,Glyma18G51950,Glyma09G40650,Glyma15G04850,Glyma16G32710,Glyma16G10760,Glyma04G34140,Glyma10G35610,Glyma10G02020,Glyma13G26250,Glyma05G30030,Glyma13G00370,Glyma19G43290,Glyma08G05120,Glyma12G17360,Glyma04G39670,Glyma02G14310,Glyma11G34490,Glyma13G06490,Glyma20G27510,Glyma08G23150,Glyma05G31810,Glyma09G37650,Glyma12G36440,Glyma08G08000,Glyma09G08180,Glyma04G09210,Glyma20G26160,Glyma14G36660,Glyma13G36600,Glyma10G03170,Glyma13G24260,Glyma19G39680,Glyma12G06530,Glyma07G08120,Glyma01G03410,Glyma09G30430,Glyma12G34330,Glyma10G30060,Glyma15G14020,Glyma06G19630,Glyma13G18690,Glyma18G50510,Glyma16G08560,Glyma16G25010,Glyma17G34190,Glyma13G05430,Glyma06G40690,Glyma18G01960,Glyma02G10710,Glyma09G03190,Glyma11G20180,Glyma07G07550,Glyma06G39990,Glyma13G17440,Glyma02G45800,Glyma16G34030,Glyma17G10420,Glyma02G11110,Glyma08G08500,Glyma19G39580,Glyma07G32200,Glyma06G15900,Glyma19G41210,Glyma07G07110,Glyma07G01890,Glyma06G21140,Glyma12G01380,Glyma15G14080,Glyma04G41060,Glyma08G09050,Glyma05G32070,Glyma09G24410,Glyma14G02740,Glyma05G33830,Glyma08G41510,Glyma10G07500,Glyma05G27850,Glyma15G23150,Glyma11G34210,Glyma17G34410,Glyma04G01440,Glyma15G20000,Glyma16G21050,Glyma09G33120,Glyma09G19730,Glyma18G09290,Glyma14G04930,Glyma20G37930,Glyma18G16060,Glyma13G16540,Glyma19G07410,Glyma18G15150,Glyma12G32880,Glyma08G39070,Glyma03G05640,Glyma03G02310,Glyma01G27440,Glyma18G06990,Glyma03G30310,Glyma12G09220,Glyma15G13290,Glyma04G35110,Glyma17G38210,Glyma13G06620,Glyma13G25970,Glyma15G34810,Glyma07G11880,Glyma08G13040,Glyma16G32700,Glyma06G47650,Glyma08G21530,Glyma20G38380,Glyma20G34060,Glyma20G29840,Glyma07G29630,Glyma05G08770,Glyma01G42240,Glyma05G30800,Glyma16G27250,Glyma17G02080,Glyma16G33590,Glyma08G16680,Glyma14G07750,Glyma07G00340,Glyma11G02150,Glyma18G50540,Glyma13G43480,Glyma17G09700,Glyma16G29040,Glyma18G09980,Glyma01G43190,Glyma02G06700,Glyma12G16450,Glyma10G41990,Glyma01G41150,Glyma20G06780,Glyma14G10790,Glyma04G36240,Glyma09G35140,Glyma16G32830,Glyma07G16260,Glyma08G44620,Glyma06G20200,Glyma20G27790,Glyma09G02930,Glyma20G27800,Glyma17G34060,Glyma15G37290,Glyma09G02960,Glyma20G35970,Glyma18G45200,Glyma18G53590,Glyma17G16070,Glyma01G00960,Glyma09G40150,Glyma18G08220,Glyma02G03760,Glyma17G03980,Glyma03G29230,Glyma07G39300,Glyma05G02470,Glyma18G38470,Glyma06G40780,Glyma18G52110,Glyma06G47870,Glyma11G19880,Glyma04G06610,Glyma09G30070,Glyma20G30100,Glyma18G49060,Glyma18G47140,Glyma13G42950,Glyma06G40710,Glyma06G40920,Glyma05G23260,Glyma09G13540,Glyma06G40740,Glyma06G02010,Glyma15G00670,Glyma07G31140,Glyma16G10080,Glyma17G09120,Glyma03G00540,Glyma18G08870,Glyma02G48100,Glyma12G34890,Glyma15G00340,Glyma10G32990,Glyma06G40350,Glyma18G10610,Glyma08G15360,Glyma14G29130,Glyma07G10460,Glyma19G35270,Glyma11G05140,Glyma05G03440,Glyma05G35800,Glyma19G00650,Glyma07G09530,Glyma18G42700,Glyma06G05910,Glyma03G32160,Glyma13G23610,Glyma03G37760,Glyma20G27660,Glyma16G27030,Glyma17G30980,Glyma08G13060,Glyma17G34170,Glyma15G14210,Glyma12G17450,Glyma19G34250,Glyma16G08460,Glyma03G42000,Glyma08G09540,Glyma10G04480,Glyma05G30260,Glyma10G35310,Glyma07G02940,Glyma14G38650,Glyma18G10470,Glyma15G04280,Glyma15G04350,Glyma19G04140,Glyma08G05720,Glyma08G43170

GO:032555	purine ribonucleotide binding	363/1695	4325/27682	4.61E-11	Glyma13G41790,Glyma04G04510,Glyma04G09210,Glyma20G26160,Glyma14G36660,Glyma13G36600,Glyma10G03170,Glyma18G00700,Glyma19G39680,Glyma04G38360,Glyma13G24260,Glyma12G06530,Glyma06G07410,Glyma07G08120,Glyma01G03410,Glyma09G30430,Glyma12G34330,Glyma10G30060,Glyma02G03070,Glyma06G16700,Glyma13G26000,Glyma06G19630,Glyma11G15520,Glyma18G50510,Glyma13G18690,Glyma16G08560,Glyma12G08900,Glyma16G25010,Glyma0028S00210,Glyma17G34190,Glyma13G05430,Glyma18G01960,Glyma06G40690,Glyma05G34540,Glyma09G03190,Glyma17G08540,Glyma17G01730,Glyma20G16170,Glyma11G20180,Glyma13G25420,Glyma07G07550,Glyma19G31720,Glyma13G23840,Glyma06G39990,Glyma13G17440,Glyma04G04380,Glyma05G34180,Glyma02G45800,Glyma08G03240,Glyma16G34030,Glyma17G10420,Glyma14G33400,Glyma02G11110,Glyma06G17560,Glyma18G07080,Glyma13G21820,Glyma07G32200,Glyma13G35790,Glyma19G39580,Glyma06G41880,Glyma06G45590,Glyma19G41210,Glyma06G15900,Glyma18G48600,Glyma07G07110,Glyma05G33240,Glyma04G05920,Glyma13G21030,Glyma01G07970,Glyma06G21140,Glyma07G01890,Glyma02G47230,Glyma18G09670,Glyma16G24940,Glyma16G04910,Glyma15G36110,Glyma20G23010,Glyma18G11590,Glyma15G10370,Glyma04G41060,Glyma08G09050,Glyma08G46130,Glyma05G32070,Glyma09G24410,Glyma18G50450,Glyma04G07370,Glyma14G02740,Glyma06G41430,Glyma08G41510,Glyma05G33830,Glyma18G40680,Glyma10G07500,Glyma05G27850,Glyma05G30650,Glyma11G34210,Glyma17G34410,Glyma15G20000,Glyma04G01440,Glyma15G37390,Glyma11G19120,Glyma16G21050,Glyma06G08480,Glyma09G33120,Glyma18G09290,Glyma09G19730,Glyma14G04930,Glyma11G37910,Glyma20G37930,Glyma11G09060,Glyma18G16060,Glyma13G16540,Glyma09G34860,Glyma20G27860,Glyma18G15150,Glyma08G39070,Glyma08G12540,Glyma12G32880,Glyma03G05640,Glyma01G10100,Glyma14G06570,Glyma03G02310,Glyma15G02520,Glyma01G27440,Glyma11G04310,Glyma18G06990,Glyma03G30310,Glyma15G37320,Glyma15G13290,Glyma16G32640,Glyma12G17340,Glyma13G40550,Glyma04G35110,Glyma13G06620,Glyma17G38210,Glyma15G34810,Glyma06G21910,Glyma07G09260,Glyma09G08370,Glyma13G25970,Glyma07G11880,Glyma14G40380,Glyma08G13040,Glyma16G32700,Glyma13G38950,Glyma08G13150,Glyma18G45440,Glyma17G33470,Glyma06G47650,Glyma07G00670,Glyma20G38380,Glyma06G20360,Glyma20G34060,Glyma20G29840,Glyma20G38610,Glyma16G02290,Glyma14G12710,Glyma01G42240,Glyma16G22370,Glyma02G09080,Glyma05G30800,Glyma16G27250,Glyma08G40030,Glyma18G52480,Glyma17G02080,Glyma03G33590,Glyma02G16630,Glyma16G33590,Glyma03G36310,Glyma14G07750,Glyma08G16680,Glyma02G40850,Glyma16G02340,Glyma10G38450.2,Glyma07G00340,Glyma11G09070,Glyma04G43190,Glyma19G31950,Glyma18G44800,Glyma11G02150,Glyma18G50540,Glyma01G42770,Glyma05G21420,Glyma13G43480,Glyma17G09700,Glyma16G29040,Glyma18G09980,Glyma02G06700,Glyma10G41990,Glyma12G16450,Glyma03G32530,Glyma14G00380,Glyma20G06780,Glyma01G41150,Glyma04G36240,Glyma14G10790,Glyma20G29060,Glyma09G35140,Glyma16G32830,Glyma07G16260,Glyma08G44620,Glyma08G40920,Glyma06G20200,Glyma20G27790,Glyma09G05550,Glyma09G28260,Glyma09G02930,Glyma19G38970,Glyma20G27800,Glyma17G34060,Glyma15G37290,Glyma09G37580,Glyma20G35970,Glyma12G03140,Glyma03G04780,Glyma01G39380,Glyma10G38810,Glyma18G45200,Glyma18G53590,Glyma17G16070,Glyma02G46070,Glyma01G00960,Glyma07G30260,Glyma09G40150,Glyma08G06730,Glyma18G08220,Glyma02G46670,Glyma14G05060,Glyma02G03760,Glyma08G06520,Glyma16G03780,Glyma09G38730,Glyma16G07060,Glyma03G29230,Glyma07G39300,Glyma05G02470,Glyma18G38470,Glyma06G40780,Glyma10G32800,Glyma04G27730,Glyma06G47870,Glyma11G19880,Glyma18G47590,Glyma07G04560,Glyma06G40110,Glyma06G08880,Glyma19G44690,Glyma13G02620,Glyma04G06610,Glyma18G50650,Glyma09G30070,Glyma10G08040,Glyma20G30100,Glyma18G49060,Glyma11G04350,Glyma08G18760,Glyma14G03040,Glyma16G33950,Glyma01G34780,Glyma18G47140,Glyma06G18700,Glyma20G27720,Glyma17G01310,Glyma13G42950,Glyma18G50440,Glyma06G40710,Glyma13G36900,Glyma06G40920,Glyma05G23260,Glyma18G46930,Glyma19G38110,Glyma13G10530,Glyma05G01460,Glyma14G36960,Glyma18G51960,Glyma13G10040,Glyma17G04470,Glyma15G17430,Glyma09G13540,Glyma06G40740,Glyma15G42030,Glyma06G02010,Glyma09G26710,Glyma09G05710,Glyma16G10080,Glyma07G31140,Glyma15G00670,Glyma17G09120,Glyma03G00540,Glyma18G04440,Glyma06G40980,Glyma03G05400,Glyma18G08870,Glyma07G12130,Glyma02G48100,Glyma12G34890,Glyma15G00340,Glyma10G32990,Glyma12G33340,Glyma06G40350,Glyma15G00950,Glyma18G10610,Glyma07G10460,Glyma14G29130,Glyma08G15360,Glyma15G37140,Glyma11G27200,Glyma13G10200,Glyma19G35270,Glyma11G05140,Glyma06G40670,Glyma04G01110,Glyma09G40650,Glyma18G51950,Glyma05G03440,Glyma15G04850,Glyma16G32710,Glyma16G10760,Glyma19G00650,Glyma18G42700,Glyma07G09530,Glyma04G34140,Glyma10G35610,Glyma10G02020,Glyma06G05910,Glyma03G32160,Glyma13G23610,Glyma13G26250,Glyma05G30030,Glyma13G00370,Glyma19G43290,Glyma03G37760,Glyma20G27660,Glyma16G27030,Glyma08G05120,Glyma17G30980,Glyma08G13060,Glyma17G34170,Glyma12G17450,Glyma12G17360,Glyma19G34250,Glyma04G39670,Glyma03G42000,Glyma10G04480,Glyma13G06490,Glyma11G34490,Glyma02G14310,Glyma20G27510,Glyma05G30260,Glyma10G35310,Glyma05G31810,Glyma08G23150,Glyma09G37650,Glyma07G02940,Glyma14G38650,Glyma18G10470,Glyma12G36440,Glyma15G04280,Glyma08G08000,Glyma15G04350,Glyma19G04140,Glyma09G08180,Glyma08G05720,Glyma08G43170
GO:032553	ribonucleotide binding	363/1695	4325/27682	4.61E-11	Glyma13G41790,Glyma04G04510,Glyma04G09210,Glyma20G26160,Glyma14G36660,Glyma13G36600,Glyma10G03170,Glyma18G00700,Glyma19G39680,Glyma04G38360,Glyma13G24260,Glyma12G06530,Glyma06G07410,Glyma07G08120,Glyma01G03410,Glyma09G30430,Glyma12G34330,Glyma10G30060,Glyma02G03070,Glyma06G16700,Glyma13G26000,Glyma06G19630,Glyma11G15520,Glyma18G50510,Glyma13G18690,Glyma16G08560,Glyma12G08900,Glyma16G25010,Glyma0028S00210,Glyma17G34190,Glyma13G05430,Glyma18G01960,Glyma06G40690,Glyma05G34540,Glyma09G03190,Glyma17G08540,Glyma17G01730,Glyma20G16170,Glyma11G20180,Glyma13G25420,Glyma07G07550,Glyma19G31720,Glyma13G23840,Glyma06G39990,Glyma13G17440,Glyma04G04380,Glyma05G34180,Glyma02G45800,Glyma08G03240,Glyma16G34030,Glyma17G10420,Glyma14G33400,Glyma02G11110,Glyma06G17560,Glyma18G07080,Glyma13G21820,Glyma07G32200,Glyma13G35790,Glyma19G39580,Glyma06G41880,Glyma06G45590,Glyma19G41210,Glyma06G15900,Glyma18G48600,Glyma07G07110,Glyma05G33240,Glyma04G05920,Glyma13G21030,Glyma01G07970,Glyma06G21140,Glyma07G01890,Glyma02G47230,Glyma18G09670,Glyma16G24940,Glyma16G04910,Glyma15G36110,Glyma20G23010,Glyma18G11590,Glyma15G10370,Glyma04G41060,Glyma08G09050,Glyma08G46130,Glyma05G32070,Glyma09G24410,Glyma18G50450,Glyma04G07370,Glyma14G02740,Glyma06G41430,Glyma08G41510,Glyma05G33830,Glyma18G40680,Glyma10G07500,Glyma05G27850,Glyma05G30650,Glyma11G34210,Glyma17G34410,Glyma15G20000,Glyma04G01440,Glyma15G37390,Glyma11G19120,Glyma16G21050,Glyma06G08480,Glyma09G33120,Glyma18G09290,Glyma09G19730,Glyma14G04930,Glyma11G37910,Glyma20G37930,Glyma11G09060,Glyma18G16060,Glyma13G16540,Glyma09G34860,Glyma20G27860,Glyma18G15150,Glyma08G39070,Glyma08G12540,Glyma12G32880,Glyma03G05640,Glyma01G10100,Glyma14G06570,Glyma03G02310,Glyma15G02520,Glyma01G27440,Glyma11G04310,Glyma18G06990,Glyma03G30310,Glyma15G37320,Glyma15G13290,Glyma16G32640,Glyma12G17340,Glyma13G40550,Glyma04G35110,Glyma13G06620,Glyma17G38210,Glyma15G34810,Glyma06G21910,Glyma07G09260,Glyma09G08370,Glyma13G25970,G

					lyma07G11880,Glyma14G40380,Glyma08G13040,Glyma16G32700,Glyma13G38950,Glyma08G13150,Glyma18G45440,Glyma17G33470,Glyma06G47650,Glyma07G00670,Glyma20G38380,Glyma06G20360,Glyma20G34060,Glyma20G29840,Glyma20G38610,Glyma16G02290,Glyma14G12710,Glyma01G42240,Glyma16G22370,Glyma02G09080,Glyma05G30800,Glyma16G27250,Glyma08G40030,Glyma18G52480,Glyma17G02080,Glyma03G33590,Glyma02G16630,Glyma16G33590,Glyma03G36310,Glyma14G07750,Glyma08G16680,Glyma02G40850,Glyma16G02340,Glyma10G38450.2,Glyma07G00340,Glyma11G09070,Glyma04G43190,Glyma19G31950,Glyma18G44800,Glyma11G02150,Glyma18G50540,Glyma01G42770,Glyma05G21420,Glyma13G43480,Glyma17G09700,Glyma16G29040,Glyma18G09980,Glyma02G06700,Glyma10G41990,Glyma12G16450,Glyma03G32530,Glyma14G00380,Glyma20G06780,Glyma01G41150,Glyma04G36240,Glyma14G10790,Glyma20G29060,Glyma09G35140,Glyma16G32830,Glyma07G16260,Glyma08G44620,Glyma08G40920,Glyma06G20200,Glyma20G27790,Glyma09G05550,Glyma09G28260,Glyma09G02930,Glyma19G38970,Glyma20G27800,Glyma17G34060,Glyma15G37290,Glyma09G37580,Glyma20G35970,Glyma12G03140,Glyma03G04780,Glyma01G39380,Glyma10G38810,Glyma18G45200,Glyma18G53590,Glyma17G16070,Glyma02G46070,Glyma01G00960,Glyma07G30260,Glyma09G40150,Glyma08G06730,Glyma18G08220,Glyma02G46670,Glyma14G05060,Glyma02G03760,Glyma08G06520,Glyma16G03780,Glyma09G38730,Glyma16G07060,Glyma03G29230,Glyma07G39300,Glyma05G02470,Glyma18G38470,Glyma06G40780,Glyma10G32800,Glyma04G27730,Glyma06G47870,Glyma11G19880,Glyma18G47590,Glyma07G04560,Glyma06G40110,Glyma06G08880,Glyma19G44690,Glyma13G02620,Glyma04G06610,Glyma18G50650,Glyma09G30070,Glyma10G08040,Glyma20G30100,Glyma18G49060,Glyma11G04350,Glyma08G18760,Glyma14G03040,Glyma16G33950,Glyma01G34780,Glyma18G47140,Glyma06G18700,Glyma20G27720,Glyma17G01310,Glyma13G42950,Glyma18G50440,Glyma06G40710,Glyma13G36900,Glyma06G40920,Glyma05G23260,Glyma18G46930,Glyma19G38110,Glyma13G10530,Glyma05G01460,Glyma14G36960,Glyma18G51960,Glyma13G10040,Glyma17G04470,Glyma15G17430,Glyma09G13540,Glyma06G40740,Glyma15G42030,Glyma06G02010,Glyma09G26710,Glyma09G05710,Glyma16G10080,Glyma07G31140,Glyma15G00670,Glyma17G09120,Glyma03G00540,Glyma18G04440,Glyma06G40980,Glyma03G05400,Glyma18G08870,Glyma07G12130,Glyma02G48100,Glyma12G34890,Glyma15G00340,Glyma10G32990,Glyma12G33340,Glyma06G40350,Glyma15G00950,Glyma18G10610,Glyma07G10460,Glyma14G29130,Glyma08G15360,Glyma15G37140,Glyma11G27200,Glyma13G10200,Glyma19G35270,Glyma11G05140,Glyma06G40670,Glyma04G01110,Glyma09G40650,Glyma18G51950,Glyma05G03440,Glyma15G04850,Glyma16G32710,Glyma16G10760,Glyma19G00650,Glyma18G42700,Glyma07G09530,Glyma04G34140,Glyma10G35610,Glyma10G02020,Glyma06G05910,Glyma03G32160,Glyma13G23610,Glyma13G26250,Glyma05G30030,Glyma13G00370,Glyma19G43290,Glyma03G37760,Glyma20G27660,Glyma16G27030,Glyma08G05120,Glyma17G30980,Glyma08G13060,Glyma17G34170,Glyma12G17450,Glyma12G17360,Glyma19G34250,Glyma04G39670,Glyma03G42000,Glyma10G04480,Glyma13G06490,Glyma11G34490,Glyma02G14310,Glyma20G27510,Glyma05G30260,Glyma10G35310,Glyma05G31810,Glyma08G23150,Glyma09G37650,Glyma07G02940,Glyma14G38650,Glyma18G10470,Glyma12G36440,Glyma15G04280,Glyma08G08000,Glyma15G04350,Glyma19G04140,Glyma09G08180,Glyma08G05720,Glyma08G43170
GO:017076	purine nucleotide binding	377/1695	4576/27682	1.87E-10	Glyma13G41790,Glyma04G04510,Glyma04G09210,Glyma20G26160,Glyma14G36660,Glyma13G36600,Glyma10G03170,Glyma18G00700,Glyma17G08610,Glyma19G39680,Glyma04G38360,Glyma13G24260,Glyma12G06530,Glyma06G07410,Glyma07G08120,Glyma01G03410,Glyma09G30430,Glyma12G34330,Glyma10G30060,Glyma02G03070,Glyma06G16700,Glyma13G26000,Glyma15G14020,Glyma06G19630,Glyma11G15520,Glyma18G50510,Glyma13G18690,Glyma16G08560,Glyma12G08900,Glyma16G25010,Glyma0028S00210,Glyma17G34190,Glyma13G05430,Glyma18G01960,Glyma06G40690,Glyma05G34540,Glyma09G03190,Glyma17G08540,Glyma17G01730,Glyma20G16170,Glyma13G25420,Glyma11G20180,Glyma07G07550,Glyma19G31720,Glyma13G23840,Glyma06G39990,Glyma13G17440,Glyma04G04380,Glyma05G34180,Glyma02G45800,Glyma08G03240,Glyma16G34030,Glyma17G10420,Glyma14G33400,Glyma02G11110,Glyma06G17560,Glyma13G21820,Glyma18G07080,Glyma08G08500,Glyma07G32200,Glyma13G35790,Glyma19G39580,Glyma06G41880,Glyma06G45590,Glyma19G41210,Glyma06G15900,Glyma18G48600,Glyma07G07110,Glyma05G33240,Glyma04G05920,Glyma01G07970,Glyma13G21030,Glyma06G21140,Glyma07G01890,Glyma02G47230,Glyma18G09670,Glyma16G24940,Glyma16G04910,Glyma15G36110,Glyma20G23010,Glyma18G11590,Glyma15G10370,Glyma15G14080,Glyma11G13120,Glyma04G41060,Glyma08G09050,Glyma08G46130,Glyma05G32070,Glyma09G24410,Glyma04G07370,Glyma18G50450,Glyma14G02740,Glyma06G41430,Glyma08G41510,Glyma05G33830,Glyma18G40680,Glyma10G07500,Glyma05G27850,Glyma15G23150,Glyma05G30650,Glyma11G34210,Glyma17G34410,Glyma15G20000,Glyma04G01440,Glyma15G37390,Glyma11G19120,Glyma16G21050,Glyma06G08480,Glyma09G33120,Glyma18G09290,Glyma09G19730,Glyma14G04930,Glyma11G37910,Glyma20G37930,Glyma11G09060,Glyma18G16060,Glyma13G16540,Glyma09G34860,Glyma20G27860,Glyma18G15150,Glyma08G39070,Glyma08G12540,Glyma12G32880,Glyma03G05640,Glyma01G10100,Glyma14G06570,Glyma03G02310,Glyma15G02520,Glyma01G27440,Glyma11G04310,Glyma18G06990,Glyma03G30310,Glyma15G37320,Glyma15G13290,Glyma16G32640,Glyma12G17340,Glyma13G40550,Glyma04G35110,Glyma13G06620,Glyma17G38210,Glyma15G34810,Glyma06G21910,Glyma07G09260,Glyma09G08370,Glyma13G25970,Glyma07G11880,Glyma14G40380,Glyma08G13040,Glyma16G32700,Glyma13G38950,Glyma08G13150,Glyma18G45440,Glyma17G33470,Glyma06G47650,Glyma07G00670,Glyma20G38380,Glyma06G20360,Glyma20G34060,Glyma20G29840,Glyma20G38610,Glyma16G02290,Glyma14G12710,Glyma01G42240,Glyma16G22370,Glyma02G09080,Glyma05G30800,Glyma16G27250,Glyma08G40030,Glyma18G52480,Glyma17G02080,Glyma03G33590,Glyma02G16630,Glyma16G33590,Glyma03G36310,Glyma14G07750,Glyma08G16680,Glyma02G40850,Glyma16G02340,Glyma10G38450.2,Glyma07G00340,Glyma11G09070,Glyma04G43190,Glyma19G31950,Glyma18G44800,Glyma15G14200,Glyma11G02150,Glyma18G50540,Glyma01G42770,Glyma05G21420,Glyma13G43480,Glyma17G09700,Glyma16G29040,Glyma18G09980,Glyma01G43190,Glyma02G06700,Glyma10G41990,Glyma12G16450,Glyma03G32530,Glyma14G00380,Glyma20G06780,Glyma01G41150,Glyma04G36240,Glyma14G10790,Glyma19G34960,Glyma20G29060,Glyma09G35140,Glyma16G32830,Glyma07G16260,Glyma08G44620,Glyma08G40920,Glyma06G20200,Glyma20G27790,Glyma09G05550,Glyma09G28260,Glyma09G02930,Glyma19G38970,Glyma20G27800,Glyma17G34060,Glyma15G37290,Glyma09G37580,Glyma20G35970,Glyma12G03140,Glyma03G04780,Glyma01G39380,Glyma10G38810,Glyma18G45200,Glyma18G53590,Glyma17G16070,Glyma02G46070,Glyma01G00960,Glyma07G30260,Glyma09G40150,Glyma08G06730,Glyma08G08550,Glyma18G08220,Glyma02G46670,Glyma14G05060,Glyma02G03760,Glyma08G06520,Glyma16G03780,Glyma09G38730,Glyma16G07060,Glyma03G29230,Glyma07G39300,Glyma05G02470,Glyma18G38470,Glyma06G40780,Glyma10G32800,Glyma04G27730,Glyma06G47870,Glyma11G19880,Glyma18G47590,Glyma07G04560,Glyma06G40110,Glyma06G08880,Glyma19G44690,Glyma13G02620,Glyma04G06610,Glyma18G50650,Glyma09G30070,Glyma10G08040,Glyma06G45340,Glyma20G30100,Glyma18G49060,Glyma11G04350,Glyma08G18760,Glyma14G03040,Glyma16G33950,Glyma01G34780,Glyma18G47140,Glyma06G18700,Glyma20G27720,Glyma17G01310,Glyma13G42950,Glyma18G50440,Glyma06G40710,Glyma13G36900,Glyma06G40920,Gly

					ma05G23260,Glyma18G46930,Glyma19G38110,Glyma13G10530,Glyma05G01460,Glyma14G36960,Glyma18G51960,Glyma13G10040,Glyma17G04470,Glyma15G17430,Glyma09G13540,Glyma06G40740,Glyma15G42030,Glyma06G02010,Glyma09G26710,Glyma15G14030,Glyma09G05710,Glyma16G10080,Glyma07G31140,Glyma15G00670,Glyma17G09120,Glyma03G00540,Glyma18G04440,Glyma06G40980,Glyma03G05400,Glyma18G08870,Glyma07G12130,Glyma02G48100,Glyma12G34890,Glyma15G00340,Glyma10G32990,Glyma12G33340,Glyma06G40350,Glyma15G00950,Glyma18G10610,Glyma07G10460,Glyma14G29130,Glyma08G15360,Glyma15G37140,Glyma11G27200,Glyma13G10200,Glyma19G35270,Glyma11G05140,Glyma06G40670,Glyma04G01110,Glyma09G40650,Glyma18G51950,Glyma05G03440,Glyma15G04850,Glyma16G32710,Glyma16G10760,Glyma19G00650,Glyma18G42700,Glyma07G09530,Glyma04G34140,Glyma10G35610,Glyma10G02020,Glyma06G05910,Glyma03G32160,Glyma13G23610,Glyma13G26250,Glyma05G30030,Glyma13G00370,Glyma19G43290,Glyma03G37760,Glyma20G27660,Glyma16G27030,Glyma08G05120,Glyma17G30980,Glyma08G13060,Glyma17G34170,Glyma15G14210,Glyma12G17450,Glyma12G17360,Glyma19G34250,Glyma04G39670,Glyma03G42000,Glyma08G09540,Glyma10G04480,Glyma13G06490,Glyma11G34490,Glyma02G14310,Glyma20G27510,Glyma05G30260,Glyma10G35310,Glyma05G31810,Glyma08G23150,Glyma09G37650,Glyma07G02940,Glyma14G38650,Glyma18G10470,Glyma12G36440,Glyma15G04280,Glyma08G08000,Glyma15G04350,Glyma19G04140,Glyma09G08180,Glyma08G05720,Glyma08G43170
GO:003824	catalytic activity	906/1695	12864/27682	1.68E-09	Glyma01G37920,Glyma19G41590,Glyma17G09860,Glyma17G08610,Glyma04G38360,Glyma08G06570,Glyma11G10320,Glyma14G37070,Glyma16G24640,Glyma10G41590,Glyma14G04850,Glyma02G03070,Glyma17G04800,Glyma06G16700,Glyma11G15520,Glyma09G02490,Glyma06G08730,Glyma09G32180,Glyma13G26260,Glyma12G08900,Glyma02G08970,Glyma0028S00210,Glyma18G10220,Glyma18G49000,Glyma06G18970,Glyma11G08560,Glyma11G13480,Glyma12G14230,Glyma20G00270,Glyma01G27810,Glyma17G08540,Glyma15G09700,Glyma17G01730,Glyma17G11620,Glyma05G35930,Glyma06G39750,Glyma19G31720,Glyma14G36420,Glyma13G23840,Glyma18G49880,Glyma04G04380,Glyma05G25550,Glyma08G03240,Glyma18G07080,Glyma13G21820,Glyma14G09510,Glyma07G18500,Glyma05G33240,Glyma04G05920,Glyma10G36320,Glyma02G47230,Glyma15G05680,Glyma07G15500,Glyma02G43200,Glyma02G12030,Glyma20G06680,Glyma15G36110,Glyma20G23010,Glyma01G03200,Glyma16G04120,Glyma15G10370,Glyma06G43050,Glyma09G23330,Glyma20G03040,Glyma18G03120,Glyma18G50450,Glyma04G07370,Glyma09G28100,Glyma08G21340,Glyma13G29390,Glyma15G23310,Glyma18G19670,Glyma07G31310,Glyma13G27840,Glyma17G13910,Glyma13G36500,Glyma16G17760,Glyma10G42870,Glyma07G07290,Glyma17G09940,Glyma08G19860,Glyma13G28750,Glyma19G43630,Glyma10G39470,Glyma03G00440,Glyma07G01250,Glyma11G04310,Glyma06G02310,Glyma14G05930,Glyma07G37520,Glyma16G32640,Glyma13G40550,Glyma20G33460,Glyma07G09260,Glyma09G08370,Glyma06G21910,Glyma04G03120,Glyma13G38950,Glyma04G16670,Glyma10G28880,Glyma08G22890,Glyma20G34790,Glyma08G11940,Glyma14G11040,Glyma10G29700,Glyma06G43940,Glyma10G33790,Glyma03G33590,Glyma05G03640,Glyma10G38450.2,Glyma11G09070,Glyma17G03650,Glyma14G12880,Glyma12G30410,Glyma01G42770,Glyma05G21420,Glyma03G04740,Glyma19G45280,Glyma03G32530,Glyma09G38820,Glyma19G34960,Glyma02G39190,Glyma11G15530,Glyma16G19340,Glyma03G03590,Glyma01G04800,Glyma05G31450,Glyma13G41800,Glyma15G02430,Glyma06G12160,Glyma09G05550,Glyma12G08200,Glyma09G28260,Glyma19G38970,Glyma06G01460,Glyma11G36410,Glyma09G37580,Glyma08G15610,Glyma15G06390,Glyma07G01230,Glyma06G30860,Glyma07G30260,Glyma02G07450,Glyma08G10660,Glyma09G31250,Glyma02G46670,Glyma14G05060,Glyma07G12060,Glyma16G07060,Glyma07G39870,Glyma03G27150,Glyma17G15350,Glyma18G09380,Glyma08G44690,Glyma10G32800,Glyma03G40450,Glyma06G24130,Glyma07G04560,Glyma14G07940,Glyma13G02620,Glyma19G44690,Glyma13G29410,Glyma15G07040,Glyma06G45340,Glyma10G08040,Glyma11G04350,Glyma09G20260,Glyma18G44940,Glyma06G12150,Glyma09G02180,Glyma13G05640,Glyma11G11500,Glyma04G43480,Glyma19G38110,Glyma02G03090,Glyma14G05300,Glyma14G36960,Glyma12G34500,Glyma15G00450,Glyma17G04470,Glyma15G29980,Glyma13G10040,Glyma15G42030,Glyma09G26710,Glyma15G14030,Glyma09G05710,Glyma01G01630,Glyma03G38620,Glyma15G34720,Glyma18G04440,Glyma03G03360,Glyma07G30750,Glyma07G12130,Glyma07G16020,Glyma02G07770,Glyma04G13670,Glyma06G40670,Glyma09G40650,Glyma19G00230,Glyma10G35610,Glyma10G02020,Glyma06G18150,Glyma13G33560,Glyma06G03160,Glyma08G10950,Glyma08G27000,Glyma13G00370,Glyma19G43290,Glyma09G29460,Glyma14G10820,Glyma08G05120,Glyma03G36090,Glyma13G33730,Glyma18G44010,Glyma02G14310,Glyma11G34490,Glyma13G06490,Glyma11G31410,Glyma05G31810,Glyma09G37650,Glyma12G36440,Glyma16G21370,Glyma08G08000,Glyma18G02160,Glyma03G22640,Glyma20G08780,Glyma08G39340,Glyma04G09210,Glyma11G05960,Glyma17G05250,Glyma12G06320,Glyma19G39680,Glyma12G06530,Glyma09G24590,Glyma01G32360,Glyma09G39660,Glyma01G03410,Glyma19G34580,Glyma12G34330,Glyma10G30060,Glyma15G14020,Glyma07G33200,Glyma13G18690,Glyma18G50510,Glyma07G36440,Glyma13G43090,Glyma20G11610,Glyma09G03190,Glyma13G32900,Glyma04G02160,Glyma12G34390,Glyma12G35390,Glyma11G37170,Glyma17G10420,Glyma02G11110,Glyma03G40860,Glyma07G03260,Glyma15G02780,Glyma19G39580,Glyma19G41210,Glyma07G07110,Glyma18G47380,Glyma04G24380,Glyma10G06090,Glyma15G13380,Glyma07G01890,Glyma19G03620,Glyma07G12010,Glyma09G39200,Glyma19G22790,Glyma16G29380,Glyma15G14080,Glyma20G34690,Glyma13G28860,Glyma08G27130,Glyma05G32070,Glyma07G38560,Glyma05G33830,Glyma08G41510,Glyma17G02150,Glyma06G09770,Glyma13G36730,Glyma13G34290,Glyma15G23150,Glyma11G34210,Glyma17G34410,Glyma04G01440,Glyma16G21050,Glyma11G02040,Glyma05G03320,Glyma10G29970,Glyma20G04810,Glyma09G19730,Glyma09G07590,Glyma20G37930,Glyma11G19170,Glyma13G16540,Glyma10G35220,Glyma06G05770,Glyma10G02920,Glyma06G06080,Glyma19G07410,Glyma05G33270,Glyma20G01060,Glyma08G10900,Glyma04G08620,Glyma02G00340,Glyma12G09220,Glyma11G14390,Glyma02G14450,Glyma10G42470,Glyma17G38210,Glyma13G06620,Glyma15G34810,Glyma07G11880,Glyma08G13040,Glyma16G32700,Glyma10G30360,Glyma13G39530,Glyma07G29630,Glyma19G29570,Glyma10G23520,Glyma05G08770,Glyma12G30780,Glyma13G33620,Glyma17G03250,Glyma12G32660,Glyma16G27250,Glyma09G39390,Glyma09G04710,Glyma05G33790,Glyma03G07540,Glyma10G34970,Glyma07G00340,Glyma05G08730,Glyma16G26400,Glyma08G20650,Glyma17G15320,Glyma18G02520,Glyma10G01820,Glyma03G26540,Glyma09G03400,Glyma18G50540,Glyma13G33320,Glyma14G12420,Glyma08G02980,Glyma02G06700,Glyma02G01770,Glyma07G10430,Glyma02G15150,Glyma01G41150,Glyma17G08000,Glyma02G42420,Glyma14G10790,Glyma10G05260,Glyma16G00480,Glyma19G37860,Glyma16G30130,Glyma09G35140,Glyma16G32830,Glyma07G06840,Glyma14G04000,Glyma16G32480,Glyma03G27360,Glyma06G18160,Glyma07G02990,Glyma20G00790,Glyma18G08940,Glyma20G39110,Glyma20G27800,Glyma17G34060,Glyma14G04890,Glyma02G16860,Glyma18G45200,Glyma13G07550,Glyma20G35420,Glyma06G09490,Glyma07G07320,Glyma14G04700,Glyma15G11570,Glyma03G29230,Glyma17G07280,Glyma07G39300,Glyma05G02470,Glyma18G53100,Glyma14G03140,Glyma07G09030,Glyma11G19880,Glyma14G03030,Glyma04G06610,Glyma09G30070,Glym

a18G49060,Glyma18G45070,Glyma08G18860,Glyma11G07760,Glyma06G47490,Glyma13G42950,Glyma16G26330,Glyma08G20140,Glyma17G36300,
Glyma05G09060,Glyma14G37440,Glyma09G13540,Glyma06G02010,Glyma07G09170,Glyma04G33990,Glyma18G04830,Glyma12G04410,Glyma05G0
5990,Glyma07G31140,Glyma12G36570,Glyma18G08870,Glyma02G48100,Glyma11G11410,Glyma20G16020,Glyma10G32990,Glyma06G40350,Glyma
14G29130,Glyma08G15360,Glyma07G10460,Glyma05G02210,Glyma15G02750,Glyma19G35270,Glyma11G05140,Glyma12G31840,Glyma08G47730,G
lyma19G00650,Glyma12G30660,Glyma10G41050,Glyma07G09530,Glyma18G42700,Glyma02G43440,Glyma11G32610,Glyma08G14900,Glyma06G059
10,Glyma03G32160,Glyma07G15780,Glyma05G00920,Glyma03G37760,Glyma09G36070,Glyma16G27030,Glyma02G03320,Glyma10G31160,Glyma17
G34170,Glyma12G17450,Glyma10G39320,Glyma16G08460,Glyma08G39510.2,Glyma17G34480,Glyma08G09540,Glyma06G15860,Glyma10G04480,Gl
yma03G27470,Glyma15G04350,Glyma19G04140,Glyma13G41790,Glyma08G24510,Glyma04G04510,Glyma07G14010,Glyma18G00700,Glyma08G007
40,Glyma05G32340,Glyma16G09890,Glyma06G07410,Glyma04G37770,Glyma08G28460,Glyma06G47380,Glyma17G02290,Glyma13G25660,Glyma09
G31840,Glyma03G04700,Glyma03G03700,Glyma03G00800,Glyma06G44010,Glyma09G00810,Glyma10G12660,Glyma04G09340,Glyma05G34540,Gly
ma14G34480,Glyma20G16170,Glyma16G28400,Glyma08G26940,Glyma08G22520,Glyma10G35560,Glyma15G06600,Glyma10G36220,Glyma06G4264
0,Glyma05G34180,Glyma17G33190,Glyma09G34110,Glyma14G33400,Glyma14G05020,Glyma06G34940,Glyma13G35790,Glyma06G45590,Glyma18G
48600,Glyma14G33700,Glyma01G36290,Glyma13G21030,Glyma01G07970,Glyma14G40510,Glyma16G04910,Glyma10G36750,Glyma08G46130,Glym
a02G07740,Glyma19G30650,Glyma12G09050,Glyma05G26960,Glyma18G40680,Glyma11G03800,Glyma09G21970,Glyma02G38280,Glyma12G34960,
Glyma09G09390,Glyma17G17780,Glyma11G19120,Glyma18G48520,Glyma06G17550,Glyma06G08480,Glyma16G32650,Glyma11G37910,Glyma11G0
9060,Glyma18G01240,Glyma09G34860,Glyma11G31700,Glyma20G27860,Glyma10G03000,Glyma01G10100,Glyma01G08600,Glyma14G06570,Glyma
15G02520,Glyma04G39120,Glyma15G37670,Glyma12G17340,Glyma12G07200,Glyma04G42830,Glyma14G40380,Glyma18G45440,Glyma08G13150,G
lyma17G33470,Glyma07G00670,Glyma06G20360,Glyma08G19890,Glyma20G38610,Glyma14G06970,Glyma16G02290,Glyma14G12710,Glyma20G294
80,Glyma13G34960,Glyma16G22370,Glyma10G34490,Glyma19G00450,Glyma02G09080,Glyma08G40030,Glyma05G03100,Glyma15G10610,Glyma03
G36310,Glyma09G41690,Glyma02G40850,Glyma16G02340,Glyma17G11760,Glyma04G43190,Glyma13G26680,Glyma19G41050,Glyma06G44970,Gly
ma16G04680,Glyma15G02980,Glyma18G44800,Glyma15G14200,Glyma18G52070,Glyma05G24830,Glyma04G31770,Glyma06G13880,Glyma04G1187
0,Glyma04G42370,Glyma08G44730,Glyma14G00380,Glyma16G29780,Glyma11G18350,Glyma17G10120,Glyma10G03200,Glyma10G43400,Glyma20G
29060,Glyma15G42080,Glyma13G25570,Glyma16G32960,Glyma12G34430,Glyma08G40810,Glyma08G40920,Glyma19G39560,Glyma18G48350,Glym
a09G07040,Glyma16G34290,Glyma12G03140,Glyma01G39380,Glyma10G38810,Glyma02G46070,Glyma08G06730,Glyma08G08550,Glyma03G18430,
Glyma08G06520,Glyma09G38730,Glyma01G01500,Glyma14G39930,Glyma15G18310,Glyma04G27730,Glyma15G21010,Glyma02G36720,Glyma08G0
6330,Glyma18G47590,Glyma06G08880,Glyma06G40110,Glyma02G13630,Glyma18G50650,Glyma02G15290,Glyma09G38460,Glyma05G30230,Glyma
14G03040,Glyma07G08400,Glyma01G34780,Glyma01G39760,Glyma06G18700,Glyma20G27720,Glyma17G01310,Glyma03G10980,Glyma09G07100,G
lyma18G50440,Glyma06G14920,Glyma19G38040,Glyma18G46930,Glyma04G30870,Glyma13G10530,Glyma05G01460,Glyma03G24260,Glyma15G174
30,Glyma07G00350,Glyma13G02420,Glyma09G41570,Glyma10G04530,Glyma05G26940,Glyma08G12070,Glyma11G35100,Glyma15G10870,Glyma19
G07330,Glyma12G33340,Glyma07G29650,Glyma13G32300,Glyma20G33150,Glyma15G41000,Glyma03G03640,Glyma13G10200,Glyma17G11670,Gly
ma04G01110,Glyma15G04850,Glyma16G32710,Glyma16G10760,Glyma09G29900,Glyma04G34140,Glyma05G28150,Glyma04G00580,Glyma20G3622
0,Glyma01G28880,Glyma10G28640,Glyma05G30030,Glyma04G11640,Glyma12G36780,Glyma13G38120,Glyma18G47400,Glyma12G01450,Glyma07G
36500.4,Glyma12G17360,Glyma03G24720,Glyma10G12530,Glyma04G39670,Glyma10G07870,Glyma02G05790,Glyma01G45470,Glyma20G27510,Gly
ma09G06570,Glyma06G48250,Glyma08G23150,Glyma15G06870,Glyma15G05350,Glyma09G08180,Glyma14G03310,Glyma04G09600,Glyma06G1594
0,Glyma05G36580,Glyma20G26160,Glyma14G36660,Glyma20G24570,Glyma13G36600,Glyma13G24260,Glyma15G08040,Glyma07G08120,Glyma09G
30430,Glyma16G08470,Glyma19G05020,Glyma19G31590,Glyma04G09670,Glyma08G43930,Glyma16G08560,Glyma17G34960,Glyma19G31230,Glym
a17G34190,Glyma02G10710,Glyma12G11780,Glyma11G29490,Glyma08G42790,Glyma06G04540,Glyma07G07550,Glyma13G32200,Glyma19G40900,
Glyma20G22780,Glyma13G17440,Glyma12G20860,Glyma02G45800,Glyma13G32770,Glyma10G36440,Glyma17G03300,Glyma08G08500,Glyma07G3
2200,Glyma09G31060,Glyma05G26040,Glyma06G15900,Glyma18G35790,Glyma11G13810,Glyma17G16950,Glyma17G04120,Glyma06G21140,Glyma
15G39150,Glyma09G08550,Glyma0048S00320,Glyma12G01380,Glyma17G29990,Glyma04G03250,Glyma09G41840,Glyma07G32590,Glyma17G01500
,Glyma02G11290,Glyma04G41060,Glyma08G09050,Glyma07G06610,Glyma20G23930,Glyma07G17140,Glyma05G07540,Glyma13G27810,Glyma07G3
2540,Glyma10G07500,Glyma05G27850,Glyma17G12550,Glyma18G43390,Glyma16G24610,Glyma20G39350,Glyma15G20000,Glyma14G37620,Glyma
03G40720,Glyma09G33120,Glyma08G08400,Glyma18G16060,Glyma03G32210,Glyma01G01900.2,Glyma13G17710,Glyma18G15150,Glyma14G08260,
Glyma07G31660,Glyma08G39070,Glyma12G32880,Glyma02G04310,Glyma07G31630,Glyma18G52290,Glyma19G37210,Glyma03G02310,Glyma02G4
8020,Glyma20G34160,Glyma03G30310,Glyma12G04200,Glyma14G01730,Glyma10G39540,Glyma05G24400,Glyma01G44350,Glyma18G11110,Glyma
08G21530,Glyma20G38380,Glyma20G34060,Glyma20G29840,Glyma06G04140,Glyma05G24050,Glyma13G37080,Glyma01G42240,Glyma18G06170,G
lyma02G05210,Glyma06G17270,Glyma01G41600,Glyma16G33590,Glyma14G07750,Glyma08G16680,Glyma09G39800,Glyma20G18980,Glyma19G018
50,Glyma11G02150,Glyma02G07690,Glyma13G43480,Glyma17G09700,Glyma16G29040,Glyma03G10390,Glyma01G43190,Glyma16G28010,Glyma04
G40970,Glyma03G31820,Glyma04G36240,Glyma08G11710,Glyma07G16260,Glyma08G44620,Glyma06G20200,Glyma08G24910,Glyma20G27790,Gly
ma03G08980,Glyma09G02930,Glyma20G38860,Glyma20G35970,Glyma09G02960,Glyma0220S00210,Glyma17G16070,Glyma18G07130,Glyma01G00
960,Glyma09G40150,Glyma09G27030,Glyma06G07380,Glyma09G23150,Glyma10G38370,Glyma02G02580,Glyma12G22230,Glyma20G19210,Glyma1
8G06570,Glyma18G38470,Glyma01G36980,Glyma18G52110,Glyma12G01480,Glyma14G16060,Glyma06G47870,Glyma10G40900,Glyma08G10880,Gl
yma12G02880,Glyma08G41930,Glyma19G43270,Glyma09G38510,Glyma20G30100,Glyma19G39180,Glyma18G47140,Glyma07G31380,Glyma06G409
20,Glyma05G23260,Glyma01G29540,Glyma13G35610,Glyma16G01090,Glyma15G34620,Glyma11G02620,Glyma13G26970,Glyma07G11320,Glyma08
G12750,Glyma13G34810,Glyma15G00670,Glyma17G09120,Glyma03G00540,Glyma12G34890,Glyma15G00340,Glyma07G05820,Glyma07G03660,Gly
ma10G30700,Glyma10G30150,Glyma05G03440,Glyma05G35800,Glyma05G33540,Glyma18G43980,Glyma06G21440,Glyma13G42680,Glyma06G1251
0,Glyma13G23610,Glyma17G05670,Glyma11G38230,Glyma20G27660,Glyma17G30980,Glyma15G14810,Glyma08G13060,Glyma15G14210,Glyma17G
14680,Glyma03G32690,Glyma19G34250,Glyma20G29700,Glyma03G42000,Glyma14G07700,Glyma15G05750,Glyma20G08160,Glyma06G38190,Glym

					a05G30260,Glyma10G35310,Glyma09G36200,Glyma07G02940,Glyma14G38650,Glyma09G28920,Glyma15G04280,Glyma05G27840,Glyma08G05720,Glyma16G24800,Glyma17G07940
GO:005529	sugar binding	30/1695	169/27682	1.30E-07	Glyma13G42680,Glyma06G40920,Glyma11G11500,Glyma04G04510,Glyma07G01250,Glyma09G21970,Glyma17G05250,Glyma13G23610,Glyma12G17340,Glyma13G34520,Glyma08G06520,Glyma07G12060,Glyma15G34810,Glyma06G45590,Glyma12G17450,Glyma12G17360,Glyma03G00540,Glyma02G05790,Glyma06G40110,Glyma07G12010,Glyma02G12030,Glyma06G40350,Glyma02G07770,Glyma09G38460,Glyma15G02750,Glyma13G34540,Glyma06G40670,Glyma11G07760,Glyma02G07740,Glyma08G20650
GO:006915	apoptosis	48/1695	352/27682	1.73E-07	Glyma13G25420,Glyma01G27440,Glyma06G39990,Glyma13G26250,Glyma18G09980,Glyma15G37320,Glyma15G13290,Glyma02G03760,Glyma18G51960,Glyma16G34030,Glyma16G03780,Glyma15G37390,Glyma13G25970,Glyma06G17560,Glyma06G40740,Glyma12G16450,Glyma20G06780,Glyma08G13040,Glyma18G43530,Glyma06G41880,Glyma06G40780,Glyma06G47650,Glyma13G26000,Glyma07G07110,Glyma10G32800,Glyma18G09290,Glyma16G10080,Glyma06G40980,Glyma03G05400,Glyma16G25010,Glyma18G09670,Glyma16G24940,Glyma18G10610,Glyma18G11590,Glyma15G37140,Glyma06G40690,Glyma16G33950,Glyma08G12540,Glyma15G37290,Glyma16G33590,Glyma18G10470,Glyma03G05640,Glyma18G51950,Glyma03G04780,Glyma06G41430,Glyma06G40710,Glyma19G31950,Glyma08G43170
GO:012501	programmed cell death	48/1695	352/27682	1.73E-07	Glyma13G25420,Glyma01G27440,Glyma06G39990,Glyma13G26250,Glyma18G09980,Glyma15G37320,Glyma15G13290,Glyma02G03760,Glyma18G51960,Glyma16G34030,Glyma16G03780,Glyma15G37390,Glyma13G25970,Glyma06G17560,Glyma06G40740,Glyma12G16450,Glyma20G06780,Glyma08G13040,Glyma18G43530,Glyma06G41880,Glyma06G40780,Glyma06G47650,Glyma13G26000,Glyma07G07110,Glyma10G32800,Glyma18G09290,Glyma16G10080,Glyma06G40980,Glyma03G05400,Glyma16G25010,Glyma18G09670,Glyma16G24940,Glyma18G10610,Glyma18G11590,Glyma15G37140,Glyma06G40690,Glyma16G33950,Glyma08G12540,Glyma15G37290,Glyma16G33590,Glyma18G10470,Glyma03G05640,Glyma18G51950,Glyma03G04780,Glyma06G41430,Glyma06G40710,Glyma19G31950,Glyma08G43170
GO:008219	cell death	51/1695	386/27682	1.98E-07	Glyma18G09980,Glyma18G51960,Glyma15G37390,Glyma12G16450,Glyma06G40740,Glyma18G43530,Glyma20G06780,Glyma13G26000,Glyma18G09290,Glyma16G10080,Glyma01G36170,Glyma06G40980,Glyma03G05400,Glyma16G25010,Glyma18G10610,Glyma15G37140,Glyma06G40690,Glyma08G12540,Glyma15G37290,Glyma18G51950,Glyma03G05640,Glyma03G04780,Glyma03G22960,Glyma13G25420,Glyma01G27440,Glyma06G39990,Glyma15G37320,Glyma13G26250,Glyma15G13290,Glyma02G03760,Glyma16G34030,Glyma16G03780,Glyma13G25970,Glyma06G17560,Glyma08G13040,Glyma06G41880,Glyma06G47650,Glyma06G40780,Glyma10G32800,Glyma07G07110,Glyma18G09670,Glyma15G08530,Glyma16G24940,Glyma18G11590,Glyma16G33950,Glyma16G33590,Glyma18G10470,Glyma06G41430,Glyma06G40710,Glyma08G43170,Glyma19G31950
GO:016265	death	51/1695	386/27682	1.98E-07	Glyma18G09980,Glyma18G51960,Glyma15G37390,Glyma12G16450,Glyma06G40740,Glyma18G43530,Glyma20G06780,Glyma13G26000,Glyma18G09290,Glyma16G10080,Glyma01G36170,Glyma06G40980,Glyma03G05400,Glyma16G25010,Glyma18G10610,Glyma15G37140,Glyma06G40690,Glyma08G12540,Glyma15G37290,Glyma18G51950,Glyma03G05640,Glyma03G04780,Glyma03G22960,Glyma13G25420,Glyma01G27440,Glyma06G39990,Glyma15G37320,Glyma13G26250,Glyma15G13290,Glyma02G03760,Glyma16G34030,Glyma16G03780,Glyma13G25970,Glyma06G17560,Glyma08G13040,Glyma06G41880,Glyma06G47650,Glyma06G40780,Glyma10G32800,Glyma07G07110,Glyma18G09670,Glyma15G08530,Glyma16G24940,Glyma18G11590,Glyma16G33950,Glyma16G33590,Glyma18G10470,Glyma06G41430,Glyma06G40710,Glyma08G43170,Glyma19G31950
GO:008152	metabolic process	799/1695	11489/27682	7.51E-07	Glyma01G37920,Glyma14G35680,Glyma19G41590,Glyma17G09860,Glyma17G08610,Glyma11G10320,Glyma14G37070,Glyma16G24640,Glyma14G04850,Glyma17G04800,Glyma06G08730,Glyma09G32180,Glyma13G26260,Glyma12G08900,Glyma02G08970,Glyma0028S00210,Glyma18G10220,Glyma06G18970,Glyma11G08560,Glyma11G13480,Glyma12G14230,Glyma17G08540,Glyma15G09700,Glyma17G01730,Glyma17G11620,Glyma06G39750,Glyma14G36420,Glyma13G23840,Glyma18G49880,Glyma05G25550,Glyma08G03240,Glyma13G21820,Glyma07G18500,Glyma05G33240,Glyma04G01610,Glyma10G36320,Glyma02G00490,Glyma15G05680,Glyma02G47230,Glyma07G15500,Glyma02G43200,Glyma04G41630,Glyma02G12030,Glyma15G36110,Glyma20G23010,Glyma07G06620,Glyma01G03200,Glyma16G04120,Glyma06G43050,Glyma09G23330,Glyma20G03040,Glyma18G03120,Glyma18G50450,Glyma09G28100,Glyma08G21340,Glyma20G31530,Glyma15G23310,Glyma18G19670,Glyma07G31310,Glyma13G27840,Glyma13G36500,Glyma05G30650,Glyma16G17760,Glyma03G26080,Glyma10G42870,Glyma07G07290,Glyma17G09940,Glyma08G19860,Glyma10G39470,Glyma06G06380.2,Glyma03G00440,Glyma03G00420,Glyma07G01250,Glyma11G04310,Glyma06G02310,Glyma16G32640,Glyma13G40550,Glyma20G33460,Glyma07G09260,Glyma06G21910,Glyma04G03120,Glyma13G38950,Glyma04G36150,Glyma04G16670,Glyma09G08260,Glyma20G34790,Glyma10G38490,Glyma14G11040,Glyma10G29700,Glyma10G33790,Glyma05G03640,Glyma10G38450.2,Glyma11G09070,Glyma17G03650,Glyma14G12880,Glyma12G30410,Glyma01G42770,Glyma05G21420,Glyma03G04740,Glyma19G45280,Glyma09G38820,Glyma02G39190,Glyma11G15530,Glyma03G03590,Glyma01G04800,Glyma05G31450,Glyma13G41800,Glyma15G02430,Glyma06G12160,Glyma09G05550,Glyma12G08200,Glyma09G28260,Glyma11G02190,Glyma06G01460,Glyma09G37580,Glyma08G15610,Glyma15G06390,Glyma06G30860,Glyma07G30260,Glyma02G46670,Glyma14G05060,Glyma07G12060,Glyma16G07060,Glyma07G39870,Glyma03G27150,Glyma03G37490,Glyma18G09380,Glyma08G44690,Glyma07G04560,Glyma14G07940,Glyma15G37240,Glyma13G02620,Glyma19G44690,Glyma13G29410,Glyma06G45340,Glyma10G08040,Glyma08G18760,Glyma09G20260,Glyma18G44940,Glyma06G12150,Glyma09G02180,Glyma13G05640,Glyma11G11500,Glyma04G43480,Glyma14G05300,Glyma14G36960,Glyma15G00450,Glyma13G25110,Glyma17G04470,Glyma15G29980,Glyma13G10040,Glyma09G26710,Glyma14G08920,Glyma15G14030,Glyma09G05710,Glyma01G01630,Glyma03G38620,Glyma15G34720,Glyma18G04440,Glyma07G12130,Glyma10G43690,Glyma04G40150,Glyma02G07770,Glyma04G13670,Glyma04G34770,Glyma06G40670,Glyma09G40650,Glyma19G00230,Glyma10G35610,Glyma14G03380,Glyma06G18150,Glyma13G33560,Glyma06G03160,Glyma15

G06040,Glyma08G10950,Glyma08G27000,Glyma13G00370,Glyma19G43290,Glyma09G29460,Glyma14G10820,Glyma03G36090,Glyma13G33730,Gly
ma18G44010,Glyma02G14310,Glyma11G34490,Glyma13G06490,Glyma11G31410,Glyma09G37650,Glyma12G36440,Glyma18G11010,Glyma16G2137
0,Glyma08G08000,Glyma10G29200,Glyma18G02160,Glyma03G22640,Glyma20G08780,Glyma08G39340,Glyma04G09210,Glyma11G05960,Glyma17G
05250,Glyma02G43080,Glyma12G06320,Glyma19G39680,Glyma12G06530,Glyma09G24590,Glyma01G32360,Glyma09G39660,Glyma01G03410,Glym
a19G34580,Glyma15G14020,Glyma07G33200,Glyma13G18690,Glyma18G50510,Glyma13G43090,Glyma20G11610,Glyma09G03190,Glyma13G32900,
Glyma04G02160,Glyma12G34390,Glyma12G35390,Glyma11G20180,Glyma09G00300,Glyma11G37170,Glyma17G10420,Glyma02G11110,Glyma03G4
0860,Glyma19G41210,Glyma04G24380,Glyma10G06090,Glyma07G01890,Glyma19G03620,Glyma07G12010,Glyma09G39200,Glyma16G29380,Glyma
15G14080,Glyma20G34690,Glyma05G32070,Glyma06G42800,Glyma06G09770,Glyma14G05050,Glyma13G36730,Glyma13G34290,Glyma15G23150,G
lyma11G34210,Glyma04G01440,Glyma10G29970,Glyma09G19730,Glyma09G07590,Glyma11G19170,Glyma13G16540,Glyma19G38720,Glyma10G352
20,Glyma06G05770,Glyma06G06080,Glyma19G07410,Glyma05G33270,Glyma20G01060,Glyma08G10900,Glyma04G08620,Glyma12G09220,Glyma12
G10580,Glyma11G14390,Glyma02G14450,Glyma20G23080,Glyma10G42470,Glyma17G38210,Glyma13G06620,Glyma15G34810,Glyma08G13040,Gly
ma16G32700,Glyma10G30360,Glyma01G01750,Glyma07G29630,Glyma10G40910,Glyma10G23520,Glyma05G08770,Glyma12G30780,Glyma13G3362
0,Glyma16G27250,Glyma09G39390,Glyma09G04710,Glyma05G33790,Glyma03G07540,Glyma11G10100,Glyma10G34970,Glyma07G00340,Glyma05G
08730,Glyma08G20650,Glyma17G15320,Glyma18G02520,Glyma10G01820,Glyma03G26540,Glyma09G03400,Glyma18G50540,Glyma13G33320,Glym
a14G12420,Glyma08G02980,Glyma16G25600,Glyma02G06700,Glyma02G01770,Glyma02G15150,Glyma01G41150,Glyma17G08000,Glyma07G00440,
Glyma02G42420,Glyma14G10790,Glyma10G05260,Glyma16G00480,Glyma16G30130,Glyma09G35140,Glyma16G32830,Glyma16G32480,Glyma03G2
7360,Glyma06G18160,Glyma07G02990,Glyma18G08940,Glyma20G39110,Glyma20G27800,Glyma14G04890,Glyma18G45200,Glyma18G08220,Glyma
20G35420,Glyma06G09490,Glyma07G07320,Glyma14G04700,Glyma15G11570,Glyma09G03660,Glyma07G39300,Glyma17G07280,Glyma05G02470,G
lyma14G03140,Glyma15G42640,Glyma07G09030,Glyma11G19880,Glyma15G14550,Glyma14G03030,Glyma04G06610,Glyma09G30070,Glyma18G490
60,Glyma18G45070,Glyma08G18860,Glyma11G07760,Glyma19G37820,Glyma13G42950,Glyma16G26330,Glyma05G09060,Glyma14G37440,Glyma09
G13540,Glyma06G02010,Glyma07G09170,Glyma04G33990,Glyma05G05990,Glyma07G31140,Glyma12G36570,Glyma18G04130,Glyma17G01570,Gly
ma02G48100,Glyma11G11410,Glyma08G22690,Glyma10G32990,Glyma06G40350,Glyma08G15360,Glyma14G29130,Glyma07G10460,Glyma15G0275
0,Glyma11G05140,Glyma08G47730,Glyma12G31840,Glyma19G00650,Glyma12G30660,Glyma18G42700,Glyma02G43440,Glyma11G32610,Glyma08G
14900,Glyma03G32160,Glyma10G12350,Glyma10G34020,Glyma03G37760,Glyma09G36070,Glyma02G03320,Glyma11G04560,Glyma10G31160,Glym
a17G34170,Glyma12G17450,Glyma10G39320,Glyma16G08460,Glyma17G34480,Glyma08G09540,Glyma06G15860,Glyma10G04480,Glyma03G27470,
Glyma15G04350,Glyma19G04140,Glyma13G41790,Glyma08G24510,Glyma04G04510,Glyma07G14010,Glyma08G00740,Glyma05G32340,Glyma16G0
9890,Glyma04G37770,Glyma08G28460,Glyma17G02290,Glyma09G31840,Glyma03G04700,Glyma03G03700,Glyma03G00800,Glyma09G00810,Glyma
04G09340,Glyma14G34480,Glyma16G28400,Glyma08G26940,Glyma08G22520,Glyma10G35560,Glyma10G36220,Glyma06G42640,Glyma17G33190,G
lyma09G34110,Glyma14G33400,Glyma14G05020,Glyma06G34940,Glyma18G32680,Glyma12G05460.3,Glyma06G45590,Glyma18G48600,Glyma14G3
3700,Glyma13G21030,Glyma01G07970,Glyma16G04910,Glyma17G09690,Glyma11G34170,Glyma08G10910,Glyma02G07740,Glyma15G40830,Glyma
19G30650,Glyma14G09950,Glyma12G09050,Glyma05G26960,Glyma18G40680,Glyma11G03800,Glyma09G21970,Glyma02G38280,Glyma08G01180,G
lyma09G09390,Glyma20G33200,Glyma17G17780,Glyma18G48520,Glyma06G17550,Glyma20G34340,Glyma06G08480,Glyma11G09060,Glyma18G012
40,Glyma09G34860,Glyma10G03000,Glyma10G28890,Glyma01G10100,Glyma01G08600,Glyma14G06570,Glyma15G02520,Glyma04G39120,Glyma11
G04020,Glyma15G37670,Glyma12G17340,Glyma12G07200,Glyma04G42830,Glyma08G13150,Glyma11G13460,Glyma17G33470,Glyma07G00670,Gly
ma15G19810,Glyma14G06970,Glyma16G02290,Glyma14G12710,Glyma20G29480,Glyma17G10710,Glyma13G34960,Glyma16G22370,Glyma10G3449
0,Glyma19G00450,Glyma02G09080,Glyma08G40030,Glyma05G03100,Glyma07G31350,Glyma15G10610,Glyma09G41690,Glyma02G40850,Glyma16G
02340,Glyma17G11760,Glyma04G43190,Glyma06G11940,Glyma13G26680,Glyma19G41050,Glyma06G44970,Glyma16G04680,Glyma18G44800,Glym
a19G27420,Glyma15G14200,Glyma02G13540,Glyma18G52070,Glyma05G24830,Glyma16G05410,Glyma06G13880,Glyma04G11870,Glyma04G42370,
Glyma08G44730,Glyma14G00380,Glyma16G29780,Glyma11G18350,Glyma17G10120,Glyma10G03200,Glyma10G43400,Glyma15G42080,Glyma18G1
4560,Glyma16G32960,Glyma12G34430,Glyma08G40810,Glyma08G40920,Glyma18G48350,Glyma16G34290,Glyma12G03140,Glyma01G39380,Glyma
10G38810,Glyma02G46070,Glyma08G06730,Glyma08G08550,Glyma03G18430,Glyma08G06520,Glyma01G37090,Glyma14G22170,Glyma01G01500,G
lyma14G39930,Glyma04G27730,Glyma15G21010,Glyma02G36720,Glyma08G06330,Glyma18G47590,Glyma06G08880,Glyma06G40110,Glyma02G136
30,Glyma18G50650,Glyma02G15290,Glyma09G38460,Glyma05G30230,Glyma14G03040,Glyma01G34780,Glyma01G39760,Glyma20G27720,Glyma03
G10980,Glyma09G07100,Glyma18G50440,Glyma06G14920,Glyma17G11130,Glyma10G33310,Glyma04G30870,Glyma05G01460,Glyma03G24260,Gly
ma15G17430,Glyma13G02420,Glyma09G41570,Glyma10G04530,Glyma11G35100,Glyma08G12070,Glyma12G36820,Glyma19G07330,Glyma19G2917
0,Glyma07G29650,Glyma12G33340,Glyma15G00950,Glyma03G03640,Glyma13G10200,Glyma15G04850,Glyma16G32710,Glyma16G10760,Glyma09G
29900,Glyma19G30630,Glyma05G28150,Glyma04G00580,Glyma20G36220,Glyma01G28880,Glyma05G30030,Glyma04G11640,Glyma12G36780,Glym
a13G38120,Glyma07G36500.4,Glyma12G17360,Glyma03G24720,Glyma10G12530,Glyma10G07870,Glyma17G10370,Glyma02G05790,Glyma01G4547
0,Glyma20G27510,Glyma09G06570,Glyma06G48250,Glyma08G23150,Glyma15G06870,Glyma15G05350,Glyma09G08180,Glyma11G02560,Glyma14G
03310,Glyma04G09600,Glyma06G15940,Glyma05G36580,Glyma14G36660,Glyma13G36600,Glyma15G08040,Glyma09G30430,Glyma16G08470,Glym
a19G05020,Glyma19G31590,Glyma04G09670,Glyma08G43930,Glyma16G08560,Glyma17G34960,Glyma19G31230,Glyma17G34190,Glyma07G10090,
Glyma18G01960,Glyma02G10710,Glyma14G40460,Glyma11G29490,Glyma08G42790,Glyma06G04540,Glyma12G20860,Glyma02G45800,Glyma13G3
2770,Glyma17G03300,Glyma08G08500,Glyma07G32200,Glyma05G26040,Glyma09G31060,Glyma11G13810,Glyma17G04120,Glyma17G16950,Glyma
15G39150,Glyma06G21140,Glyma09G08550,Glyma0048S00320,Glyma12G01380,Glyma17G29990,Glyma04G03250,Glyma17G14760,Glyma07G32590
,Glyma17G01500,Glyma02G11290,Glyma16G28070,Glyma07G06610,Glyma07G17140,Glyma20G23930,Glyma12G37040,Glyma09G24410,Glyma05G0
7540,Glyma13G27810,Glyma13G18510,Glyma10G07500,Glyma16G24610,Glyma18G43390,Glyma03G40720,Glyma09G33120,Glyma18G16060,Glyma
03G32210,Glyma09G28040,Glyma20G33570,Glyma20G38370,Glyma13G17710,Glyma08G16150,Glyma07G31660,Glyma14G08260,Glyma18G15150,G
lyma12G32880,Glyma08G39070,Glyma06G19310,Glyma02G04310,Glyma07G31630,Glyma18G52290,Glyma19G37210,Glyma03G02310,Glyma02G480

					20,Glyma20G34160,Glyma14G01730,Glyma12G04200,Glyma10G39540,Glyma01G44350,Glyma18G11110,Glyma08G21530,Glyma20G34060,Glyma06G04140,Glyma13G37080,Glyma18G06170,Glyma12G30010,Glyma02G05210,Glyma06G17270,Glyma17G02080,Glyma01G41600,Glyma09G39800,Glyma05G00310,Glyma14G07750,Glyma20G18980,Glyma19G01850,Glyma11G02150,Glyma10G06500,Glyma02G09370,Glyma13G43480,Glyma17G09700,Glyma05G37970,Glyma01G43190,Glyma04G40970,Glyma10G41990,Glyma03G31820,Glyma09G29980,Glyma07G16260,Glyma08G44620,Glyma06G20200,Glyma20G27790,Glyma09G00280,Glyma03G08980,Glyma07G09820,Glyma14G01440,Glyma09G02930,Glyma20G38860,Glyma09G02960,Glyma20G35970,Glyma0220S00210,Glyma18G53590,Glyma17G16070,Glyma01G00960,Glyma09G40150,Glyma09G27030,Glyma06G07380,Glyma09G23150,Glyma10G38370,Glyma02G02580,Glyma18G14770,Glyma09G00580,Glyma18G38470,Glyma18G06570,Glyma01G36980,Glyma18G52110,Glyma12G01480,Glyma06G47870,Glyma10G40900,Glyma08G10880,Glyma12G02880,Glyma05G36200,Glyma19G43270,Glyma20G30100,Glyma02G44040,Glyma18G47140,Glyma07G31380,Glyma06G40920,Glyma05G23260,Glyma07G39980,Glyma06G11820,Glyma16G01090,Glyma15G34620,Glyma18G01530,Glyma11G02620,Glyma05G01510,Glyma13G26970,Glyma07G11320,Glyma08G12750,Glyma13G34810,Glyma15G00670,Glyma17G09120,Glyma03G00540,Glyma12G34890,Glyma15G00340,Glyma07G05820,Glyma05G03440,Glyma05G35800,Glyma05G33540,Glyma18G43980,Glyma06G21440,Glyma13G42680,Glyma06G12510,Glyma13G23610,Glyma17G05670,Glyma11G38230,Glyma20G27660,Glyma15G14810,Glyma08G13060,Glyma15G14210,Glyma17G14680,Glyma03G32690,Glyma19G34250,Glyma03G42000,Glyma14G07700,Glyma15G05750,Glyma05G03930,Glyma20G08160,Glyma06G38190,Glyma05G30260,Glyma07G02940,Glyma14G38650,Glyma09G28920,Glyma15G04280,Glyma05G27840,Glyma08G05720,Glyma17G07940
GO:030246	carbohydrate binding	38/1695	271/27682	1.62E-06	Glyma13G42680,Glyma06G40920,Glyma11G11500,Glyma04G04510,Glyma06G09770,Glyma07G01250,Glyma15G37670,Glyma09G21970,Glyma17G05250,Glyma13G23610,Glyma12G17340,Glyma13G34520,Glyma08G06520,Glyma07G12060,Glyma15G34810,Glyma06G45590,Glyma01G36980,Glyma12G17450,Glyma04G09670,Glyma12G17360,Glyma03G00540,Glyma02G05790,Glyma06G40110,Glyma07G12010,Glyma01G02560,Glyma02G12030,Glyma03G08980,Glyma06G40350,Glyma02G07770,Glyma09G38460,Glyma06G17270,Glyma15G02750,Glyma13G34540,Glyma12G34140,Glyma06G40670,Glyma11G07760,Glyma02G07740,Glyma08G20650
GO:009987	cellular process	696/1695	9950/27682	3.92E-06	Glyma13G41790,Glyma01G37920,Glyma14G35680,Glyma19G41590,Glyma04G04510,Glyma10G34000,Glyma07G14010,Glyma11G07090,Glyma20G24040,Glyma18G00700,Glyma05G32340,Glyma16G09890,Glyma11G07050,Glyma06G07410,Glyma08G28460,Glyma04G37770,Glyma18G43530,Glyma06G47380,Glyma13G26000,Glyma06G15970,Glyma11G15520,Glyma07G17380,Glyma06G07760,Glyma13G26260,Glyma12G08900,Glyma11G11970,Glyma03G00800,Glyma13G43670,Glyma12G14230,Glyma16G01660,Glyma17G01730,Glyma17G11620,Glyma13G25420,Glyma10G42490,Glyma10G35560,Glyma08G22520,Glyma20G24710,Glyma08G03660,Glyma06G39750,Glyma14G36420,Glyma13G23840,Glyma10G36220,Glyma18G49880,Glyma04G04380,Glyma05G25550,Glyma08G03240,Glyma14G33400,Glyma14G05020,Glyma11G35820,Glyma06G17560,Glyma0021S00410,Glyma06G34940,Glyma13G21820,Glyma12G05460.3,Glyma06G41880,Glyma18G32680,Glyma06G45590,Glyma18G48600,Glyma09G05580,Glyma07G18500,Glyma05G33240,Glyma14G33700,Glyma20G33590,Glyma06G10060,Glyma04G01610,Glyma01G07970,Glyma13G21030,Glyma15G11650,Glyma10G36320,Glyma12G07120,Glyma02G00490,Glyma10G29000,Glyma02G47230,Glyma15G05680,Glyma18G09670,Glyma04G41630,Glyma16G24940,Glyma02G12030,Glyma15G36110,Glyma07G06620,Glyma16G04120,Glyma18G11590,Glyma18G10990,Glyma15G10370,Glyma06G43050,Glyma18G03120,Glyma20G03040,Glyma17G09690,Glyma08G46130,Glyma09G33170,Glyma08G10910,Glyma11G34170,Glyma18G50450,Glyma19G30650,Glyma04G07370,Glyma19G29860,Glyma06G41430,Glyma09G28100,Glyma14G09950,Glyma05G26960,Glyma12G09050,Glyma11G03800,Glyma18G40680,Glyma20G31530,Glyma12G04330,Glyma02G38280,Glyma08G01180,Glyma18G48210,Glyma05G30650,Glyma16G17760,Glyma17G17780,Glyma03G26080,Glyma15G37390,Glyma13G02910,Glyma17G09940,Glyma13G35080,Glyma06G17550,Glyma08G19860,Glyma06G08480,Glyma13G28750,Glyma11G09060,Glyma18G02960,Glyma09G34860,Glyma13G32670,Glyma08G12540,Glyma10G28890,Glyma09G07900,Glyma01G10100,Glyma14G06570,Glyma06G06380.2,Glyma15G02520,Glyma03G00420,Glyma11G04020,Glyma04G39120,Glyma11G04310,Glyma11G00440,Glyma02G47850.2,Glyma15G37320,Glyma16G32640,Glyma12G17340,Glyma13G40550,Glyma04G42830,Glyma05G08230,Glyma07G09260,Glyma14G40380,Glyma13G38950,Glyma04G36150,Glyma08G13150,Glyma17G33470,Glyma11G13460,Glyma04G16670,Glyma07G00670,Glyma09G08260,Glyma15G19810,Glyma16G02290,Glyma14G12710,Glyma20G29480,Glyma17G10710,Glyma10G34490,Glyma16G22370,Glyma10G29700,Glyma02G09080,Glyma08G40030,Glyma05G03640,Glyma15G10630,Glyma05G03100,Glyma07G31350,Glyma02G40850,Glyma16G02340,Glyma10G38450.2,Glyma17G11760,Glyma11G09070,Glyma06G11940,Glyma04G43190,Glyma13G26680,Glyma19G31950,Glyma19G41050,Glyma14G12880,Glyma19G27420,Glyma18G44800,Glyma01G42770,Glyma04G14770,Glyma05G21420,Glyma18G52070,Glyma16G05410,Glyma17G34780,Glyma14G00380,Glyma20G33320,Glyma10G03200,Glyma11G15530,Glyma15G42080,Glyma01G04800,Glyma16G32960,Glyma05G31450,Glyma18G14560,Glyma08G40920,Glyma19G43700,Glyma09G05550,Glyma06G12160,Glyma18G48350,Glyma09G28260,Glyma07G11240,Glyma18G07460,Glyma11G02190,Glyma06G01460,Glyma09G37580,Glyma09G07040,Glyma16G34290,Glyma12G03140,Glyma08G15610,Glyma03G04780,Glyma01G39380,Glyma10G38810,Glyma14G34710,Glyma06G30860,Glyma02G46070,Glyma13G37440,Glyma07G30260,Glyma08G06730,Glyma02G46670,Glyma14G05060,Glyma08G06520,Glyma16G03780,Glyma16G04050,Glyma16G07060,Glyma01G37090,Glyma07G32720,Glyma14G22170,Glyma03G37490,Glyma05G07650,Glyma10G32800,Glyma04G27730,Glyma15G18310,Glyma02G36720,Glyma08G06330,Glyma18G47590,Glyma07G04560,Glyma14G07940,Glyma06G40110,Glyma12G06920,Glyma06G08880,Glyma15G37240,Glyma19G44690,Glyma13G02620,Glyma13G29410,Glyma18G50650,Glyma10G08040,Glyma08G03320,Glyma08G18760,Glyma05G30230,Glyma14G03040,Glyma16G33950,Glyma01G34780,Glyma20G25660,Glyma09G02180,Glyma09G02280,Glyma20G27720,Glyma13G05640,Glyma18G50440,Glyma09G34750,Glyma19G38040,Glyma17G11130,Glyma19G38110,Glyma05G01460,Glyma14G36960,Glyma06G47470,Glyma18G51960,Glyma13G25110,Glyma03G24260,Glyma13G10040,Glyma15G29980,Glyma17G04470,Glyma15G17430,Glyma14G06340,Glyma13G02420,Glyma10G04530,Glyma09G05710,Glyma16G25540,Glyma03G38620,Glyma11G35100,Glyma12G36820,Glyma19G29170,Glyma18G04440,Glyma06G40980,Glyma03G05400,Glyma12G08840,Glyma07G12130,Glyma10G43690,Glyma12G33340,Glyma15G00950,Glyma04G40150,Glyma04G34770,Glyma15G37140,Glyma13G10200,Glyma06G40670,Glyma16G05850,Glyma04G01110,Glyma03G40290,Glyma09G40650,Glyma18G51950,Glyma15G04850,Glyma16G32710,Glyma16G10760,Glyma09G29900,Glyma02G48150,Glyma10G35610,Glyma09G42010,Glyma19G30630,Glyma10G02020,Glyma05G28150,Glyma14G03380,Glyma04G00580,Glyma07G06310,Glyma06G10440,Glyma13G26250,Glyma13G33560,Glyma18G08030,Glyma15G06040,Glyma05G30030,Glyma13G00370,Glyma19G00610,Glyma19G43290,Glyma14G10820,Glyma19G42900,Glyma12G17360,Glyma03G36090,Glyma10G12530,Glyma13G33730,Glyma17G10370,Glyma15G08530,G

					lyma13G06490,Glyma11G34490,Glyma02G14310,Glyma20G27510,Glyma14G37890,Glyma11G31410,Glyma11G32970,Glyma05G31810,Glyma08G23150,Glyma09G37650,Glyma18G11010,Glyma12G36440,Glyma15G06870,Glyma08G08000,Glyma10G29200,Glyma18G02160,Glyma11G02560,Glyma20G08780,Glyma09G08180,Glyma14G03310,Glyma10G30020,Glyma04G09210,Glyma05G36580,Glyma08G21720,Glyma14G36660,Glyma02G43080,Glyma13G36600,Glyma19G39680,Glyma02G04500,Glyma01G32360,Glyma15G08040,Glyma01G03410,Glyma19G34580,Glyma09G30430,Glyma12G34330,Glyma10G30060,Glyma19G05020,Glyma07G33200,Glyma18G50510,Glyma13G18690,Glyma16G08560,Glyma16G25010,Glyma19G31230,Glyma17G34190,Glyma01G40080,Glyma07G10090,Glyma13G05430,Glyma18G01960,Glyma11G09770,Glyma06G40690,Glyma02G10710,Glyma14G40460,Glyma09G03190,Glyma12G34390,Glyma12G35390,Glyma11G29490,Glyma11G20180,Glyma09G00300,Glyma11G37170,Glyma06G39990,Glyma20G36390,Glyma13G17440,Glyma15G25450,Glyma12G20860,Glyma02G45800,Glyma16G34030,Glyma17G10420,Glyma02G11110,Glyma07G32200,Glyma05G26040,Glyma19G41210,Glyma07G07110,Glyma07G04740,Glyma07G00840,Glyma10G06090,Glyma06G21140,Glyma07G01890,Glyma09G08550,Glyma0048S00320,Glyma12G01380,Glyma17G29990,Glyma08G14400,Glyma04G08600,Glyma20G34690,Glyma16G28070,Glyma05G32070,Glyma11G14960,Glyma07G06610,Glyma07G17140,Glyma09G24410,Glyma12G37040,Glyma05G33830,Glyma11G01800,Glyma13G18510,Glyma13G27810,Glyma02G04490,Glyma06G42800,Glyma14G05050,Glyma10G07500,Glyma13G36730,Glyma13G34290,Glyma18G43390,Glyma15G23150,Glyma11G01370,Glyma11G25250,Glyma11G34210,Glyma04G01440,Glyma14G37620,Glyma03G40720,Glyma09G33120,Glyma18G09290,Glyma09G19730,Glyma14G04930,Glyma09G07590,Glyma05G35710,Glyma18G16060,Glyma03G32210,Glyma13G16540,Glyma10G35220,Glyma20G38370,Glyma08G16150,Glyma19G07410,Glyma18G15150,Glyma02G42270,Glyma05G33270,Glyma08G39070,Glyma08G10900,Glyma12G32880,Glyma07G11590,Glyma06G19310,Glyma03G05640,Glyma02G04310,Glyma07G31630,Glyma02G45500,Glyma03G22960,Glyma03G02310,Glyma01G27440,Glyma20G34160,Glyma03G30310,Glyma12G09220,Glyma06G04790,Glyma14G01730,Glyma15G13290,Glyma20G23080,Glyma13G06620,Glyma17G38210,Glyma15G34810,Glyma13G25970,Glyma08G13040,Glyma16G32700,Glyma06G47650,Glyma08G21530,Glyma20G38380,Glyma01G01750,Glyma20G34060,Glyma05G34160,Glyma07G29630,Glyma20G29840,Glyma08G40980,Glyma08G42880,Glyma05G08770,Glyma13G37080,Glyma01G42240,Glyma18G06170,Glyma12G30780,Glyma14G02650,Glyma09G39390,Glyma16G27250,Glyma06G17270,Glyma17G02080,Glyma01G41600,Glyma16G33590,Glyma11G10100,Glyma05G00310,Glyma08G16680,Glyma09G39800,Glyma10G34970,Glyma07G00340,Glyma03G32900,Glyma17G15320,Glyma10G01820,Glyma10G06500,Glyma11G02150,Glyma03G26540,Glyma02G09370,Glyma10G41560,Glyma18G50540,Glyma13G33320,Glyma13G43480,Glyma17G09700,Glyma14G12420,Glyma05G37970,Glyma18G09980,Glyma16G25600,Glyma02G06700,Glyma02G01770,Glyma10G41990,Glyma12G16450,Glyma20G06780,Glyma03G31820,Glyma17G08000,Glyma07G00440,Glyma09G29980,Glyma14G10790,Glyma10G05260,Glyma17G26440,Glyma16G00480,Glyma09G35140,Glyma16G32830,Glyma01G36170,Glyma07G16260,Glyma08G44620,Glyma16G32480,Glyma06G20200,Glyma20G27790,Glyma06G18160,Glyma09G00280,Glyma07G02990,Glyma20G00790,Glyma14G01440,Glyma09G02930,Glyma20G27800,Glyma15G37290,Glyma14G04890,Glyma20G38860,Glyma06G47110,Glyma20G35970,Glyma09G02960,Glyma0220S00210,Glyma08G14520,Glyma18G45200,Glyma18G53590,Glyma17G16070,Glyma01G00960,Glyma09G40150,Glyma09G27030,Glyma18G08220,Glyma06G07380,Glyma09G23150,Glyma10G38370,Glyma02G03760,Glyma02G02580,Glyma18G14770,Glyma16G29510,Glyma12G09680,Glyma17G07280,Glyma07G39300,Glyma09G03660,Glyma09G00580,Glyma05G02470,Glyma18G38470,Glyma06G40780,Glyma01G36980,Glyma05G21910,Glyma18G52110,Glyma14G03140,Glyma15G42640,Glyma06G47870,Glyma11G19880,Glyma02G15490,Glyma15G14550,Glyma14G03030,Glyma13G05130,Glyma05G36200,Glyma19G43270,Glyma09G30070,Glyma09G32510,Glyma20G30100,Glyma18G49060,Glyma02G44040,Glyma08G18860,Glyma18G48470,Glyma17G36440,Glyma18G47140,Glyma17G02560,Glyma13G42950,Glyma09G05540,Glyma06G40710,Glyma03G29280,Glyma06G40920,Glyma07G39980,Glyma05G23260,Glyma01G29540,Glyma18G01530,Glyma14G37440,Glyma05G01510,Glyma09G13540,Glyma06G40740,Glyma06G02010,Glyma07G11320,Glyma04G33990,Glyma12G04410,Glyma05G05990,Glyma16G10080,Glyma07G31140,Glyma15G00670,Glyma17G09120,Glyma03G00540,Glyma12G36570,Glyma18G04130,Glyma18G17290,Glyma18G08870,Glyma17G01570,Glyma02G48100,Glyma08G22690,Glyma12G34890,Glyma15G00340,Glyma10G32990,Glyma06G40350,Glyma07G03660,Glyma18G10610,Glyma07G10460,Glyma14G29130,Glyma08G15360,Glyma10G08700,Glyma19G41890,Glyma11G05140,Glyma04G33950,Glyma05G03440,Glyma05G35800,Glyma02G39150,Glyma19G00650,Glyma12G31840,Glyma12G30660,Glyma06G21440,Glyma18G42700,Glyma07G09530,Glyma19G33030,Glyma03G32160,Glyma13G23610,Glyma11G38230,Glyma10G12350,Glyma05G00920,Glyma03G39320,Glyma03G37760,Glyma20G27660,Glyma16G27030,Glyma10G42350,Glyma17G34400,Glyma15G14810,Glyma08G13060,Glyma11G04560,Glyma17G34170,Glyma12G17450,Glyma10G39320,Glyma19G34250,Glyma16G08460,Glyma05G03310,Glyma03G42000,Glyma08G09540,Glyma06G15860,Glyma05G03930,Glyma06G38190,Glyma05G30260,Glyma03G27470,Glyma07G02940,Glyma14G38650,Glyma18G10470,Glyma15G04280,Glyma05G27840,Glyma15G04350,Glyma19G04140,Glyma08G05720,Glyma11G03140,Glyma17G07940,Glyma08G43170
GO:044238	primary metabolic process	587/1695	8248/27682	5.18E-06	Glyma13G41790,Glyma01G37920,Glyma14G35680,Glyma04G04510,Glyma07G14010,Glyma05G32340,Glyma16G09890,Glyma14G37070,Glyma08G28460,Glyma04G37770,Glyma14G04850,Glyma17G04800,Glyma06G08730,Glyma13G26260,Glyma12G08900,Glyma02G08970,Glyma0028S00210,Glyma03G00800,Glyma11G13480,Glyma12G14230,Glyma09G00810,Glyma04G09340,Glyma14G34480,Glyma17G08540,Glyma15G09700,Glyma17G01730,Glyma17G11620,Glyma10G35560,Glyma08G22520,Glyma06G39750,Glyma13G23840,Glyma10G36220,Glyma18G49880,Glyma06G42640,Glyma17G33190,Glyma05G25550,Glyma08G03240,Glyma14G33400,Glyma14G05020,Glyma06G34940,Glyma13G21820,Glyma12G05460.3,Glyma18G32680,Glyma06G45590,Glyma18G48600,Glyma07G18500,Glyma05G33240,Glyma14G33700,Glyma13G21030,Glyma01G07970,Glyma10G36320,Glyma02G00490,Glyma15G05680,Glyma02G47230,Glyma02G43200,Glyma04G41630,Glyma02G12030,Glyma15G36110,Glyma20G23010,Glyma07G06620,Glyma16G04120,Glyma20G03040,Glyma18G03120,Glyma17G09690,Glyma02G07740,Glyma08G10910,Glyma11G34170,Glyma19G30650,Glyma18G50450,Glyma09G28100,Glyma14G09950,Glyma12G09050,Glyma05G26960,Glyma08G21340,Glyma18G40680,Glyma11G03800,Glyma20G31530,Glyma15G23310,Glyma18G19670,Glyma13G27840,Glyma09G21970,Glyma08G01180,Glyma09G09390,Glyma05G30650,Glyma16G17760,Glyma17G17780,Glyma03G26080,Glyma07G07290,Glyma18G48520,Glyma17G09940,Glyma06G17550,Glyma08G19860,Glyma20G34340,Glyma06G08480,Glyma11G09060,Glyma09G34860,Glyma10G28890,Glyma01G10100,Glyma01G08600,Glyma14G06570,Glyma06G06380.2,Glyma15G02520,Glyma03G00440,Glyma03G00420,Glyma07G01250,Glyma11G04020,Glyma04G39120,Glyma11G04310,Glyma15G37670,Glyma16G32640,Glyma12G17340,Glyma13G40550,Glyma04G42830,Glyma07G09260,Glyma06G21910,Glyma04G03120,Glyma13G38950,Glyma08G13150,Glyma04G36150,Glyma17G33470,Glyma11G13460,Glyma04G16670,Glyma07G00670,Glyma14G06970,Glyma16G02290,Glyma14G12710,Glyma20G29480,Glyma17G10710,Glyma16G22370,Glyma10G34490,G

lyma10G29700,Glyma02G09080,Glyma08G40030,Glyma05G03640,Glyma05G03100,Glyma07G31350,Glyma02G40850,Glyma16G02340,Glyma17G117
60,Glyma10G38450.2,Glyma11G09070,Glyma06G11940,Glyma04G43190,Glyma13G26680,Glyma19G41050,Glyma06G44970,Glyma14G12880,Glyma1
6G04680,Glyma19G27420,Glyma18G44800,Glyma12G30410,Glyma01G42770,Glyma05G21420,Glyma05G24830,Glyma16G05410,Glyma14G00380,Gl
yma16G29780,Glyma11G18350,Glyma02G39190,Glyma15G42080,Glyma11G15530,Glyma16G32960,Glyma05G31450,Glyma18G14560,Glyma08G408
10,Glyma13G41800,Glyma08G40920,Glyma15G02430,Glyma06G12160,Glyma09G05550,Glyma12G08200,Glyma18G48350,Glyma09G28260,Glyma11
G02190,Glyma06G01460,Glyma09G37580,Glyma16G34290,Glyma12G03140,Glyma08G15610,Glyma01G39380,Glyma10G38810,Glyma06G30860,Gly
ma02G46070,Glyma07G30260,Glyma08G06730,Glyma02G46670,Glyma14G05060,Glyma08G06520,Glyma07G12060,Glyma16G07060,Glyma01G3709
0,Glyma14G22170,Glyma03G37490,Glyma18G09380,Glyma01G01500,Glyma14G39930,Glyma04G27730,Glyma15G21010,Glyma02G36720,Glyma18G
47590,Glyma07G04560,Glyma06G40110,Glyma06G08880,Glyma15G37240,Glyma13G02620,Glyma13G29410,Glyma02G13630,Glyma18G50650,Glym
a10G08040,Glyma09G38460,Glyma08G18760,Glyma05G30230,Glyma14G03040,Glyma06G12150,Glyma01G34780,Glyma09G02180,Glyma20G27720,
Glyma13G05640,Glyma09G07100,Glyma18G50440,Glyma06G14920,Glyma11G11500,Glyma04G43480,Glyma17G11130,Glyma04G30870,Glyma10G3
3310,Glyma05G01460,Glyma14G05300,Glyma14G36960,Glyma03G24260,Glyma13G25110,Glyma15G29980,Glyma17G04470,Glyma13G10040,Glyma
15G17430,Glyma13G02420,Glyma09G26710,Glyma14G08920,Glyma10G04530,Glyma09G05710,Glyma01G01630,Glyma03G38620,Glyma11G35100,G
lyma19G07330,Glyma12G36820,Glyma19G29170,Glyma18G04440,Glyma07G12130,Glyma12G33340,Glyma15G00950,Glyma02G07770,Glyma04G401
50,Glyma04G34770,Glyma13G10200,Glyma06G40670,Glyma09G40650,Glyma15G04850,Glyma16G32710,Glyma16G10760,Glyma19G00230,Glyma09
G29900,Glyma10G35610,Glyma19G30630,Glyma05G28150,Glyma14G03380,Glyma04G00580,Glyma20G36220,Glyma13G33560,Glyma06G03160,Gly
ma15G06040,Glyma05G30030,Glyma13G00370,Glyma19G43290,Glyma14G10820,Glyma13G38120,Glyma07G36500.4,Glyma12G17360,Glyma03G247
20,Glyma03G36090,Glyma10G12530,Glyma13G33730,Glyma17G10370,Glyma10G07870,Glyma02G05790,Glyma01G45470,Glyma02G14310,Glyma11
G34490,Glyma13G06490,Glyma20G27510,Glyma11G31410,Glyma09G06570,Glyma06G48250,Glyma08G23150,Glyma09G37650,Glyma12G36440,Gly
ma18G11010,Glyma08G08000,Glyma10G29200,Glyma15G05350,Glyma18G02160,Glyma11G02560,Glyma09G08180,Glyma20G08780,Glyma08G3934
0,Glyma14G03310,Glyma04G09210,Glyma06G15940,Glyma05G36580,Glyma14G36660,Glyma17G05250,Glyma13G36600,Glyma02G43080,Glyma19G
39680,Glyma12G06530,Glyma01G32360,Glyma15G08040,Glyma01G03410,Glyma09G30430,Glyma19G34580,Glyma19G31590,Glyma04G09670,Glym
a07G33200,Glyma13G18690,Glyma18G50510,Glyma16G08560,Glyma19G31230,Glyma17G34190,Glyma18G01960,Glyma02G10710,Glyma14G40460,
Glyma09G03190,Glyma11G29490,Glyma12G35390,Glyma08G42790,Glyma06G04540,Glyma11G20180,Glyma09G00300,Glyma11G37170,Glyma12G2
0860,Glyma02G45800,Glyma17G10420,Glyma02G11110,Glyma17G03300,Glyma07G32200,Glyma05G26040,Glyma19G41210,Glyma11G13810,Glyma
04G24380,Glyma17G16950,Glyma17G04120,Glyma10G06090,Glyma06G21140,Glyma07G01890,Glyma09G08550,Glyma0048S00320,Glyma07G12010
,Glyma12G01380,Glyma09G39200,Glyma17G14760,Glyma07G32590,Glyma17G01500,Glyma20G34690,Glyma05G32070,Glyma07G06610,Glyma07G1
7140,Glyma09G24410,Glyma12G37040,Glyma05G07540,Glyma13G18510,Glyma13G27810,Glyma06G42800,Glyma06G09770,Glyma14G05050,Glyma
10G07500,Glyma13G36730,Glyma13G34290,Glyma18G43390,Glyma15G23150,Glyma11G34210,Glyma04G01440,Glyma09G33120,Glyma09G19730,G
lyma09G07590,Glyma18G16060,Glyma13G16540,Glyma09G28040,Glyma10G35220,Glyma20G33570,Glyma13G17710,Glyma06G06080,Glyma08G161
50,Glyma19G07410,Glyma18G15150,Glyma05G33270,Glyma08G39070,Glyma08G10900,Glyma12G32880,Glyma02G04310,Glyma07G31630,Glyma18
G52290,Glyma03G02310,Glyma04G08620,Glyma20G34160,Glyma12G04200,Glyma12G09220,Glyma14G01730,Glyma10G42470,Glyma20G23080,Gly
ma13G06620,Glyma17G38210,Glyma15G34810,Glyma08G13040,Glyma16G32700,Glyma18G11110,Glyma01G01750,Glyma20G34060,Glyma07G2963
0,Glyma10G40910,Glyma10G23520,Glyma13G37080,Glyma18G06170,Glyma12G30780,Glyma02G05210,Glyma09G39390,Glyma16G27250,Glyma06G
17270,Glyma17G02080,Glyma01G41600,Glyma11G10100,Glyma14G07750,Glyma05G00310,Glyma09G39800,Glyma05G08730,Glyma07G00340,Glym
a08G20650,Glyma17G15320,Glyma10G06500,Glyma11G02150,Glyma03G26540,Glyma02G09370,Glyma18G50540,Glyma13G33320,Glyma13G43480,
Glyma17G09700,Glyma14G12420,Glyma16G25600,Glyma02G06700,Glyma02G01770,Glyma10G41990,Glyma01G41150,Glyma03G31820,Glyma17G0
8000,Glyma02G42420,Glyma07G00440,Glyma09G29980,Glyma14G10790,Glyma16G00480,Glyma09G35140,Glyma16G32830,Glyma07G16260,Glyma
08G44620,Glyma16G32480,Glyma06G20200,Glyma20G27790,Glyma03G08980,Glyma09G00280,Glyma14G01440,Glyma09G02930,Glyma20G39110,G
lyma20G27800,Glyma14G04890,Glyma20G38860,Glyma20G35970,Glyma09G02960,Glyma0220S00210,Glyma18G45200,Glyma18G53590,Glyma17G1
6070,Glyma01G00960,Glyma09G40150,Glyma09G27030,Glyma18G08220,Glyma06G07380,Glyma06G09490,Glyma09G23150,Glyma10G38370,Glyma
02G02580,Glyma18G14770,Glyma17G07280,Glyma07G39300,Glyma09G03660,Glyma09G00580,Glyma05G02470,Glyma18G06570,Glyma18G38470,G
lyma01G36980,Glyma18G52110,Glyma14G03140,Glyma12G01480,Glyma15G42640,Glyma06G47870,Glyma11G19880,Glyma15G14550,Glyma08G108
80,Glyma12G02880,Glyma04G06610,Glyma05G36200,Glyma19G43270,Glyma09G30070,Glyma20G30100,Glyma18G49060,Glyma02G44040,Glyma08
G18860,Glyma18G47140,Glyma11G07760,Glyma13G42950,Glyma06G40920,Glyma07G39980,Glyma05G23260,Glyma16G01090,Glyma14G37440,Gly
ma05G01510,Glyma09G13540,Glyma06G02010,Glyma07G11320,Glyma04G33990,Glyma08G12750,Glyma05G05990,Glyma07G31140,Glyma15G0067
0,Glyma17G09120,Glyma03G00540,Glyma12G36570,Glyma18G04130,Glyma17G01570,Glyma11G11410,Glyma02G48100,Glyma12G34890,Glyma15G
00340,Glyma10G32990,Glyma06G40350,Glyma07G10460,Glyma14G29130,Glyma08G15360,Glyma15G02750,Glyma05G03440,Glyma19G00650,Glym
a12G31840,Glyma12G30660,Glyma06G21440,Glyma18G42700,Glyma13G42680,Glyma02G43440,Glyma11G32610,Glyma03G32160,Glyma13G23610,
Glyma17G05670,Glyma11G38230,Glyma10G12350,Glyma10G34020,Glyma03G37760,Glyma20G27660,Glyma02G03320,Glyma15G14810,Glyma08G1
3060,Glyma10G31160,Glyma17G34170,Glyma17G14680,Glyma12G17450,Glyma10G39320,Glyma03G32690,Glyma19G34250,Glyma08G09540,Glyma
06G15860,Glyma14G07700,Glyma05G03930,Glyma06G38190,Glyma05G30260,Glyma03G27470,Glyma07G02940,Glyma14G38650,Glyma09G28920,G
lyma15G04280,Glyma15G04350,Glyma19G04140,Glyma08G05720,Glyma17G07940

GO:016817	hydrolase activity, acting on acid anhydrides	118/1695	1336/27682	3.73E-05	Glyma20G26160,Glyma16G29040,Glyma18G00700,Glyma13G24260,Glyma04G38360,Glyma12G06530,Glyma06G07410,Glyma07G08120,Glyma03G32530,Glyma01G41150,Glyma12G34330,Glyma04G36240,Glyma10G30060,Glyma02G03070,Glyma06G16700,Glyma20G29060,Glyma11G15520,Glyma09G02490,Glyma06G20200,Glyma0028S00210,Glyma09G28260,Glyma09G02930,Glyma19G38970,Glyma17G34060,Glyma05G34540,Glyma17G08540,Glyma20G16170,Glyma07G01230,Glyma07G07550,Glyma19G31720,Glyma13G17440,Glyma04G04380,Glyma05G34180,Glyma05G25550,Glyma17G10420,Glyma09G38730,Glyma03G29230,Glyma15G02780,Glyma18G07080,Glyma19G39580,Glyma13G35790,Glyma06G15900,Glyma10G32800,Glyma07G07110,Glyma04G05920,Glyma15G13380,Glyma01G07970,Glyma06G21140,Glyma04G06610,Glyma20G23010,Glyma15G10370,Glyma11G04350,Glyma04G41060,Glyma08G09050,Glyma08G46130,Glyma06G18700,Glyma17G01310,Glyma04G07370,Glyma05G33830,Glyma08G41510,Glyma18G46930,Glyma19G38110,Glyma13G10530,Glyma05G27850,Glyma05G01460,Glyma17G34410,Glyma15G20000,Glyma11G19120,Glyma07G00350,Glyma16G21050,Glyma15G42030,Glyma09G05710,Glyma12G04410,Glyma15G00670,Glyma11G37910,Glyma20G37930,Glyma18G08870,Glyma09G34860,Glyma15G00340,Glyma12G33340,Glyma19G35270,Glyma04G01110,Glyma05G03440,Glyma16G10760,Glyma07G09530,Glyma04G34140,Glyma10G02020,Glyma11G04310,Glyma06G05910,Glyma03G30310,Glyma09G08370,Glyma06G21910,Glyma07G11880,Glyma16G27030,Glyma08G05120,Glyma14G40380,Glyma17G30980,Glyma18G45440,Glyma20G38380,Glyma06G20360,Glyma20G34060,Glyma20G29840,Glyma10G28880,Glyma20G38610,Glyma19G34250,Glyma04G39670,Glyma01G42240,Glyma02G09080,Glyma03G33590,Glyma10G35310,Glyma08G23150,Glyma05G31810,Glyma16G33590,Glyma07G02940,Glyma03G36310,Glyma14G07750,Glyma08G16680,Glyma09G08180
GO:008150	biological_process	1166/1695	17817/27682	4.13E-05	Glyma01G37920,Glyma14G35680,Glyma19G41590,Glyma17G09860,Glyma17G08610,Glyma05G25270,Glyma08G06570,Glyma11G10320,Glyma07G15260,Glyma14G37070,Glyma04G01760,Glyma16G24640,Glyma18G43530,Glyma14G04850,Glyma17G04800,Glyma17G17770,Glyma08G03560,Glyma13G26000,Glyma06G15970,Glyma11G15520,Glyma18G51080,Glyma06G08730,Glyma06G07760,Glyma09G32180,Glyma13G26260,Glyma12G08900,Glyma02G08970,Glyma0028S00210,Glyma11G11970,Glyma18G10220,Glyma06G18970,Glyma11G08560,Glyma11G13480,Glyma12G14230,Glyma16G01660,Glyma17G08540,Glyma15G09700,Glyma17G01730,Glyma17G11620,Glyma13G25420,Glyma05G35930,Glyma06G39750,Glyma14G36420,Glyma13G23840,Glyma18G49880,Glyma04G04380,Glyma05G25550,Glyma08G03240,Glyma11G35820,Glyma06G17560,Glyma0021S00410,Glyma02G47820,Glyma13G21820,Glyma11G33210,Glyma06G41880,Glyma09G05580,Glyma07G18500,Glyma03G04380,Glyma05G29970,Glyma05G33240,Glyma20G33590,Glyma06G10060,Glyma04G01610,Glyma15G11650,Glyma10G36320,Glyma12G07120,Glyma02G00490,Glyma02G47230,Glyma15G05680,Glyma07G15500,Glyma18G09670,Glyma02G43200,Glyma04G41630,Glyma02G12030,Glyma15G36110,Glyma20G23010,Glyma07G06620,Glyma01G03200,Glyma16G04120,Glyma15G10370,Glyma18G10990,Glyma18G11590,Glyma06G45490,Glyma06G43050,Glyma09G23330,Glyma11G13120,Glyma18G03120,Glyma20G03040,Glyma18G50450,Glyma04G07370,Glyma09G28100,Glyma08G21340,Glyma20G31530,Glyma15G23310,Glyma18G19670,Glyma12G04330,Glyma07G31310,Glyma13G27840,Glyma07G05660,Glyma13G36500,Glyma05G30650,Glyma16G17760,Glyma03G26080,Glyma10G42870,Glyma15G37390,Glyma07G07290,Glyma17G09940,Glyma13G35080,Glyma08G19860,Glyma01G40760,Glyma13G28750,Glyma18G02960,Glyma01G39540,Glyma10G39470,Glyma06G06380.2,Glyma03G00440,Glyma03G00420,Glyma07G01250,Glyma11G04310,Glyma06G02310,Glyma11G00440,Glyma02G47850.2,Glyma15G37320,Glyma16G32640,Glyma13G40550,Glyma20G33460,Glyma07G09260,Glyma06G21910,Glyma04G03120,Glyma03G41800,Glyma13G38950,Glyma04G36150,Glyma04G16670,Glyma09G08260,Glyma10G28880,Glyma20G34790,Glyma10G38490,Glyma14G11040,Glyma10G29700,Glyma10G33790,Glyma05G03640,Glyma10G38450.2,Glyma11G09070,Glyma17G03650,Glyma14G12880,Glyma02G37720,Glyma12G30410,Glyma04G14770,Glyma01G42770,Glyma05G21420,Glyma03G04740,Glyma18G41270,Glyma17G34780,Glyma19G45280,Glyma02G10420,Glyma09G38820,Glyma20G33320,Glyma02G39190,Glyma11G15530,Glyma03G03590,Glyma01G04800,Glyma05G31450,Glyma13G41800,Glyma19G43700,Glyma15G02430,Glyma06G12160,Glyma09G05550,Glyma12G08200,Glyma09G28260,Glyma07G11240,Glyma18G07460,Glyma11G02190,Glyma06G01460,Glyma09G37580,Glyma08G15610,Glyma03G04780,Glyma15G06390,Glyma14G34710,Glyma06G30860,Glyma13G34770,Glyma07G30260,Glyma02G46670,Glyma14G05060,Glyma09G24220,Glyma07G12060,Glyma11G35320,Glyma16G07060,Glyma07G32720,Glyma07G39870,Glyma03G27150,Glyma03G37490,Glyma18G09380,Glyma08G44690,Glyma05G07650,Glyma10G32800,Glyma18G15970,Glyma05G01450,Glyma07G04560,Glyma14G07940,Glyma12G06920,Glyma15G37240,Glyma19G44690,Glyma13G02620,Glyma13G29410,Glyma15G07040,Glyma06G45340,Glyma10G08040,Glyma08G18760,Glyma09G20260,Glyma18G44940,Glyma06G12150,Glyma09G02180,Glyma13G05640,Glyma03G02610,Glyma09G35860,Glyma13G36900,Glyma11G11500,Glyma04G43480,Glyma19G38110,Glyma14G05300,Glyma14G36960,Glyma06G47470,Glyma12G34500,Glyma12G29730,Glyma15G00450,Glyma13G25110,Glyma15G29980,Glyma17G04470,Glyma13G10040,Glyma09G26710,Glyma14G08920,Glyma15G14030,Glyma16G25540,Glyma09G05710,Glyma01G01630,Glyma03G38620,Glyma15G34720,Glyma18G04440,Glyma06G40980,Glyma15G15080,Glyma12G08840,Glyma03G03360,Glyma07G30750,Glyma07G12130,Glyma10G43690,Glyma02G07770,Glyma04G40150,Glyma04G13670,Glyma04G34770,Glyma06G40670,Glyma18G51950,Glyma09G40650,Glyma19G00230,Glyma02G48150,Glyma10G35610,Glyma09G42010,Glyma10G02020,Glyma14G03380,Glyma06G18150,Glyma13G33560,Glyma06G03160,Glyma15G06040,Glyma08G10950,Glyma08G27000,Glyma13G00370,Glyma19G43290,Glyma09G29460,Glyma14G10820,Glyma03G36090,Glyma13G33730,Glyma17G20070,Glyma18G44010,Glyma15G08530,Glyma02G14310,Glyma11G34490,Glyma13G06490,Glyma11G31410,Glyma14G37890,Glyma15G13950,Glyma11G32970,Glyma05G31810,Glyma09G37650,Glyma12G36440,Glyma18G11010,Glyma16G21370,Glyma08G08000,Glyma10G29200,Glyma18G02160,Glyma03G22640,Glyma20G08780,Glyma08G39340,Glyma10G30020,Glyma04G09210,Glyma02G40320,Glyma11G05960,Glyma17G05250,Glyma02G43080,Glyma12G06320,Glyma19G39680,Glyma12G06530,Glyma09G24590,Glyma01G32360,Glyma09G39660,Glyma01G03410,Glyma19G34580,Glyma12G34330,Glyma10G30060,Glyma15G14020,Glyma07G33200,Glyma06G19630,Glyma12G00380,Glyma02G11140,Glyma13G18690,Glyma18G50510,Glyma09G29080,Glyma13G05430,Glyma13G43090,Glyma01G31840,Glyma20G11610,Glyma09G03190,Glyma13G32900,Glyma04G02160,Glyma12G34390,Glyma12G35390,Glyma11G20180,Glyma09G00300,Glyma11G37170,Glyma06G39990,Glyma20G36390,Glyma15G25450,Glyma17G10420,Glyma02G11110,Glyma03G40860,Glyma09G37370,Glyma02G07460,Glyma19G41210,Glyma14G32210,Glyma07G07110,Glyma04G24380,Glyma10G06090,Glyma07G01890,Glyma19G03620,Glyma07G12010,Glyma09G39200,Glyma19G22790,Glyma16G29380,Glyma13G22760,Glyma04G08600,Glyma15G14080,Glyma20G34690,Glyma17G14830,Glyma05G32070,Glyma11G14960,Glyma05G33830,Glyma06G17420,Glyma11G01800,Gly

ma06G42800,Glyma02G04490,Glyma06G09770,Glyma14G05050,Glyma13G36730,Glyma06G15220,Glyma03G38280,Glyma13G34290,Glyma15G2315
0,Glyma02G45150.2,Glyma11G25250,Glyma13G40310,Glyma11G34210,Glyma04G01440,Glyma10G29970,Glyma09G19730,Glyma09G07590,Glyma05
G35710,Glyma01G22910,Glyma11G19170,Glyma13G16540,Glyma19G38720,Glyma10G35220,Glyma06G05770,Glyma06G06080,Glyma19G07410,Gly
ma20G01060,Glyma05G33270,Glyma08G10900,Glyma07G11590,Glyma19G05800,Glyma08G17190,Glyma13G35740,Glyma02G45500,Glyma03G0544
0,Glyma04G08620,Glyma13G43950,Glyma12G10580,Glyma12G09220,Glyma06G04790,Glyma11G14390,Glyma02G14450,Glyma10G42470,Glyma10G
31520,Glyma04G35110,Glyma03G30970,Glyma20G23080,Glyma13G06620,Glyma17G38210,Glyma15G34810,Glyma08G13040,Glyma16G32700,Glym
a12G08030,Glyma06G03950,Glyma10G30360,Glyma01G01750,Glyma07G29630,Glyma10G40910,Glyma10G23520,Glyma08G42880,Glyma05G08770,
Glyma02G13420,Glyma12G30780,Glyma13G33620,Glyma09G39390,Glyma16G27250,Glyma18G45340,Glyma09G04710,Glyma03G07540,Glyma05G3
3790,Glyma11G10100,Glyma10G34970,Glyma05G08730,Glyma07G00340,Glyma08G20650,Glyma03G32900,Glyma18G02520,Glyma17G15320,Glyma
04G35730,Glyma10G01820,Glyma03G26540,Glyma18G50540,Glyma09G03400,Glyma13G33320,Glyma14G12420,Glyma08G02980,Glyma18G09980,G
lyma16G25600,Glyma02G06700,Glyma02G15150,Glyma02G01770,Glyma12G16450,Glyma01G41150,Glyma17G08000,Glyma16G29670,Glyma02G424
20,Glyma07G00440,Glyma14G10790,Glyma10G05260,Glyma17G26440,Glyma16G00480,Glyma16G30130,Glyma09G35140,Glyma16G32830,Glyma16
G32480,Glyma10G13720,Glyma03G27360,Glyma06G18160,Glyma07G02990,Glyma18G08940,Glyma20G00790,Glyma20G39110,Glyma20G27800,Gly
ma14G04890,Glyma06G47110,Glyma08G14520,Glyma18G45200,Glyma17G10950,Glyma20G35420,Glyma18G08220,Glyma07G07320,Glyma06G0949
0,Glyma14G04700,Glyma15G11570,Glyma12G09680,Glyma17G07280,Glyma07G39300,Glyma09G03660,Glyma05G02470,Glyma06G40780,Glyma05G
21910,Glyma14G03140,Glyma15G42640,Glyma07G09030,Glyma11G19880,Glyma15G14550,Glyma14G03030,Glyma13G05130,Glyma04G06610,Glym
a09G30070,Glyma18G49060,Glyma18G45070,Glyma08G18860,Glyma17G36440,Glyma11G07760,Glyma17G02560,Glyma19G37820,Glyma13G42950,
Glyma09G05540,Glyma16G26330,Glyma05G31190,Glyma09G37230,Glyma17G07860,Glyma05G09060,Glyma14G37440,Glyma09G13540,Glyma06G4
0740,Glyma07G09170,Glyma06G02010,Glyma04G33990,Glyma12G04410,Glyma05G05990,Glyma16G10080,Glyma07G31140,Glyma12G36570,Glyma
18G04130,Glyma18G08870,Glyma13G38460,Glyma17G01570,Glyma11G11410,Glyma02G48100,Glyma08G22690,Glyma10G32990,Glyma06G40350,G
lyma07G10460,Glyma14G29130,Glyma08G15360,Glyma15G02750,Glyma11G05140,Glyma19G00650,Glyma12G31840,Glyma08G47730,Glyma15G012
70,Glyma12G30660,Glyma18G42700,Glyma07G09530,Glyma02G43440,Glyma19G33030,Glyma11G32610,Glyma10G38580,Glyma08G14900,Glyma12
G34110,Glyma03G32160,Glyma19G40560,Glyma10G12350,Glyma05G00920,Glyma01G20710,Glyma10G34020,Glyma03G37760,Glyma09G36070,Gly
ma16G27030,Glyma17G34400,Glyma02G03320,Glyma11G04560,Glyma10G31160,Glyma17G34170,Glyma12G17450,Glyma08G45020,Glyma10G3932
0,Glyma16G08460,Glyma08G39510.2,Glyma17G34480,Glyma08G09540,Glyma10G04480,Glyma06G15860,Glyma05G00330,Glyma03G27470,Glyma18
G10470,Glyma15G04350,Glyma19G04140,Glyma13G41790,Glyma04G04510,Glyma08G24510,Glyma10G34000,Glyma07G14010,Glyma11G07090,Gly
ma20G24040,Glyma18G00700,Glyma05G32340,Glyma08G00740,Glyma16G09890,Glyma11G07050,Glyma06G07410,Glyma11G20030,Glyma08G2846
0,Glyma04G37770,Glyma03G37870,Glyma17G02290,Glyma06G47380,Glyma07G17380,Glyma09G31840,Glyma04G37780,Glyma03G04700,Glyma03G
03700,Glyma04G33110,Glyma03G00800,Glyma13G43670,Glyma09G00810,Glyma04G09340,Glyma14G34480,Glyma08G26940,Glyma16G28400,Glym
a10G42490,Glyma10G35560,Glyma08G22520,Glyma20G24710,Glyma08G03660,Glyma10G36220,Glyma06G42640,Glyma17G33190,Glyma09G34110,
Glyma14G33400,Glyma14G05020,Glyma06G34940,Glyma18G06790,Glyma12G05460.3,Glyma18G32680,Glyma06G45590,Glyma18G48600,Glyma09G
40440,Glyma14G33700,Glyma13G21030,Glyma01G07970,Glyma10G29000,Glyma16G24940,Glyma16G04910,Glyma15G05190,Glyma17G09690,Glym
a08G46130,Glyma09G33170,Glyma02G07740,Glyma08G10910,Glyma11G34170,Glyma20G24800,Glyma15G40830,Glyma19G30650,Glyma06G41430,
Glyma19G29860,Glyma14G09950,Glyma14G24590,Glyma12G09050,Glyma05G26960,Glyma18G40680,Glyma11G03800,Glyma17G17120,Glyma02G3
8280,Glyma09G21970,Glyma08G01180,Glyma12G09980,Glyma18G48210,Glyma09G09390,Glyma17G17780,Glyma20G33200,Glyma18G48520,Glyma
13G02910,Glyma06G17550,Glyma20G34340,Glyma06G08480,Glyma18G05020,Glyma11G09060,Glyma18G01240,Glyma03G00280,Glyma09G34860,G
lyma13G32670,Glyma10G03000,Glyma10G28890,Glyma08G12540,Glyma09G07900,Glyma15G23560,Glyma01G10100,Glyma01G08600,Glyma13G235
70,Glyma14G06570,Glyma15G02520,Glyma11G04020,Glyma04G39120,Glyma15G37670,Glyma12G17340,Glyma12G07200,Glyma03G38480,Glyma15
G13640,Glyma04G42830,Glyma05G08230,Glyma14G40380,Glyma08G13150,Glyma17G33470,Glyma11G13460,Glyma07G00670,Glyma03G40770,Gly
ma15G19810,Glyma14G06970,Glyma16G02290,Glyma14G12710,Glyma20G29480,Glyma17G10710,Glyma13G34960,Glyma16G22370,Glyma10G3449
0,Glyma14G11030,Glyma19G00450,Glyma02G09080,Glyma08G13300,Glyma09G00360,Glyma08G40030,Glyma15G10630,Glyma05G03100,Glyma07G
31350,Glyma17G17010,Glyma15G10610,Glyma09G41690,Glyma02G40850,Glyma16G02340,Glyma17G11760,Glyma06G11940,Glyma04G43190,Glym
a13G26680,Glyma19G31950,Glyma19G41050,Glyma06G44970,Glyma16G04680,Glyma19G27420,Glyma18G44800,Glyma15G14200,Glyma15G01150,
Glyma17G04710,Glyma02G13540,Glyma18G52070,Glyma05G24830,Glyma16G05410,Glyma20G33390,Glyma06G13880,Glyma04G11870,Glyma04G4
2370,Glyma08G44730,Glyma14G00380,Glyma16G29780,Glyma11G18350,Glyma17G10120,Glyma10G03200,Glyma10G43400,Glyma15G42080,Glyma
20G36750,Glyma16G32960,Glyma18G14560,Glyma12G34430,Glyma08G40810,Glyma08G40920,Glyma11G06500,Glyma16G06670,Glyma18G48350,G
lyma16G01940,Glyma09G07040,Glyma16G34290,Glyma12G03140,Glyma01G39380,Glyma10G38810,Glyma02G46070,Glyma13G37440,Glyma08G067
30,Glyma08G08550,Glyma03G18430,Glyma11G13960,Glyma08G06520,Glyma16G03780,Glyma16G04050,Glyma01G37090,Glyma14G22170,Glyma01
G01500,Glyma14G39930,Glyma04G27730,Glyma15G18310,Glyma15G21010,Glyma02G36720,Glyma08G06330,Glyma18G47590,Glyma06G40110,Gly
ma06G08880,Glyma02G13630,Glyma18G50650,Glyma02G15290,Glyma08G03320,Glyma09G38460,Glyma05G30230,Glyma14G03040,Glyma16G3395
0,Glyma01G34780,Glyma20G25660,Glyma01G39760,Glyma09G02280,Glyma20G27720,Glyma08G14370,Glyma03G10980,Glyma09G07100,Glyma18G
50440,Glyma06G14920,Glyma09G34750,Glyma19G38040,Glyma05G37270,Glyma18G46930,Glyma17G11130,Glyma04G30870,Glyma10G33310,Glym
a05G01460,Glyma08G10080,Glyma18G51960,Glyma03G24260,Glyma15G17430,Glyma14G06340,Glyma07G00350,Glyma13G02420,Glyma09G41570,
Glyma10G04530,Glyma08G12070,Glyma11G35100,Glyma19G07330,Glyma12G36820,Glyma19G29170,Glyma03G05400,Glyma12G33340,Glyma07G2
9650,Glyma13G32300,Glyma15G00950,Glyma15G37140,Glyma03G03640,Glyma13G10200,Glyma16G05850,Glyma04G01110,Glyma03G40290,Glyma
15G04850,Glyma16G32710,Glyma16G10760,Glyma09G29900,Glyma19G30630,Glyma05G28150,Glyma04G00580,Glyma20G36220,Glyma07G06310,G
lyma01G28880,Glyma06G10440,Glyma13G26250,Glyma18G08030,Glyma05G30030,Glyma19G00610,Glyma11G05720,Glyma04G11640,Glyma09G269
60,Glyma19G42900,Glyma12G36780,Glyma13G38120,Glyma07G36500.4,Glyma12G17360,Glyma03G24720,Glyma10G12530,Glyma17G10370,Glyma1

					0G07870,Glyma02G05790,Glyma01G44150,Glyma01G45470,Glyma20G27510,Glyma06G15840,Glyma09G06570,Glyma06G48250,Glyma08G23150,Glyma17G07010,Glyma12G08640,Glyma15G06870,Glyma15G05350,Glyma11G02560,Glyma09G08180,Glyma14G03310,Glyma05G26640,Glyma04G09600,Glyma06G15940,Glyma05G36580,Glyma08G21720,Glyma14G36660,Glyma13G36600,Glyma15G13190,Glyma10G03170,Glyma02G04500,Glyma15G08040,Glyma09G30430,Glyma16G08470,Glyma19G05020,Glyma19G31590,Glyma04G09670,Glyma08G43930,Glyma16G08560,Glyma16G25010,Glyma09G34400,Glyma17G34960,Glyma19G31230,Glyma09G31650,Glyma17G34190,Glyma01G40080,Glyma07G10090,Glyma18G01960,Glyma11G09770,Glyma06G40690,Glyma02G10710,Glyma14G40460,Glyma11G29490,Glyma08G42790,Glyma06G04540,Glyma07G07550,Glyma13G17440,Glyma01G38780,Glyma12G20860,Glyma02G45800,Glyma05G01380,Glyma13G32770,Glyma16G34030,Glyma17G03300,Glyma08G08500,Glyma03G32550,Glyma07G32200,Glyma09G31060,Glyma05G26040,Glyma11G13810,Glyma07G04740,Glyma07G00840,Glyma17G16950,Glyma17G04120,Glyma11G10240,Glyma06G21140,Glyma15G39150,Glyma09G08550,Glyma0048S00320,Glyma12G01380,Glyma04G03250,Glyma17G29990,Glyma08G14400,Glyma17G14760,Glyma07G32590,Glyma02G11290,Glyma17G01500,Glyma16G28070,Glyma07G06610,Glyma20G23930,Glyma07G17140,Glyma09G24410,Glyma12G37040,Glyma05G07540,Glyma13G18510,Glyma13G27810,Glyma10G07500,Glyma19G29820,Glyma18G43390,Glyma16G24610,Glyma11G01370,Glyma14G37620,Glyma03G40720,Glyma13G26280,Glyma09G33120,Glyma18G09290,Glyma14G04930,Glyma18G16060,Glyma03G32210,Glyma09G28040,Glyma20G33570,Glyma17G05880,Glyma20G38370,Glyma13G17710,Glyma15G01010,Glyma08G16150,Glyma18G15150,Glyma14G08260,Glyma07G31660,Glyma02G42270,Glyma08G39070,Glyma12G32880,Glyma06G19310,Glyma03G05640,Glyma02G04310,Glyma07G31630,Glyma18G52290,Glyma04G09580,Glyma19G37210,Glyma03G22960,Glyma03G02310,Glyma02G48020,Glyma01G27440,Glyma20G34160,Glyma03G30310,Glyma12G04200,Glyma14G01730,Glyma15G13290,Glyma10G39540,Glyma03G32730,Glyma15G00590,Glyma13G25970,Glyma01G44350,Glyma18G11110,Glyma06G47650,Glyma08G21530,Glyma20G38380,Glyma20G34060,Glyma18G03800,Glyma05G34160,Glyma20G29840,Glyma08G40980,Glyma06G04140,Glyma20G24840,Glyma13G37080,Glyma01G42240,Glyma12G30010,Glyma18G06170,Glyma02G05210,Glyma14G02650,Glyma06G17270,Glyma17G02080,Glyma01G41600,Glyma16G33590,Glyma14G07750,Glyma05G00310,Glyma08G16680,Glyma09G39800,Glyma20G18980,Glyma19G01850,Glyma10G06500,Glyma11G02150,Glyma02G09370,Glyma10G41560,Glyma13G43480,Glyma15G23620,Glyma17G09700,Glyma05G37970,Glyma01G43190,Glyma09G39000,Glyma19G35620,Glyma10G41990,Glyma04G40970,Glyma20G06780,Glyma03G31820,Glyma05G36130,Glyma09G29980,Glyma01G36170,Glyma07G16260,Glyma08G44620,Glyma06G20200,Glyma02G42740,Glyma20G27790,Glyma02G33090,Glyma03G08980,Glyma09G00280,Glyma07G09820,Glyma13G26170,Glyma14G01440,Glyma09G02930,Glyma15G37290,Glyma20G38860,Glyma20G35970,Glyma09G02960,Glyma0220S00210,Glyma18G53590,Glyma17G16070,Glyma01G00960,Glyma09G40150,Glyma07G03160,Glyma09G27030,Glyma06G07380,Glyma09G23150,Glyma10G38370,Glyma02G03760,Glyma02G02580,Glyma18G14770,Glyma16G29510,Glyma09G00580,Glyma18G06570,Glyma18G38470,Glyma01G36980,Glyma14G01980,Glyma18G52110,Glyma12G01480,Glyma06G47870,Glyma02G15490,Glyma16G27330,Glyma10G40900,Glyma08G10880,Glyma12G02880,Glyma05G36200,Glyma19G43270,Glyma09G32510,Glyma20G30100,Glyma02G44040,Glyma18G48470,Glyma18G47140,Glyma07G31380,Glyma20G23480,Glyma06G40710,Glyma03G29280,Glyma06G40920,Glyma07G39980,Glyma05G23260,Glyma01G29540,Glyma06G11820,Glyma16G01090,Glyma15G34620,Glyma18G01530,Glyma11G02620,Glyma05G01510,Glyma10G03870,Glyma13G26970,Glyma07G11320,Glyma08G12750,Glyma13G34810,Glyma08G21740,Glyma15G00670,Glyma17G09120,Glyma03G00540,Glyma18G17290,Glyma12G34890,Glyma15G00340,Glyma07G05820,Glyma07G03660,Glyma18G10610,Glyma10G08700,Glyma19G41890,Glyma04G33950,Glyma01G34560,Glyma05G03440,Glyma05G35800,Glyma05G33540,Glyma18G43980,Glyma02G39150,Glyma06G21440,Glyma13G42680,Glyma06G12510,Glyma05G23680,Glyma15G36890,Glyma13G23610,Glyma17G05670,Glyma11G38230,Glyma03G39320,Glyma20G27660,Glyma10G42350,Glyma15G14810,Glyma08G13060,Glyma15G14210,Glyma17G14680,Glyma03G32690,Glyma19G34250,Glyma05G03310,Glyma03G42000,Glyma14G07700,Glyma15G05750,Glyma13G04800,Glyma05G03930,Glyma20G08160,Glyma06G38190,Glyma05G30260,Glyma07G02940,Glyma14G38650,Glyma09G28920,Glyma15G04280,Glyma05G27840,Glyma08G05720,Glyma11G03140,Glyma17G07940,Glyma08G43170
GO:006952	defense response	45/1695	395/27682	4.74E-05	Glyma13G25420,Glyma01G27440,Glyma06G39990,Glyma13G26250,Glyma18G09980,Glyma15G37320,Glyma15G13290,Glyma02G03760,Glyma18G51960,Glyma16G34030,Glyma16G03780,Glyma15G37390,Glyma13G25970,Glyma06G17560,Glyma06G40740,Glyma12G16450,Glyma20G06780,Glyma06G41880,Glyma06G40780,Glyma06G47650,Glyma03G40770,Glyma13G26000,Glyma07G07110,Glyma10G32800,Glyma18G09290,Glyma16G10080,Glyma06G40980,Glyma03G05400,Glyma16G25010,Glyma18G09670,Glyma09G29080,Glyma16G24940,Glyma18G10610,Glyma15G37140,Glyma06G40690,Glyma16G33950,Glyma15G37290,Glyma16G33590,Glyma18G10470,Glyma03G05640,Glyma18G51950,Glyma03G04780,Glyma06G41430,Glyma06G40710,Glyma08G43170

GO:016462	pyrophosphatase activity	114/1695	1296/27682	5.98E-05	Glyma20G26160,Glyma16G29040,Glyma18G00700,Glyma13G24260,Glyma04G38360,Glyma12G06530,Glyma06G07410,Glyma07G08120,Glyma03G32530,Glyma01G41150,Glyma12G34330,Glyma04G36240,Glyma10G30060,Glyma02G03070,Glyma06G16700,Glyma20G29060,Glyma11G15520,Glyma06G20200,Glyma0028S00210,Glyma09G28260,Glyma09G02930,Glyma19G38970,Glyma17G34060,Glyma05G34540,Glyma17G08540,Glyma20G16170,Glyma07G07550,Glyma19G31720,Glyma13G17440,Glyma04G04380,Glyma05G34180,Glyma05G25550,Glyma17G10420,Glyma09G38730,Glyma03G29230,Glyma18G07080,Glyma19G39580,Glyma13G35790,Glyma06G15900,Glyma10G32800,Glyma07G07110,Glyma04G05920,Glyma01G07970,Glyma06G21140,Glyma04G06610,Glyma20G23010,Glyma15G10370,Glyma11G04350,Glyma04G41060,Glyma08G09050,Glyma08G46130,Glyma06G18700,Glyma17G01310,Glyma04G07370,Glyma05G33830,Glyma08G41510,Glyma18G46930,Glyma19G38110,Glyma05G27850,Glyma13G10530,Glyma05G01460,Glyma17G34410,Glyma15G20000,Glyma11G19120,Glyma07G00350,Glyma16G21050,Glyma15G42030,Glyma09G05710,Glyma12G04410,Glyma15G00670,Glyma11G37910,Glyma20G37930,Glyma18G08870,Glyma09G34860,Glyma15G00340,Glyma12G33340,Glyma19G35270,Glyma04G01110,Glyma05G03440,Glyma16G10760,Glyma07G09530,Glyma04G34140,Glyma10G02020,Glyma11G04310,Glyma06G05910,Glyma03G30310,Glyma09G08370,Glyma06G21910,Glyma07G11880,Glyma16G27030,Glyma08G05120,Glyma14G40380,Glyma17G30980,Glyma18G45440,Glyma20G38380,Glyma06G20360,Glyma20G34060,Glyma20G29840,Glyma10G28880,Glyma20G38610,Glyma19G34250,Glyma04G39670,Glyma01G42240,Glyma02G09080,Glyma03G33590,Glyma10G35310,Glyma08G23150,Glyma05G31810,Glyma16G33590,Glyma07G02940,Glyma03G36310,Glyma14G07750,Glyma08G16680,Glyma09G08180
GO:017111	nucleoside-triphosphatase activity	112/1695	1271/27682	6.45E-05	Glyma20G26160,Glyma16G29040,Glyma18G00700,Glyma13G24260,Glyma04G38360,Glyma12G06530,Glyma06G07410,Glyma07G08120,Glyma03G32530,Glyma01G41150,Glyma12G34330,Glyma04G36240,Glyma10G30060,Glyma02G03070,Glyma06G16700,Glyma20G29060,Glyma11G15520,Glyma06G20200,Glyma0028S00210,Glyma09G28260,Glyma09G02930,Glyma19G38970,Glyma17G34060,Glyma05G34540,Glyma17G08540,Glyma20G16170,Glyma07G07550,Glyma19G31720,Glyma04G04380,Glyma13G17440,Glyma05G34180,Glyma05G25550,Glyma17G10420,Glyma09G38730,Glyma03G29230,Glyma18G07080,Glyma19G39580,Glyma13G35790,Glyma06G15900,Glyma10G32800,Glyma07G07110,Glyma04G05920,Glyma01G07970,Glyma06G21140,Glyma04G06610,Glyma20G23010,Glyma15G10370,Glyma11G04350,Glyma04G41060,Glyma08G09050,Glyma08G46130,Glyma06G18700,Glyma17G01310,Glyma04G07370,Glyma05G33830,Glyma08G41510,Glyma18G46930,Glyma19G38110,Glyma05G27850,Glyma13G10530,Glyma05G01460,Glyma17G34410,Glyma15G20000,Glyma11G19120,Glyma16G21050,Glyma15G42030,Glyma09G05710,Glyma12G04410,Glyma15G00670,Glyma11G37910,Glyma20G37930,Glyma18G08870,Glyma09G34860,Glyma15G00340,Glyma12G33340,Glyma19G35270,Glyma04G01110,Glyma05G03440,Glyma16G10760,Glyma07G09530,Glyma04G34140,Glyma10G02020,Glyma11G04310,Glyma06G05910,Glyma03G30310,Glyma09G08370,Glyma06G21910,Glyma07G11880,Glyma16G27030,Glyma08G05120,Glyma14G40380,Glyma17G30980,Glyma18G45440,Glyma20G38380,Glyma06G20360,Glyma20G34060,Glyma20G29840,Glyma20G38610,Glyma19G34250,Glyma04G39670,Glyma01G42240,Glyma02G09080,Glyma03G33590,Glyma10G35310,Glyma08G23150,Glyma05G31810,Glyma16G33590,Glyma07G02940,Glyma03G36310,Glyma14G07750,Glyma08G16680,Glyma09G08180
GO:016818	hydrolase activity, acting on acid anhydrides, in phosphorus-containing anhydrides	114/1695	1317/27682	1.15E-04	Glyma20G26160,Glyma16G29040,Glyma18G00700,Glyma13G24260,Glyma04G38360,Glyma12G06530,Glyma06G07410,Glyma07G08120,Glyma03G32530,Glyma01G41150,Glyma12G34330,Glyma04G36240,Glyma10G30060,Glyma02G03070,Glyma06G16700,Glyma20G29060,Glyma11G15520,Glyma06G20200,Glyma0028S00210,Glyma09G28260,Glyma09G02930,Glyma19G38970,Glyma17G34060,Glyma05G34540,Glyma17G08540,Glyma20G16170,Glyma07G07550,Glyma19G31720,Glyma13G17440,Glyma04G04380,Glyma05G34180,Glyma05G25550,Glyma17G10420,Glyma09G38730,Glyma03G29230,Glyma18G07080,Glyma19G39580,Glyma13G35790,Glyma06G15900,Glyma10G32800,Glyma07G07110,Glyma04G05920,Glyma01G07970,Glyma06G21140,Glyma04G06610,Glyma20G23010,Glyma15G10370,Glyma11G04350,Glyma04G41060,Glyma08G09050,Glyma08G46130,Glyma06G18700,Glyma17G01310,Glyma04G07370,Glyma05G33830,Glyma08G41510,Glyma18G46930,Glyma19G38110,Glyma05G27850,Glyma13G10530,Glyma05G01460,Glyma17G34410,Glyma15G20000,Glyma11G19120,Glyma07G00350,Glyma16G21050,Glyma15G42030,Glyma09G05710,Glyma12G04410,Glyma15G00670,Glyma11G37910,Glyma20G37930,Glyma18G08870,Glyma09G34860,Glyma15G00340,Glyma12G33340,Glyma19G35270,Glyma04G01110,Glyma05G03440,Glyma16G10760,Glyma07G09530,Glyma04G34140,Glyma10G02020,Glyma11G04310,Glyma06G05910,Glyma03G30310,Glyma09G08370,Glyma06G21910,Glyma07G11880,Glyma16G27030,Glyma08G05120,Glyma14G40380,Glyma17G30980,Glyma18G45440,Glyma20G38380,Glyma06G20360,Glyma20G34060,Glyma20G29840,Glyma10G28880,Glyma20G38610,Glyma19G34250,Glyma04G39670,Glyma01G42240,Glyma02G09080,Glyma03G33590,Glyma10G35310,Glyma08G23150,Glyma05G31810,Glyma16G33590,Glyma07G02940,Glyma03G36310,Glyma14G07750,Glyma08G16680,Glyma09G08180
GO:004553	hydrolase activity, hydrolyzing O-glycosyl compounds	60/1695	605/27682	1.68E-04	Glyma05G26960,Glyma16G04680,Glyma11G11500,Glyma06G15940,Glyma04G30870,Glyma15G23310,Glyma18G19670,Glyma05G36580,Glyma09G21970,Glyma17G05250,Glyma14G05300,Glyma05G24830,Glyma14G37070,Glyma07G07290,Glyma16G29780,Glyma14G04850,Glyma11G18350,Glyma19G31590,Glyma08G40810,Glyma13G41800,Glyma02G07770,Glyma15G02750,Glyma18G52290,Glyma14G34480,Glyma19G00230,Glyma13G42680,Glyma08G26940,Glyma03G00440,Glyma07G01250,Glyma15G37670,Glyma06G03160,Glyma17G33190,Glyma08G27000,Glyma07G12060,Glyma04G03120,Glyma17G03300,Glyma17G07280,Glyma18G06570,Glyma13G38120,Glyma01G36980,Glyma11G13810,Glyma14G39930,Glyma12G01480,Glyma14G03140,Glyma02G05790,Glyma01G45470,Glyma07G12010,Glyma09G39200,Glyma14G07700,Glyma06G17270,Glyma03G27470,Glyma06G12150,Glyma09G28920,Glyma11G07760,Glyma02G07740,Glyma05G08730,Glyma09G07100,Glyma08G20650,Glyma06G14920,Glyma08G39340

GO:016798	hydrolase activity, acting on glycosyl bonds	62/1695	632/27682	1.78E-04	Glyma05G26960,Glyma16G04680,Glyma11G11500,Glyma06G15940,Glyma15G23310,Glyma04G30870,Glyma18G19670,Glyma05G36580,Glyma09G21970,Glyma17G05250,Glyma14G05300,Glyma05G24830,Glyma14G37070,Glyma07G07290,Glyma16G29780,Glyma14G04850,Glyma11G18350,Glyma19G31590,Glyma08G40810,Glyma13G41800,Glyma06G12160,Glyma02G07770,Glyma15G02750,Glyma17G11670,Glyma18G52290,Glyma14G34480,Glyma19G00230,Glyma13G42680,Glyma08G26940,Glyma03G00440,Glyma07G01250,Glyma15G37670,Glyma06G03160,Glyma17G33190,Glyma08G27000,Glyma07G12060,Glyma04G03120,Glyma17G03300,Glyma17G07280,Glyma18G06570,Glyma13G38120,Glyma01G36980,Glyma11G13810,Glyma14G39930,Glyma12G01480,Glyma14G03140,Glyma02G05790,Glyma01G45470,Glyma07G12010,Glyma09G39200,Glyma14G07700,Glyma06G17270,Glyma03G27470,Glyma06G12150,Glyma09G28920,Glyma11G07760,Glyma02G07740,Glyma05G08730,Glyma09G07100,Glyma08G20650,Glyma06G14920,Glyma08G39340
GO:016787	hydrolase activity	307/1695	4203/27682	3.86E-04	Glyma19G41590,Glyma06G15940,Glyma05G36580,Glyma20G26160,Glyma17G05250,Glyma20G24570,Glyma18G00700,Glyma04G38360,Glyma13G24260,Glyma12G06530,Glyma06G07410,Glyma07G08120,Glyma14G37070,Glyma12G34330,Glyma14G04850,Glyma10G30060,Glyma02G03070,Glyma17G04800,Glyma19G31590,Glyma06G16700,Glyma11G15520,Glyma09G02490,Glyma06G08730,Glyma0028S00210,Glyma18G49000,Glyma09G00810,Glyma20G00270,Glyma10G12660,Glyma05G34540,Glyma12G35390,Glyma14G34480,Glyma17G08540,Glyma15G09700,Glyma20G16170,Glyma06G04540,Glyma08G26940,Glyma07G07550,Glyma08G22520,Glyma05G35930,Glyma19G31720,Glyma11G37170,Glyma10G36220,Glyma13G17440,Glyma06G42640,Glyma04G04380,Glyma05G34180,Glyma17G33190,Glyma05G25550,Glyma17G10420,Glyma15G02780,Glyma17G03300,Glyma18G07080,Glyma13G35790,Glyma19G39580,Glyma14G09510,Glyma06G15900,Glyma07G18500,Glyma11G13810,Glyma07G07110,Glyma04G24380,Glyma04G05920,Glyma17G16950,Glyma17G04120,Glyma15G13380,Glyma01G07970,Glyma06G21140,Glyma07G12010,Glyma14G40510,Glyma02G43200,Glyma09G39200,Glyma19G22790,Glyma20G06680,Glyma10G36750,Glyma20G23010,Glyma07G32590,Glyma15G10370,Glyma17G01500,Glyma04G41060,Glyma08G09050,Glyma08G46130,Glyma02G07740,Glyma19G30650,Glyma04G07370,Glyma07G38560,Glyma05G07540,Glyma08G41510,Glyma05G33830,Glyma05G26960,Glyma08G21340,Glyma07G32540,Glyma17G02150,Glyma15G23310,Glyma18G19670,Glyma05G27850,Glyma13G27840,Glyma09G21970,Glyma17G13910,Glyma17G12550,Glyma13G34290,Glyma12G34960,Glyma18G43390,Glyma20G39350,Glyma09G09390,Glyma17G17780,Glyma17G34410,Glyma15G20000,Glyma11G19120,Glyma07G07290,Glyma18G48520,Glyma16G21050,Glyma05G03320,Glyma11G37910,Glyma20G37930,Glyma09G34860,Glyma06G05770,Glyma13G17710,Glyma05G33270,Glyma08G10900,Glyma01G08600,Glyma18G52290,Glyma03G00440,Glyma04G08620,Glyma07G01250,Glyma11G04310,Glyma15G37670,Glyma03G30310,Glyma12G04200,Glyma14G05930,Glyma04G42830,Glyma09G08370,Glyma06G21910,Glyma07G11880,Glyma04G03120,Glyma14G40380,Glyma18G45440,Glyma04G16670,Glyma20G38380,Glyma06G20360,Glyma20G34060,Glyma20G29840,Glyma20G38610,Glyma10G28880,Glyma14G06970,Glyma10G23520,Glyma06G04140,Glyma20G34790,Glyma05G24050,Glyma01G42240,Glyma08G11940,Glyma18G06170,Glyma02G05210,Glyma10G29700,Glyma02G09080,Glyma06G17270,Glyma03G33590,Glyma16G33590,Glyma03G36310,Glyma14G07750,Glyma08G16680,Glyma05G08730,Glyma17G11760,Glyma08G20650,Glyma13G26680,Glyma06G44970,Glyma14G12880,Glyma16G04680,Glyma15G02980,Glyma12G30410,Glyma02G07690,Glyma13G33320,Glyma14G12420,Glyma16G29040,Glyma03G10390,Glyma05G24830,Glyma02G15150,Glyma03G32530,Glyma01G41150,Glyma16G29780,Glyma02G42420,Glyma04G36240,Glyma11G18350,Glyma19G37860,Glyma02G39190,Glyma20G29060,Glyma08G40810,Glyma13G41800,Glyma06G20200,Glyma08G24910,Glyma15G02430,Glyma06G12160,Glyma12G08200,Glyma09G28260,Glyma09G02930,Glyma20G39110,Glyma19G38970,Glyma17G34060,Glyma14G04890,Glyma07G01230,Glyma18G07130,Glyma02G07450,Glyma09G31250,Glyma09G38730,Glyma07G12060,Glyma03G29230,Glyma17G07280,Glyma20G19210,Glyma18G06570,Glyma01G36980,Glyma18G09380,Glyma01G01500,Glyma14G39930,Glyma14G03140,Glyma10G32800,Glyma12G01480,Glyma15G21010,Glyma07G09030,Glyma08G10880,Glyma12G02880,Glyma02G13630,Glyma04G06610,Glyma11G04350,Glyma06G12150,Glyma11G07760,Glyma06G18700,Glyma17G01310,Glyma06G47490,Glyma09G07100,Glyma06G14920,Glyma11G11500,Glyma04G43480,Glyma18G46930,Glyma13G35610,Glyma19G38110,Glyma04G30870,Glyma08G20140,Glyma13G10530,Glyma16G01090,Glyma05G01460,Glyma14G05300,Glyma15G29980,Glyma07G00350,Glyma15G42030,Glyma07G11320,Glyma08G12750,Glyma09G05710,Glyma01G01630,Glyma12G04410,Glyma19G07330,Glyma15G00670,Glyma18G08870,Glyma03G03360,Glyma11G11410,Glyma15G00340,Glyma20G16020,Glyma07G16020,Glyma12G33340,Glyma02G07770,Glyma15G02750,Glyma19G35270,Glyma17G11670,Glyma04G01110,Glyma10G30150,Glyma05G03440,Glyma16G10760,Glyma07G09530,Glyma19G00230,Glyma13G42680,Glyma02G43440,Glyma04G34140,Glyma11G32610,Glyma10G02020,Glyma20G36220,Glyma06G05910,Glyma17G05670,Glyma06G03160,Glyma08G27000,Glyma16G27030,Glyma08G05120,Glyma17G30980,Glyma13G38120,Glyma02G03320,Glyma15G14810,Glyma10G31160,Glyma17G14680,Glyma12G01450,Glyma03G32690,Glyma07G36500.4,Glyma19G34250,Glyma03G24720,Glyma04G39670,Glyma10G07870,Glyma02G05790,Glyma20G29700,Glyma01G45470,Glyma14G07700,Glyma09G06570,Glyma06G48250,Glyma10G35310,Glyma03G27470,Glyma05G31810,Glyma08G23150,Glyma07G02940,Glyma09G28920,Glyma05G27840,Glyma15G05350,Glyma09G08180,Glyma08G39340
GO:004888	transmembrane receptor activity	25/1695	197/27682	4.45E-04	Glyma01G27440,Glyma02G03760,Glyma16G34030,Glyma16G03780,Glyma12G16450,Glyma04G01760,Glyma06G40740,Glyma20G06780,Glyma06G41880,Glyma06G40780,Glyma19G41210,Glyma03G38620,Glyma10G32800,Glyma16G10080,Glyma06G40980,Glyma13G38460,Glyma16G25010,Glyma09G29080,Glyma16G24940,Glyma16G06670,Glyma06G40690,Glyma16G33950,Glyma16G33590,Glyma06G41430,Glyma06G40710

GO:004672	protein kinase activity	162/1695	2056/27682	4.97E-04	Glyma04G04510,Glyma04G09210,Glyma18G44800,Glyma11G02150,Glyma18G50540,Glyma01G42770,Glyma13G43480,Glyma05G21420,Glyma14G36660,Glyma13G36600,Glyma02G06700,Glyma14G00380,Glyma01G03410,Glyma09G30430,Glyma14G10790,Glyma09G35140,Glyma16G32830,Glyma18G50510,Glyma13G18690,Glyma07G16260,Glyma08G44620,Glyma08G40920,Glyma16G08560,Glyma12G08900,Glyma20G27790,Glyma17G34190,Glyma09G05550,Glyma20G27800,Glyma09G37580,Glyma20G35970,Glyma09G03190,Glyma01G39380,Glyma17G01730,Glyma10G38810,Glyma18G45200,Glyma17G16070,Glyma02G46070,Glyma07G30260,Glyma13G23840,Glyma09G40150,Glyma08G06730,Glyma02G46670,Glyma14G05060,Glyma02G45800,Glyma08G06520,Glyma14G33400,Glyma02G11110,Glyma16G07060,Glyma13G21820,Glyma05G02470,Glyma18G38470,Glyma06G45590,Glyma19G41210,Glyma18G48600,Glyma05G33240,Glyma06G47870,Glyma18G47590,Glyma06G40110,Glyma07G01890,Glyma06G08880,Glyma13G02620,Glyma02G47230,Glyma18G50650,Glyma15G36110,Glyma20G30100,Glyma18G49060,Glyma14G03040,Glyma01G34780,Glyma18G47140,Glyma20G27720,Glyma13G42950,Glyma18G50450,Glyma18G50440,Glyma06G40920,Glyma05G23260,Glyma18G40680,Glyma10G07500,Glyma14G36960,Glyma11G34210,Glyma04G01440,Glyma17G04470,Glyma13G10040,Glyma15G17430,Glyma09G13540,Glyma06G02010,Glyma06G08480,Glyma03G38620,Glyma09G33120,Glyma09G19730,Glyma07G31140,Glyma03G00540,Glyma18G04440,Glyma11G09060,Glyma18G16060,Glyma13G16540,Glyma02G48100,Glyma12G34890,Glyma10G32990,Glyma06G40350,Glyma07G10460,Glyma14G29130,Glyma18G15150,Glyma08G39070,Glyma12G32880,Glyma06G40670,Glyma09G40650,Glyma15G04850,Glyma01G10100,Glyma16G32710,Glyma19G00650,Glyma18G42700,Glyma14G06570,Glyma03G02310,Glyma10G35610,Glyma15G02520,Glyma03G32160,Glyma13G23610,Glyma16G32640,Glyma12G17340,Glyma05G30030,Glyma13G40550,Glyma13G00370,Glyma13G06620,Glyma19G43290,Glyma03G37760,Glyma17G38210,Glyma20G27660,Glyma15G34810,Glyma07G09260,Glyma08G13040,Glyma16G32700,Glyma13G38950,Glyma08G13150,Glyma08G13060,Glyma17G33470,Glyma07G00670,Glyma17G34170,Glyma12G17450,Glyma12G17360,Glyma16G02290,Glyma14G12710,Glyma16G22370,Glyma13G06490,Glyma11G34490,Glyma02G14310,Glyma20G27510,Glyma16G27250,Glyma08G40030,Glyma05G30260,Glyma09G37650,Glyma14G38650,Glyma12G36440,Glyma15G04280,Glyma08G08000,Glyma02G40850,Glyma16G02340,Glyma07G00340,Glyma15G04350,Glyma19G04140,Glyma11G09070,Glyma04G43190,Glyma08G05720
GO:006468	protein amino acid phosphorylation	161/1695	2046/27682	5.48E-04	Glyma04G04510,Glyma04G09210,Glyma18G44800,Glyma11G02150,Glyma18G50540,Glyma01G42770,Glyma13G43480,Glyma05G21420,Glyma14G36660,Glyma13G36600,Glyma02G06700,Glyma14G00380,Glyma01G03410,Glyma09G30430,Glyma14G10790,Glyma09G35140,Glyma16G32830,Glyma18G50510,Glyma13G18690,Glyma07G16260,Glyma08G44620,Glyma08G40920,Glyma16G08560,Glyma12G08900,Glyma20G27790,Glyma17G34190,Glyma09G05550,Glyma20G27800,Glyma09G37580,Glyma20G35970,Glyma09G03190,Glyma01G39380,Glyma17G01730,Glyma10G38810,Glyma18G45200,Glyma17G16070,Glyma02G46070,Glyma07G30260,Glyma13G23840,Glyma09G40150,Glyma08G06730,Glyma02G46670,Glyma14G05060,Glyma02G45800,Glyma08G06520,Glyma14G33400,Glyma02G11110,Glyma16G07060,Glyma13G21820,Glyma05G02470,Glyma18G38470,Glyma06G45590,Glyma19G41210,Glyma18G48600,Glyma05G33240,Glyma06G47870,Glyma18G47590,Glyma06G40110,Glyma07G01890,Glyma06G08880,Glyma13G02620,Glyma02G47230,Glyma18G50650,Glyma15G36110,Glyma20G30100,Glyma18G49060,Glyma14G03040,Glyma01G34780,Glyma18G47140,Glyma20G27720,Glyma13G42950,Glyma18G50450,Glyma18G50440,Glyma06G40920,Glyma05G23260,Glyma18G40680,Glyma10G07500,Glyma14G36960,Glyma11G34210,Glyma04G01440,Glyma17G04470,Glyma13G10040,Glyma15G17430,Glyma09G13540,Glyma06G02010,Glyma06G08480,Glyma09G33120,Glyma09G19730,Glyma07G31140,Glyma03G00540,Glyma18G04440,Glyma11G09060,Glyma18G16060,Glyma13G16540,Glyma02G48100,Glyma12G34890,Glyma10G32990,Glyma06G40350,Glyma07G10460,Glyma14G29130,Glyma18G15150,Glyma08G39070,Glyma12G32880,Glyma06G40670,Glyma09G40650,Glyma15G04850,Glyma01G10100,Glyma16G32710,Glyma19G00650,Glyma18G42700,Glyma14G06570,Glyma03G02310,Glyma10G35610,Glyma15G02520,Glyma03G32160,Glyma13G23610,Glyma16G32640,Glyma12G17340,Glyma05G30030,Glyma13G40550,Glyma13G00370,Glyma13G06620,Glyma19G43290,Glyma03G37760,Glyma17G38210,Glyma20G27660,Glyma15G34810,Glyma07G09260,Glyma08G13040,Glyma16G32700,Glyma13G38950,Glyma08G13150,Glyma08G13060,Glyma17G33470,Glyma07G00670,Glyma17G34170,Glyma12G17450,Glyma12G17360,Glyma16G02290,Glyma14G12710,Glyma16G22370,Glyma13G06490,Glyma11G34490,Glyma02G14310,Glyma20G27510,Glyma16G27250,Glyma08G40030,Glyma05G30260,Glyma09G37650,Glyma14G38650,Glyma12G36440,Glyma15G04280,Glyma08G08000,Glyma02G40850,Glyma16G02340,Glyma07G00340,Glyma15G04350,Glyma19G04140,Glyma11G09070,Glyma04G43190,Glyma08G05720
GO:015298	solute:cation antiporter activity	12/1695	66/27682	6.01E-04	Glyma17G34780,Glyma10G30020,Glyma19G41890,Glyma19G42900,Glyma13G02910,Glyma03G40290,Glyma17G02560,Glyma06G47110,Glyma06G15970,Glyma03G39320,Glyma08G03320,Glyma03G32900
GO:015299	solute:hydrogen antiporter activity	12/1695	66/27682	6.01E-04	Glyma17G34780,Glyma10G30020,Glyma19G41890,Glyma19G42900,Glyma13G02910,Glyma03G40290,Glyma17G02560,Glyma06G47110,Glyma06G15970,Glyma03G39320,Glyma08G03320,Glyma03G32900

GO:031224	intrinsic to membrane	125/1695	1535/27682	6.52E-04	Glyma10G30020,Glyma04G35730,Glyma10G41560,Glyma20G38960,Glyma11G07090,Glyma01G43190,Glyma17G08610,Glyma17G34780,Glyma12G06530,Glyma11G07050,Glyma12G16450,Glyma04G01760,Glyma15G15810,Glyma20G06780,Glyma06G15970,Glyma16G19340,Glyma01G36170,Glyma02G14440,Glyma06G20200,Glyma16G25010,Glyma09G34400,Glyma02G08970,Glyma09G29080,Glyma0028S00210,Glyma11G09770,Glyma06G40690,Glyma13G43670,Glyma01G31840,Glyma06G47110,Glyma11G16270,Glyma13G05770,Glyma13G37440,Glyma09G00300,Glyma11G37170,Glyma02G03760,Glyma16G34030,Glyma16G03780,Glyma17G10420,Glyma12G09680,Glyma19G05460,Glyma14G05020,Glyma02G47820,Glyma06G41880,Glyma06G40780,Glyma12G32920,Glyma05G21910,Glyma10G32800,Glyma07G04740,Glyma07G00840,Glyma12G32210,Glyma15G11650,Glyma06G21140,Glyma12G06920,Glyma16G27330,Glyma12G07120,Glyma10G29000,Glyma16G06730,Glyma16G24940,Glyma09G32510,Glyma08G03320,Glyma16G33950,Glyma15G05190,Glyma20G25660,Glyma08G46130,Glyma17G02560,Glyma11G14960,Glyma06G41430,Glyma06G40710,Glyma05G01460,Glyma06G47470,Glyma18G01530,Glyma11G25250,Glyma06G40740,Glyma13G02910,Glyma16G25540,Glyma09G05710,Glyma16G32650,Glyma16G10080,Glyma15G00670,Glyma05G35710,Glyma10G44190,Glyma06G40980,Glyma13G38460,Glyma18G08870,Glyma03G00280,Glyma20G16020,Glyma12G33340,Glyma02G12320,Glyma19G41890,Glyma08G10900,Glyma04G33950,Glyma03G40290,Glyma02G39150,Glyma16G10760,Glyma15G01270,Glyma03G22960,Glyma02G48150,Glyma03G05440,Glyma01G27440,Glyma13G43950,Glyma13G44520,Glyma05G08880,Glyma18G08030,Glyma03G39320,Glyma10G31520,Glyma05G26750,Glyma19G42900,Glyma19G37360,Glyma15G25180,Glyma20G38380,Glyma08G45020,Glyma08G40980,Glyma20G04160,Glyma01G22650,Glyma12G36250,Glyma15G08530,Glyma10G29700,Glyma08G23150,Glyma01G22990,Glyma15G10630,Glyma13G07400,Glyma16G33590,Glyma07G02940,Glyma03G32900,Glyma05G38020
GO:004872	receptor activity	25/1695	203/27682	6.96E-04	Glyma01G27440,Glyma02G03760,Glyma16G34030,Glyma16G03780,Glyma12G16450,Glyma04G01760,Glyma06G40740,Glyma20G06780,Glyma06G41880,Glyma06G40780,Glyma19G41210,Glyma03G38620,Glyma10G32800,Glyma16G10080,Glyma06G40980,Glyma13G38460,Glyma16G25010,Glyma09G29080,Glyma16G24940,Glyma16G06670,Glyma06G40690,Glyma16G33950,Glyma16G33590,Glyma06G41430,Glyma06G40710
GO:004650	polygalacturonase activity	13/1695	77/27682	7.55E-04	Glyma15G23310,Glyma04G30870,Glyma06G15940,Glyma18G19670,Glyma09G39200,Glyma17G03300,Glyma07G07290,Glyma16G29780,Glyma14G04850,Glyma05G08730,Glyma12G01480,Glyma19G00230,Glyma08G39340
GO:004674	protein serine/threonine kinase activity	150/1695	1901/27682	7.55E-04	Glyma04G04510,Glyma04G09210,Glyma18G44800,Glyma11G02150,Glyma18G50540,Glyma01G42770,Glyma13G43480,Glyma05G21420,Glyma14G36660,Glyma13G36600,Glyma02G06700,Glyma01G03410,Glyma09G30430,Glyma14G10790,Glyma09G35140,Glyma13G18690,Glyma18G50510,Glyma07G16260,Glyma08G44620,Glyma16G08560,Glyma12G08900,Glyma20G27790,Glyma17G34190,Glyma09G05550,Glyma20G27800,Glyma09G37580,Glyma20G35970,Glyma09G03190,Glyma01G39380,Glyma17G01730,Glyma10G38810,Glyma18G45200,Glyma17G16070,Glyma02G46070,Glyma07G30260,Glyma13G23840,Glyma09G40150,Glyma08G06730,Glyma02G46670,Glyma14G05060,Glyma02G45800,Glyma08G06520,Glyma14G33400,Glyma02G11110,Glyma16G07060,Glyma13G21820,Glyma05G02470,Glyma18G38470,Glyma06G45590,Glyma18G48600,Glyma05G33240,Glyma06G47870,Glyma18G47590,Glyma06G40110,Glyma06G08880,Glyma07G01890,Glyma13G02620,Glyma02G47230,Glyma18G50650,Glyma15G36110,Glyma20G30100,Glyma18G49060,Glyma14G03040,Glyma01G34780,Glyma18G47140,Glyma20G27720,Glyma13G42950,Glyma18G50450,Glyma18G50440,Glyma06G40920,Glyma18G40680,Glyma10G07500,Glyma14G36960,Glyma11G34210,Glyma04G01440,Glyma13G10040,Glyma15G17430,Glyma09G13540,Glyma06G02010,Glyma06G08480,Glyma09G33120,Glyma09G19730,Glyma07G31140,Glyma03G00540,Glyma18G04440,Glyma11G09060,Glyma18G16060,Glyma13G16540,Glyma02G48100,Glyma12G34890,Glyma10G32990,Glyma06G40350,Glyma07G10460,Glyma14G29130,Glyma18G15150,Glyma08G39070,Glyma12G32880,Glyma06G40670,Glyma09G40650,Glyma15G04850,Glyma01G10100,Glyma16G32710,Glyma19G00650,Glyma18G42700,Glyma14G06570,Glyma03G02310,Glyma15G02520,Glyma03G32160,Glyma13G23610,Glyma16G32640,Glyma12G17340,Glyma05G30030,Glyma13G40550,Glyma13G00370,Glyma13G06620,Glyma17G38210,Glyma19G43290,Glyma20G27660,Glyma15G34810,Glyma07G09260,Glyma08G13040,Glyma13G38950,Glyma08G13060,Glyma17G33470,Glyma07G00670,Glyma17G34170,Glyma12G17450,Glyma12G17360,Glyma16G02290,Glyma14G12710,Glyma16G22370,Glyma02G14310,Glyma13G06490,Glyma20G27510,Glyma08G40030,Glyma16G27250,Glyma05G30260,Glyma09G37650,Glyma14G38650,Glyma12G36440,Glyma15G04280,Glyma02G40850,Glyma08G08000,Glyma16G02340,Glyma07G00340,Glyma15G04350,Glyma19G04140,Glyma11G09070,Glyma04G43190,Glyma08G05720
GO:008839	dihydrodipicolinate reductase activity	3/1695	4/27682	8.75E-04	Glyma20G29480,Glyma07G06610,Glyma10G38370
GO:016773	phosphotransferase activity, alcohol group as acceptor	178/1695	2318/27682	8.86E-04	Glyma04G04510,Glyma04G09210,Glyma18G44800,Glyma11G02150,Glyma18G50540,Glyma01G42770,Glyma05G21420,Glyma13G43480,Glyma17G09700,Glyma14G36660,Glyma13G36600,Glyma19G39680,Glyma02G06700,Glyma14G00380,Glyma01G03410,Glyma09G30430,Glyma14G10790,Glyma09G35140,Glyma16G32830,Glyma18G50510,Glyma13G18690,Glyma07G16260,Glyma08G44620,Glyma08G40920,Glyma16G08560,Glyma12G08900,Glyma20G27790,Glyma09G05550,Glyma17G34190,Glyma20G27800,Glyma09G37580,Glyma04G09340,Glyma20G35970,Glyma09G03190,Glyma01G39380,Glyma17G01730,Glyma10G38810,Glyma18G45200,Glyma17G16070,Glyma02G46070,Glyma07G30260,Glyma13G23840,Glyma09G40150,Glyma08G06730,Glyma02G46670,Glyma06G09490,Glyma09G23150,Glyma14G05060,Glyma02G45800,Glyma08G03240,Glyma08G06520,Glyma14G33400,Glyma02G11110,Glyma16G07060,Glyma13G21820,Glyma05G02470,Glyma18G38470,Glyma06G45590,Glyma19G41210,Glyma18G48600,Glyma05G33240,Glyma06G47870,Glyma08G06330,Glyma18G47590,Glyma13G21030,Glyma06G40110,Glyma07G01890,Glyma06G08880,Glyma19G44690,Glyma02G47230,Glyma13G02620,Glyma18G50650,Glyma09G30070,Glyma15G36110,Glyma20G30100,Glyma18G49060,Glyma14G03040,Glyma01G34780,Glyma18G47140,Glyma20G27720,Glyma13G05640,Glyma13G42950,Glyma18G50450,Glyma18G50440,Glyma06G40920,Glyma05G23260,Glyma18G40680,Glyma10G07500,Glyma14G36960,Glyma11G34210,Glyma04G01440,Glyma13G10040,Glyma17G04470,Glyma15G17430,Glyma09G13540,Glyma06G02010,Glyma09G26710,Glyma08G19860,Glyma06G08480,Glyma03G38620,Glyma09G33120,Glyma09G19730,Glyma07G31140,Glyma03G00540,Glyma

					18G04440,Glyma11G09060,Glyma18G16060,Glyma13G16540,Glyma07G12130,Glyma02G48100,Glyma12G34890,Glyma10G32990,Glyma06G40350,Glyma07G10460,Glyma14G29130,Glyma18G15150,Glyma08G39070,Glyma12G32880,Glyma06G40670,Glyma09G40650,Glyma15G04850,Glyma01G10100,Glyma16G32710,Glyma19G00650,Glyma18G42700,Glyma14G06570,Glyma03G02310,Glyma10G35610,Glyma15G02520,Glyma03G32160,Glyma13G23610,Glyma16G32640,Glyma12G17340,Glyma05G30030,Glyma13G40550,Glyma13G00370,Glyma13G06620,Glyma19G43290,Glyma03G37760,Glyma17G38210,Glyma20G27660,Glyma15G34810,Glyma07G09260,Glyma08G13040,Glyma16G32700,Glyma13G38950,Glyma08G13150,Glyma08G13060,Glyma17G33470,Glyma07G00670,Glyma17G34170,Glyma12G17450,Glyma12G17360,Glyma16G02290,Glyma14G12710,Glyma03G42000,Glyma10G34490,Glyma16G22370,Glyma13G06490,Glyma11G34490,Glyma02G14310,Glyma20G27510,Glyma16G27250,Glyma08G40030,Glyma05G30260,Glyma09G37650,Glyma14G38650,Glyma12G36440,Glyma15G04280,Glyma08G08000,Glyma02G40850,Glyma16G02340,Glyma07G00340,Glyma15G04350,Glyma19G04140,Glyma11G09070,Glyma04G43190,Glyma08G05720
GO:016310	phosphorylation	164/1695	2120/27682	1.03E-03	Glyma04G04510,Glyma04G09210,Glyma18G44800,Glyma11G02150,Glyma18G50540,Glyma01G42770,Glyma13G43480,Glyma05G21420,Glyma14G36660,Glyma13G36600,Glyma02G06700,Glyma14G00380,Glyma01G03410,Glyma09G30430,Glyma14G10790,Glyma09G35140,Glyma16G32830,Glyma18G50510,Glyma13G18690,Glyma07G16260,Glyma08G44620,Glyma08G40920,Glyma16G08560,Glyma12G08900,Glyma20G27790,Glyma17G34190,Glyma09G05550,Glyma20G27800,Glyma09G37580,Glyma20G35970,Glyma09G03190,Glyma01G39380,Glyma17G01730,Glyma10G38810,Glyma18G45200,Glyma17G16070,Glyma02G46070,Glyma07G30260,Glyma13G23840,Glyma09G40150,Glyma08G06730,Glyma02G46670,Glyma14G05060,Glyma02G45800,Glyma08G03240,Glyma08G06520,Glyma14G33400,Glyma02G11110,Glyma16G07060,Glyma13G21820,Glyma05G02470,Glyma18G38470,Glyma06G45590,Glyma19G41210,Glyma18G48600,Glyma05G33240,Glyma06G47870,Glyma18G47590,Glyma06G40110,Glyma07G01890,Glyma06G08880,Glyma19G44690,Glyma02G47230,Glyma13G02620,Glyma18G50650,Glyma15G36110,Glyma20G30100,Glyma18G49060,Glyma14G03040,Glyma01G34780,Glyma18G47140,Glyma20G27720,Glyma13G42950,Glyma18G50450,Glyma18G50440,Glyma06G40920,Glyma05G23260,Glyma18G40680,Glyma10G07500,Glyma14G36960,Glyma11G34210,Glyma04G01440,Glyma13G10040,Glyma17G04470,Glyma15G17430,Glyma09G13540,Glyma06G02010,Glyma06G08480,Glyma09G33120,Glyma09G19730,Glyma07G31140,Glyma03G00540,Glyma18G04440,Glyma11G09060,Glyma18G16060,Glyma13G16540,Glyma02G48100,Glyma12G34890,Glyma10G32990,Glyma06G40350,Glyma07G10460,Glyma14G29130,Glyma18G15150,Glyma08G39070,Glyma12G32880,Glyma06G40670,Glyma09G40650,Glyma15G04850,Glyma01G10100,Glyma16G32710,Glyma19G00650,Glyma18G42700,Glyma14G06570,Glyma03G02310,Glyma10G35610,Glyma15G02520,Glyma03G32160,Glyma13G23610,Glyma16G32640,Glyma12G17340,Glyma05G30030,Glyma13G40550,Glyma13G00370,Glyma13G06620,Glyma19G43290,Glyma03G37760,Glyma17G38210,Glyma20G27660,Glyma15G34810,Glyma07G09260,Glyma08G13040,Glyma16G32700,Glyma13G38950,Glyma08G13150,Glyma08G13060,Glyma17G33470,Glyma07G00670,Glyma17G34170,Glyma12G17450,Glyma12G17360,Glyma16G02290,Glyma14G12710,Glyma03G42000,Glyma16G22370,Glyma13G06490,Glyma11G34490,Glyma02G14310,Glyma20G27510,Glyma16G27250,Glyma08G40030,Glyma05G30260,Glyma09G37650,Glyma14G38650,Glyma12G36440,Glyma15G04280,Glyma08G08000,Glyma02G40850,Glyma16G02340,Glyma07G00340,Glyma15G04350,Glyma19G04140,Glyma11G09070,Glyma04G43190,Glyma08G05720
GO:016301	kinase activity	179/1695	2347/27682	1.17E-03	Glyma04G04510,Glyma04G09210,Glyma18G44800,Glyma11G02150,Glyma18G50540,Glyma01G42770,Glyma05G21420,Glyma13G43480,Glyma17G09700,Glyma14G36660,Glyma13G36600,Glyma05G32340,Glyma19G39680,Glyma02G06700,Glyma14G00380,Glyma01G03410,Glyma09G30430,Glyma14G10790,Glyma09G35140,Glyma16G32830,Glyma18G50510,Glyma13G18690,Glyma07G16260,Glyma08G44620,Glyma08G40920,Glyma16G08560,Glyma12G08900,Glyma20G27790,Glyma09G05550,Glyma17G34190,Glyma20G27800,Glyma09G37580,Glyma20G35970,Glyma09G03190,Glyma08G15610,Glyma01G39380,Glyma17G01730,Glyma10G38810,Glyma18G45200,Glyma17G16070,Glyma02G46070,Glyma07G30260,Glyma13G23840,Glyma09G40150,Glyma08G06730,Glyma02G46670,Glyma09G23150,Glyma14G05060,Glyma02G45800,Glyma08G03240,Glyma08G06520,Glyma14G33400,Glyma02G11110,Glyma16G07060,Glyma13G21820,Glyma05G02470,Glyma18G38470,Glyma06G45590,Glyma19G41210,Glyma18G48600,Glyma05G33240,Glyma06G47870,Glyma08G06330,Glyma18G47590,Glyma07G04560,Glyma13G21030,Glyma06G40110,Glyma07G01890,Glyma06G08880,Glyma02G47230,Glyma19G44690,Glyma13G02620,Glyma18G50650,Glyma09G30070,Glyma15G36110,Glyma20G30100,Glyma18G49060,Glyma14G03040,Glyma01G34780,Glyma18G47140,Glyma20G27720,Glyma13G05640,Glyma13G42950,Glyma18G50450,Glyma18G50440,Glyma06G40920,Glyma05G23260,Glyma18G40680,Glyma10G07500,Glyma14G36960,Glyma11G34210,Glyma04G01440,Glyma13G10040,Glyma17G04470,Glyma15G17430,Glyma09G13540,Glyma06G02010,Glyma08G19860,Glyma06G08480,Glyma03G38620,Glyma09G33120,Glyma09G19730,Glyma07G31140,Glyma03G00540,Glyma18G04440,Glyma11G09060,Glyma18G16060,Glyma13G16540,Glyma07G12130,Glyma02G48100,Glyma12G34890,Glyma10G32990,Glyma06G40350,Glyma07G10460,Glyma14G29130,Glyma18G15150,Glyma08G39070,Glyma11G05140,Glyma12G32880,Glyma06G40670,Glyma09G40650,Glyma15G04850,Glyma01G10100,Glyma16G32710,Glyma19G00650,Glyma18G42700,Glyma14G06570,Glyma03G02310,Glyma10G35610,Glyma15G02520,Glyma03G32160,Glyma13G23610,Glyma16G32640,Glyma12G17340,Glyma05G30030,Glyma13G40550,Glyma13G00370,Glyma13G06620,Glyma19G43290,Glyma03G37760,Glyma17G38210,Glyma20G27660,Glyma15G34810,Glyma07G09260,Glyma08G13040,Glyma16G32700,Glyma13G38950,Glyma08G13150,Glyma08G13060,Glyma17G33470,Glyma07G00670,Glyma17G34170,Glyma12G17450,Glyma12G17360,Glyma16G02290,Glyma14G12710,Glyma03G42000,Glyma10G34490,Glyma16G22370,Glyma13G06490,Glyma11G34490,Glyma02G14310,Glyma20G27510,Glyma16G27250,Glyma08G40030,Glyma05G30260,Glyma09G37650,Glyma14G38650,Glyma12G36440,Glyma15G04280,Glyma08G08000,Glyma02G40850,Glyma16G02340,Glyma07G00340,Glyma15G04350,Glyma19G04140,Glyma11G09070,Glyma04G43190,Glyma08G05720

GO:043170	macromolecule metabolic process	409/1695	5862/27682	1.34E-03	Glyma13G41790,Glyma14G35680,Glyma04G04510,Glyma16G09890,Glyma04G37770,Glyma17G04800,Glyma06G08730,Glyma13G26260,Glyma12G08900,Glyma0028S00210,Glyma09G00810,Glyma17G08540,Glyma15G09700,Glyma17G01730,Glyma08G26940,Glyma08G22520,Glyma13G23840,Glyma10G36220,Glyma18G49880,Glyma06G42640,Glyma17G33190,Glyma05G25550,Glyma14G33400,Glyma13G21820,Glyma18G32680,Glyma12G05460.3,Glyma06G45590,Glyma18G48600,Glyma07G18500,Glyma05G33240,Glyma14G33700,Glyma02G00490,Glyma02G47230,Glyma02G43200,Glyma04G41630,Glyma02G12030,Glyma15G36110,Glyma07G06620,Glyma20G23010,Glyma18G03120,Glyma17G09690,Glyma11G34170,Glyma08G10910,Glyma19G30650,Glyma18G50450,Glyma14G09950,Glyma05G26960,Glyma18G40680,Glyma20G31530,Glyma13G27840,Glyma09G09390,Glyma05G30650,Glyma16G17760,Glyma17G17780,Glyma03G26080,Glyma18G48520,Glyma17G09940,Glyma06G17550,Glyma06G08480,Glyma11G09060,Glyma09G34860,Glyma10G28890,Glyma01G10100,Glyma01G08600,Glyma14G06570,Glyma06G06380.2,Glyma15G02520,Glyma03G00420,Glyma11G04310,Glyma04G39120,Glyma11G04020,Glyma16G32640,Glyma12G17340,Glyma13G40550,Glyma04G42830,Glyma07G09260,Glyma06G21910,Glyma13G38950,Glyma08G13150,Glyma04G36150,Glyma11G13460,Glyma17G33470,Glyma07G00670,Glyma14G06970,Glyma16G02290,Glyma14G12710,Glyma17G10710,Glyma16G22370,Glyma10G29700,Glyma02G09080,Glyma08G40030,Glyma05G03640,Glyma07G31350,Glyma02G40850,Glyma16G02340,Glyma17G11760,Glyma10G38450.2,Glyma11G09070,Glyma04G43190,Glyma06G11940,Glyma13G26680,Glyma19G41050,Glyma14G12880,Glyma18G44800,Glyma19G27420,Glyma12G30410,Glyma01G42770,Glyma05G21420,Glyma16G05410,Glyma14G00380,Glyma11G15530,Glyma18G14560,Glyma08G40920,Glyma06G12160,Glyma09G05550,Glyma12G08200,Glyma09G28260,Glyma18G48350,Glyma11G02190,Glyma09G37580,Glyma12G03140,Glyma01G39380,Glyma10G38810,Glyma06G30860,Glyma02G46070,Glyma07G30260,Glyma08G06730,Glyma02G46670,Glyma14G05060,Glyma08G06520,Glyma16G07060,Glyma01G37090,Glyma14G22170,Glyma03G37490,Glyma18G09380,Glyma04G27730,Glyma15G21010,Glyma02G36720,Glyma18G47590,Glyma06G08880,Glyma06G40110,Glyma15G37240,Glyma13G02620,Glyma02G13630,Glyma18G50650,Glyma10G08040,Glyma08G18760,Glyma05G30230,Glyma14G03040,Glyma01G34780,Glyma09G02180,Glyma20G27720,Glyma18G50440,Glyma17G11130,Glyma14G36960,Glyma03G24260,Glyma13G25110,Glyma17G04470,Glyma15G29980,Glyma13G10040,Glyma15G17430,Glyma13G02420,Glyma10G04530,Glyma11G35100,Glyma03G38620,Glyma12G36820,Glyma18G04440,Glyma19G29170,Glyma15G00950,Glyma04G40150,Glyma13G10200,Glyma06G40670,Glyma09G40650,Glyma15G04850,Glyma16G32710,Glyma10G35610,Glyma19G30630,Glyma05G28150,Glyma04G00580,Glyma14G03380,Glyma20G36220,Glyma13G33560,Glyma15G06040,Glyma05G30030,Glyma08G27000,Glyma13G00370,Glyma19G43290,Glyma13G38120,Glyma07G36500.4,Glyma12G17360,Glyma03G24720,Glyma03G36090,Glyma10G12530,Glyma10G07870,Glyma17G10370,Glyma02G14310,Glyma11G34490,Glyma13G06490,Glyma11G31410,Glyma20G27510,Glyma09G06570,Glyma09G37650,Glyma12G36440,Glyma18G11010,Glyma10G29200,Glyma08G08000,Glyma15G05350,Glyma09G08180,Glyma11G02560,Glyma14G03310,Glyma04G09210,Glyma14G36660,Glyma13G36600,Glyma02G43080,Glyma12G06530,Glyma15G08040,Glyma01G03410,Glyma09G30430,Glyma19G34580,Glyma07G33200,Glyma13G18690,Glyma18G50510,Glyma16G08560,Glyma17G34190,Glyma18G01960,Glyma14G40460,Glyma09G03190,Glyma08G42790,Glyma06G04540,Glyma11G20180,Glyma09G00300,Glyma11G37170,Glyma12G20860,Glyma02G45800,Glyma02G11110,Glyma07G32200,Glyma05G26040,Glyma19G41210,Glyma04G24380,Glyma17G04120,Glyma17G16950,Glyma07G01890,Glyma0048S00320,Glyma17G01500,Glyma20G34690,Glyma05G32070,Glyma12G37040,Glyma09G24410,Glyma05G07540,Glyma13G18510,Glyma06G42800,Glyma14G05050,Glyma10G07500,Glyma15G23150,Glyma18G43390,Glyma11G34210,Glyma04G01440,Glyma09G33120,Glyma09G19730,Glyma18G16060,Glyma13G16540,Glyma09G28040,Glyma10G35220,Glyma20G33570,Glyma13G17710,Glyma08G16150,Glyma18G15150,Glyma05G33270,Glyma12G32880,Glyma08G10900,Glyma08G39070,Glyma07G31630,Glyma03G02310,Glyma04G08620,Glyma12G09220,Glyma12G04200,Glyma20G23080,Glyma17G38210,Glyma13G06620,Glyma15G34810,Glyma08G13040,Glyma16G32700,Glyma18G11110,Glyma01G01750,Glyma20G34060,Glyma10G23520,Glyma18G06170,Glyma12G30780,Glyma16G27250,Glyma17G02080,Glyma11G10100,Glyma05G00310,Glyma14G07750,Glyma07G00340,Glyma11G02150,Glyma10G06500,Glyma02G09370,Glyma18G50540,Glyma13G43480,Glyma13G33320,Glyma14G12420,Glyma16G25600,Glyma02G06700,Glyma10G41990,Glyma03G31820,Glyma01G41150,Glyma17G08000,Glyma07G00440,Glyma14G10790,Glyma09G29980,Glyma16G00480,Glyma09G35140,Glyma16G32830,Glyma07G16260,Glyma08G44620,Glyma20G27790,Glyma09G00280,Glyma14G01440,Glyma20G39110,Glyma09G02930,Glyma20G27800,Glyma14G04890,Glyma20G38860,Glyma20G35970,Glyma0220S00210,Glyma18G45200,Glyma18G53590,Glyma17G16070,Glyma01G00960,Glyma09G40150,Glyma06G07380,Glyma18G08220,Glyma02G02580,Glyma18G14770,Glyma09G03660,Glyma07G39300,Glyma17G07280,Glyma09G00580,Glyma05G02470,Glyma18G38470,Glyma14G03140,Glyma15G42640,Glyma06G47870,Glyma11G19880,Glyma15G14550,Glyma08G10880,Glyma12G02880,Glyma04G06610,Glyma05G36200,Glyma19G43270,Glyma20G30100,Glyma18G49060,Glyma02G44040,Glyma08G18860,Glyma18G47140,Glyma19G37820,Glyma13G42950,Glyma06G40920,Glyma05G23260,Glyma07G39980,Glyma06G11820,Glyma16G01090,Glyma05G01510,Glyma09G13540,Glyma06G02010,Glyma07G11320,Glyma07G31140,Glyma17G09120,Glyma03G00540,Glyma12G36570,Glyma18G04130,Glyma17G01570,Glyma02G48100,Glyma11G11410,Glyma12G34890,Glyma10G32990,Glyma06G40350,Glyma08G15360,Glyma14G29130,Glyma07G10460,Glyma05G03440,Glyma12G31840,Glyma19G00650,Glyma06G21440,Glyma18G42700,Glyma11G32610,Glyma13G23610,Glyma03G32160,Glyma17G05670,Glyma10G12350,Glyma10G34020,Glyma03G37760,Glyma20G27660,Glyma02G03320,Glyma08G13060,Glyma17G34170,Glyma17G14680,Glyma12G17450,Glyma08G09540,Glyma06G15860,Glyma05G03930,Glyma06G38190,Glyma05G30260,Glyma14G38650,Glyma15G04280,Glyma15G04350,Glyma19G04140,Glyma08G05720,Glyma17G07940
GO:006885	regulation of pH	4/1695	9/27682	1.38E-03	Glyma10G30020,Glyma19G42900,Glyma03G40290,Glyma17G02560
GO:055067	monovalent inorganic cation homeostasis	4/1695	9/27682	1.38E-03	Glyma10G30020,Glyma19G42900,Glyma03G40290,Glyma17G02560
GO:005451	monovalent cation:hydrogen antiporter activity	4/1695	9/27682	1.38E-03	Glyma10G30020,Glyma19G42900,Glyma03G40290,Glyma17G02560

GO:015491	cation:cation antiporter activity	4/1695	9/27682	1.38E-03	Glyma10G30020,Glyma19G42900,Glyma03G40290,Glyma17G02560
GO:015385	sodium:hydrogen antiporter activity	4/1695	9/27682	1.38E-03	Glyma10G30020,Glyma19G42900,Glyma03G40290,Glyma17G02560
GO:015300	solute:solute antiporter activity	13/1695	82/27682	1.38E-03	Glyma10G30020,Glyma13G43950,Glyma03G39320,Glyma08G03320,Glyma17G34780,Glyma19G41890,Glyma13G02910,Glyma19G42900,Glyma03G40290,Glyma17G02560,Glyma06G47110,Glyma06G15970,Glyma03G32900
GO:005975	carbohydrate metabolic process	104/1695	1270/27682	1.51E-03	Glyma14G03310,Glyma16G04680,Glyma06G15940,Glyma05G36580,Glyma17G05250,Glyma05G24830,Glyma14G37070,Glyma17G08000,Glyma16G29780,Glyma14G04850,Glyma11G18350,Glyma19G31590,Glyma04G09670,Glyma05G31450,Glyma16G32960,Glyma13G41800,Glyma08G40810,Glyma03G08980,Glyma02G10710,Glyma20G38860,Glyma04G09340,Glyma16G34290,Glyma14G34480,Glyma06G30860,Glyma09G27030,Glyma06G09490,Glyma09G23150,Glyma17G33190,Glyma12G20860,Glyma08G03240,Glyma07G12060,Glyma17G03300,Glyma17G07280,Glyma18G06570,Glyma01G36980,Glyma11G13810,Glyma18G52110,Glyma12G01480,Glyma14G03140,Glyma14G39930,Glyma14G33700,Glyma02G36720,Glyma09G08550,Glyma07G12010,Glyma12G01380,Glyma09G39200,Glyma02G12030,Glyma17G14760,Glyma09G30070,Glyma09G38460,Glyma05G30230,Glyma06G12150,Glyma11G07760,Glyma13G05640,Glyma02G07740,Glyma09G07100,Glyma09G28100,Glyma06G14920,Glyma05G26960,Glyma11G11500,Glyma06G09770,Glyma04G30870,Glyma15G23310,Glyma18G19670,Glyma09G21970,Glyma14G05300,Glyma07G07290,Glyma09G26710,Glyma13G02420,Glyma10G04530,Glyma12G36570,Glyma07G12130,Glyma02G07770,Glyma19G07410,Glyma15G02750,Glyma18G52290,Glyma12G31840,Glyma19G00230,Glyma13G42680,Glyma03G00440,Glyma07G01250,Glyma04G39120,Glyma15G37670,Glyma13G33560,Glyma06G03160,Glyma11G38230,Glyma04G03120,Glyma13G38120,Glyma02G05790,Glyma01G45470,Glyma10G34490,Glyma06G15860,Glyma14G07700,Glyma06G17270,Glyma05G03640,Glyma03G27470,Glyma05G03100,Glyma09G28920,Glyma09G39800,Glyma18G02160,Glyma05G08730,Glyma20G08780,Glyma08G20650,Glyma08G39340
GO:006796	phosphate metabolic process	167/1695	2186/27682	1.59E-03	Glyma04G04510,Glyma04G09210,Glyma18G44800,Glyma11G02150,Glyma18G50540,Glyma01G42770,Glyma13G43480,Glyma05G21420,Glyma14G36660,Glyma13G36600,Glyma02G06700,Glyma14G00380,Glyma01G03410,Glyma09G30430,Glyma14G10790,Glyma09G35140,Glyma16G32830,Glyma18G50510,Glyma13G18690,Glyma07G16260,Glyma08G44620,Glyma08G40920,Glyma16G08560,Glyma12G08900,Glyma20G27790,Glyma17G34190,Glyma09G05550,Glyma20G27800,Glyma09G37580,Glyma20G35970,Glyma09G03190,Glyma01G39380,Glyma17G01730,Glyma10G38810,Glyma18G45200,Glyma17G16070,Glyma02G46070,Glyma07G30260,Glyma13G23840,Glyma09G40150,Glyma08G06730,Glyma10G36220,Glyma02G46670,Glyma14G05060,Glyma02G45800,Glyma08G03240,Glyma08G06520,Glyma14G33400,Glyma02G11110,Glyma16G07060,Glyma13G21820,Glyma05G02470,Glyma18G38470,Glyma06G45590,Glyma19G41210,Glyma18G48600,Glyma05G33240,Glyma06G47870,Glyma18G47590,Glyma06G40110,Glyma07G01890,Glyma06G08880,Glyma19G44690,Glyma02G47230,Glyma13G02620,Glyma12G01380,Glyma18G50650,Glyma15G36110,Glyma20G30100,Glyma18G49060,Glyma14G03040,Glyma01G34780,Glyma18G47140,Glyma20G27720,Glyma13G42950,Glyma18G50450,Glyma18G50440,Glyma06G40920,Glyma05G23260,Glyma18G40680,Glyma10G07500,Glyma14G36960,Glyma11G34210,Glyma04G01440,Glyma13G10040,Glyma17G04470,Glyma15G17430,Glyma09G13540,Glyma06G02010,Glyma06G08480,Glyma09G33120,Glyma09G19730,Glyma07G31140,Glyma03G00540,Glyma18G04440,Glyma11G09060,Glyma18G16060,Glyma13G16540,Glyma02G48100,Glyma12G34890,Glyma10G32990,Glyma06G40350,Glyma07G10460,Glyma14G29130,Glyma19G07410,Glyma18G15150,Glyma08G39070,Glyma12G32880,Glyma06G40670,Glyma09G40650,Glyma15G04850,Glyma01G10100,Glyma16G32710,Glyma19G00650,Glyma18G42700,Glyma14G06570,Glyma03G02310,Glyma10G35610,Glyma15G02520,Glyma03G32160,Glyma13G23610,Glyma16G32640,Glyma12G17340,Glyma05G30030,Glyma13G40550,Glyma13G00370,Glyma13G06620,Glyma19G43290,Glyma03G37760,Glyma17G38210,Glyma20G27660,Glyma15G34810,Glyma07G09260,Glyma08G13040,Glyma16G32700,Glyma13G38950,Glyma08G13150,Glyma08G13060,Glyma17G33470,Glyma07G00670,Glyma17G34170,Glyma12G17450,Glyma12G17360,Glyma16G02290,Glyma14G12710,Glyma03G42000,Glyma16G22370,Glyma13G06490,Glyma11G34490,Glyma02G14310,Glyma20G27510,Glyma16G27250,Glyma08G40030,Glyma05G30260,Glyma09G37650,Glyma14G38650,Glyma12G36440,Glyma15G04280,Glyma08G08000,Glyma02G40850,Glyma16G02340,Glyma07G00340,Glyma15G04350,Glyma19G04140,Glyma11G09070,Glyma04G43190,Glyma08G05720

GO:006793	phosphorus metabolic process	167/1695	2186/27682	1.59E-03	Glyma04G04510,Glyma04G09210,Glyma18G44800,Glyma11G02150,Glyma18G50540,Glyma01G42770,Glyma13G43480,Glyma05G21420,Glyma14G36660,Glyma13G36600,Glyma02G06700,Glyma14G00380,Glyma01G03410,Glyma09G30430,Glyma14G10790,Glyma09G35140,Glyma16G32830,Glyma18G50510,Glyma13G18690,Glyma07G16260,Glyma08G44620,Glyma08G40920,Glyma16G08560,Glyma12G08900,Glyma20G27790,Glyma17G34190,Glyma09G05550,Glyma20G27800,Glyma09G37580,Glyma20G35970,Glyma09G03190,Glyma01G39380,Glyma17G01730,Glyma10G38810,Glyma18G45200,Glyma17G16070,Glyma02G46070,Glyma07G30260,Glyma13G23840,Glyma09G40150,Glyma08G06730,Glyma10G36220,Glyma02G46670,Glyma14G05060,Glyma02G45800,Glyma08G03240,Glyma08G06520,Glyma14G33400,Glyma02G11110,Glyma16G07060,Glyma13G21820,Glyma05G02470,Glyma18G38470,Glyma06G45590,Glyma19G41210,Glyma18G48600,Glyma05G33240,Glyma06G47870,Glyma18G47590,Glyma06G40110,Glyma07G01890,Glyma06G08880,Glyma19G44690,Glyma02G47230,Glyma13G02620,Glyma12G01380,Glyma18G50650,Glyma15G36110,Glyma20G30100,Glyma18G49060,Glyma14G03040,Glyma01G34780,Glyma18G47140,Glyma20G27720,Glyma13G42950,Glyma18G50450,Glyma18G50440,Glyma06G40920,Glyma05G23260,Glyma18G40680,Glyma10G07500,Glyma14G36960,Glyma11G34210,Glyma04G01440,Glyma13G10040,Glyma17G04470,Glyma15G17430,Glyma09G13540,Glyma06G02010,Glyma06G08480,Glyma09G33120,Glyma09G19730,Glyma07G31140,Glyma03G00540,Glyma18G04440,Glyma11G09060,Glyma18G16060,Glyma13G16540,Glyma02G48100,Glyma12G34890,Glyma10G32990,Glyma06G40350,Glyma07G10460,Glyma14G29130,Glyma19G07410,Glyma18G15150,Glyma08G39070,Glyma12G32880,Glyma06G40670,Glyma09G40650,Glyma15G04850,Glyma01G10100,Glyma16G32710,Glyma19G00650,Glyma18G42700,Glyma14G06570,Glyma03G02310,Glyma10G35610,Glyma15G02520,Glyma03G32160,Glyma13G23610,Glyma16G32640,Glyma12G17340,Glyma05G30030,Glyma13G40550,Glyma13G00370,Glyma13G06620,Glyma19G43290,Glyma03G37760,Glyma17G38210,Glyma20G27660,Glyma15G34810,Glyma07G09260,Glyma08G13040,Glyma16G32700,Glyma13G38950,Glyma08G13150,Glyma08G13060,Glyma17G33470,Glyma07G00670,Glyma17G34170,Glyma12G17450,Glyma12G17360,Glyma16G02290,Glyma14G12710,Glyma03G42000,Glyma16G22370,Glyma13G06490,Glyma11G34490,Glyma02G14310,Glyma20G27510,Glyma16G27250,Glyma08G40030,Glyma05G30260,Glyma09G37650,Glyma14G38650,Glyma12G36440,Glyma15G04280,Glyma08G08000,Glyma02G40850,Glyma16G02340,Glyma07G00340,Glyma15G04350,Glyma19G04140,Glyma11G09070,Glyma04G43190,Glyma08G05720
GO:002376	immune system process	20/1695	158/27682	1.63E-03	Glyma16G10080,Glyma01G27440,Glyma06G40980,Glyma16G25010,Glyma09G29080,Glyma16G24940,Glyma02G03760,Glyma16G34030,Glyma16G03780,Glyma06G40690,Glyma16G33950,Glyma06G40740,Glyma12G16450,Glyma20G06780,Glyma16G33590,Glyma06G41880,Glyma06G40780,Glyma10G32800,Glyma06G41430,Glyma06G40710
GO:045087	innate immune response	20/1695	158/27682	1.63E-03	Glyma16G10080,Glyma01G27440,Glyma06G40980,Glyma16G25010,Glyma09G29080,Glyma16G24940,Glyma02G03760,Glyma16G34030,Glyma16G03780,Glyma06G40690,Glyma16G33950,Glyma06G40740,Glyma12G16450,Glyma20G06780,Glyma16G33590,Glyma06G41880,Glyma06G40780,Glyma10G32800,Glyma06G41430,Glyma06G40710
GO:006955	immune response	20/1695	158/27682	1.63E-03	Glyma16G10080,Glyma01G27440,Glyma06G40980,Glyma16G25010,Glyma09G29080,Glyma16G24940,Glyma02G03760,Glyma16G34030,Glyma16G03780,Glyma06G40690,Glyma16G33950,Glyma06G40740,Glyma12G16450,Glyma20G06780,Glyma16G33590,Glyma06G41880,Glyma06G40780,Glyma10G32800,Glyma06G41430,Glyma06G40710
GO:016887	ATPase activity	53/1695	571/27682	1.76E-03	Glyma08G41510,Glyma13G10530,Glyma20G26160,Glyma05G01460,Glyma07G08120,Glyma15G20000,Glyma03G32530,Glyma16G21050,Glyma01G41150,Glyma09G05710,Glyma12G04410,Glyma20G29060,Glyma15G00670,Glyma11G37910,Glyma18G08870,Glyma06G20200,Glyma09G34860,Glyma15G00340,Glyma12G33340,Glyma09G02930,Glyma19G38970,Glyma19G35270,Glyma05G03440,Glyma16G10760,Glyma20G16170,Glyma04G34140,Glyma05G34180,Glyma05G25550,Glyma17G10420,Glyma09G38730,Glyma09G08370,Glyma07G11880,Glyma03G29230,Glyma18G07080,Glyma17G30980,Glyma06G15900,Glyma20G38380,Glyma06G20360,Glyma20G34060,Glyma20G38610,Glyma19G34250,Glyma04G39670,Glyma01G07970,Glyma06G21140,Glyma02G09080,Glyma20G23010,Glyma03G33590,Glyma10G35310,Glyma08G23150,Glyma07G02940,Glyma08G46130,Glyma03G36310,Glyma09G08180

1. Maere S. *et al*. BiNGO: a Cytoscape plugin to assess overrepresentation of gene ontology categories in biological networks. *Bioinformatics*, **21**, 3448-3449 (2005).

2. Falcon S. & Gentleman R. Using GOstats to test gene lists for GO term association. *Bioinformatics*, **23**, 257–258 (2007).

Supplementary Table 6. Region of Present Absent Variation (PAV) in three gene pools (wild, landrace and elite) and gene list.

PAV_genes	Wild	Landrace	Elite	Interpro
GlymaPAV0000	0	3	3	IPR005162; Retrotransposon gag protein
GlymaPAV0001	0	1	0	IPR000780; MCP methyltransferase, CheR-type IPR022642; MCP methyltransferase, CheR-type, SAM-binding domain, C-terminal
GlymaPAV0002	5	5	12	IPR005162; Retrotransposon gag protein
GlymaPAV0003	3	3	0	
GlymaPAV0004	3	4	5	
GlymaPAV0005	0	1	2	
GlymaPAV0006	0	0	2	
GlymaPAV0007	0	0	10	
GlymaPAV0008	2	9	0	IPR001128; Cytochrome P450
GlymaPAV0009	1	0	0	IPR005162; Retrotransposon gag protein
GlymaPAV0010	3	2	4	IPR002559; Transposase, IS4-like IPR003201; Transposase Tn5, dimerisation
GlymaPAV0011	1	0	2	
GlymaPAV0012	0	2	2	IPR002525; Transposase, IS111A/IS1328/IS1533, N-terminal IPR003346; Transposase, IS116/IS110/IS902
GlymaPAV0013	0	6	3	
GlymaPAV0014	1	6	0	
GlymaPAV0015	0	0	1	
GlymaPAV0016	0	0	1	
GlymaPAV0017	1	10	4	
GlymaPAV0018	0	1	1	
GlymaPAV0019	3	3	1	IPR005162; Retrotransposon gag protein
GlymaPAV0020	0	1	0	
GlymaPAV0021	0	2	0	IPR007087; Zinc finger, C2H2-type IPR015880; Zinc finger, C2H2-like
GlymaPAV0022	0	0	2	
GlymaPAV0023	3	0	4	
GlymaPAV0024	0	0	1	IPR002068; Heat shock protein Hsp20
GlymaPAV0025	0	1	1	IPR000157; Toll-Interleukin receptor
GlymaPAV0026	0	1	0	IPR000568; ATPase, F0 complex, subunit A IPR023011; ATPase, F0 complex, subunit A, active site
GlymaPAV0027	1	1	0	
GlymaPAV0028	0	4	2	IPR015706; RNA-directed DNA polymerase (reverse transcriptase)
GlymaPAV0029	1	11	4	IPR013103; Reverse transcriptase, RNA-dependent DNA polymerase
GlymaPAV0030	7	11	4	IPR001611; Leucine-rich repeat
GlymaPAV0031	1	1	6	IPR001584; Integrase, catalytic core
GlymaPAV0032	1	0	0	IPR001584; Integrase, catalytic core
GlymaPAV0033	4	4	3	IPR001584; Integrase, catalytic core IPR015416; Zinc finger, H2C2-type, histone UAS binding
GlymaPAV0034	1	0	0	IPR000719; Protein kinase, catalytic domain IPR017442; Serine/threonine-protein kinase-like domain
GlymaPAV0035	0	1	0	
GlymaPAV0036	3	0	0	
GlymaPAV0037	1	1	5	
GlymaPAV0038	1	0	0	IPR000477; Reverse transcriptase IPR015706; RNA-directed DNA polymerase (reverse transcriptase)
GlymaPAV0039	2	0	0	
GlymaPAV0040	1	0	0	
GlymaPAV0041	1	2	0	IPR001584; Integrase, catalytic core IPR015416; Zinc finger, H2C2-type, histone UAS binding
GlymaPAV0042	0	0	2	
GlymaPAV0043	6	5	0	IPR000477; Reverse transcriptase IPR015706; RNA-directed DNA polymerase (reverse transcriptase)
GlymaPAV0044	2	0	0	
GlymaPAV0045	0	1	1	IPR013103; Reverse transcriptase, RNA-dependent DNA polymerase
GlymaPAV0046	3	23	8	
GlymaPAV0047	0	0	1	IPR000477; Reverse transcriptase IPR015706; RNA-directed DNA polymerase (reverse transcriptase)
GlymaPAV0048	1	1	1	
GlymaPAV0049	1	5	2	IPR019557; Aminotransferase-like, plant mobile domain
GlymaPAV0050	0	2	0	

GlymaPAV0051	0	1	1	IPR001584; Integrase, catalytic core
GlymaPAV0052	7	0	4	IPR013103; Reverse transcriptase, RNA-dependent DNA polymerase
GlymaPAV0053	1	9	5	IPR001584; Integrase, catalytic core
GlymaPAV0054	0	5	1	
GlymaPAV0055	2	2	6	IPR013103; Reverse transcriptase, RNA-dependent DNA polymerase
GlymaPAV0056	1	4	0	
GlymaPAV0057	1	2	4	IPR001584; Integrase, catalytic core
GlymaPAV0058	6	15	28	IPR001584; Integrase, catalytic core IPR015416; Zinc finger, H2C2-type, histone UAS binding
GlymaPAV0059	2	1	0	IPR019557; Aminotransferase-like, plant mobile domain
GlymaPAV0060	0	12	0	
GlymaPAV0061	0	0	1	
GlymaPAV0062	1	0	0	
GlymaPAV0063	1	0	0	
GlymaPAV0064	0	1	0	IPR013103; Reverse transcriptase, RNA-dependent DNA polymerase
GlymaPAV0065	4	3	6	IPR001584; Integrase, catalytic core
GlymaPAV0066	4	2	0	IPR000477; Reverse transcriptase
GlymaPAV0067	2	0	0	
GlymaPAV0068	9	38	37	IPR005162; Retrotransposon gag protein
GlymaPAV0069	1	16	11	IPR013103; Reverse transcriptase, RNA-dependent DNA polymerase
GlymaPAV0070	2	2	0	IPR019557; Aminotransferase-like, plant mobile domain
GlymaPAV0071	7	4	3	IPR013103; Reverse transcriptase, RNA-dependent DNA polymerase
GlymaPAV0072	1	2	0	
GlymaPAV0073	0	2	0	
GlymaPAV0074	0	1	1	IPR001584; Integrase, catalytic core
GlymaPAV0075	1	0	0	
GlymaPAV0076	0	1	0	IPR001584; Integrase, catalytic core
GlymaPAV0077	4	0	2	IPR005162; Retrotransposon gag protein
GlymaPAV0078	1	3	9	IPR004242; Transposon, En/Spm-like
GlymaPAV0079	0	3	4	
GlymaPAV0080	1	2	1	
GlymaPAV0081	1	0	0	IPR013103; Reverse transcriptase, RNA-dependent DNA polymerase
GlymaPAV0082	1	2	2	
GlymaPAV0083	1	1	0	
GlymaPAV0084	1	7	1	IPR001584; Integrase, catalytic core
GlymaPAV0085	0	1	0	
GlymaPAV0086	2	0	4	IPR001022; Tobamoviral movement protein
GlymaPAV0087	0	1	2	
GlymaPAV0088	1	5	4	
GlymaPAV0089	2	0	0	
GlymaPAV0090	5	10	20	IPR015416; Zinc finger, H2C2-type, histone UAS binding
GlymaPAV0091	1	2	0	
GlymaPAV0092	0	0	1	IPR013103; Reverse transcriptase, RNA-dependent DNA polymerase
GlymaPAV0093	7	29	31	
GlymaPAV0094	2	7	4	IPR001584; Integrase, catalytic core
GlymaPAV0095	5	2	6	
GlymaPAV0096	4	5	14	IPR013103; Reverse transcriptase, RNA-dependent DNA polymerase
GlymaPAV0097	0	0	4	
GlymaPAV0098	0	0	1	IPR001584; Integrase, catalytic core
GlymaPAV0099	0	2	4	
GlymaPAV0100	2	19	12	
GlymaPAV0101	5	16	20	IPR013103; Reverse transcriptase, RNA-dependent DNA polymerase
GlymaPAV0102	3	7	5	IPR001584; Integrase, catalytic core
GlymaPAV0103	0	1	2	IPR013103; Reverse transcriptase, RNA-dependent DNA polymerase
GlymaPAV0104	2	0	1	IPR013103; Reverse transcriptase, RNA-dependent DNA polymerase
GlymaPAV0105	4	1	13	
GlymaPAV0106	1	0	0	IPR001584; Integrase, catalytic core
GlymaPAV0107	2	2	0	IPR001584; Integrase, catalytic core
GlymaPAV0108	1	2	6	

GlymaPAV0109	2	11	9	IPR013103; Reverse transcriptase, RNA-dependent DNA polymerase
GlymaPAV0110	1	17	0	IPR001584; Integrase, catalytic core
GlymaPAV0111	0	5	9	IPR013103; Reverse transcriptase, RNA-dependent DNA polymerase
GlymaPAV0112	3	2	0	IPR001584; Integrase, catalytic core
GlymaPAV0113	5	0	0	IPR013103; Reverse transcriptase, RNA-dependent DNA polymerase
GlymaPAV0114	5	2	15	IPR013103; Reverse transcriptase, RNA-dependent DNA polymerase
GlymaPAV0115	4	22	10	IPR001584; Integrase, catalytic core
GlymaPAV0116	0	3	1	IPR005162; Retrotransposon gag protein
GlymaPAV0117	0	3	2	IPR013103; Reverse transcriptase, RNA-dependent DNA polymerase
GlymaPAV0118	1	3	8	
GlymaPAV0119	1	7	4	
GlymaPAV0120	0	0	1	
GlymaPAV0121	2	0	3	
GlymaPAV0122	3	4	9	IPR013103; Reverse transcriptase, RNA-dependent DNA polymerase
GlymaPAV0123	0	2	0	IPR013103; Reverse transcriptase, RNA-dependent DNA polymerase
GlymaPAV0124	1	0	2	
GlymaPAV0125	6	0	0	IPR001584; Integrase, catalytic core
GlymaPAV0126	4	10	6	
GlymaPAV0127	2	3	2	
GlymaPAV0128	4	5	15	
GlymaPAV0129	1	1	4	
GlymaPAV0130	1	0	0	IPR019557; Aminotransferase-like, plant mobile domain
GlymaPAV0131	4	0	0	
GlymaPAV0132	3	0	0	
GlymaPAV0133	0	4	3	
GlymaPAV0134	0	1	2	IPR000477; Reverse transcriptase
GlymaPAV0135	4	0	0	
GlymaPAV0136	1	4	0	
GlymaPAV0137	5	0	0	
GlymaPAV0138	1	0	3	
GlymaPAV0139	1	0	3	IPR015706; RNA-directed DNA polymerase (reverse transcriptase)
GlymaPAV0140	0	1	0	
GlymaPAV0141	0	0	5	
GlymaPAV0142	0	8	1	
GlymaPAV0143	0	0	1	
GlymaPAV0144	0	3	0	
GlymaPAV0145	0	1	1	
GlymaPAV0146	2	0	4	
GlymaPAV0147	1	0	0	
GlymaPAV0148	0	4	0	
GlymaPAV0149	3	1	2	
GlymaPAV0150	0	0	4	
GlymaPAV0151	1	5	0	
GlymaPAV0152	3	7	1	
GlymaPAV0153	3	2	1	
GlymaPAV0154	0	0	1	
GlymaPAV0155	1	0	8	
GlymaPAV0156	0	0	1	IPR001584; Integrase, catalytic core
GlymaPAV0157	0	3	0	
GlymaPAV0158	1	3	1	
GlymaPAV0159	5	0	0	
GlymaPAV0160	2	0	0	IPR015706; RNA-directed DNA polymerase (reverse transcriptase)
GlymaPAV0161	0	3	2	
GlymaPAV0162	2	0	2	
GlymaPAV0163	1	2	3	
GlymaPAV0164	0	1	2	IPR000477; Reverse transcriptase
GlymaPAV0165	0	1	1	IPR015706; RNA-directed DNA polymerase (reverse transcriptase)
GlymaPAV0166	1	0	3	

GlymaPAV0167	2	0	0	
GlymaPAV0168	2	3	0	
GlymaPAV0169	1	0	0	
GlymaPAV0170	3	6	3	IPR000504; RNA recognition motif domain
GlymaPAV0171	1	3	6	
GlymaPAV0172	0	0	1	
GlymaPAV0173	4	4	0	
GlymaPAV0174	4	15	9	
GlymaPAV0175	1	0	0	
GlymaPAV0176	1	0	2	
GlymaPAV0177	1	4	0	
GlymaPAV0178	0	4	0	
GlymaPAV0179	1	5	0	
GlymaPAV0180	4	9	12	
GlymaPAV0181	1	15	3	IPR000477; Reverse transcriptase
GlymaPAV0182	3	9	2	
GlymaPAV0183	2	1	11	
GlymaPAV0184	1	2	0	
GlymaPAV0185	3	0	3	
GlymaPAV0186	1	0	0	
GlymaPAV0187	0	0	5	
GlymaPAV0188	1	1	2	
GlymaPAV0189	0	6	2	
GlymaPAV0190	0	12	0	
GlymaPAV0191	2	6	3	
GlymaPAV0192	0	0	1	
GlymaPAV0193	1	2	1	
GlymaPAV0194	2	0	3	
GlymaPAV0195	0	1	1	
GlymaPAV0196	1	2	2	
GlymaPAV0197	3	1	3	
GlymaPAV0198	2	3	1	
GlymaPAV0199	0	4	2	
GlymaPAV0200	0	0	1	
GlymaPAV0201	1	21	2	
GlymaPAV0202	0	5	0	
GlymaPAV0203	2	0	0	
GlymaPAV0204	1	0	0	
GlymaPAV0205	0	0	4	IPR015706; RNA-directed DNA polymerase (reverse transcriptase)
GlymaPAV0206	2	1	1	
GlymaPAV0207	5	2	2	
GlymaPAV0208	5	2	4	
GlymaPAV0209	6	0	4	
GlymaPAV0210	2	0	2	
GlymaPAV0211	1	1	0	
GlymaPAV0212	0	0	3	
GlymaPAV0213	3	0	0	IPR001878; Zinc finger, CCHC-type
GlymaPAV0214	0	7	0	
GlymaPAV0215	3	3	1	
GlymaPAV0216	0	1	2	
GlymaPAV0217	0	1	2	
GlymaPAV0218	1	2	0	
GlymaPAV0219	4	0	2	
GlymaPAV0220	7	1	3	
GlymaPAV0221	0	2	1	
GlymaPAV0222	2	3	5	
GlymaPAV0223	2	0	2	
GlymaPAV0224	1	1	1	

GlymaPAV0225	1	1	7	
GlymaPAV0226	0	0	1	
GlymaPAV0227	0	0	2	
GlymaPAV0228	0	3	10	
GlymaPAV0229	1	0	0	
GlymaPAV0230	1	0	2	
GlymaPAV0231	0	1	2	
GlymaPAV0232	0	1	4	
GlymaPAV0233	5	6	1	
GlymaPAV0234	1	10	5	
GlymaPAV0235	0	0	2	
GlymaPAV0236	3	1	2	
GlymaPAV0237	0	0	1	
GlymaPAV0238	2	0	0	
GlymaPAV0239	1	0	1	
GlymaPAV0240	4	1	11	
GlymaPAV0241	2	0	2	
GlymaPAV0242	6	2	5	
GlymaPAV0243	3	0	1	
GlymaPAV0244	0	1	0	
GlymaPAV0245	0	1	0	
GlymaPAV0246	1	1	4	
GlymaPAV0247	2	14	0	
GlymaPAV0248	0	0	4	
GlymaPAV0249	0	1	5	
GlymaPAV0250	8	1	3	
GlymaPAV0251	3	0	0	
GlymaPAV0252	4	5	19	
GlymaPAV0253	0	3	5	
GlymaPAV0254	0	1	0	
GlymaPAV0255	1	2	1	
GlymaPAV0256	2	10	9	IPR018150; Aminoacyl-tRNA synthetase, class II (D/K/N)-like
GlymaPAV0257	5	15	27	
GlymaPAV0258	2	0	0	
GlymaPAV0259	0	2	2	
GlymaPAV0260	4	0	0	
GlymaPAV0261	1	1	8	
GlymaPAV0262	0	2	0	
GlymaPAV0263	0	0	2	
GlymaPAV0264	1	0	0	
GlymaPAV0265	1	1	4	
GlymaPAV0266	0	4	0	
GlymaPAV0267	5	2	1	
GlymaPAV0268	0	1	1	
GlymaPAV0269	0	3	0	
GlymaPAV0270	1	1	2	
GlymaPAV0271	0	7	1	
GlymaPAV0272	1	3	9	
GlymaPAV0273	1	0	0	IPR001878; Zinc finger, CCHC-type
GlymaPAV0274	2	2	6	IPR019557; Aminotransferase-like, plant mobile domain
GlymaPAV0275	5	1	6	
GlymaPAV0276	0	1	2	
GlymaPAV0277	2	5	1	
GlymaPAV0278	0	1	1	
GlymaPAV0279	6	13	6	
GlymaPAV0280	1	0	2	
GlymaPAV0281	3	3	3	
GlymaPAV0282	0	6	0	

GlymaPAV0283	1	2	6	
GlymaPAV0284	0	0	1	IPR004158; Protein of unknown function DUF247, plant
GlymaPAV0285	0	0	1	
GlymaPAV0286	0	0	1	
GlymaPAV0287	0	5	2	
GlymaPAV0288	0	1	1	
GlymaPAV0289	2	0	0	
GlymaPAV0290	2	1	2	
GlymaPAV0291	1	2	0	IPR000953; Chromo domain
GlymaPAV0292	1	13	11	
GlymaPAV0293	1	2	2	IPR008906; HAT dimerisation
GlymaPAV0294	0	0	1	
GlymaPAV0295	3	4	2	
GlymaPAV0296	1	0	1	IPR000782; FAS1 domain
GlymaPAV0297	0	2	0	
GlymaPAV0298	2	5	10	
GlymaPAV0299	2	13	8	IPR015706; RNA-directed DNA polymerase (reverse transcriptase)
GlymaPAV0300	5	5	9	IPR001611; Leucine-rich repeat IPR013210; Leucine-rich repeat-containing N-terminal, type 2
GlymaPAV0301	0	0	3	
GlymaPAV0302	4	3	0	
GlymaPAV0303	4	6	3	
GlymaPAV0304	7	26	40	IPR013242; Retroviral aspartyl protease
GlymaPAV0305	2	3	0	
GlymaPAV0306	4	16	7	
GlymaPAV0307	3	0	0	
GlymaPAV0308	0	0	2	
GlymaPAV0309	1	7	3	
GlymaPAV0310	0	0	2	
GlymaPAV0311	2	1	0	
GlymaPAV0312	5	18	20	
GlymaPAV0313	2	1	2	
GlymaPAV0314	1	1	2	
GlymaPAV0315	4	2	2	
GlymaPAV0316	0	0	1	
GlymaPAV0317	1	2	1	
GlymaPAV0318	2	2	6	IPR019557; Aminotransferase-like, plant mobile domain
GlymaPAV0319	1	2	0	
GlymaPAV0320	3	2	1	
GlymaPAV0321	2	1	0	
GlymaPAV0322	2	1	0	
GlymaPAV0323	1	0	2	
GlymaPAV0324	0	2	0	
GlymaPAV0325	2	7	13	
GlymaPAV0326	0	0	5	
GlymaPAV0327	2	3	6	
GlymaPAV0328	1	0	2	
GlymaPAV0329	5	4	1	
GlymaPAV0330	0	0	2	
GlymaPAV0331	1	0	1	
GlymaPAV0332	2	8	4	
GlymaPAV0333	1	1	0	
GlymaPAV0334	0	1	2	
GlymaPAV0335	0	0	1	
GlymaPAV0336	1	2	0	IPR002100; Transcription factor, MADS-box
GlymaPAV0337	1	0	0	
GlymaPAV0338	3	3	2	
GlymaPAV0339	1	0	0	
GlymaPAV0340	0	4	0	

GlymaPAV0341	1	0	1	
GlymaPAV0342	4	0	0	
GlymaPAV0343	0	4	2	
GlymaPAV0344	4	2	0	
GlymaPAV0345	1	10	4	
GlymaPAV0346	2	0	1	
GlymaPAV0347	1	2	8	IPR000477; Reverse transcriptase
GlymaPAV0348	0	2	0	
GlymaPAV0349	0	0	1	
GlymaPAV0350	1	2	15	
GlymaPAV0351	5	20	4	
GlymaPAV0352	0	4	9	
GlymaPAV0353	4	0	1	
GlymaPAV0354	3	3	0	
GlymaPAV0355	0	2	0	
GlymaPAV0356	1	3	2	
GlymaPAV0357	1	8	4	
GlymaPAV0358	2	0	1	
GlymaPAV0359	1	0	6	
GlymaPAV0360	1	4	3	
GlymaPAV0361	3	1	0	
GlymaPAV0362	2	0	0	
GlymaPAV0363	0	11	2	
GlymaPAV0364	3	2	11	
GlymaPAV0365	0	1	0	
GlymaPAV0366	5	6	0	
GlymaPAV0367	0	2	1	
GlymaPAV0368	0	12	4	
GlymaPAV0369	1	1	9	
GlymaPAV0370	3	2	9	IPR000953; Chromo domain
GlymaPAV0371	0	3	0	
GlymaPAV0372	2	4	1	
GlymaPAV0373	1	11	0	
GlymaPAV0374	5	7	5	
GlymaPAV0375	0	0	2	
GlymaPAV0376	2	0	0	
GlymaPAV0377	0	3	0	IPR000953; Chromo domain
GlymaPAV0378	6	10	14	
GlymaPAV0379	1	0	0	
GlymaPAV0380	5	5	28	IPR000953; Chromo domain
GlymaPAV0381	1	4	2	
GlymaPAV0382	1	0	0	
GlymaPAV0383	0	2	1	
GlymaPAV0384	5	2	9	
GlymaPAV0385	2	5	7	
GlymaPAV0386	0	1	1	IPR005135; Endonuclease/exonuclease/phosphatase
GlymaPAV0387	1	2	2	IPR015706; RNA-directed DNA polymerase (reverse transcriptase)
GlymaPAV0388	1	1	2	
GlymaPAV0389	2	0	1	
GlymaPAV0390	2	1	6	
GlymaPAV0391	2	20	10	
GlymaPAV0392	2	0	1	
GlymaPAV0393	0	1	0	
GlymaPAV0394	3	0	0	
GlymaPAV0395	0	0	2	IPR001878; Zinc finger, CCHC-type
GlymaPAV0396	1	4	9	IPR018150; Aminoacyl-tRNA synthetase, class II (D/K/N)-like
GlymaPAV0397	0	1	2	
GlymaPAV0398	2	2	8	

GlymaPAV0399	0	0	6	
GlymaPAV0400	2	0	0	
GlymaPAV0401	0	6	16	
GlymaPAV0402	7	22	20	
GlymaPAV0403	2	1	2	
GlymaPAV0404	1	0	0	
GlymaPAV0405	0	1	0	
GlymaPAV0406	4	1	1	
GlymaPAV0407	0	1	2	
GlymaPAV0408	3	5	3	IPR013242; Retroviral aspartyl protease
GlymaPAV0409	3	0	3	
GlymaPAV0410	0	2	0	
GlymaPAV0411	2	1	0	
GlymaPAV0412	2	0	0	
GlymaPAV0413	2	2	1	
GlymaPAV0414	2	3	7	
GlymaPAV0415	0	0	1	
GlymaPAV0416	4	0	0	
GlymaPAV0417	4	0	0	
GlymaPAV0418	1	10	1	
GlymaPAV0419	1	1	0	
GlymaPAV0420	1	0	0	
GlymaPAV0421	0	0	1	
GlymaPAV0422	0	0	8	
GlymaPAV0423	0	2	1	
GlymaPAV0424	1	0	0	
GlymaPAV0425	3	3	4	
GlymaPAV0426	9	3	1	IPR015706; RNA-directed DNA polymerase (reverse transcriptase)
GlymaPAV0427	1	5	0	
GlymaPAV0428	6	2	1	
GlymaPAV0429	3	1	2	
GlymaPAV0430	0	1	8	
GlymaPAV0431	2	1	5	
GlymaPAV0432	0	6	3	
GlymaPAV0433	1	2	4	
GlymaPAV0434	2	2	2	IPR019557; Aminotransferase-like, plant mobile domain
GlymaPAV0435	0	1	0	
GlymaPAV0436	5	13	12	
GlymaPAV0437	8	15	8	
GlymaPAV0438	2	1	0	
GlymaPAV0439	1	0	0	
GlymaPAV0440	0	6	0	
GlymaPAV0441	0	3	8	
GlymaPAV0442	2	2	0	
GlymaPAV0443	0	0	1	
GlymaPAV0444	2	1	3	
GlymaPAV0445	1	1	3	
GlymaPAV0446	6	2	1	
GlymaPAV0447	1	9	1	
GlymaPAV0448	2	6	12	
GlymaPAV0449	1	2	1	
GlymaPAV0450	0	0	1	IPR013242; Retroviral aspartyl protease
GlymaPAV0451	1	1	5	
GlymaPAV0452	6	2	1	
GlymaPAV0453	4	0	1	
GlymaPAV0454	0	0	1	IPR018061; Peptidase A2A, retrovirus RVP subgroup
GlymaPAV0455	2	2	0	
GlymaPAV0456	1	12	3	

GlymaPAV0457	2	4	2	IPR005162; Retrotransposon gag protein
GlymaPAV0458	1	3	5	
GlymaPAV0459	2	1	0	
GlymaPAV0460	1	4	0	
GlymaPAV0461	0	3	1	
GlymaPAV0462	3	0	1	
GlymaPAV0463	1	0	1	
GlymaPAV0464	5	12	1	
GlymaPAV0465	2	0	0	
GlymaPAV0466	1	0	0	
GlymaPAV0467	1	7	0	
GlymaPAV0468	3	2	0	
GlymaPAV0469	0	1	2	
GlymaPAV0470	1	1	9	
GlymaPAV0471	2	2	4	
GlymaPAV0472	0	2	1	
GlymaPAV0473	0	0	3	
GlymaPAV0474	0	3	0	
GlymaPAV0475	6	3	6	
GlymaPAV0476	1	0	0	
GlymaPAV0477	2	2	0	
GlymaPAV0478	0	1	7	
GlymaPAV0479	2	0	0	
GlymaPAV0480	0	2	0	
GlymaPAV0481	1	0	7	
GlymaPAV0482	6	5	0	
GlymaPAV0483	5	3	3	IPR002156; Ribonuclease H domain
GlymaPAV0484	2	0	0	
GlymaPAV0485	0	2	2	
GlymaPAV0486	0	0	2	
GlymaPAV0487	1	0	0	
GlymaPAV0488	1	0	0	
GlymaPAV0489	0	1	1	IPR005162; Retrotransposon gag protein
GlymaPAV0490	0	6	1	
GlymaPAV0491	0	5	2	
GlymaPAV0492	2	0	12	
GlymaPAV0493	7	1	10	
GlymaPAV0494	0	2	0	
GlymaPAV0495	0	3	0	IPR019557; Aminotransferase-like, plant mobile domain
GlymaPAV0496	4	0	0	
GlymaPAV0497	0	6	0	
GlymaPAV0498	2	3	0	
GlymaPAV0499	0	1	2	
GlymaPAV0500	6	7	21	IPR013242; Retroviral aspartyl protease IPR018150; Aminoacyl-tRNA synthetase, class II (D/K/N)-like
GlymaPAV0501	2	3	6	
GlymaPAV0502	0	2	1	IPR018150; Aminoacyl-tRNA synthetase, class II (D/K/N)-like
GlymaPAV0503	2	3	1	
GlymaPAV0504	1	7	0	
GlymaPAV0505	5	0	0	
GlymaPAV0506	8	5	6	IPR019103; Peptidase aspartic, eukaryotic predicted
GlymaPAV0507	1	3	0	IPR015416; Zinc finger, H2C2-type, histone UAS binding
GlymaPAV0508	4	4	9	
GlymaPAV0509	5	0	7	IPR015706; RNA-directed DNA polymerase (reverse transcriptase)
GlymaPAV0510	2	11	5	
GlymaPAV0511	2	2	3	
GlymaPAV0512	3	1	1	
GlymaPAV0513	0	0	1	
GlymaPAV0514	1	4	0	

GlymaPAV0515	5	4	1	
GlymaPAV0516	1	1	0	
GlymaPAV0517	2	7	1	
GlymaPAV0518	2	0	1	
GlymaPAV0519	0	2	10	
GlymaPAV0520	6	3	2	IPR000477; Reverse transcriptase
GlymaPAV0521	3	3	2	IPR005162; Retrotransposon gag protein
GlymaPAV0522	2	4	2	
GlymaPAV0523	3	5	0	
GlymaPAV0524	1	5	3	
GlymaPAV0525	2	3	4	
GlymaPAV0526	0	1	0	
GlymaPAV0527	1	0	0	
GlymaPAV0528	0	1	6	
GlymaPAV0529	2	0	1	
GlymaPAV0530	5	0	6	
GlymaPAV0531	1	0	0	
GlymaPAV0532	10	5	10	
GlymaPAV0533	1	0	0	
GlymaPAV0534	1	2	2	
GlymaPAV0535	0	0	1	
GlymaPAV0536	1	3	0	
GlymaPAV0537	2	0	1	
GlymaPAV0538	2	5	0	
GlymaPAV0539	1	1	5	
GlymaPAV0540	1	1	0	
GlymaPAV0541	5	1	4	
GlymaPAV0542	4	7	4	IPR001878; Zinc finger, CCHC-type IPR013242; Retroviral aspartyl protease
GlymaPAV0543	1	1	1	
GlymaPAV0544	2	1	8	
GlymaPAV0545	0	1	1	
GlymaPAV0546	1	7	0	
GlymaPAV0547	3	3	0	
GlymaPAV0548	0	14	3	IPR015706; RNA-directed DNA polymerase (reverse transcriptase)
GlymaPAV0549	1	0	1	
GlymaPAV0550	0	1	0	
GlymaPAV0551	3	4	0	IPR015416; Zinc finger, H2C2-type, histone UAS binding
GlymaPAV0552	0	3	4	
GlymaPAV0553	0	0	4	IPR001878; Zinc finger, CCHC-type
GlymaPAV0554	1	5	5	
GlymaPAV0555	1	0	6	
GlymaPAV0556	1	0	0	IPR015706; RNA-directed DNA polymerase (reverse transcriptase)
GlymaPAV0557	2	4	0	
GlymaPAV0558	0	0	1	
GlymaPAV0559	2	4	2	
GlymaPAV0560	0	0	1	IPR001750; NADH:ubiquinone/plastoquinone oxidoreductase IPR003918; NADH:ubiquinone oxidoreductase
GlymaPAV0561	0	2	0	
GlymaPAV0562	0	1	0	
GlymaPAV0563	0	0	1	
GlymaPAV0564	1	0	1	
GlymaPAV0565	0	2	1	
GlymaPAV0566	0	2	0	
GlymaPAV0567	7	0	0	
GlymaPAV0568	1	3	1	
GlymaPAV0569	1	1	4	
GlymaPAV0570	1	0	0	
GlymaPAV0571	0	2	1	
GlymaPAV0572	0	1	4	

GlymaPAV0573	2	0	0	
GlymaPAV0574	0	0	1	
GlymaPAV0575	3	2	1	
GlymaPAV0576	1	8	1	IPR013103; Reverse transcriptase, RNA-dependent DNA polymerase
GlymaPAV0577	0	4	0	
GlymaPAV0578	0	1	16	
GlymaPAV0579	0	6	2	IPR001584; Integrase, catalytic core
GlymaPAV0580	2	1	0	
GlymaPAV0581	0	0	1	
GlymaPAV0582	6	7	1	IPR005162; Retrotransposon gag protein
GlymaPAV0583	1	4	0	
GlymaPAV0584	0	2	4	
GlymaPAV0585	2	1	11	
GlymaPAV0586	0	3	3	IPR000477; Reverse transcriptase IPR015706; RNA-directed DNA polymerase (reverse transcriptase)
GlymaPAV0587	1	0	3	
GlymaPAV0588	2	1	0	
GlymaPAV0589	0	1	2	IPR000477; Reverse transcriptase IPR015706; RNA-directed DNA polymerase (reverse transcriptase)
GlymaPAV0590	0	0	2	
GlymaPAV0591	1	0	0	IPR018289; MULE transposase, conserved domain
GlymaPAV0592	0	0	6	IPR013103; Reverse transcriptase, RNA-dependent DNA polymerase
GlymaPAV0593	0	1	0	IPR005481; Carbamoyl-phosphate synthase, large subunit, N-terminal
GlymaPAV0594	1	3	0	
GlymaPAV0595	2	0	0	IPR001611; Leucine-rich repeat
GlymaPAV0596	1	15	1	
GlymaPAV0597	4	10	23	IPR001584; Integrase, catalytic core
GlymaPAV0598	4	3	11	IPR013242; Retroviral aspartyl protease
GlymaPAV0599	2	0	0	IPR004242; Transposon, En/Spm-like
GlymaPAV0600	4	14	17	IPR000477; Reverse transcriptase
GlymaPAV0601	1	15	5	IPR000477; Reverse transcriptase
GlymaPAV0602	0	1	0	IPR001789; Signal transduction response regulator, receiver domain
GlymaPAV0603	0	0	1	IPR001584; Integrase, catalytic core
GlymaPAV0604	0	1	0	IPR001123; Lysine exporter protein (LYSE/YGGA)
GlymaPAV0605	0	1	0	IPR001750; NADH:ubiquinone/plastoquinone oxidoreductase
GlymaPAV0606	0	0	7	
GlymaPAV0607	0	1	0	IPR004114; THUMP IPR020536; Thiamine biosynthesis protein, C-terminal
GlymaPAV0608	2	2	2	IPR000298; Cytochrome c oxidase, subunit III
GlymaPAV0609	0	0	4	
GlymaPAV0610	2	0	0	
GlymaPAV0611	0	1	0	
GlymaPAV0612	7	18	14	IPR005162; Retrotransposon gag protein
GlymaPAV0613	4	6	1	
GlymaPAV0614	2	2	2	IPR001750; NADH:ubiquinone/plastoquinone oxidoreductase
GlymaPAV0615	2	0	3	
GlymaPAV0616	1	4	5	
GlymaPAV0617	1	7	0	IPR007021; Domain of unknown function DUF659
GlymaPAV0618	1	0	1	IPR013103; Reverse transcriptase, RNA-dependent DNA polymerase
GlymaPAV0619	3	17	40	
GlymaPAV0620	3	0	0	IPR001584; Integrase, catalytic core
GlymaPAV0621	6	15	2	IPR001584; Integrase, catalytic core
GlymaPAV0622	3	0	0	IPR005162; Retrotransposon gag protein
GlymaPAV0623	5	0	0	IPR013103; Reverse transcriptase, RNA-dependent DNA polymerase
GlymaPAV0624	1	0	0	
GlymaPAV0625	8	12	30	IPR001584; Integrase, catalytic core
GlymaPAV0626	0	0	1	IPR000477; Reverse transcriptase IPR015706; RNA-directed DNA polymerase (reverse transcriptase)
GlymaPAV0627	1	6	1	IPR001584; Integrase, catalytic core
GlymaPAV0628	0	8	1	IPR004000; Actin-like IPR015623; Actin-related protein 3
GlymaPAV0629	1	1	1	
GlymaPAV0630	0	2	3	IPR004242; Transposon, En/Spm-like

GlymaPAV0631	1	0	2	
GlymaPAV0632	4	1	0	
GlymaPAV0633	2	1	1	
GlymaPAV0634	0	0	1	IPR013103; Reverse transcriptase, RNA-dependent DNA polymerase
GlymaPAV0635	1	2	0	IPR000477; Reverse transcriptase IPR015706; RNA-directed DNA polymerase (reverse transcriptase)
GlymaPAV0636	0	4	0	IPR004242; Transposon, En/Spm-like
GlymaPAV0637	0	2	9	IPR000477; Reverse transcriptase IPR015706; RNA-directed DNA polymerase (reverse transcriptase)
GlymaPAV0638	1	4	0	IPR000477; Reverse transcriptase
GlymaPAV0639	4	4	5	IPR001584; Integrase, catalytic core
GlymaPAV0640	2	4	15	
GlymaPAV0641	2	0	0	
GlymaPAV0642	3	12	20	
GlymaPAV0643	4	2	4	IPR000477; Reverse transcriptase
GlymaPAV0644	0	0	5	
GlymaPAV0645	2	0	1	IPR005135; Endonuclease/exonuclease/phosphatase IPR015706; RNA-directed DNA polymerase (reverse transcriptase)
GlymaPAV0646	10	40	56	
GlymaPAV0647	1	0	0	
GlymaPAV0648	5	19	7	
GlymaPAV0649	6	3	2	
GlymaPAV0650	0	0	4	IPR000477; Reverse transcriptase IPR015706; RNA-directed DNA polymerase (reverse transcriptase)
GlymaPAV0651	1	0	0	IPR005135; Endonuclease/exonuclease/phosphatase IPR015706; RNA-directed DNA polymerase (reverse transcriptase)
GlymaPAV0652	4	1	4	
GlymaPAV0653	0	1	0	IPR001623; Heat shock protein DnaJ, N-terminal IPR003095; Heat shock protein DnaJ IPR015609; Molecular chaperone, heat shock protein, Hsp40, DnaJ IPR018253; Heat shock protein DnaJ, conserved site
GlymaPAV0654	0	3	1	IPR001584; Integrase, catalytic core
GlymaPAV0655	0	1	2	IPR001584; Integrase, catalytic core
GlymaPAV0656	0	1	2	
GlymaPAV0657	2	6	5	IPR019557; Aminotransferase-like, plant mobile domain
GlymaPAV0658	2	11	11	IPR000477; Reverse transcriptase
GlymaPAV0659	3	1	1	
GlymaPAV0660	3	12	4	IPR001584; Integrase, catalytic core
GlymaPAV0661	3	4	3	IPR001611; Leucine-rich repeat IPR003591; Leucine-rich repeat, typical subtype
GlymaPAV0662	0	1	0	
GlymaPAV0663	0	9	0	
GlymaPAV0664	4	1	13	IPR000767; Disease resistance protein IPR001611; Leucine-rich repeat IPR002182; NB-ARC
GlymaPAV0665	2	5	5	
GlymaPAV0666	5	2	5	
GlymaPAV0667	0	0	2	
GlymaPAV0668	0	0	1	
GlymaPAV0669	0	1	3	
GlymaPAV0670	3	6	10	IPR000453; Chorismate synthase
GlymaPAV0671	0	1	0	
GlymaPAV0672	1	0	0	IPR013955; Replication factor A, C-terminal
GlymaPAV0673	1	1	1	
GlymaPAV0674	0	5	0	
GlymaPAV0675	2	1	0	
GlymaPAV0676	2	0	4	
GlymaPAV0677	1	0	0	
GlymaPAV0678	1	0	0	
GlymaPAV0679	0	0	1	
GlymaPAV0680	0	1	0	
GlymaPAV0681	0	0	1	
GlymaPAV0682	2	6	3	
GlymaPAV0683	0	2	0	IPR003323; Ovarian tumour, otubain
GlymaPAV0684	0	3	5	
GlymaPAV0685	0	1	0	

GlymaPAV0686	4	16	19	
GlymaPAV0687	1	0	3	IPR003871; Domain of unknown function DUF223
GlymaPAV0688	4	6	2	
GlymaPAV0689	1	0	1	IPR015706; RNA-directed DNA polymerase (reverse transcriptase)
GlymaPAV0690	1	1	0	IPR003323; Ovarian tumour, otubain
GlymaPAV0691	1	0	4	IPR015706; RNA-directed DNA polymerase (reverse transcriptase)
GlymaPAV0692	0	1	4	
GlymaPAV0693	1	1	3	
GlymaPAV0694	1	2	0	
GlymaPAV0695	0	1	3	
GlymaPAV0696	1	3	4	
GlymaPAV0697	1	3	12	
GlymaPAV0698	1	0	1	
GlymaPAV0699	0	3	10	IPR005162; Retrotransposon gag protein
GlymaPAV0700	1	3	7	
GlymaPAV0701	1	0	0	
GlymaPAV0702	1	0	0	IPR015706; RNA-directed DNA polymerase (reverse transcriptase)
GlymaPAV0703	4	9	8	
GlymaPAV0704	5	0	0	
GlymaPAV0705	2	0	0	IPR001480; Bulb-type lectin domain
GlymaPAV0706	4	1	8	
GlymaPAV0707	2	1	0	
GlymaPAV0708	0	0	2	IPR007644; RNA polymerase, beta subunit, protrusion
GlymaPAV0709	0	2	0	
GlymaPAV0710	0	6	0	
GlymaPAV0711	1	5	2	
GlymaPAV0712	0	1	1	
GlymaPAV0713	3	10	0	
GlymaPAV0714	3	3	0	
GlymaPAV0715	1	8	9	
GlymaPAV0716	2	6	4	
GlymaPAV0717	0	1	0	
GlymaPAV0718	2	7	7	
GlymaPAV0719	0	1	2	
GlymaPAV0720	0	1	0	
GlymaPAV0721	1	0	2	IPR004875; DDE superfamily endonuclease, CENP-B-like IPR004906; Pogo transposase / Cenp-B / PDC2, subgroup, DNA-binding HTH domain IPR006600; Pogo transposase / Cenp-B / PDC2, DNA-binding HTH domain
GlymaPAV0722	3	0	1	IPR003656; Zinc finger, BED-type predicted
GlymaPAV0723	2	0	3	
GlymaPAV0724	1	7	0	
GlymaPAV0725	2	2	15	
GlymaPAV0726	4	0	0	
GlymaPAV0727	1	2	0	
GlymaPAV0728	0	3	0	
GlymaPAV0729	3	0	2	
GlymaPAV0730	2	0	0	IPR015706; RNA-directed DNA polymerase (reverse transcriptase)
GlymaPAV0731	4	1	0	
GlymaPAV0732	0	4	5	IPR001878; Zinc finger, CCHC-type IPR005162; Retrotransposon gag protein IPR013242; Retroviral aspartyl protease
GlymaPAV0733	4	1	0	
GlymaPAV0734	1	7	5	
GlymaPAV0735	1	1	2	
GlymaPAV0736	7	2	1	IPR005162; Retrotransposon gag protein
GlymaPAV0737	1	0	1	IPR006912; Putative harbinger transposase-derived nuclease
GlymaPAV0738	1	11	0	IPR004158; Protein of unknown function DUF247, plant
GlymaPAV0739	1	0	0	
GlymaPAV0740	0	0	1	
GlymaPAV0741	2	11	12	

GlymaPAV0742	1	2	2	IPR013148; Glycosyl hydrolases family 32, N-terminal
GlymaPAV0743	0	2	2	IPR005162; Retrotransposon gag protein
GlymaPAV0744	5	13	12	
GlymaPAV0745	0	1	0	
GlymaPAV0746	0	0	2	IPR000814; TATA-box binding protein
GlymaPAV0747	4	3	3	IPR000477; Reverse transcriptase
GlymaPAV0748	0	3	0	IPR001878; Zinc finger, CCHC-type
GlymaPAV0749	3	0	0	
GlymaPAV0750	0	1	0	IPR005162; Retrotransposon gag protein
GlymaPAV0751	2	2	3	
GlymaPAV0752	0	2	6	
GlymaPAV0753	1	0	0	
GlymaPAV0754	0	1	0	IPR013103; Reverse transcriptase, RNA-dependent DNA polymerase
GlymaPAV0755	1	0	0	
GlymaPAV0756	3	10	1	IPR001584; Integrase, catalytic core
GlymaPAV0757	6	15	18	IPR015706; RNA-directed DNA polymerase (reverse transcriptase)
GlymaPAV0758	1	6	4	
GlymaPAV0759	1	0	0	
GlymaPAV0760	0	3	1	IPR005162; Retrotransposon gag protein
GlymaPAV0761	0	0	1	IPR013103; Reverse transcriptase, RNA-dependent DNA polymerase
GlymaPAV0762	2	1	4	
GlymaPAV0763	0	3	3	IPR002885; Pentatricopeptide repeat
GlymaPAV0764	3	6	3	IPR013103; Reverse transcriptase, RNA-dependent DNA polymerase
GlymaPAV0765	1	1	1	IPR000477; Reverse transcriptase
GlymaPAV0766	2	0	0	IPR015706; RNA-directed DNA polymerase (reverse transcriptase)
GlymaPAV0767	1	19	2	
GlymaPAV0768	5	14	16	IPR013103; Reverse transcriptase, RNA-dependent DNA polymerase
GlymaPAV0769	2	4	3	IPR000477; Reverse transcriptase
GlymaPAV0770	0	17	1	
GlymaPAV0771	5	3	23	IPR002156; Ribonuclease H domain
GlymaPAV0772	0	1	5	IPR008906; HAT dimerisation
GlymaPAV0773	1	12	12	IPR005162; Retrotransposon gag protein
GlymaPAV0774	2	0	2	
GlymaPAV0775	5	2	0	IPR013103; Reverse transcriptase, RNA-dependent DNA polymerase
GlymaPAV0776	0	5	21	IPR013103; Reverse transcriptase, RNA-dependent DNA polymerase
GlymaPAV0777	4	36	38	IPR013103; Reverse transcriptase, RNA-dependent DNA polymerase
GlymaPAV0778	1	8	4	
GlymaPAV0779	1	5	0	IPR005162; Retrotransposon gag protein
GlymaPAV0780	0	0	1	
GlymaPAV0781	5	10	20	IPR001584; Integrase, catalytic core IPR015416; Zinc finger, H2C2-type, histone UAS binding
GlymaPAV0782	4	3	4	
GlymaPAV0783	2	2	2	IPR000477; Reverse transcriptase IPR015706; RNA-directed DNA polymerase (reverse transcriptase)
GlymaPAV0784	0	1	0	
GlymaPAV0785	2	2	3	IPR006527; F-box associated domain, type 1
GlymaPAV0786	0	4	0	
GlymaPAV0787	0	4	5	IPR000477; Reverse transcriptase IPR015706; RNA-directed DNA polymerase (reverse transcriptase)
GlymaPAV0788	0	1	0	
GlymaPAV0789	8	16	8	IPR000477; Reverse transcriptase IPR013242; Retroviral aspartyl protease
GlymaPAV0790	1	0	4	
GlymaPAV0791	3	0	0	IPR015706; RNA-directed DNA polymerase (reverse transcriptase)
GlymaPAV0792	1	1	0	
GlymaPAV0793	0	1	2	
GlymaPAV0794	3	2	6	IPR000477; Reverse transcriptase
GlymaPAV0795	0	13	13	
GlymaPAV0796	0	0	1	
GlymaPAV0797	0	0	1	
GlymaPAV0798	0	1	1	IPR001584; Integrase, catalytic core
GlymaPAV0799	4	4	1	IPR002156; Ribonuclease H domain

GlymaPAV0800	3	5	1	IPR000477; Reverse transcriptase
GlymaPAV0801	1	7	0	
GlymaPAV0802	4	1	4	IPR001584; Integrase, catalytic core
GlymaPAV0803	3	1	0	IPR000477; Reverse transcriptase
GlymaPAV0804	1	1	4	
GlymaPAV0805	2	0	0	
GlymaPAV0806	0	2	0	
GlymaPAV0807	2	0	1	
GlymaPAV0808	2	2	4	
GlymaPAV0809	0	8	11	
GlymaPAV0810	0	0	3	IPR000477; Reverse transcriptase IPR015706; RNA-directed DNA polymerase (reverse transcriptase)
GlymaPAV0811	0	2	0	IPR013103; Reverse transcriptase, RNA-dependent DNA polymerase
GlymaPAV0812	2	0	0	IPR010285; DNA helicase PIF1, ATP-dependent
GlymaPAV0813	1	7	2	
GlymaPAV0814	5	6	13	IPR001584; Integrase, catalytic core
GlymaPAV0815	3	0	0	IPR001584; Integrase, catalytic core
GlymaPAV0816	0	3	0	
GlymaPAV0817	2	0	2	IPR000999; Ribonuclease III
GlymaPAV0818	1	2	0	IPR006564; Zinc finger, PMZ-type IPR007527; Zinc finger, SWIM-type
GlymaPAV0819	1	1	9	IPR019557; Aminotransferase-like, plant mobile domain
GlymaPAV0820	1	2	2	IPR001878; Zinc finger, CCHC-type
GlymaPAV0821	1	0	2	IPR000477; Reverse transcriptase
GlymaPAV0822	0	1	1	
GlymaPAV0823	4	3	1	
GlymaPAV0824	4	8	3	IPR000477; Reverse transcriptase
GlymaPAV0825	1	4	10	IPR015416; Zinc finger, H2C2-type, histone UAS binding
GlymaPAV0826	2	5	4	
GlymaPAV0827	2	0	2	
GlymaPAV0828	1	0	2	IPR001584; Integrase, catalytic core
GlymaPAV0829	1	2	3	IPR000477; Reverse transcriptase
GlymaPAV0830	0	1	0	
GlymaPAV0831	0	1	2	
GlymaPAV0832	0	0	1	IPR001128; Cytochrome P450 IPR002401; Cytochrome P450, E-class, group I IPR017972; Cytochrome P450, conserved site
GlymaPAV0833	5	0	0	
GlymaPAV0834	1	0	2	IPR001128; Cytochrome P450 IPR002401; Cytochrome P450, E-class, group I IPR017972; Cytochrome P450, conserved site
GlymaPAV0835	7	2	5	IPR001584; Integrase, catalytic core
GlymaPAV0836	4	0	1	IPR002761; Domain of unknown function DUF71, ATP-binding domain
GlymaPAV0837	1	0	0	IPR000477; Reverse transcriptase IPR015706; RNA-directed DNA polymerase (reverse transcriptase)
GlymaPAV0838	1	2	0	IPR000477; Reverse transcriptase IPR015706; RNA-directed DNA polymerase (reverse transcriptase)
GlymaPAV0839	0	1	2	
GlymaPAV0840	1	13	3	IPR000477; Reverse transcriptase IPR015706; RNA-directed DNA polymerase (reverse transcriptase)
GlymaPAV0841	1	0	0	IPR005135; Endonuclease/exonuclease/phosphatase
GlymaPAV0842	0	1	0	
GlymaPAV0843	1	15	2	
GlymaPAV0844	0	7	4	
GlymaPAV0845	2	2	8	
GlymaPAV0846	1	0	3	
GlymaPAV0847	0	1	0	IPR002068; Heat shock protein Hsp20
GlymaPAV0848	1	2	0	IPR015706; RNA-directed DNA polymerase (reverse transcriptase)
GlymaPAV0849	3	10	1	IPR000722; RNA polymerase, alpha subunit
GlymaPAV0850	1	13	8	
GlymaPAV0851	0	0	1	IPR019557; Aminotransferase-like, plant mobile domain
GlymaPAV0852	1	5	2	IPR013103; Reverse transcriptase, RNA-dependent DNA polymerase
GlymaPAV0853	5	32	31	
GlymaPAV0854	1	2	1	
GlymaPAV0855	1	3	2	

GlymaPAV0856	1	1	9	IPR001878; Zinc finger, CCHC-type
GlymaPAV0857	1	0	0	
GlymaPAV0858	0	0	4	IPR000719; Protein kinase, catalytic domain IPR017442; Serine/threonine-protein kinase-like domain
GlymaPAV0859	0	2	0	
GlymaPAV0860	2	2	2	IPR001878; Zinc finger, CCHC-type
GlymaPAV0861	1	0	3	IPR002198; Short-chain dehydrogenase/reductase SDR
GlymaPAV0862	1	8	9	
GlymaPAV0863	5	2	5	
GlymaPAV0864	2	0	0	
GlymaPAV0865	0	0	1	
GlymaPAV0866	0	0	1	
GlymaPAV0867	1	1	0	IPR003377; Cornichon
GlymaPAV0868	1	1	0	IPR001128; Cytochrome P450
GlymaPAV0869	1	2	0	
GlymaPAV0870	0	2	1	
GlymaPAV0871	1	0	0	
GlymaPAV0872	1	4	0	
GlymaPAV0873	2	2	1	
GlymaPAV0874	1	20	2	IPR015706; RNA-directed DNA polymerase (reverse transcriptase)
GlymaPAV0875	0	2	1	IPR018289; MULE transposase, conserved domain
GlymaPAV0876	0	1	1	
GlymaPAV0877	0	4	0	
GlymaPAV0878	1	0	0	IPR018289; MULE transposase, conserved domain
GlymaPAV0879	1	0	1	IPR000719; Protein kinase, catalytic domain IPR017442; Serine/threonine-protein kinase-like domain IPR020636; Calcium/calmodulin-dependent protein kinase-like IPR020660; CBL-interacting protein kinase
GlymaPAV0880	5	3	0	
GlymaPAV0881	1	0	1	
GlymaPAV0882	2	1	10	
GlymaPAV0883	1	0	7	
GlymaPAV0884	1	2	2	
GlymaPAV0885	1	0	0	
GlymaPAV0886	0	1	0	
GlymaPAV0887	2	1	4	IPR015706; RNA-directed DNA polymerase (reverse transcriptase)
GlymaPAV0888	2	0	1	
GlymaPAV0889	0	0	2	
GlymaPAV0890	1	11	1	IPR000477; Reverse transcriptase
GlymaPAV0891	3	5	1	
GlymaPAV0892	7	7	35	
GlymaPAV0893	4	13	13	IPR001584; Integrase, catalytic core IPR015416; Zinc finger, H2C2-type, histone UAS binding
GlymaPAV0894	0	1	0	
GlymaPAV0895	0	2	1	
GlymaPAV0896	0	1	0	
GlymaPAV0897	1	6	4	
GlymaPAV0898	1	0	1	
GlymaPAV0899	2	2	0	IPR015706; RNA-directed DNA polymerase (reverse transcriptase)
GlymaPAV0900	4	13	14	IPR005162; Retrotransposon gag protein
GlymaPAV0901	8	1	0	IPR000477; Reverse transcriptase
GlymaPAV0902	1	4	0	IPR015706; RNA-directed DNA polymerase (reverse transcriptase)
GlymaPAV0903	0	3	0	
GlymaPAV0904	0	3	1	IPR001584; Integrase, catalytic core
GlymaPAV0905	2	0	1	IPR000477; Reverse transcriptase
GlymaPAV0906	5	20	13	
GlymaPAV0907	0	5	2	IPR015706; RNA-directed DNA polymerase (reverse transcriptase)
GlymaPAV0908	0	6	2	
GlymaPAV0909	0	0	1	
GlymaPAV0910	6	0	1	
GlymaPAV0911	1	8	6	
GlymaPAV0912	3	27	29	IPR001584; Integrase, catalytic core

GlymaPAV0913	0	5	0	
GlymaPAV0914	0	1	2	
GlymaPAV0915	4	15	15	IPR001584; Integrase, catalytic core
GlymaPAV0916	1	0	0	
GlymaPAV0917	2	1	0	
GlymaPAV0918	0	0	5	
GlymaPAV0919	1	1	0	
GlymaPAV0920	0	1	4	
GlymaPAV0921	0	1	2	IPR010285; DNA helicase PIF1, ATP-dependent
GlymaPAV0922	1	1	0	
GlymaPAV0923	1	0	0	
GlymaPAV0924	1	0	0	
GlymaPAV0925	0	1	2	IPR000477; Reverse transcriptase IPR015706; RNA-directed DNA polymerase (reverse transcriptase)
GlymaPAV0926	0	0	1	IPR000440; NADH:ubiquinone/plastoquinone oxidoreductase, chain 3
GlymaPAV0927	1	0	3	IPR013103; Reverse transcriptase, RNA-dependent DNA polymerase
GlymaPAV0928	2	14	6	IPR001584; Integrase, catalytic core
GlymaPAV0929	2	6	7	
GlymaPAV0930	0	0	1	IPR013103; Reverse transcriptase, RNA-dependent DNA polymerase
GlymaPAV0931	1	0	0	IPR001878; Zinc finger, CCHC-type
GlymaPAV0932	3	25	5	
GlymaPAV0933	1	0	0	IPR019557; Aminotransferase-like, plant mobile domain
GlymaPAV0934	1	7	1	IPR019557; Aminotransferase-like, plant mobile domain
GlymaPAV0935	2	5	7	IPR002085; Alcohol dehydrogenase superfamily, zinc-containing
GlymaPAV0936	0	0	1	
GlymaPAV0937	1	1	1	IPR013103; Reverse transcriptase, RNA-dependent DNA polymerase
GlymaPAV0938	0	1	1	
GlymaPAV0939	0	2	4	IPR013103; Reverse transcriptase, RNA-dependent DNA polymerase
GlymaPAV0940	1	11	4	
GlymaPAV0941	1	0	0	IPR019557; Aminotransferase-like, plant mobile domain
GlymaPAV0942	2	3	6	
GlymaPAV0943	3	2	2	IPR019636; Cell wall-associated hydrolase
GlymaPAV0944	4	11	11	IPR013103; Reverse transcriptase, RNA-dependent DNA polymerase
GlymaPAV0945	0	2	1	
GlymaPAV0946	3	0	0	
GlymaPAV0947	0	0	3	
GlymaPAV0948	0	1	0	
GlymaPAV0949	1	2	6	IPR005162; Retrotransposon gag protein
GlymaPAV0950	0	0	8	
GlymaPAV0951	0	0	5	
GlymaPAV0952	0	2	0	
GlymaPAV0953	0	4	1	
GlymaPAV0954	0	0	3	
GlymaPAV0955	1	7	3	
GlymaPAV0956	0	1	0	
GlymaPAV0957	0	1	0	
GlymaPAV0958	5	11	14	
GlymaPAV0959	1	0	1	
GlymaPAV0960	1	8	1	
GlymaPAV0961	1	13	8	IPR019557; Aminotransferase-like, plant mobile domain
GlymaPAV0962	2	1	7	IPR005162; Retrotransposon gag protein
GlymaPAV0963	0	1	0	
GlymaPAV0964	1	12	2	
GlymaPAV0965	0	0	1	IPR001750; NADH:ubiquinone/plastoquinone oxidoreductase
GlymaPAV0966	0	3	0	
GlymaPAV0967	1	22	24	IPR003480; Transferase
GlymaPAV0968	0	2	0	
GlymaPAV0969	3	7	10	
GlymaPAV0970	2	4	3	

GlymaPAV0971	3	0	1	
GlymaPAV0972	0	2	3	
GlymaPAV0973	1	0	0	
GlymaPAV0974	2	0	0	IPR000767; Disease resistance protein IPR002182; NB-ARC
GlymaPAV0975	0	6	1	
GlymaPAV0976	0	0	3	
GlymaPAV0977	1	0	0	
GlymaPAV0978	0	1	6	
GlymaPAV0979	0	1	0	
GlymaPAV0980	0	6	12	
GlymaPAV0981	0	0	1	
GlymaPAV0982	0	2	1	IPR006904; Protein of unknown function DUF716, TMEM45
GlymaPAV0983	0	1	0	
GlymaPAV0984	0	2	0	
GlymaPAV0985	3	7	9	IPR005162; Retrotransposon gag protein
GlymaPAV0986	1	1	5	IPR013242; Retroviral aspartyl protease
GlymaPAV0987	0	20	3	
GlymaPAV0988	1	1	9	
GlymaPAV0989	2	3	5	IPR013242; Retroviral aspartyl protease
GlymaPAV0990	0	3	2	
GlymaPAV0991	1	0	1	IPR002902; Domain of unknown function DUF26
GlymaPAV0992	2	0	3	IPR005162; Retrotransposon gag protein
GlymaPAV0993	1	5	9	IPR005162; Retrotransposon gag protein
Total	1579	3139	3184	

Supplementary Table 7. Population structure analysis of soybean germplasm

Location	ID	K=2			K=3			K=4				K=5					K=6					
China	HN063	HN063	0.18	0.82	0.27	0.73	0.00	0.00	0.75	0.00	0.25	0.31	0.29	0.00	0.41	0.00	0.00	0.27	0.43	0.30	0.00	0.00
South Korea	HN052	HN052	0.36	0.64	0.00	1.00	0.00	0.00	1.00	0.00	0.00	1.00	0.00	0.00	0.00	0.00	0.06	0.94	0.00	0.00	0.00	0.00
South Korea	HN046	HN046	0.35	0.65	0.00	1.00	0.00	0.00	1.00	0.00	0.00	1.00	0.00	0.00	0.00	0.00	0.05	0.96	0.00	0.00	0.00	0.00
South Korea	HN053	HN053	0.34	0.66	0.00	1.00	0.00	0.00	1.00	0.00	0.00	1.00	0.00	0.00	0.00	0.00	0.00	1.00	0.00	0.00	0.00	0.00
Japan	HN040	HN040	0.46	0.54	0.00	0.89	0.11	0.41	0.44	0.15	0.00	0.86	0.00	0.14	0.00	0.00	0.20	0.80	0.00	0.00	0.00	0.00
South Korea	HN045	HN045	0.42	0.58	0.00	1.00	0.00	1.00	0.00	0.00	0.00	1.00	0.00	0.00	0.00	0.00	0.07	0.93	0.00	0.00	0.00	0.00
Japan	HN039	HN039	0.47	0.53	0.00	0.80	0.20	0.80	0.00	0.20	0.00	0.79	0.00	0.19	0.00	0.02	0.27	0.73	0.00	0.00	0.00	0.00
South Korea	HN051	HN051	0.66	0.35	0.08	0.38	0.54	0.10	0.28	0.55	0.08	0.21	0.11	0.60	0.09	0.00	0.60	0.17	0.00	0.09	0.00	0.15
China	HN031	HN031	0.65	0.35	0.26	0.15	0.59	0.00	0.15	0.60	0.25	0.00	0.28	0.58	0.09	0.06	0.63	0.00	0.00	0.25	0.00	0.12
Japan	HN032	HN032	0.50	0.50	0.38	0.20	0.42	0.00	0.20	0.43	0.37	0.00	0.39	0.30	0.14	0.18	0.47	0.00	0.00	0.38	0.00	0.15
China	HN058	HN058	0.64	0.36	0.38	0.01	0.61	0.02	0.00	0.61	0.38	0.00	0.39	0.51	0.00	0.10	0.61	0.00	0.00	0.37	0.00	0.02
China	HN097	HN097	0.59	0.41	0.42	0.01	0.57	0.01	0.01	0.57	0.42	0.00	0.43	0.46	0.00	0.11	0.57	0.00	0.00	0.41	0.00	0.02
South Korea	HN007	HN007	0.73	0.27	0.28	0.02	0.70	0.02	0.00	0.70	0.28	0.00	0.29	0.63	0.00	0.08	0.69	0.00	0.00	0.28	0.00	0.03
South Korea	HN023	HN023	0.81	0.19	0.18	0.04	0.78	0.01	0.03	0.78	0.18	0.00	0.20	0.72	0.01	0.08	0.78	0.00	0.00	0.17	0.00	0.05
Japan	HN011	HN011	0.53	0.47	0.49	0.00	0.51	0.00	0.00	0.51	0.49	0.00	0.50	0.36	0.00	0.14	0.51	0.00	0.00	0.49	0.00	0.00
China	HN080	HN080	0.54	0.46	0.47	0.00	0.53	0.00	0.00	0.53	0.48	0.00	0.48	0.37	0.00	0.15	0.52	0.00	0.00	0.47	0.01	0.00
China	HN078	HN078	0.58	0.42	0.43	0.01	0.56	0.00	0.01	0.56	0.43	0.00	0.43	0.40	0.01	0.16	0.56	0.00	0.00	0.42	0.02	0.00
China	HN079	HN079	0.58	0.42	0.43	0.02	0.55	0.00	0.02	0.55	0.42	0.00	0.43	0.39	0.02	0.17	0.56	0.00	0.00	0.42	0.03	0.00
Japan	HN021	HN021	0.46	0.54	0.54	0.04	0.42	0.05	0.00	0.41	0.54	0.01	0.55	0.33	0.02	0.10	0.42	0.00	0.00	0.54	0.00	0.04
China	HN067	HN067	0.47	0.53	0.55	0.00	0.45	0.00	0.00	0.45	0.55	0.00	0.55	0.26	0.00	0.19	0.45	0.00	0.00	0.55	0.00	0.00
China	HN009	HN009	0.51	0.49	0.51	0.00	0.49	0.00	0.00	0.49	0.51	0.00	0.52	0.31	0.00	0.17	0.49	0.00	0.00	0.51	0.00	0.00
China	HN010	HN010	0.17	0.83	0.83	0.00	0.17	0.00	0.00	0.17	0.83	0.00	0.83	0.10	0.00	0.07	0.17	0.00	0.00	0.83	0.00	0.00
China	HN077	HN077	0.23	0.77	0.75	0.04	0.21	0.00	0.04	0.21	0.75	0.00	0.75	0.17	0.03	0.05	0.22	0.00	0.00	0.75	0.04	0.00
China	HN016	HN016	0.00	1.00	1.00	0.00	0.00	0.00	0.00	0.00	1.00	0.00	0.98	0.00	0.01	0.01	0.02	0.00	0.00	0.98	0.00	0.00
China	HN075	HN075	0.00	1.00	1.00	0.00	0.00	0.00	0.00	0.00	1.00	0.00	1.00	0.00	0.00	0.00	0.00	0.00	0.00	1.00	0.00	0.00
China	HN012	HN012	0.00	1.00	1.00	0.00	0.00	0.00	0.00	0.00	1.00	0.00	0.99	0.00	0.01	0.00	0.02	0.00	0.00	0.98	0.00	0.00
China	HN025	HN025	0.00	1.00	1.00	0.00	0.00	0.00	0.00	0.00	1.00	0.00	0.99	0.00	0.01	0.00	0.01	0.00	0.01	0.99	0.00	0.00
China	HN076	HN076	0.00	1.00	1.00	0.00	0.00	0.00	0.00	0.00	1.00	0.00	1.00	0.00	0.00	0.00	0.00	0.00	0.00	1.00	0.00	0.00
China	HN022	HN022	0.00	1.00	1.00	0.00	0.00	0.00	0.00	0.00	1.00	0.00	1.00	0.00	0.00	0.00	0.00	0.00	0.00	1.00	0.00	0.00
China	HN015	HN015	0.00	1.00	1.00	0.00	0.00	0.00	0.00	0.00	1.00	0.00	1.00	0.00	0.00	0.00	0.00	0.00	0.00	1.00	0.00	0.00
Japan	HN106	HN106	0.00	1.00	1.00	0.00	0.00	0.00	0.00	0.00	1.00	0.00	1.00	0.00	0.00	0.00	0.00	0.00	0.00	1.00	0.00	0.00
China	HN008	HN008	0.00	1.00	1.00	0.00	0.00	0.00	0.00	0.00	1.00	0.00	1.00	0.00	0.00	0.00	0.00	0.00	0.00	1.00	0.00	0.00
China	HN024	HN024	0.30	0.70	0.72	0.00	0.28	0.00	0.00	0.28	0.72	0.00	0.72	0.12	0.00	0.16	0.29	0.00	0.00	0.71	0.00	0.00
North Korea	HN026	HN026	0.29	0.71	0.72	0.00	0.28	0.00	0.00	0.28	0.72	0.00	0.72	0.16	0.00	0.12	0.28	0.00	0.00	0.72	0.00	0.00
N/A	HN104	HN104	0.20	0.80	0.80	0.00	0.20	0.00	0.00	0.20	0.80	0.00	0.80	0.13	0.00	0.06	0.21	0.00	0.00	0.80	0.00	0.00
North Korea	HN068	HN068	0.05	0.95	0.94	0.00	0.06	0.00	0.00	0.06	0.94	0.00	0.94	0.00	0.00	0.06	0.07	0.00	0.00	0.93	0.00	0.00
Japan	HN034	HN034	0.06	0.94	0.93	0.00	0.07	0.00	0.00	0.07	0.93	0.00	0.94	0.06	0.00	0.01	0.07	0.00	0.00	0.93	0.00	0.00
China	HN060	HN060	0.02	0.98	0.96	0.00	0.04	0.00	0.00	0.04	0.96	0.00	0.97	0.03	0.00	0.00	0.05	0.00	0.00	0.95	0.00	0.00
China	HN017	HN017	0.04	0.96	0.96	0.00	0.04	0.00	0.00	0.04	0.96	0.00	0.96	0.00	0.00	0.05	0.05	0.00	0.00	0.96	0.00	0.00

China	**HN020**	HN020	0.05	0.95	0.95	0.00	0.05	0.00	0.00	0.05	0.95	0.00	0.94	0.00	0.00	0.06	0.06	0.00	0.00	0.94	0.00	0.00
China	**HN004**	HN004	0.02	0.99	0.97	0.00	0.03	0.00	0.00	0.03	0.97	0.00	0.97	0.00	0.00	0.03	0.03	0.00	0.00	0.97	0.00	0.00
China	**HN072**	HN072	0.00	1.00	1.00	0.00	0.00	0.00	0.00	0.00	1.00	0.00	1.00	0.00	0.00	0.00	0.00	0.00	0.00	1.00	0.00	0.00
China	**HN047**	HN047	0.00	1.00	1.00	0.00	0.00	0.00	0.00	0.00	1.00	0.00	1.00	0.00	0.00	0.00	0.00	0.00	0.00	1.00	0.00	0.00
China	**HN069**	HN069	0.00	1.00	1.00	0.00	0.00	0.00	0.00	0.00	1.00	0.00	1.00	0.00	0.00	0.00	0.00	0.00	0.00	1.00	0.00	0.00
China	**HN003**	HN003	0.00	1.00	1.00	0.00	0.00	0.00	0.00	0.00	1.00	0.00	1.00	0.00	0.00	0.00	0.00	0.00	0.00	1.00	0.00	0.00
China	**HN018**	HN018	0.00	1.00	1.00	0.00	0.00	0.00	0.00	0.00	1.00	0.00	1.00	0.00	0.00	0.00	0.00	0.00	0.00	1.00	0.00	0.00
China	**HN055**	HN055	0.00	1.00	0.98	0.00	0.02	0.00	0.00	0.02	0.98	0.00	0.97	0.00	0.00	0.03	0.02	0.00	0.00	0.98	0.00	0.00
China	**HN005**	HN005	0.00	1.00	1.00	0.00	0.00	0.00	0.00	0.00	1.00	0.00	1.00	0.00	0.00	0.00	0.00	0.00	0.00	1.00	0.00	0.00
China	**HN019**	HN019	0.00	1.00	1.00	0.00	0.00	0.00	0.00	0.00	1.00	0.00	1.00	0.00	0.00	0.00	0.00	0.00	0.00	1.00	0.00	0.00
China	**HN002**	HN002	0.00	1.00	1.00	0.00	0.00	0.00	0.00	0.00	1.00	0.00	1.00	0.00	0.00	0.00	0.00	0.00	0.00	1.00	0.00	0.00
China	**HN091**	HN091	0.83	0.17	0.16	0.03	0.81	0.04	0.00	0.80	0.16	0.00	0.17	0.64	0.01	0.18	0.81	0.00	0.00	0.16	0.00	0.04
China	**HN074**	HN074	0.78	0.22	0.24	0.00	0.76	0.00	0.00	0.75	0.25	0.00	0.24	0.60	0.00	0.16	0.75	0.00	0.00	0.24	0.01	0.00
China	**HN090**	HN090	0.79	0.21	0.19	0.05	0.76	0.06	0.00	0.75	0.19	0.00	0.20	0.63	0.03	0.14	0.77	0.00	0.00	0.18	0.00	0.05
Vietnam	**HN098**	HN098	0.80	0.20	0.19	0.03	0.78	0.04	0.00	0.77	0.20	0.00	0.20	0.66	0.01	0.13	0.78	0.00	0.00	0.19	0.00	0.03
China	**HN083**	HN083	0.71	0.29	0.31	0.00	0.69	0.00	0.00	0.69	0.31	0.00	0.31	0.50	0.00	0.19	0.69	0.00	0.00	0.30	0.01	0.00
China	**HN082**	HN082	0.57	0.43	0.45	0.00	0.55	0.00	0.00	0.55	0.45	0.00	0.46	0.42	0.00	0.13	0.55	0.00	0.00	0.45	0.01	0.00
China	**HN085**	HN085	0.63	0.37	0.39	0.00	0.61	0.00	0.00	0.61	0.39	0.00	0.39	0.50	0.00	0.10	0.61	0.00	0.00	0.39	0.00	0.00
China	**HN084**	HN084	0.59	0.41	0.44	0.00	0.57	0.00	0.00	0.57	0.44	0.00	0.44	0.45	0.00	0.12	0.57	0.00	0.00	0.43	0.00	0.00
China	**HN086**	HN086	0.59	0.41	0.43	0.00	0.57	0.00	0.00	0.57	0.43	0.00	0.43	0.46	0.00	0.11	0.57	0.00	0.00	0.43	0.00	0.00
Sweden	**HN105**	HN105	0.90	0.10	0.10	0.04	0.86	0.06	0.00	0.85	0.10	0.00	0.12	0.78	0.00	0.11	0.86	0.00	0.00	0.09	0.00	0.04
U.S.	**HN103**	HN103	1.00	0.00	0.00	0.00	1.00	0.00	0.00	1.00	0.00	0.00	0.00	0.67	0.00	0.33	1.00	0.00	0.00	0.00	0.00	0.00
Japan	**HN107**	HN107	1.00	0.00	0.00	0.05	0.95	0.07	0.00	0.93	0.00	0.00	0.00	0.97	0.00	0.03	0.95	0.00	0.00	0.00	0.00	0.05
Japan	**HN037**	HN037	1.00	0.00	0.00	0.06	0.94	0.08	0.00	0.92	0.00	0.02	0.00	0.94	0.00	0.04	0.95	0.00	0.00	0.00	0.00	0.06
Japan	**HN050**	HN050	1.00	0.00	0.00	0.03	0.97	0.05	0.00	0.95	0.00	0.00	0.00	0.94	0.00	0.06	0.97	0.00	0.00	0.00	0.00	0.03
South Korea	**HN089**	HN089	1.00	0.00	0.00	0.06	0.94	0.09	0.00	0.92	0.00	0.00	0.00	0.93	0.00	0.07	0.95	0.00	0.00	0.00	0.00	0.05
Japan	**HN038**	HN038	1.00	0.00	0.00	0.03	0.97	0.06	0.00	0.95	0.00	0.00	0.00	0.96	0.00	0.04	0.97	0.00	0.00	0.00	0.00	0.03
North Korea	**HN096**	HN096	0.97	0.03	0.03	0.04	0.94	0.02	0.02	0.93	0.03	0.00	0.03	0.87	0.00	0.10	0.94	0.00	0.00	0.01	0.00	0.05
North Korea	**HN033**	HN033	1.00	0.00	0.00	0.00	1.00	0.02	0.00	0.99	0.00	0.00	0.00	0.87	0.00	0.13	0.98	0.00	0.00	0.00	0.00	0.02
South Korea	**HN049**	HN049	1.00	0.00	0.00	0.00	1.00	0.00	0.00	1.00	0.00	0.00	0.00	0.94	0.00	0.06	1.00	0.00	0.00	0.00	0.00	0.00
South Korea	**HN048**	HN048	1.00	0.00	0.00	0.06	0.94	0.07	0.00	0.93	0.00	0.00	0.00	0.98	0.00	0.02	0.94	0.00	0.00	0.00	0.00	0.06
North Korea	**HN093**	HN093	1.00	0.00	0.00	0.00	1.00	0.02	0.00	0.98	0.00	0.00	0.00	0.88	0.00	0.12	0.98	0.00	0.00	0.00	0.00	0.02
South Korea	**HN006**	HN006	1.00	0.00	0.00	0.03	0.97	0.04	0.00	0.96	0.00	0.00	0.01	0.95	0.00	0.04	0.96	0.00	0.00	0.00	0.00	0.04
South Korea	**HN035**	HN035	1.00	0.00	0.00	0.00	1.00	0.00	0.00	1.00	0.00	0.00	0.00	1.00	0.00	0.00	0.98	0.00	0.00	0.00	0.00	0.02
North Korea	**HN095**	HN095	1.00	0.00	0.00	0.01	0.99	0.03	0.00	0.98	0.00	0.00	0.00	1.00	0.00	0.00	0.98	0.00	0.00	0.00	0.00	0.02
North Korea	**HN094**	HN094	1.00	0.00	0.02	0.00	0.98	0.01	0.00	0.97	0.02	0.00	0.00	0.92	0.00	0.08	0.97	0.00	0.00	0.02	0.00	0.02
South Korea	**HN044**	HN044	1.00	0.00	0.00	0.00	1.00	0.00	0.00	1.00	0.00	0.00	0.00	1.00	0.00	0.00	1.00	0.00	0.00	0.00	0.00	0.00
South Korea	**HN043**	HN043	1.00	0.00	0.00	0.00	1.00	0.00	0.00	1.00	0.00	0.00	0.00	1.00	0.00	0.00	1.00	0.00	0.00	0.00	0.00	0.00
South Korea	**HN041**	HN041	1.00	0.00	0.00	0.00	1.00	0.00	0.00	1.00	0.00	0.00	0.00	1.00	0.00	0.00	1.00	0.00	0.00	0.00	0.00	0.00
South Korea	**HN042**	HN042	1.00	0.00	0.00	0.00	1.00	0.00	0.00	1.00	0.00	0.00	0.00	1.00	0.00	0.00	1.00	0.00	0.00	0.00	0.00	0.00
China	**HN057**	HN057	0.93	0.07	0.09	0.00	0.91	0.00	0.00	0.91	0.09	0.00	0.08	0.44	0.00	0.49	0.91	0.00	0.00	0.09	0.00	0.00
Russian Federation	**HN054**	HN054	0.98	0.02	0.04	0.00	0.96	0.00	0.00	0.96	0.04	0.00	0.03	0.34	0.00	0.63	0.96	0.00	0.00	0.04	0.00	0.00

China	**HN056**	HN056	0.85	0.15	0.17	0.00	0.83	0.00	0.00	0.83	0.17	0.00	0.15	0.20	0.00	0.65	0.83	0.00	0.00	0.17	0.00	0.00
China	**HN059**	HN059	0.86	0.14	0.16	0.00	0.84	0.00	0.00	0.84	0.16	0.00	0.15	0.36	0.00	0.50	0.84	0.00	0.00	0.16	0.00	0.00
U.S.	**HN013**	HN013	0.95	0.05	0.07	0.00	0.93	0.00	0.00	0.93	0.07	0.00	0.06	0.26	0.00	0.68	0.93	0.00	0.00	0.08	0.00	0.00
U.S.	**HN027**	HN027	0.98	0.02	0.04	0.00	0.96	0.00	0.00	0.96	0.04	0.00	0.03	0.13	0.00	0.85	0.96	0.00	0.00	0.05	0.00	0.00
Nepal	**HN061**	HN061	0.98	0.02	0.04	0.00	0.96	0.00	0.00	0.96	0.04	0.00	0.03	0.47	0.00	0.50	0.96	0.00	0.00	0.04	0.00	0.00
U.S.	**HN071**	HN071	0.95	0.05	0.07	0.00	0.93	0.00	0.00	0.93	0.07	0.00	0.06	0.48	0.00	0.46	0.93	0.00	0.00	0.07	0.00	0.00
U.S.	**HN014**	HN014	1.00	0.00	0.00	0.00	1.00	0.00	0.00	1.00	0.00	0.00	0.00	0.16	0.00	0.84	1.00	0.00	0.00	0.00	0.00	0.00
U.S.	**HN001**	HN001	1.00	0.01	0.03	0.00	0.97	0.00	0.00	0.97	0.03	0.00	0.00	0.25	0.00	0.75	0.97	0.00	0.00	0.03	0.00	0.00
U.S.	**HN100**	HN100	1.00	0.00	0.00	0.00	1.00	0.00	0.00	1.00	0.00	0.00	0.00	0.44	0.00	0.56	1.00	0.00	0.00	0.00	0.00	0.00
U.S.	**HN028**	HN028	1.00	0.00	0.00	0.00	1.00	0.00	0.00	1.00	0.00	0.00	0.00	0.36	0.00	0.64	1.00	0.00	0.00	0.00	0.00	0.00
Former Serbia	**HN064**	HN064	0.86	0.14	0.17	0.00	0.83	0.00	0.00	0.83	0.17	0.00	0.15	0.22	0.00	0.63	0.83	0.00	0.00	0.17	0.00	0.00
U.S.	**HN070**	HN070	0.97	0.03	0.05	0.00	0.95	0.00	0.00	0.95	0.05	0.00	0.03	0.20	0.00	0.77	0.95	0.00	0.00	0.05	0.00	0.00
China	**HN062**	HN062	0.96	0.04	0.06	0.00	0.94	0.00	0.00	0.94	0.06	0.00	0.04	0.20	0.00	0.75	0.94	0.00	0.00	0.06	0.00	0.00
China	**HN081**	HN081	0.81	0.19	0.23	0.00	0.77	0.00	0.00	0.77	0.23	0.00	0.19	0.09	0.00	0.72	0.77	0.00	0.00	0.23	0.00	0.00
U.S.	**HN092**	HN092	0.96	0.04	0.07	0.00	0.93	0.00	0.00	0.93	0.07	0.00	0.03	0.00	0.00	0.97	0.93	0.00	0.00	0.07	0.00	0.00
N/A	**HN101**	HN101	0.93	0.07	0.10	0.00	0.90	0.00	0.00	0.90	0.10	0.00	0.07	0.07	0.00	0.87	0.89	0.00	0.00	0.11	0.00	0.00
U.S.	**HN030**	HN030	1.00	0.00	0.01	0.00	0.99	0.00	0.00	0.99	0.01	0.00	0.00	0.00	0.00	1.00	0.99	0.00	0.00	0.02	0.00	0.00
U.S.	**HN099**	HN099	0.99	0.01	0.04	0.00	0.96	0.00	0.00	0.96	0.04	0.00	0.00	0.00	0.00	1.00	0.96	0.00	0.00	0.04	0.00	0.00
U.S.	**HN073**	HN073	1.00	0.00	0.00	0.00	1.00	0.00	0.00	1.00	0.00	0.00	0.00	0.00	0.00	1.00	1.00	0.00	0.00	0.00	0.00	0.00
N/A	**HN088**	HN088	1.00	0.00	0.00	0.00	1.00	0.00	0.00	1.00	0.00	0.00	0.00	0.00	0.00	1.00	1.00	0.00	0.00	0.00	0.00	0.00
U.S.	**HN102**	HN102	1.00	0.00	0.01	0.00	0.99	0.00	0.00	0.99	0.01	0.00	0.00	0.12	0.00	0.88	0.99	0.00	0.00	0.01	0.00	0.00
U.S.	**HN029**	HN029	1.00	0.00	0.00	0.00	1.00	0.00	0.00	1.00	0.00	0.00	0.00	0.00	0.00	1.00	1.00	0.00	0.00	0.00	0.00	0.00
U.S.	**HN066**	HN066	1.00	0.00	0.00	0.00	1.00	0.00	0.00	1.00	0.00	0.00	0.00	0.00	0.00	1.00	1.00	0.00	0.00	0.00	0.00	0.00
U.S.	**HN087**	HN087	1.00	0.00	0.00	0.00	1.00	0.00	0.00	1.00	0.00	0.00	0.00	0.00	0.00	1.00	1.00	0.00	0.00	0.00	0.00	0.00
U.S.	**HN065**	HN065	1.00	0.00	0.00	0.00	1.00	0.00	0.00	1.00	0.00	0.00	0.00	0.00	0.00	1.00	1.00	0.00	0.00	0.00	0.00	0.00

Supplementary Table 8. Distribution of LD blocks in three soybean gene pools.

Distance(bp)	Landrace	Elite	Wild	Distance(bp)	Landrace	Elite	Wild
3	0.4371	0.4594	0.4017	124110	0.2086	0.2039	0.1644
4	0.4168	0.4369	0.3862	124310	0.2083	0.2036	0.1642
5	0.4196	0.4358	0.3809	124510	0.2082	0.2037	0.1641
6	0.4098	0.4259	0.3752	124710	0.2085	0.2037	0.1642
7	0.4106	0.4286	0.3720	124910	0.2080	0.2032	0.1642
8	0.4072	0.4236	0.3684	125110	0.2080	0.2031	0.1641
9	0.4076	0.4238	0.3677	125310	0.2080	0.2035	0.1641
10	0.4051	0.4207	0.3653	125510	0.2081	0.2030	0.1642
110	0.3981	0.4163	0.3489	125710	0.2080	0.2032	0.1642
310	0.3867	0.4040	0.3335	125910	0.2077	0.2031	0.1640
510	0.3809	0.3979	0.3264	126110	0.2077	0.2030	0.1642
710	0.3763	0.3929	0.3206	126310	0.2076	0.2029	0.1640
910	0.3734	0.3897	0.3164	126510	0.2073	0.2031	0.1639
1110	0.3703	0.3859	0.3125	126710	0.2073	0.2031	0.1640
1310	0.3676	0.3829	0.3084	126910	0.2074	0.2029	0.1638
1510	0.3653	0.3802	0.3053	127110	0.2071	0.2030	0.1638
1710	0.3628	0.3774	0.3022	127310	0.2072	0.2024	0.1637
1910	0.3605	0.3745	0.2991	127510	0.2069	0.2025	0.1637
2110	0.3590	0.3725	0.2966	127710	0.2070	0.2024	0.1636
2310	0.3568	0.3704	0.2940	127910	0.2070	0.2024	0.1636
2510	0.3548	0.3683	0.2914	128110	0.2068	0.2022	0.1637
2710	0.3527	0.3660	0.2890	128310	0.2068	0.2022	0.1636
2910	0.3514	0.3641	0.2868	128510	0.2066	0.2021	0.1636
3110	0.3494	0.3620	0.2844	128710	0.2065	0.2021	0.1635
3310	0.3478	0.3601	0.2823	128910	0.2066	0.2021	0.1635
3510	0.3461	0.3582	0.2803	129110	0.2063	0.2017	0.1634
3710	0.3445	0.3564	0.2782	129310	0.2063	0.2018	0.1634
3910	0.3435	0.3552	0.2763	129510	0.2065	0.2018	0.1634
4110	0.3420	0.3534	0.2746	129710	0.2062	0.2017	0.1634
4310	0.3405	0.3514	0.2727	129910	0.2062	0.2015	0.1632
4510	0.3391	0.3499	0.2710	130110	0.2061	0.2016	0.1631
4710	0.3375	0.3480	0.2692	130310	0.2061	0.2014	0.1631
4910	0.3363	0.3469	0.2677	130510	0.2061	0.2011	0.1630
5110	0.3350	0.3455	0.2661	130710	0.2059	0.2014	0.1630
5310	0.3338	0.3437	0.2646	130910	0.2057	0.2012	0.1629
5510	0.3329	0.3427	0.2632	131110	0.2055	0.2010	0.1629
5710	0.3317	0.3412	0.2617	131310	0.2056	0.2010	0.1629
5910	0.3303	0.3395	0.2602	131510	0.2057	0.2010	0.1629
6110	0.3292	0.3387	0.2588	131710	0.2054	0.2009	0.1628
6310	0.3283	0.3371	0.2577	131910	0.2054	0.2009	0.1627
6510	0.3272	0.3358	0.2562	132110	0.2051	0.2005	0.1627
6710	0.3259	0.3346	0.2550	132310	0.2050	0.2004	0.1626
6910	0.3254	0.3335	0.2540	132510	0.2053	0.2004	0.1626
7110	0.3240	0.3325	0.2527	132710	0.2048	0.2004	0.1626
7310	0.3231	0.3313	0.2517	132910	0.2051	0.2004	0.1627
7510	0.3223	0.3303	0.2506	133110	0.2050	0.2002	0.1625
7710	0.3214	0.3292	0.2494	133310	0.2046	0.2001	0.1626
7910	0.3204	0.3282	0.2482	133510	0.2046	0.2001	0.1624
8110	0.3198	0.3271	0.2472	133710	0.2044	0.2001	0.1624
8310	0.3187	0.3261	0.2462	133910	0.2045	0.2002	0.1625
8510	0.3179	0.3248	0.2450	134110	0.2046	0.2002	0.1625

8710	0.3167	0.3237	0.2439	134310	0.2047	0.1999	0.1623
8910	0.3160	0.3229	0.2431	134510	0.2048	0.1999	0.1622
9110	0.3151	0.3220	0.2422	134710	0.2044	0.1997	0.1623
9310	0.3143	0.3211	0.2413	134910	0.2043	0.1997	0.1623
9510	0.3136	0.3202	0.2405	135110	0.2044	0.1997	0.1623
9710	0.3132	0.3194	0.2394	135310	0.2041	0.1995	0.1622
9910	0.3122	0.3187	0.2388	135510	0.2040	0.1992	0.1622
10110	0.3116	0.3177	0.2379	135710	0.2039	0.1992	0.1620
10310	0.3105	0.3165	0.2367	135910	0.2040	0.1991	0.1621
10510	0.3099	0.3160	0.2359	136110	0.2039	0.1990	0.1622
10710	0.3093	0.3150	0.2351	136310	0.2037	0.1987	0.1620
10910	0.3084	0.3144	0.2345	136510	0.2039	0.1989	0.1620
11110	0.3078	0.3137	0.2340	136710	0.2039	0.1988	0.1620
11310	0.3072	0.3127	0.2331	136910	0.2038	0.1988	0.1620
11510	0.3064	0.3121	0.2324	137110	0.2036	0.1986	0.1618
11710	0.3058	0.3112	0.2317	137310	0.2037	0.1985	0.1618
11910	0.3052	0.3106	0.2309	137510	0.2035	0.1985	0.1618
12110	0.3044	0.3098	0.2304	137710	0.2033	0.1984	0.1617
12310	0.3040	0.3091	0.2297	137910	0.2033	0.1981	0.1618
12510	0.3035	0.3082	0.2290	138110	0.2033	0.1981	0.1616
12710	0.3027	0.3078	0.2287	138310	0.2032	0.1981	0.1617
12910	0.3023	0.3074	0.2280	138510	0.2030	0.1980	0.1616
13110	0.3020	0.3065	0.2275	138710	0.2031	0.1982	0.1617
13310	0.3012	0.3060	0.2267	138910	0.2028	0.1983	0.1617
13510	0.3006	0.3051	0.2261	139110	0.2029	0.1982	0.1617
13710	0.3003	0.3043	0.2256	139310	0.2029	0.1984	0.1618
13910	0.2993	0.3038	0.2248	139510	0.2032	0.1982	0.1617
14110	0.2988	0.3032	0.2244	139710	0.2026	0.1978	0.1616
14310	0.2982	0.3025	0.2239	139910	0.2023	0.1977	0.1616
14510	0.2979	0.3019	0.2234	140110	0.2025	0.1978	0.1615
14710	0.2974	0.3013	0.2226	140310	0.2027	0.1980	0.1615
14910	0.2967	0.3007	0.2219	140510	0.2023	0.1975	0.1615
15110	0.2961	0.3001	0.2214	140710	0.2023	0.1976	0.1614
15310	0.2957	0.2994	0.2209	140910	0.2022	0.1974	0.1615
15510	0.2952	0.2994	0.2204	141110	0.2019	0.1972	0.1612
15710	0.2949	0.2987	0.2199	141310	0.2019	0.1975	0.1612
15910	0.2944	0.2981	0.2194	141510	0.2018	0.1970	0.1610
16110	0.2939	0.2977	0.2191	141710	0.2016	0.1970	0.1611
16310	0.2937	0.2972	0.2185	141910	0.2017	0.1969	0.1612
16510	0.2935	0.2965	0.2180	142110	0.2016	0.1969	0.1611
16710	0.2930	0.2959	0.2176	142310	0.2015	0.1968	0.1611
16910	0.2923	0.2950	0.2169	142510	0.2015	0.1970	0.1611
17110	0.2919	0.2946	0.2166	142710	0.2016	0.1971	0.1611
17310	0.2913	0.2940	0.2163	142910	0.2014	0.1966	0.1610
17510	0.2908	0.2935	0.2159	143110	0.2016	0.1965	0.1610
17710	0.2903	0.2928	0.2152	143310	0.2013	0.1964	0.1609
17910	0.2901	0.2925	0.2147	143510	0.2014	0.1964	0.1607
18110	0.2896	0.2919	0.2144	143710	0.2013	0.1964	0.1609
18310	0.2891	0.2914	0.2141	143910	0.2012	0.1965	0.1608
18510	0.2888	0.2908	0.2136	144110	0.2014	0.1964	0.1610
18710	0.2883	0.2903	0.2130	144310	0.2012	0.1961	0.1609
18910	0.2879	0.2900	0.2127	144510	0.2012	0.1961	0.1609
19110	0.2875	0.2893	0.2126	144710	0.2011	0.1959	0.1608
19310	0.2869	0.2888	0.2118	144910	0.2015	0.1962	0.1609

19510	0.2867	0.2888	0.2115	145110	0.2012	0.1959	0.1608
19710	0.2861	0.2880	0.2112	145310	0.2010	0.1959	0.1607
19910	0.2857	0.2876	0.2108	145510	0.2009	0.1958	0.1607
20110	0.2855	0.2872	0.2102	145710	0.2007	0.1956	0.1606
20310	0.2854	0.2867	0.2101	145910	0.2009	0.1957	0.1607
20510	0.2847	0.2866	0.2097	146110	0.2008	0.1957	0.1606
20710	0.2844	0.2859	0.2095	146310	0.2008	0.1954	0.1605
20910	0.2837	0.2854	0.2093	146510	0.2008	0.1957	0.1607
21110	0.2837	0.2853	0.2090	146710	0.2003	0.1952	0.1605
21310	0.2832	0.2848	0.2087	146910	0.2004	0.1954	0.1604
21510	0.2832	0.2844	0.2084	147110	0.2003	0.1953	0.1604
21710	0.2827	0.2839	0.2080	147310	0.2003	0.1954	0.1605
21910	0.2821	0.2835	0.2075	147510	0.2004	0.1951	0.1604
22110	0.2821	0.2833	0.2073	147710	0.2003	0.1950	0.1603
22310	0.2819	0.2830	0.2071	147910	0.2000	0.1945	0.1603
22510	0.2814	0.2828	0.2069	148110	0.1998	0.1945	0.1602
22710	0.2811	0.2823	0.2066	148310	0.1998	0.1947	0.1601
22910	0.2808	0.2820	0.2061	148510	0.1999	0.1948	0.1602
23110	0.2804	0.2812	0.2059	148710	0.1998	0.1950	0.1602
23310	0.2798	0.2810	0.2056	148910	0.1997	0.1947	0.1601
23510	0.2798	0.2810	0.2054	149110	0.1995	0.1944	0.1602
23710	0.2796	0.2807	0.2051	149310	0.1997	0.1947	0.1603
23910	0.2791	0.2802	0.2047	149510	0.1994	0.1945	0.1602
24110	0.2791	0.2798	0.2046	149710	0.1994	0.1944	0.1602
24310	0.2786	0.2796	0.2041	149910	0.1995	0.1945	0.1600
24510	0.2779	0.2789	0.2040	150110	0.1995	0.1944	0.1600
24710	0.2781	0.2786	0.2036	150310	0.1995	0.1944	0.1600
24910	0.2777	0.2781	0.2034	150510	0.1991	0.1941	0.1599
25110	0.2773	0.2778	0.2029	150710	0.1990	0.1940	0.1599
25310	0.2775	0.2775	0.2028	150910	0.1991	0.1939	0.1599
25510	0.2770	0.2774	0.2026	151110	0.1986	0.1939	0.1598
25710	0.2766	0.2765	0.2021	151310	0.1986	0.1937	0.1597
25910	0.2762	0.2764	0.2021	151510	0.1985	0.1937	0.1597
26110	0.2759	0.2762	0.2017	151710	0.1987	0.1938	0.1599
26310	0.2754	0.2758	0.2014	151910	0.1985	0.1937	0.1598
26510	0.2751	0.2753	0.2010	152110	0.1985	0.1934	0.1598
26710	0.2748	0.2750	0.2011	152310	0.1986	0.1935	0.1598
26910	0.2746	0.2747	0.2008	152510	0.1985	0.1935	0.1597
27110	0.2743	0.2745	0.2007	152710	0.1983	0.1935	0.1600
27310	0.2745	0.2744	0.2003	152910	0.1983	0.1934	0.1597
27510	0.2742	0.2742	0.2001	153110	0.1980	0.1931	0.1598
27710	0.2738	0.2735	0.1999	153310	0.1981	0.1931	0.1599
27910	0.2734	0.2731	0.1996	153510	0.1978	0.1931	0.1598
28110	0.2732	0.2732	0.1995	153710	0.1980	0.1932	0.1597
28310	0.2730	0.2728	0.1992	153910	0.1981	0.1932	0.1598
28510	0.2722	0.2720	0.1990	154110	0.1978	0.1929	0.1597
28710	0.2723	0.2717	0.1985	154310	0.1977	0.1927	0.1597
28910	0.2719	0.2716	0.1986	154510	0.1979	0.1928	0.1597
29110	0.2715	0.2711	0.1982	154710	0.1976	0.1927	0.1597
29310	0.2715	0.2710	0.1980	154910	0.1979	0.1928	0.1596
29510	0.2714	0.2709	0.1980	155110	0.1978	0.1926	0.1598
29710	0.2712	0.2704	0.1977	155310	0.1978	0.1928	0.1596
29910	0.2709	0.2700	0.1975	155510	0.1974	0.1927	0.1596
30110	0.2708	0.2700	0.1974	155710	0.1975	0.1925	0.1596

30310	0.2702	0.2696	0.1971	155910	0.1975	0.1925	0.1598
30510	0.2700	0.2693	0.1969	156110	0.1971	0.1921	0.1595
30710	0.2697	0.2693	0.1968	156310	0.1975	0.1922	0.1597
30910	0.2691	0.2687	0.1965	156510	0.1970	0.1923	0.1597
31110	0.2691	0.2685	0.1964	156710	0.1972	0.1924	0.1595
31310	0.2689	0.2681	0.1963	156910	0.1975	0.1923	0.1595
31510	0.2686	0.2678	0.1961	157110	0.1972	0.1921	0.1595
31710	0.2686	0.2675	0.1958	157310	0.1970	0.1919	0.1594
31910	0.2682	0.2674	0.1957	157510	0.1969	0.1920	0.1595
32110	0.2676	0.2669	0.1953	157710	0.1966	0.1916	0.1594
32310	0.2677	0.2671	0.1953	157910	0.1969	0.1916	0.1595
32510	0.2674	0.2666	0.1952	158110	0.1966	0.1915	0.1595
32710	0.2673	0.2666	0.1949	158310	0.1968	0.1917	0.1594
32910	0.2672	0.2664	0.1948	158510	0.1966	0.1915	0.1593
33110	0.2670	0.2661	0.1947	158710	0.1967	0.1914	0.1593
33310	0.2664	0.2655	0.1944	158910	0.1964	0.1911	0.1592
33510	0.2661	0.2654	0.1942	159110	0.1966	0.1914	0.1593
33710	0.2659	0.2653	0.1942	159310	0.1965	0.1914	0.1594
33910	0.2660	0.2649	0.1939	159510	0.1964	0.1913	0.1594
34110	0.2655	0.2648	0.1938	159710	0.1963	0.1912	0.1593
34310	0.2653	0.2645	0.1935	159910	0.1965	0.1913	0.1593
34510	0.2652	0.2642	0.1934	160110	0.1963	0.1913	0.1593
34710	0.2649	0.2640	0.1932	160310	0.1960	0.1912	0.1593
34910	0.2650	0.2636	0.1934	160510	0.1960	0.1910	0.1592
35110	0.2645	0.2634	0.1931	160710	0.1963	0.1910	0.1592
35310	0.2643	0.2635	0.1927	160910	0.1962	0.1911	0.1592
35510	0.2643	0.2630	0.1928	161110	0.1962	0.1910	0.1592
35710	0.2639	0.2626	0.1926	161310	0.1960	0.1909	0.1591
35910	0.2637	0.2622	0.1926	161510	0.1961	0.1911	0.1590
36110	0.2634	0.2618	0.1922	161710	0.1960	0.1907	0.1590
36310	0.2630	0.2614	0.1920	161910	0.1959	0.1905	0.1589
36510	0.2631	0.2615	0.1920	162110	0.1957	0.1907	0.1589
36710	0.2628	0.2614	0.1917	162310	0.1960	0.1907	0.1589
36910	0.2626	0.2611	0.1916	162510	0.1958	0.1905	0.1590
37110	0.2623	0.2607	0.1914	162710	0.1958	0.1906	0.1589
37310	0.2621	0.2607	0.1912	162910	0.1953	0.1904	0.1588
37510	0.2619	0.2604	0.1911	163110	0.1955	0.1903	0.1588
37710	0.2617	0.2600	0.1911	163310	0.1954	0.1902	0.1588
37910	0.2615	0.2598	0.1908	163510	0.1955	0.1904	0.1587
38110	0.2611	0.2595	0.1907	163710	0.1955	0.1903	0.1589
38310	0.2610	0.2594	0.1905	163910	0.1955	0.1903	0.1587
38510	0.2605	0.2589	0.1902	164110	0.1954	0.1904	0.1587
38710	0.2605	0.2588	0.1899	164310	0.1954	0.1903	0.1587
38910	0.2605	0.2583	0.1897	164510	0.1951	0.1902	0.1586
39110	0.2605	0.2586	0.1899	164710	0.1953	0.1900	0.1586
39310	0.2598	0.2578	0.1896	164910	0.1953	0.1901	0.1586
39510	0.2597	0.2575	0.1894	165110	0.1951	0.1900	0.1585
39710	0.2596	0.2575	0.1894	165310	0.1949	0.1896	0.1585
39910	0.2592	0.2570	0.1893	165510	0.1948	0.1897	0.1586
40110	0.2593	0.2570	0.1891	165710	0.1948	0.1895	0.1585
40310	0.2588	0.2565	0.1888	165910	0.1945	0.1894	0.1585
40510	0.2586	0.2562	0.1887	166110	0.1948	0.1896	0.1586
40710	0.2584	0.2560	0.1886	166310	0.1944	0.1894	0.1584
40910	0.2581	0.2559	0.1885	166510	0.1945	0.1895	0.1585

41110	0.2579	0.2555	0.1882	166710	0.1946	0.1892	0.1584
41310	0.2582	0.2557	0.1883	166910	0.1945	0.1892	0.1584
41510	0.2579	0.2553	0.1882	167110	0.1942	0.1889	0.1585
41710	0.2574	0.2550	0.1878	167310	0.1943	0.1892	0.1583
41910	0.2577	0.2551	0.1878	167510	0.1943	0.1891	0.1585
42110	0.2573	0.2545	0.1876	167710	0.1939	0.1887	0.1583
42310	0.2569	0.2545	0.1876	167910	0.1938	0.1888	0.1583
42510	0.2567	0.2544	0.1874	168110	0.1937	0.1888	0.1583
42710	0.2563	0.2540	0.1871	168310	0.1938	0.1889	0.1584
42910	0.2562	0.2535	0.1870	168510	0.1936	0.1888	0.1583
43110	0.2562	0.2534	0.1871	168710	0.1938	0.1888	0.1584
43310	0.2558	0.2531	0.1868	168910	0.1936	0.1887	0.1582
43510	0.2559	0.2529	0.1866	169110	0.1936	0.1889	0.1582
43710	0.2556	0.2528	0.1867	169310	0.1933	0.1884	0.1581
43910	0.2555	0.2527	0.1865	169510	0.1935	0.1886	0.1581
44110	0.2553	0.2524	0.1864	169710	0.1938	0.1886	0.1582
44310	0.2552	0.2520	0.1865	169910	0.1934	0.1885	0.1583
44510	0.2551	0.2520	0.1864	170110	0.1934	0.1883	0.1582
44710	0.2548	0.2520	0.1863	170310	0.1934	0.1881	0.1583
44910	0.2548	0.2517	0.1861	170510	0.1934	0.1883	0.1581
45110	0.2543	0.2514	0.1859	170710	0.1931	0.1881	0.1580
45310	0.2543	0.2514	0.1859	170910	0.1932	0.1882	0.1579
45510	0.2542	0.2510	0.1858	171110	0.1932	0.1878	0.1578
45710	0.2541	0.2509	0.1856	171310	0.1931	0.1880	0.1579
45910	0.2537	0.2505	0.1854	171510	0.1931	0.1879	0.1579
46110	0.2534	0.2503	0.1853	171710	0.1929	0.1880	0.1579
46310	0.2535	0.2502	0.1854	171910	0.1930	0.1879	0.1577
46510	0.2529	0.2500	0.1851	172110	0.1928	0.1876	0.1579
46710	0.2527	0.2497	0.1850	172310	0.1925	0.1875	0.1578
46910	0.2525	0.2495	0.1850	172510	0.1925	0.1875	0.1579
47110	0.2522	0.2492	0.1850	172710	0.1925	0.1877	0.1580
47310	0.2524	0.2492	0.1847	172910	0.1925	0.1875	0.1579
47510	0.2522	0.2489	0.1847	173110	0.1923	0.1874	0.1578
47710	0.2520	0.2490	0.1845	173310	0.1922	0.1874	0.1580
47910	0.2519	0.2488	0.1844	173510	0.1919	0.1870	0.1578
48110	0.2518	0.2486	0.1843	173710	0.1920	0.1872	0.1578
48310	0.2517	0.2484	0.1842	173910	0.1918	0.1871	0.1576
48510	0.2516	0.2480	0.1841	174110	0.1920	0.1872	0.1578
48710	0.2514	0.2477	0.1840	174310	0.1917	0.1868	0.1577
48910	0.2510	0.2476	0.1840	174510	0.1917	0.1870	0.1577
49110	0.2511	0.2474	0.1839	174710	0.1917	0.1869	0.1576
49310	0.2506	0.2471	0.1838	174910	0.1915	0.1865	0.1577
49510	0.2508	0.2471	0.1836	175110	0.1914	0.1868	0.1575
49710	0.2505	0.2471	0.1835	175310	0.1915	0.1870	0.1576
49910	0.2503	0.2468	0.1835	175510	0.1916	0.1869	0.1577
50110	0.2502	0.2466	0.1834	175710	0.1914	0.1867	0.1578
50310	0.2500	0.2465	0.1833	175910	0.1916	0.1866	0.1578
50510	0.2499	0.2461	0.1832	176110	0.1914	0.1867	0.1578
50710	0.2498	0.2460	0.1830	176310	0.1916	0.1866	0.1577
50910	0.2495	0.2459	0.1830	176510	0.1914	0.1863	0.1578
51110	0.2492	0.2453	0.1829	176710	0.1912	0.1865	0.1576
51310	0.2490	0.2454	0.1830	176910	0.1911	0.1865	0.1575
51510	0.2491	0.2455	0.1826	177110	0.1911	0.1863	0.1577
51710	0.2494	0.2457	0.1828	177310	0.1910	0.1863	0.1575

51910	0.2489	0.2450	0.1825	177510	0.1911	0.1866	0.1577
52110	0.2491	0.2450	0.1827	177710	0.1910	0.1863	0.1577
52310	0.2489	0.2451	0.1824	177910	0.1909	0.1862	0.1576
52510	0.2487	0.2450	0.1824	178110	0.1909	0.1863	0.1576
52710	0.2483	0.2443	0.1823	178310	0.1907	0.1860	0.1577
52910	0.2481	0.2440	0.1820	178510	0.1908	0.1860	0.1576
53110	0.2479	0.2436	0.1818	178710	0.1907	0.1859	0.1574
53310	0.2479	0.2438	0.1819	178910	0.1906	0.1858	0.1575
53510	0.2479	0.2436	0.1819	179110	0.1905	0.1858	0.1575
53710	0.2478	0.2435	0.1818	179310	0.1902	0.1856	0.1573
53910	0.2474	0.2431	0.1816	179510	0.1902	0.1857	0.1574
54110	0.2472	0.2430	0.1815	179710	0.1903	0.1856	0.1573
54310	0.2468	0.2427	0.1813	179910	0.1903	0.1854	0.1573
54510	0.2468	0.2425	0.1813	180110	0.1900	0.1853	0.1572
54710	0.2469	0.2423	0.1813	180310	0.1900	0.1853	0.1572
54910	0.2465	0.2422	0.1812	180510	0.1901	0.1852	0.1573
55110	0.2464	0.2422	0.1813	180710	0.1899	0.1853	0.1573
55310	0.2464	0.2421	0.1812	180910	0.1899	0.1852	0.1572
55510	0.2459	0.2416	0.1809	181110	0.1898	0.1851	0.1572
55710	0.2461	0.2417	0.1810	181310	0.1897	0.1852	0.1571
55910	0.2458	0.2415	0.1807	181510	0.1899	0.1851	0.1572
56110	0.2457	0.2412	0.1807	181710	0.1895	0.1847	0.1569
56310	0.2456	0.2412	0.1806	181910	0.1896	0.1846	0.1570
56510	0.2452	0.2409	0.1805	182110	0.1896	0.1847	0.1572
56710	0.2450	0.2405	0.1807	182310	0.1892	0.1848	0.1570
56910	0.2449	0.2405	0.1803	182510	0.1893	0.1846	0.1570
57110	0.2446	0.2402	0.1804	182710	0.1896	0.1846	0.1570
57310	0.2446	0.2401	0.1802	182910	0.1892	0.1845	0.1570
57510	0.2447	0.2402	0.1801	183110	0.1891	0.1842	0.1569
57710	0.2445	0.2401	0.1802	183310	0.1890	0.1843	0.1568
57910	0.2444	0.2399	0.1800	183510	0.1892	0.1845	0.1568
58110	0.2439	0.2394	0.1799	183710	0.1891	0.1842	0.1568
58310	0.2440	0.2396	0.1799	183910	0.1892	0.1841	0.1567
58510	0.2434	0.2393	0.1797	184110	0.1889	0.1841	0.1567
58710	0.2433	0.2393	0.1796	184310	0.1888	0.1839	0.1566
58910	0.2432	0.2391	0.1796	184510	0.1891	0.1840	0.1568
59110	0.2432	0.2390	0.1797	184710	0.1888	0.1839	0.1566
59310	0.2428	0.2392	0.1793	184910	0.1887	0.1838	0.1566
59510	0.2428	0.2385	0.1792	185110	0.1887	0.1837	0.1564
59710	0.2427	0.2385	0.1791	185310	0.1886	0.1838	0.1567
59910	0.2427	0.2384	0.1791	185510	0.1886	0.1839	0.1568
60110	0.2424	0.2381	0.1791	185710	0.1886	0.1838	0.1567
60310	0.2420	0.2380	0.1788	185910	0.1886	0.1835	0.1564
60510	0.2421	0.2378	0.1788	186110	0.1881	0.1833	0.1565
60710	0.2420	0.2377	0.1786	186310	0.1883	0.1833	0.1565
60910	0.2417	0.2377	0.1787	186510	0.1879	0.1830	0.1565
61110	0.2415	0.2376	0.1787	186710	0.1883	0.1831	0.1565
61310	0.2415	0.2374	0.1786	186910	0.1881	0.1833	0.1565
61510	0.2411	0.2370	0.1784	187110	0.1880	0.1832	0.1565
61710	0.2413	0.2371	0.1785	187310	0.1879	0.1830	0.1565
61910	0.2411	0.2369	0.1783	187510	0.1879	0.1830	0.1567
62110	0.2413	0.2371	0.1785	187710	0.1882	0.1832	0.1566
62310	0.2412	0.2366	0.1784	187910	0.1879	0.1828	0.1565
62510	0.2407	0.2366	0.1782	188110	0.1879	0.1829	0.1564

62710	0.2405	0.2362	0.1779	188310	0.1879	0.1831	0.1564
62910	0.2405	0.2362	0.1780	188510	0.1879	0.1831	0.1564
63110	0.2407	0.2362	0.1781	188710	0.1878	0.1829	0.1565
63310	0.2403	0.2362	0.1778	188910	0.1880	0.1830	0.1566
63510	0.2404	0.2361	0.1776	189110	0.1880	0.1829	0.1565
63710	0.2401	0.2356	0.1777	189310	0.1881	0.1830	0.1564
63910	0.2400	0.2355	0.1776	189510	0.1879	0.1829	0.1564
64110	0.2399	0.2354	0.1776	189710	0.1881	0.1830	0.1565
64310	0.2397	0.2351	0.1776	189910	0.1879	0.1829	0.1564
64510	0.2402	0.2353	0.1775	190110	0.1877	0.1828	0.1564
64710	0.2400	0.2351	0.1776	190310	0.1878	0.1825	0.1564
64910	0.2397	0.2348	0.1775	190510	0.1876	0.1823	0.1563
65110	0.2393	0.2345	0.1774	190710	0.1876	0.1823	0.1562
65310	0.2392	0.2345	0.1771	190910	0.1875	0.1824	0.1562
65510	0.2390	0.2343	0.1772	191110	0.1873	0.1822	0.1562
65710	0.2391	0.2340	0.1771	191310	0.1875	0.1822	0.1563
65910	0.2391	0.2343	0.1771	191510	0.1874	0.1821	0.1563
66110	0.2388	0.2340	0.1769	191710	0.1874	0.1823	0.1563
66310	0.2384	0.2336	0.1768	191910	0.1876	0.1822	0.1562
66510	0.2384	0.2333	0.1768	192110	0.1875	0.1819	0.1565
66710	0.2382	0.2332	0.1768	192310	0.1874	0.1821	0.1564
66910	0.2380	0.2333	0.1767	192510	0.1873	0.1820	0.1562
67110	0.2380	0.2331	0.1767	192710	0.1871	0.1818	0.1561
67310	0.2378	0.2331	0.1767	192910	0.1871	0.1817	0.1562
67510	0.2375	0.2330	0.1766	193110	0.1868	0.1816	0.1560
67710	0.2375	0.2327	0.1767	193310	0.1868	0.1816	0.1561
67910	0.2372	0.2324	0.1764	193510	0.1871	0.1817	0.1560
68110	0.2370	0.2322	0.1763	193710	0.1868	0.1818	0.1560
68310	0.2368	0.2319	0.1763	193910	0.1864	0.1815	0.1560
68510	0.2370	0.2321	0.1763	194110	0.1865	0.1814	0.1560
68710	0.2367	0.2320	0.1761	194310	0.1863	0.1813	0.1560
68910	0.2365	0.2318	0.1760	194510	0.1864	0.1812	0.1562
69110	0.2366	0.2316	0.1760	194710	0.1864	0.1815	0.1560
69310	0.2366	0.2317	0.1760	194910	0.1864	0.1814	0.1560
69510	0.2366	0.2315	0.1758	195110	0.1863	0.1815	0.1560
69710	0.2364	0.2311	0.1759	195310	0.1863	0.1812	0.1560
69910	0.2366	0.2314	0.1757	195510	0.1863	0.1813	0.1559
70110	0.2362	0.2310	0.1757	195710	0.1863	0.1816	0.1559
70310	0.2362	0.2309	0.1757	195910	0.1863	0.1816	0.1560
70510	0.2359	0.2307	0.1755	196110	0.1865	0.1815	0.1561
70710	0.2359	0.2308	0.1755	196310	0.1862	0.1810	0.1559
70910	0.2355	0.2304	0.1753	196510	0.1861	0.1811	0.1560
71110	0.2354	0.2306	0.1754	196710	0.1858	0.1812	0.1559
71310	0.2352	0.2303	0.1753	196910	0.1860	0.1812	0.1560
71510	0.2352	0.2301	0.1752	197110	0.1858	0.1813	0.1559
71710	0.2347	0.2297	0.1753	197310	0.1855	0.1812	0.1559
71910	0.2346	0.2299	0.1753	197510	0.1856	0.1810	0.1560
72110	0.2348	0.2298	0.1752	197710	0.1855	0.1811	0.1561
72310	0.2346	0.2295	0.1749	197910	0.1856	0.1808	0.1558
72510	0.2346	0.2296	0.1749	198110	0.1857	0.1808	0.1559
72710	0.2346	0.2294	0.1748	198310	0.1856	0.1806	0.1558
72910	0.2344	0.2294	0.1748	198510	0.1854	0.1808	0.1560
73110	0.2341	0.2288	0.1748	198710	0.1853	0.1806	0.1559
73310	0.2345	0.2290	0.1746	198910	0.1854	0.1806	0.1558

73510	0.2339	0.2286	0.1744	199110	0.1853	0.1807	0.1557
73710	0.2336	0.2284	0.1742	199310	0.1851	0.1803	0.1558
73910	0.2337	0.2284	0.1743	199510	0.1851	0.1806	0.1557
74110	0.2335	0.2284	0.1744	199710	0.1850	0.1805	0.1558
74310	0.2336	0.2282	0.1743	199910	0.1852	0.1805	0.1559
74510	0.2333	0.2281	0.1742	200110	0.1852	0.1806	0.1557
74710	0.2332	0.2281	0.1741	200310	0.1853	0.1807	0.1558
74910	0.2330	0.2278	0.1741	200510	0.1853	0.1805	0.1557
75110	0.2330	0.2277	0.1740	200710	0.1852	0.1807	0.1556
75310	0.2330	0.2278	0.1740	200910	0.1850	0.1805	0.1557
75510	0.2327	0.2276	0.1740	201110	0.1852	0.1804	0.1555
75710	0.2329	0.2278	0.1739	201310	0.1851	0.1805	0.1555
75910	0.2327	0.2274	0.1739	201510	0.1850	0.1803	0.1557
76110	0.2324	0.2273	0.1738	201710	0.1848	0.1801	0.1554
76310	0.2323	0.2273	0.1738	201910	0.1850	0.1801	0.1556
76510	0.2323	0.2272	0.1736	202110	0.1848	0.1800	0.1555
76710	0.2323	0.2270	0.1737	202310	0.1850	0.1802	0.1556
76910	0.2322	0.2269	0.1736	202510	0.1850	0.1803	0.1557
77110	0.2320	0.2268	0.1735	202710	0.1848	0.1803	0.1559
77310	0.2316	0.2263	0.1734	202910	0.1847	0.1800	0.1556
77510	0.2319	0.2262	0.1735	203110	0.1847	0.1801	0.1556
77710	0.2316	0.2263	0.1735	203310	0.1846	0.1798	0.1555
77910	0.2313	0.2260	0.1735	203510	0.1848	0.1797	0.1556
78110	0.2311	0.2257	0.1735	203710	0.1844	0.1796	0.1554
78310	0.2311	0.2255	0.1734	203910	0.1843	0.1797	0.1556
78510	0.2307	0.2255	0.1733	204110	0.1842	0.1796	0.1555
78710	0.2305	0.2258	0.1733	204310	0.1843	0.1795	0.1555
78910	0.2305	0.2252	0.1730	204510	0.1844	0.1796	0.1556
79110	0.2304	0.2252	0.1731	204710	0.1843	0.1796	0.1555
79310	0.2303	0.2252	0.1730	204910	0.1845	0.1796	0.1554
79510	0.2299	0.2248	0.1728	205110	0.1838	0.1792	0.1553
79710	0.2301	0.2247	0.1727	205310	0.1841	0.1794	0.1554
79910	0.2300	0.2244	0.1728	205510	0.1841	0.1796	0.1554
80110	0.2302	0.2245	0.1727	205710	0.1842	0.1794	0.1555
80310	0.2299	0.2244	0.1727	205910	0.1842	0.1794	0.1555
80510	0.2299	0.2245	0.1727	206110	0.1837	0.1792	0.1553
80710	0.2298	0.2244	0.1727	206310	0.1839	0.1793	0.1554
80910	0.2297	0.2243	0.1726	206510	0.1836	0.1791	0.1551
81110	0.2293	0.2241	0.1727	206710	0.1837	0.1790	0.1554
81310	0.2294	0.2241	0.1727	206910	0.1839	0.1793	0.1553
81510	0.2291	0.2239	0.1725	207110	0.1840	0.1789	0.1553
81710	0.2291	0.2236	0.1725	207310	0.1836	0.1788	0.1553
81910	0.2289	0.2235	0.1724	207510	0.1837	0.1788	0.1550
82110	0.2288	0.2235	0.1725	207710	0.1834	0.1787	0.1552
82310	0.2283	0.2232	0.1723	207910	0.1838	0.1790	0.1551
82510	0.2288	0.2232	0.1723	208110	0.1838	0.1787	0.1549
82710	0.2283	0.2230	0.1722	208310	0.1835	0.1784	0.1549
82910	0.2283	0.2227	0.1724	208510	0.1835	0.1786	0.1550
83110	0.2281	0.2226	0.1724	208710	0.1834	0.1786	0.1550
83310	0.2279	0.2226	0.1721	208910	0.1836	0.1787	0.1550
83510	0.2280	0.2226	0.1722	209110	0.1835	0.1785	0.1551
83710	0.2276	0.2224	0.1720	209310	0.1832	0.1786	0.1549
83910	0.2276	0.2219	0.1720	209510	0.1833	0.1781	0.1549
84110	0.2275	0.2221	0.1721	209710	0.1833	0.1782	0.1550

84310	0.2277	0.2220	0.1720	209910	0.1832	0.1785	0.1550
84510	0.2274	0.2218	0.1718	210110	0.1832	0.1781	0.1550
84710	0.2275	0.2217	0.1720	210310	0.1835	0.1783	0.1551
84910	0.2272	0.2217	0.1719	210510	0.1833	0.1781	0.1551
85110	0.2274	0.2217	0.1718	210710	0.1830	0.1781	0.1549
85310	0.2272	0.2215	0.1717	210910	0.1831	0.1781	0.1551
85510	0.2272	0.2215	0.1716	211110	0.1829	0.1780	0.1549
85710	0.2266	0.2212	0.1717	211310	0.1827	0.1779	0.1548
85910	0.2268	0.2214	0.1716	211510	0.1829	0.1781	0.1550
86110	0.2266	0.2213	0.1716	211710	0.1829	0.1779	0.1549
86310	0.2267	0.2212	0.1715	211910	0.1831	0.1780	0.1551
86510	0.2262	0.2211	0.1715	212110	0.1830	0.1779	0.1549
86710	0.2261	0.2209	0.1715	212310	0.1829	0.1778	0.1549
86910	0.2261	0.2208	0.1713	212510	0.1830	0.1779	0.1550
87110	0.2258	0.2208	0.1714	212710	0.1827	0.1775	0.1549
87310	0.2257	0.2204	0.1713	212910	0.1831	0.1780	0.1550
87510	0.2258	0.2205	0.1714	213110	0.1829	0.1779	0.1550
87710	0.2258	0.2206	0.1714	213310	0.1827	0.1776	0.1548
87910	0.2255	0.2203	0.1712	213510	0.1827	0.1777	0.1550
88110	0.2254	0.2203	0.1712	213710	0.1828	0.1778	0.1549
88310	0.2254	0.2204	0.1711	213910	0.1825	0.1777	0.1548
88510	0.2254	0.2204	0.1711	214110	0.1828	0.1776	0.1549
88710	0.2252	0.2200	0.1708	214310	0.1828	0.1777	0.1548
88910	0.2254	0.2204	0.1710	214510	0.1825	0.1776	0.1548
89110	0.2253	0.2202	0.1709	214710	0.1827	0.1774	0.1548
89310	0.2252	0.2199	0.1707	214910	0.1827	0.1775	0.1547
89510	0.2250	0.2198	0.1708	215110	0.1825	0.1775	0.1548
89710	0.2249	0.2195	0.1707	215310	0.1827	0.1775	0.1546
89910	0.2248	0.2192	0.1706	215510	0.1826	0.1773	0.1547
90110	0.2245	0.2194	0.1706	215710	0.1827	0.1776	0.1546
90310	0.2247	0.2192	0.1706	215910	0.1827	0.1777	0.1547
90510	0.2246	0.2192	0.1705	216110	0.1829	0.1778	0.1547
90710	0.2246	0.2191	0.1705	216310	0.1828	0.1776	0.1547
90910	0.2242	0.2190	0.1705	216510	0.1824	0.1774	0.1547
91110	0.2241	0.2191	0.1704	216710	0.1824	0.1774	0.1546
91310	0.2244	0.2190	0.1706	216910	0.1823	0.1774	0.1546
91510	0.2242	0.2187	0.1705	217110	0.1825	0.1774	0.1546
91710	0.2239	0.2188	0.1703	217310	0.1825	0.1772	0.1546
91910	0.2241	0.2188	0.1704	217510	0.1823	0.1770	0.1544
92110	0.2240	0.2188	0.1702	217710	0.1823	0.1771	0.1546
92310	0.2237	0.2185	0.1701	217910	0.1822	0.1771	0.1546
92510	0.2236	0.2182	0.1702	218110	0.1823	0.1771	0.1546
92710	0.2236	0.2182	0.1701	218310	0.1821	0.1767	0.1546
92910	0.2231	0.2180	0.1700	218510	0.1822	0.1770	0.1546
93110	0.2230	0.2179	0.1700	218710	0.1822	0.1771	0.1547
93310	0.2231	0.2178	0.1700	218910	0.1821	0.1768	0.1545
93510	0.2232	0.2179	0.1700	219110	0.1820	0.1770	0.1546
93710	0.2229	0.2176	0.1699	219310	0.1820	0.1769	0.1545
93910	0.2228	0.2175	0.1699	219510	0.1819	0.1770	0.1546
94110	0.2229	0.2174	0.1698	219710	0.1817	0.1766	0.1544
94310	0.2224	0.2171	0.1696	219910	0.1819	0.1768	0.1544
94510	0.2225	0.2169	0.1696	220110	0.1817	0.1766	0.1543
94710	0.2222	0.2169	0.1696	220310	0.1817	0.1762	0.1543
94910	0.2224	0.2168	0.1695	220510	0.1818	0.1767	0.1543

95110	0.2220	0.2165	0.1694	220710	0.1819	0.1766	0.1543
95310	0.2217	0.2164	0.1693	220910	0.1817	0.1764	0.1543
95510	0.2219	0.2162	0.1693	221110	0.1817	0.1763	0.1545
95710	0.2217	0.2161	0.1691	221310	0.1818	0.1763	0.1542
95910	0.2215	0.2161	0.1693	221510	0.1817	0.1764	0.1543
96110	0.2216	0.2164	0.1693	221710	0.1816	0.1762	0.1542
96310	0.2214	0.2161	0.1691	221910	0.1817	0.1763	0.1541
96510	0.2213	0.2160	0.1691	222110	0.1817	0.1763	0.1542
96710	0.2211	0.2158	0.1691	222310	0.1815	0.1763	0.1541
96910	0.2213	0.2159	0.1690	222510	0.1816	0.1761	0.1541
97110	0.2209	0.2157	0.1690	222710	0.1818	0.1763	0.1541
97310	0.2210	0.2156	0.1689	222910	0.1815	0.1762	0.1541
97510	0.2209	0.2158	0.1691	223110	0.1813	0.1763	0.1543
97710	0.2204	0.2151	0.1690	223310	0.1816	0.1764	0.1542
97910	0.2205	0.2151	0.1691	223510	0.1816	0.1762	0.1542
98110	0.2203	0.2149	0.1688	223710	0.1814	0.1762	0.1541
98310	0.2199	0.2146	0.1688	223910	0.1815	0.1764	0.1540
98510	0.2200	0.2146	0.1687	224110	0.1811	0.1759	0.1541
98710	0.2198	0.2145	0.1686	224310	0.1814	0.1759	0.1540
98910	0.2195	0.2144	0.1686	224510	0.1808	0.1756	0.1541
99110	0.2195	0.2144	0.1685	224710	0.1807	0.1755	0.1538
99310	0.2197	0.2143	0.1686	224910	0.1811	0.1757	0.1539
99510	0.2194	0.2144	0.1687	225110	0.1809	0.1758	0.1540
99710	0.2196	0.2141	0.1686	225310	0.1809	0.1757	0.1538
99910	0.2197	0.2141	0.1686	225510	0.1807	0.1756	0.1540
100110	0.2193	0.2138	0.1685	225710	0.1809	0.1756	0.1539
100310	0.2193	0.2138	0.1684	225910	0.1809	0.1758	0.1538
100510	0.2191	0.2137	0.1685	226110	0.1810	0.1756	0.1538
100710	0.2189	0.2132	0.1683	226310	0.1807	0.1758	0.1538
100910	0.2191	0.2135	0.1684	226510	0.1808	0.1757	0.1537
101110	0.2189	0.2132	0.1683	226710	0.1807	0.1756	0.1538
101310	0.2188	0.2129	0.1682	226910	0.1804	0.1753	0.1537
101510	0.2186	0.2132	0.1682	227110	0.1805	0.1757	0.1537
101710	0.2186	0.2129	0.1680	227310	0.1805	0.1756	0.1537
101910	0.2184	0.2130	0.1682	227510	0.1805	0.1756	0.1537
102110	0.2183	0.2129	0.1683	227710	0.1804	0.1753	0.1535
102310	0.2182	0.2129	0.1682	227910	0.1807	0.1754	0.1537
102510	0.2183	0.2126	0.1680	228110	0.1802	0.1752	0.1535
102710	0.2183	0.2129	0.1680	228310	0.1802	0.1752	0.1537
102910	0.2181	0.2126	0.1682	228510	0.1803	0.1753	0.1536
103110	0.2181	0.2125	0.1680	228710	0.1801	0.1752	0.1535
103310	0.2180	0.2123	0.1680	228910	0.1800	0.1749	0.1534
103510	0.2179	0.2127	0.1678	229110	0.1800	0.1751	0.1535
103710	0.2179	0.2126	0.1677	229310	0.1798	0.1749	0.1534
103910	0.2178	0.2124	0.1677	229510	0.1799	0.1751	0.1533
104110	0.2174	0.2122	0.1677	229710	0.1796	0.1749	0.1534
104310	0.2175	0.2121	0.1676	229910	0.1796	0.1749	0.1533
104510	0.2173	0.2118	0.1675	230110	0.1796	0.1749	0.1534
104710	0.2176	0.2121	0.1675	230310	0.1796	0.1749	0.1533
104910	0.2174	0.2120	0.1674	230510	0.1798	0.1748	0.1533
105110	0.2173	0.2121	0.1673	230710	0.1797	0.1750	0.1533
105310	0.2171	0.2117	0.1674	230910	0.1797	0.1750	0.1532
105510	0.2170	0.2117	0.1673	231110	0.1796	0.1748	0.1533
105710	0.2170	0.2118	0.1672	231310	0.1794	0.1747	0.1534

105910	0.2170	0.2117	0.1672
106110	0.2168	0.2115	0.1671
106310	0.2167	0.2113	0.1671
106510	0.2162	0.2111	0.1671
106710	0.2161	0.2111	0.1670
106910	0.2163	0.2111	0.1670
107110	0.2162	0.2112	0.1668
107310	0.2160	0.2108	0.1668
107510	0.2159	0.2108	0.1667
107710	0.2158	0.2107	0.1668
107910	0.2155	0.2105	0.1668
108110	0.2156	0.2105	0.1667
108310	0.2154	0.2103	0.1666
108510	0.2152	0.2101	0.1666
108710	0.2150	0.2101	0.1664
108910	0.2149	0.2101	0.1665
109110	0.2150	0.2102	0.1666
109310	0.2151	0.2101	0.1666
109510	0.2148	0.2100	0.1664
109710	0.2148	0.2097	0.1664
109910	0.2148	0.2098	0.1664
110110	0.2146	0.2098	0.1665
110310	0.2145	0.2095	0.1663
110510	0.2145	0.2098	0.1663
110710	0.2144	0.2096	0.1663
110910	0.2143	0.2092	0.1662
111110	0.2143	0.2093	0.1661
111310	0.2142	0.2094	0.1660
111510	0.2138	0.2092	0.1660
111710	0.2137	0.2090	0.1661
111910	0.2136	0.2090	0.1658
112110	0.2136	0.2087	0.1659
112310	0.2134	0.2086	0.1658
112510	0.2135	0.2087	0.1659
112710	0.2133	0.2086	0.1660
112910	0.2132	0.2085	0.1658
113110	0.2130	0.2083	0.1657
113310	0.2129	0.2083	0.1656
113510	0.2129	0.2080	0.1656
113710	0.2127	0.2079	0.1657
113910	0.2125	0.2077	0.1655
114110	0.2129	0.2080	0.1655
114310	0.2125	0.2077	0.1654
114510	0.2126	0.2078	0.1656
114710	0.2127	0.2075	0.1656
114910	0.2125	0.2076	0.1655
115110	0.2124	0.2074	0.1654
115310	0.2124	0.2072	0.1654
115510	0.2123	0.2071	0.1655
115710	0.2124	0.2071	0.1653
115910	0.2123	0.2073	0.1654
116110	0.2123	0.2072	0.1654
116310	0.2118	0.2069	0.1653
116510	0.2118	0.2068	0.1652

231510	0.1796	0.1748	0.1534
231710	0.1796	0.1745	0.1533
231910	0.1794	0.1744	0.1533
232110	0.1792	0.1742	0.1532
232310	0.1792	0.1744	0.1532
232510	0.1790	0.1743	0.1532
232710	0.1791	0.1742	0.1531
232910	0.1794	0.1744	0.1533
233110	0.1791	0.1741	0.1532
233310	0.1787	0.1740	0.1530
233510	0.1791	0.1741	0.1530
233710	0.1791	0.1739	0.1531
233910	0.1789	0.1740	0.1531
234110	0.1787	0.1739	0.1532
234310	0.1788	0.1739	0.1532
234510	0.1784	0.1737	0.1530
234710	0.1785	0.1737	0.1530
234910	0.1785	0.1736	0.1530
235110	0.1786	0.1736	0.1530
235310	0.1783	0.1735	0.1529
235510	0.1784	0.1737	0.1530
235710	0.1784	0.1735	0.1531
235910	0.1786	0.1736	0.1531
236110	0.1785	0.1736	0.1530
236310	0.1783	0.1732	0.1530
236510	0.1786	0.1736	0.1530
236710	0.1785	0.1735	0.1531
236910	0.1785	0.1735	0.1529
237110	0.1783	0.1734	0.1529
237310	0.1784	0.1734	0.1529
237510	0.1781	0.1733	0.1529
237710	0.1783	0.1733	0.1530
237910	0.1781	0.1733	0.1528
238110	0.1778	0.1731	0.1528
238310	0.1780	0.1731	0.1528
238510	0.1780	0.1732	0.1529
238710	0.1781	0.1731	0.1530
238910	0.1777	0.1729	0.1528
239110	0.1779	0.1731	0.1529
239310	0.1778	0.1728	0.1528
239510	0.1777	0.1728	0.1529
239710	0.1776	0.1726	0.1527
239910	0.1777	0.1726	0.1527
240110	0.1776	0.1727	0.1528
240310	0.1779	0.1726	0.1529
240510	0.1778	0.1726	0.1527
240710	0.1777	0.1725	0.1527
240910	0.1774	0.1723	0.1527
241110	0.1772	0.1721	0.1526
241310	0.1775	0.1723	0.1527
241510	0.1775	0.1721	0.1529
241710	0.1775	0.1723	0.1528
241910	0.1773	0.1723	0.1527
242110	0.1773	0.1720	0.1527

116710	0.2116	0.2066	0.1651
116910	0.2115	0.2066	0.1653
117110	0.2113	0.2064	0.1650
117310	0.2113	0.2063	0.1651
117510	0.2114	0.2060	0.1651
117710	0.2114	0.2061	0.1651
117910	0.2112	0.2060	0.1651
118110	0.2110	0.2058	0.1650
118310	0.2110	0.2058	0.1650
118510	0.2107	0.2058	0.1650
118710	0.2110	0.2061	0.1650
118910	0.2110	0.2057	0.1650
119110	0.2109	0.2058	0.1649
119310	0.2109	0.2058	0.1649
119510	0.2107	0.2057	0.1650
119710	0.2104	0.2055	0.1650
119910	0.2105	0.2056	0.1650
120110	0.2103	0.2054	0.1650
120310	0.2100	0.2051	0.1648
120510	0.2101	0.2053	0.1647
120710	0.2101	0.2051	0.1648
120910	0.2100	0.2048	0.1648
121110	0.2100	0.2050	0.1648
121310	0.2101	0.2049	0.1648
121510	0.2102	0.2050	0.1645
121710	0.2098	0.2048	0.1646
121910	0.2097	0.2048	0.1647
122110	0.2096	0.2049	0.1645
122310	0.2095	0.2049	0.1646
122510	0.2094	0.2046	0.1646
122710	0.2093	0.2047	0.1646
122910	0.2093	0.2044	0.1645
123110	0.2092	0.2042	0.1645
123310	0.2088	0.2038	0.1645
123510	0.2088	0.2043	0.1643
123710	0.2090	0.2044	0.1643
123910	0.2087	0.2041	0.1644

242310	0.1771	0.1720	0.1527
242510	0.1773	0.1719	0.1527
242710	0.1773	0.1722	0.1527
242910	0.1770	0.1717	0.1528
243110	0.1770	0.1717	0.1525
243310	0.1769	0.1716	0.1525
243510	0.1767	0.1717	0.1526
243710	0.1765	0.1713	0.1525
243910	0.1766	0.1715	0.1527
244110	0.1765	0.1712	0.1525
244310	0.1768	0.1715	0.1526
244510	0.1763	0.1715	0.1526
244710	0.1766	0.1714	0.1525
244910	0.1766	0.1715	0.1525
245110	0.1762	0.1711	0.1525
245310	0.1762	0.1712	0.1524
245510	0.1762	0.1711	0.1524
245710	0.1763	0.1713	0.1525
245910	0.1761	0.1709	0.1524
246110	0.1761	0.1709	0.1525
246310	0.1761	0.1710	0.1525
246510	0.1760	0.1712	0.1524
246710	0.1760	0.1708	0.1525
246910	0.1761	0.1708	0.1523
247110	0.1758	0.1708	0.1524
247310	0.1757	0.1706	0.1523
247510	0.1757	0.1704	0.1523
247710	0.1756	0.1704	0.1526
247910	0.1758	0.1705	0.1524
248110	0.1758	0.1706	0.1523
248310	0.1755	0.1704	0.1524
248510	0.1753	0.1703	0.1522
248710	0.1756	0.1705	0.1523
248910	0.1755	0.1702	0.1522
249110	0.1751	0.1701	0.1523
249310	0.1751	0.1704	0.1522
249510	0.1753	0.1706	0.1523
249710	0.175114	0.17045	0.152187

Supplementary Table 9. Gene list (496 genes) from the sweep region harboring the QTL associated with major traits.

No	Gene	Start : End	Size	Trait	QTLs	Annotation
1	Glyma02g10630	7939100:11465400	3526300	YIELD	Q_COLYLDIR_2-1	PTHR21726:SF16,PTHR21726 AT2G39435.1 Phosphatidylinositol N-acetyglucosaminlytransferase subunit P-related
2	Glyma02g10630	7939100:11465400	3526300	YIELD	Q_COLYLDIR_2-1	PTHR21726:SF16,PTHR21726 AT2G39435.1 Phosphatidylinositol N-acetyglucosaminlytransferase subunit P-related
3	Glyma02g10640	7939100:11465400	3526300	YIELD	Q_COLYLDIR_2-1	PF01423 PTHR23338:SF18,PTHR23338 KOG3428 AT4G02840.1 Small nuclear ribonucleoprotein family protein
4	Glyma02g10660	7939100:11465400	3526300	YIELD	Q_COLYLDIR_2-1	PF00462 PTHR10168 KOG1752 GO:0009055,GO:0015035,GO:0045454 AT5G14070.1 ROXY2 Thioredoxin superfamily protein
5	Glyma02g10690	7939100:11465400	3526300	YIELD	Q_COLYLDIR_2-1	PF01842 PTHR13734,PTHR13734:SF23 GO:0016597,GO:0008152 AT1G69040.2 ACR4 ACT domain repeat 4
6	Glyma02g10710	7939100:11465400	3526300	YIELD	Q_COLYLDIR_2-1	PF02781,PF00479 PTHR23429 KOG0563 1.1.1.49 K00036 GO:0004345,GO:0050661,GO:0006006,GO:0055114 AT5G13110.1 G6PD2 glucose-6-phosphate dehydrogenase 2
7	Glyma02g10720	7939100:11465400	3526300	YIELD	Q_COLYLDIR_2-1	
8	Glyma03g26060	3699336:4200251	500915	YIELD	Q_COLYLDIR_5-2	PF02298 GO:0005507,GO:0009055 AT2G32300.1 UCC1 uclacyanin 1
9	Glyma03g26080	3699336:4200251	500915	YIELD	Q_COLYLDIR_5-2	PF00573 PTHR10746 KOG1624 GO:0003735,GO:0006412,GO:0005840 AT1G07320.1 RPL4 ribosomal protein L4
10	Glyma03g26540	3699336:4200251	500915	YIELD	Q_COLYLDIR_5-2	AT4G37930.1 SHM1,SHMT1,STM serine transhydroxymethyltransferase 1
11	Glyma03g26550	3699336:4200251	500915	YIELD	Q_COLYLDIR_5-2	AT3G20810.2 2-oxoglutarate (2OG) and Fe(II)-dependent oxygenase superfamily protein
12	Glyma03g26580	3699336:4200251	500915	YIELD	Q_COLYLDIR_5-2	PF00179 PTHR24067:SF8,PTHR24067 KOG0896 GO:0016881 AT3G52560.1 MMZ4,UEV1D,UEV1D-4 ubiquitin E2 variant 1D-4
13	Glyma03g26590	3699336:4200251	500915	YIELD	Q_COLYLDIR_5-2	PF00106 PTHR24322 KOG0725 GO:0016491,GO:0008152 AT3G51680.1 NAD(P)-binding Rossmann-fold superfamily protein
14	Glyma03g30640	3699336:4200251	500915	YIELD	Q_COLYLDIR_5-2	PF03171 KOG4176 GO:0016491,GO:0016706,GO:0055114 AT4G02940.1 oxidoreductase, 2OG-Fe(II) oxygenase family protein
15	Glyma03g30650	3699336:4200251	500915	YIELD	Q_COLYLDIR_5-2	
16	Glyma03g30660	3699336:4200251	500915	YIELD	Q_COLYLDIR_5-2	AT2G48090.1
17	Glyma03g30673	3699336:4200251	500915	YIELD	Q_COLYLDIR_5-2	AT2G42190.1
18	Glyma03g30686	3699336:4200251	500915	YIELD	Q_COLYLDIR_5-2	PF00225 PTHR24115:SF176,PTHR24115 GO:0003777,GO:0005524,GO:0007018 AT2G21380.1 Kinesin motor family protein
19	Glyma03g30700	3699336:4200251	500915	YIELD	Q_COLYLDIR_5-2	
20	Glyma05g04250	3183520:4168520	985000	WHOLE-PLANT	Pl ht 24-3	PF02365 GO:0003677,GO:0006355 AT1G12260.1 ANAC007,EMB2749,NAC007,VND4 NAC 7
21	Glyma05g04260	3183520:4168520	985000	WHOLE-PLANT	Pl ht 24-3	PF04690 AT2G45190.1 AFO,FIL,YAB1 Plant-specific transcription factor YABBY family protein
22	Glyma05g04270	3183520:4168520	985000	WHOLE-PLANT	Pl ht 24-3	PF07732,PF00394,PF07731 PTHR11709,PTHR11709:SF18 KOG1263 GO:0016491,GO:0055114,GO:0005507 AT4G12420.1 SKU5 Cupredoxin superfamily protein
23	Glyma05g04610	3183520:4168520	985000	WHOLE-PLANT	Pl ht 24-3	
24	Glyma05g04630	3183520:4168520	985000	WHOLE-PLANT	Pl ht 24-3	PF01501 PTHR11183:SF5,PTHR11183 GO:0016757 AT4G16600.1 Nucleotide-diphospho-sugar transferases superfamily protein
25	Glyma05g04650	3183520:4168520	985000	WHOLE-PLANT	Pl ht 24-3	PF04614 PTHR12774 KOG3133 K13337 GO:0005777 AT5G17550.1 AtPEX19-2,PEX19-2 peroxin 19-2
26	Glyma05g04660	3183520:4168520	985000	WHOLE-PLANT	Pl ht 24-3	PF00152 PTHR22594:SF6,PTHR22594 KOG0554 6.1.1.22 K01893 GO:0000166,GO:0004812,GO:0005524,GO:0006418,GO:0005737,GO:0006412 AT4G17300.1 ATNS1,NS1,OVA8 Class II aminoacyl-tRNA and biotin synthetases superfamily
27	Glyma05g04670	3183520:4168520	985000	WHOLE-PLANT	Pl ht 24-3	PF04758 PTHR12650 KOG0009 K02983 GO:0003735,GO:0006412,GO:0005622,GO:0005840 AT5G56670.1 Ribosomal protein S30 family protein

28	Glyma05g04680	3183520:4168520	985000	WHOLE-PLANT	Pl ht 24-3	
29	Glyma05g04690	3183520:4168520	985000	WHOLE-PLANT	Pl ht 24-3	PTHR11389,PTHR11389:SF378 AT3G60580.1 C2H2-like zinc finger protein
30	Glyma05g04700	3183520:4168520	985000	WHOLE-PLANT	Pl ht 24-3	PF00892 GO:0016020 AT5G47470.1 Nodulin MtN21 /EamA-like transporter family protein
31	Glyma05g04720	3183520:4168520	985000	WHOLE-PLANT	Pl ht 24-3	PF10250 AT4G16650.1 O-fucosyltransferase family protein
32	Glyma05g04740	3183520:4168520	985000	WHOLE-PLANT	Pl ht 24-3	PF04784 PTHR23054 AT5G47380.1 Protein of unknown function, DUF547
33	Glyma05g04750	3183520:4168520	985000	WHOLE-PLANT	Pl ht 24-3	PF00551 PTHR10520,PTHR10520:SF7 KOG3076 3.5.1.10 K01433 GO:0016742,GO:0009058,GO:0008864,GO:0006189 AT5G47435.1 formyltetrahydrofolate deformylase, putative
34	Glyma05g04760	3183520:4168520	985000	WHOLE-PLANT	Pl ht 24-3	PF05703,PF08458 PTHR24730:SF7,PTHR24730 AT4G17350.1 Plant protein of unknown function (DUF828) with plant pleckstrin homology-like region
35	Glyma05g04770	3183520:4168520	985000	WHOLE-PLANT	Pl ht 24-3	PF01501 PTHR11183:SF7,PTHR11183 KOG1950 GO:0016757 AT3G18660.2 GUX1,PGSIP1 plant glycogenin-like starch initiation protein 1
36	Glyma05g04840	3183520:4168520	985000	WHOLE-PLANT	Pl ht 24-3	AT4G16695.1
37	Glyma05g04860	3183520:4168520	985000	WHOLE-PLANT	Pl ht 24-3	PF02894,PF01408 PTHR22604:SF14,PTHR22604 KOG2741 GO:0016491,GO:0008152,GO:0055114 AT4G17370.1 Oxidoreductase family protein
38	Glyma05g04870	3183520:4168520	985000	WHOLE-PLANT	Pl ht 24-3	PF00827 PTHR11847 KOG1678 K02877 GO:0003735,GO:0006412,GO:0005840,GO:0005622 AT4G16720.1 Ribosomal protein L23/L15e family protein
39	Glyma05g04880	3183520:4168520	985000	WHOLE-PLANT	Pl ht 24-3	PF08783 PTHR15439 KOG0314 GO:0008270,GO:0005634 AT5G47430.1 DWNN domain, a CCHC-type zinc finger
40	Glyma05g04900	3183520:4168520	985000	WHOLE-PLANT	Pl ht 24-3	PF00249 PTHR10641 KOG0048 GO:0003677 AT3G13540.1 ATMYB5,MYB5 myb domain protein 5
41	Glyma05g04910	3183520:4168520	985000	WHOLE-PLANT	Pl ht 24-3	AT5G47400.1
42	Glyma05g04920	3183520:4168520	985000	WHOLE-PLANT	Pl ht 24-3	PF00847 GO:0003700,GO:0006355 AT2G44940.1 Integrase-type DNA-binding superfamily protein
43	Glyma05g04940	3183520:4168520	985000	WHOLE-PLANT	Pl ht 24-3	PF01756,PF02770,PF00441 PTHR10909:SF11,PTHR10909 KOG0136 1.3.3.6 K00232 GO:0016627,GO:0055114,GO:0003997,GO:0006635,GO:0005777,GO:0003995 AT4G16760.1 ACX1,ATACX1 acyl-CoA oxidase 1
44	Glyma05g04950	3183520:4168520	985000	WHOLE-PLANT	Pl ht 24-3	PF00249 PTHR12374 GO:0003677 AT5G47390.1 myb-like transcription factor family protein
45	Glyma05g05520	4925000:5122000	197000	POD	Pod mat 16-1	PF00244 PTHR18860 GO:0019904 AT4G09000.1 GF14 CHI,GRF1 general regulatory factor 1
46	Glyma05g05540	4925000:5122000	197000	POD	Pod mat 16-1	PF00069 PTHR24343:SF58,PTHR24343 KOG0583 GO:0004672,GO:0005524,GO:0006468 AT4G33950.1 ATOST1,OST1,P44,SNRK2-6,SNRK2.6,SRK2E Protein kinase superfamily protein
47	Glyma05g05580	4925000:5122000	197000	POD	Pod mat 16-1	PTHR23056,PTHR23056:SF16 KOG0034 K06268 AT5G47100.1 ATCBL9,CBL9 calcineurin B-like protein 9
48	Glyma05g05610	4925000:5122000	197000	POD	Pod mat 16-1	PF09747 KOG3044 AT5G47090.1
49	Glyma05g05620	4925000:5122000	197000	POD	Pod mat 16-1	PF01214 PTHR11740 KOG3092 GO:0019887,GO:0005956 AT5G47080.1 CKB1 casein kinase II beta chain 1
50	Glyma05g05650	4925000:5122000	197000	POD	Pod mat 16-1	PF01214 PTHR11740 KOG3092 K03115 GO:0019887,GO:0005956 AT2G44680.1 CKB4 casein kinase II beta subunit 4
51	Glyma05g05660	4925000:5122000	197000	POD	Pod mat 16-1	ATMG00030.1
52	Glyma05g14740	14775000:19109000	4334000	WHOLE-PLANT	CWP 1-1	PF01165 GO:0003735,GO:0006412,GO:0005622,GO:0005840 AT3G27160.1 GHS1 Ribosomal protein S21 family protein
53	Glyma05g14760	14775000:19109000	4334000	WHOLE-PLANT	CWP 1-1	PF00654,PF00571 PTHR11689:SF11,PTHR11689 KOG0474 GO:0005515,GO:0005247,GO:0006821,GO:0055085,GO:0016020 AT3G27170.1 ATCLC-B,CLC-B chloride channel B
54	Glyma05g14770	14775000:19109000	4334000	WHOLE-PLANT	CWP 1-1	PF00675 PTHR11851,PTHR11851:SF34 GO:0004222,GO:0006508 AT5G42390.1 Insulinase (Peptidase family M16) family protein
55	Glyma05g14780	14775000:19109000	4334000	WHOLE-PLANT	CWP 1-1	PF05617 AT1G57775.1 Protein of unknown function (DUF784)

56	Glyma05g14800	14775000:19109000	4334000	WHOLE-PLANT	CWP 1-1	PF02298 GO:0005507,GO:0009055 AT3G27200.1 Cupredoxin superfamily protein
57	Glyma05g14810	14775000:19109000	4334000	WHOLE-PLANT	CWP 1-1	
58	Glyma05g14820	14775000:19109000	4334000	WHOLE-PLANT	CWP 1-1	PF03140 AT3G50180.1 Plant protein of unknown function (DUF247)
59	Glyma05g14850	14775000:19109000	4334000	WHOLE-PLANT	CWP 1-1	PF00011 PTHR11527,PTHR11527:SF14 AT1G53540.1 HSP20-like chaperones superfamily protein
60	Glyma05g14860	14775000:19109000	4334000	WHOLE-PLANT	CWP 1-1	PF03140 AT3G50120.1 Plant protein of unknown function (DUF247)
61	Glyma05g14870	14775000:19109000	4334000	WHOLE-PLANT	CWP 1-1	AT3G01860.1
62	Glyma05g15100	14775000:19109000	4334000	WHOLE-PLANT	CWP 1-1	PF04935 PTHR14369 KOG2885 AT5G05210.1 Surfeit locus protein 6
63	Glyma05g15130	14775000:19109000	4334000	WHOLE-PLANT	CWP 1-1	PF00012 PTHR19375:SF1,PTHR19375 GO:0005524 AT5G28540.1 BIP1 heat shock protein 70 (Hsp 70) family protein
64	Glyma05g15150	14775000:19109000	4334000	WHOLE-PLANT	CWP 1-1	PF00560,PF07714 PTHR24420,PTHR24420:SF465 KOG1187 GO:0004672,GO:0005524,GO:0006468,GO:0005515 AT5G14210.1 Leucine-rich repeat protein kinase family protein
65	Glyma05g15170	14775000:19109000	4334000	WHOLE-PLANT	CWP 1-1	PF02536 PTHR13068 KOG1267 AT4G14605.1 Mitochondrial transcription termination factor family protein
66	Glyma05g21411	14775000:19109000	4334000	WHOLE-PLANT	CWP 1-2	
67	Glyma07g12180	7683000:11623000	3940000	WHOLE-PLANT	Cnpy ht 2-1	PF01554 PTHR11206:SF38,PTHR11206 KOG1347 GO:0015238,GO:0015297,GO:0006855,GO:0055085,GO:0016020 AT4G29140.1 MATE efflux family protein
68	Glyma07g12190	7683000:11623000	3940000	WHOLE-PLANT	Cnpy ht 2-1	PF03727,PF00349 PTHR19443 KOG1369 2.7.1.1 K00844 GO:0005524,GO:0016773,GO:0005975 AT4G29130.1 ATHXK1,GIN2,HXK1 hexokinase 1
69	Glyma07g12210	7683000:11623000	3940000	WHOLE-PLANT	Cnpy ht 2-1	PF03171 PTHR10209,PTHR10209:SF55 KOG0143 GO:0016491,GO:0016706,GO:0055114 AT3G13610.1 2-oxoglutarate (2OG) and Fe(II)-dependent oxygenase superfamily protein
70	Glyma07g12250	7683000:11623000	3940000	WHOLE-PLANT	Cnpy ht 2-1	AT1G05350.1 NAD(P)-binding Rossmann-fold superfamily protein
71	Glyma07g12280	7683000:11623000	3940000	WHOLE-PLANT	Cnpy ht 2-1	PF03987 PTHR12866 KOG4741 AT3G07525.1 ATATG10,ATG10 autophagocytosis-associated family protein
72	Glyma07g12340	7683000:11623000	3940000	WHOLE-PLANT	Cnpy ht 2-1	PF00749 PTHR10119:SF1,PTHR10119 KOG1149 6.1.1.17 K01885 GO:0000166,GO:0005524,GO:0016876,GO:0043039,GO:0005737,GO:0006412 AT5G64050.1 ATERS,ERS,OVA3 glutamate tRNA synthetase
73	Glyma07g12380	7683000:11623000	3940000	WHOLE-PLANT	Cnpy ht 2-1	AT5G17610.1
74	Glyma07g12400	7683000:11623000	3940000	WHOLE-PLANT	Cnpy ht 2-1	PTHR11639,PTHR11639:SF43 AT1G64850.1 Calcium-binding EF hand family protein
75	Glyma07g12960	7683000:11623000	3940000	WHOLE-PLANT	Cnpy ht 2-1	PF10536 AT1G17930.1 Aminotransferase-like, plant mobile domain family protein
76	Glyma07g12970	7683000:11623000	3940000	WHOLE-PLANT	Cnpy ht 2-1	PF12600 AT2G44640.1
77	Glyma07g12980	7683000:11623000	3940000	WHOLE-PLANT	Cnpy ht 2-1	PF00166 PTHR10772:SF0,PTHR10772 KOG1641 GO:0006457,GO:0005737 AT3G60210.1 GroES-like family protein
78	Glyma07g12990	7683000:11623000	3940000	WHOLE-PLANT	Cnpy ht 2-1	PTHR14155:SF2,PTHR14155 AT3G60220.1 ATL4,TL4 TOXICOS EN LEVADURA 4
79	Glyma07g13005	7683000:11623000	3940000	WHOLE-PLANT	Cnpy ht 2-1	PF03153 PTHR12694:SF2,PTHR12694 GO:0006367,GO:0005672,GO:0003702 AT1G07480.1 Transcription factor IIA, alpha/beta subunit
80	Glyma07g13020	7683000:11623000	3940000	WHOLE-PLANT	Cnpy ht 2-1	AT4G26760.1 MAP65-2 microtubule-associated protein 65-2
81	Glyma07g13040	7683000:11623000	3940000	WHOLE-PLANT	Cnpy ht 2-1	PF00637,PF10367,PF10366,PF00780 PTHR12894:SF4,PTHR12894 KOG2063 GO:0006886,GO:0016192,GO:0005083 AT1G22860.1 Vacuolar sorting protein 39

82	Glyma07g13070	7683000:11623000	3940000	WHOLE-PLANT	Cnpy ht 2-1	AT1G02180.1 ferredoxin-related
83	Glyma07g13100	7683000:11623000	3940000	WHOLE-PLANT	Cnpy ht 2-1	PF03171 PTHR10209,PTHR10209:SF55 KOG0143 GO:0016491,GO:0016706,GO:0055114 AT5G59540.1 2-oxoglutarate (2OG) and Fe(II)-dependent oxygenase superfamily protein
84	Glyma07g13120	7683000:11623000	3940000	WHOLE-PLANT	Cnpy ht 2-1	PF00637,PF12451 PTHR23323:SF24,PTHR23323 KOG2114 GO:0006886,GO:0016192 AT2G05170.1 ATVPS11,VPS11 vacuolar protein sorting 11
85	Glyma07g13130	7683000:11623000	3940000	WHOLE-PLANT	Cnpy ht 2-1	PF00201 PTHR11926:SF15,PTHR11926 KOG1192 GO:0016758,GO:0008152 AT4G01070.1 GT72B1,UGT72B1 UDP-Glycosyltransferase superfamily protein
86	Glyma09g08070	3370670:8049420	4678750	OTHER-SEED	Sd cadmium 1-1	PF00583 PTHR23091:SF22,PTHR23091 GO:0008080,GO:0016747,GO:0008152 AT2G06025.1 Acyl-CoA N-acyltransferases (NAT) superfamily protein
87	Glyma09g08080	3370670:8049420	4678750	OTHER-SEED	Sd cadmium 1-1	PF03133 PTHR12241:SF13,PTHR12241 GO:0004835,GO:0006464 AT1G77550.1 tubulin-tyrosine ligases;tubulin-tyrosine ligases
88	Glyma09g08090	3370670:8049420	4678750	OTHER-SEED	Sd cadmium 1-1	AT2G06005.1 FIP1 FRIGIDA interacting protein 1
89	Glyma09g08100	3370670:8049420	4678750	OTHER-SEED	Sd cadmium 1-1	PF08246,PF00112 PTHR12411:SF50,PTHR12411 KOG1543 3.4.22.16 K01366 GO:0008234,GO:0006508,GO:0004197 AT3G45310.1 Cysteine proteinases superfamily protein
90	Glyma09g15980	17336000:20094000	2758000	OTHER-SEED	Glycitein 2-4	PF03372 PTHR12121,PTHR12121:SF20 KOG0620 3.1.-.- K12603 AT3G58580.1 DNAse I-like superfamily protein
91	Glyma10g24430	19798500:39380300	19581800	YIELD	Q_COLYLDIR_10	PF00957 PTHR21136,PTHR21136:SF46 KOG0859 K08511 GO:0016192,GO:0016021 AT1G04760.1 ATVAMP726,VAMP726 vesicle-associated membrane protein 726
92	Glyma10g28500	19798500:39380300	19581800	YIELD	Q_COLYLDIR_10	PF02773,PF00438,PF02772 PTHR11964 KOG1506 2.5.1.6 K00789 GO:0004478,GO:0006556,GO:0005524 AT4G01850.1 AtSAM2,MAT2,SAM-2,SAM2 S-adenosylmethionine synthetase 2
93	Glyma10g28530	19798500:39380300	19581800	YIELD	Q_COLYLDIR_10	PF00069 PTHR24057 KOG0658 2.7.1.- K00924 GO:0004672,GO:0005524,GO:0006468 AT5G26751.1 ATSK11,SK 11 shaggy-related kinase 11
94	Glyma10g28540	19798500:39380300	19581800	YIELD	Q_COLYLDIR_10	PF00642,PF06220 PTHR16465 GO:0003676,GO:0008270 AT5G26749.1 C2H2 and C2HC zinc fingers superfamily protein
95	Glyma10g28560	19798500:39380300	19581800	YIELD	Q_COLYLDIR_10	PF01470 PTHR23402 KOG4755 GO:0006508 AT1G56700.1 Peptidase C15, pyroglutamyl peptidase I-like
96	Glyma10g28600	19798500:39380300	19581800	YIELD	Q_COLYLDIR_10	PTHR12847 KOG2355 K12608 AT5G02270.1 ATNAP9,NAP9 non-intrinsic ABC protein 9
97	Glyma10g28610	19798500:39380300	19581800	YIELD	Q_COLYLDIR_10	PF01501 PTHR11183,PTHR11183:SF2 GO:0016757 AT2G47180.1 AtGolS1,GolS1 galactinol synthase 1
98	Glyma10g28620	19798500:39380300	19581800	YIELD	Q_COLYLDIR_10	PF05832 PTHR13019 KOG3195 GO:0016021 AT1G09330.1
99	Glyma10g28640	19798500:39380300	19581800	YIELD	Q_COLYLDIR_10	PF04853 AT1G56560.1 Plant neutral invertase family protein
100	Glyma10g28650	19798500:39380300	19581800	YIELD	Q_COLYLDIR_10	PTHR10870 GO:0008853,GO:0006281,GO:0005634,GO:0003684 AT4G17760.1 damaged DNA binding;exodeoxyribonuclease IIIs
101	Glyma10g28680	19798500:39380300	19581800	YIELD	Q_COLYLDIR_10	PF04398 AT1G09310.1 Protein of unknown function, DUF538
102	Glyma10g28690	19798500:39380300	19581800	YIELD	Q_COLYLDIR_10	PF00557,PF05195 PTHR10804,PTHR10804:SF8 KOG2414 3.4.11.9 K01262 GO:0009987,GO:0004177,GO:0030145 AT1G09300.1 Metallopeptidase M24 family protein
103	Glyma10g28700	19798500:39380300	19581800	YIELD	Q_COLYLDIR_10	PF03959 PTHR18838,PTHR18838:SF15 KOG2551 GO:0016787 AT1G09280.1
104	Glyma10g28710	19798500:39380300	19581800	YIELD	Q_COLYLDIR_10	
105	Glyma10g28730	19798500:39380300	19581800	YIELD	Q_COLYLDIR_10	PF09597 AT5G26800.1
106	Glyma10g28740	19798500:39380300	19581800	YIELD	Q_COLYLDIR_10	AT1G09250.1 basic helix-loop-helix (bHLH) DNA-binding superfamily protein
107	Glyma10g28760	19798500:39380300	19581800	YIELD	Q_COLYLDIR_10	PF06963 PTHR11660:SF1,PTHR11660 KOG2601 GO:0005381,GO:0034755,GO:0016021 AT5G26820.1 ATIREG3,IREG3,MAR1,RTS3 iron-regulated protein 3
108	Glyma10g28800	19798500:39380300	19581800	YIELD	Q_COLYLDIR_10	PF00515 PTHR22904:SF95,PTHR22904 GO:0005515 AT1G56440.1 Tetratricopeptide repeat (TPR)-like superfamily protein
109	Glyma10g28810	19798500:39380300	19581800	YIELD	Q_COLYLDIR_10	PTHR12444 KOG1877 AT5G26850.1 Uncharacterized protein
110	Glyma10g28820	19798500:39380300	19581800	YIELD	Q_COLYLDIR_10	PF03790,PF00046,PF03789,PF03791 PTHR11850:SF45,PTHR11850 KOG0773 GO:0003700,GO:0043565,GO:0006355,GO:0003677,GO:0005634 AT1G23380.1 KNAT6,KNAT6L,KNAT6S KNOTTED1-like homeobox gene 6
111	Glyma10g28830	19798500:39380300	19581800	YIELD	Q_COLYLDIR_10	AT1G56423.1

112	Glyma10g28840	19798500:39380300	19581800	YIELD	Q_COLYLDIR_10	PF00004,PF02190,PF05362 PTHR10046,PTHR10046:SF23 KOG2004 3.4.21.- K08675 GO:0005524,GO:0004176,GO:0006508,GO:0004252,GO:0009378,GO:0006281,GO:0006310,GO:0016887 AT5G26860.1 LON1,LON_ARA_ARA lon protease 1
113	Glyma10g29050	19798500:39380300	19581800	YIELD	Q_COLYLDIR_10	PF00225,PF00307 PTHR24115,PTHR24115:SF135 KOG0239 GO:0003777,GO:0005524,GO:0007018,GO:0005515 AT5G27000.1 ATK4,KATD kinesin 4
114	Glyma10g29060	19798500:39380300	19581800	YIELD	Q_COLYLDIR_10	PF00481 PTHR13832:SF148,PTHR13832 KOG0698 GO:0003824 AT1G09160.1 Protein phosphatase 2C family protein
115	Glyma10g29070	19798500:39380300	19581800	YIELD	Q_COLYLDIR_10	PF00436 PTHR10302 KOG1653 GO:0003697,GO:0006260 AT4G11060.1 MTSSB mitochondrially targeted single-stranded DNA binding protein
116	Glyma10g29080	19798500:39380300	19581800	YIELD	Q_COLYLDIR_10	PF03371 PTHR23142 KOG2889 AT2G40650.1 PRP38 family protein
117	Glyma10g29090	19798500:39380300	19581800	YIELD	Q_COLYLDIR_10	PF00400 PTHR22847,PTHR22847:SF52 KOG0266 GO:0005515 AT5G27030.1 TPR3 TOPLESS-related 3
118	Glyma10g29580	19798500:39380300	19581800	YIELD	Q_COLYLDIR_10	PF08356,PF08477,PF08355 PTHR24072,PTHR24072:SF12 KOG1707 GO:0005525,GO:0007264,GO:0005622 AT5G27540.1 emb2473,MIRO1 MIRO-related GTP-ase 1
119	Glyma10g29590	19798500:39380300	19581800	YIELD	Q_COLYLDIR_10	PF04893 PTHR12822 KOG3114 GO:0016020 AT5G27490.1 Integral membrane Yip1 family protein
120	Glyma10g29600	19798500:39380300	19581800	YIELD	Q_COLYLDIR_10	PF00587,PF02403 PTHR11778 KOG2509 6.1.1.11 K01875 GO:0000166,GO:0004812,GO:0005524,GO:0006418,GO:0005737,GO:0004828,GO:0006434,GO:0006412 AT5G27470.1 seryl-tRNA synthetase / serine--tRNA ligase
121	Glyma10g29610	19798500:39380300	19581800	YIELD	Q_COLYLDIR_10	AT1G54920.2
122	Glyma10g29620	19798500:39380300	19581800	YIELD	Q_COLYLDIR_10	AT3G06035.1 Glycoprotein membrane precursor GPI-anchored
123	Glyma10g29630	19798500:39380300	19581800	YIELD	Q_COLYLDIR_10	PF00106 PTHR24315,PTHR24315:SF77 KOG0725 GO:0016491,GO:0008152 AT1G54870.1 NAD(P)-binding Rossmann-fold superfamily protein
124	Glyma10g29640	19798500:39380300	19581800	YIELD	Q_COLYLDIR_10	PF01425 PTHR11895:SF6,PTHR11895 KOG1211 GO:0016884 AT1G08980.1 AMI1,ATAMI1,ATTOC64-I,TOC64-I amidase 1
125	Glyma10g29650	19798500:39380300	19581800	YIELD	Q_COLYLDIR_10	PF00097 PTHR12456,PTHR12456:SF2 AT3G05250.1 RING/U-box superfamily protein
126	Glyma10g31970	19798500:39380300	19581800	YIELD	Q_COLYLDIR_10	PF00856 PTHR13271 KOG1337 GO:0005515 AT3G56570.1 SET domain-containing protein
127	Glyma10g37100	44778100:59592500	14814400	YIELD	Q_CLAYLDRF_10-1	PF00076 PTHR24011,PTHR24011:SF180 GO:0003676 AT1G21320.2 nucleotide binding;nucleic acid binding
128	Glyma10g37110	44778100:59592500	14814400	YIELD	Q_CLAYLDRF_10-1	PF00415,PF01363,PF08381 PTHR22870,PTHR22870:SF25 KOG1427 GO:0046872 AT5G42140.1 Regulator of chromosome condensation (RCC1) family with FYVE zinc finger
129	Glyma10g37120	44778100:59592500	14814400	YIELD	Q_CLAYLDRF_10-1	PF00069,PF00139 PTHR24420,PTHR24420:SF261 KOG1187 GO:0004672,GO:0005524,GO:0006468,GO:0005488 AT5G42120.1 Concanavalin A-like lectin protein kinase family protein
130	Glyma10g37130	44778100:59592500	14814400	YIELD	Q_CLAYLDRF_10-1	AT1G43770.2 RING/FYVE/PHD zinc finger superfamily protein
131	Glyma10g37140	44778100:59592500	14814400	YIELD	Q_CLAYLDRF_10-1	AT1G43770.2 RING/FYVE/PHD zinc finger superfamily protein
132	Glyma10g37150	44778100:59592500	14814400	YIELD	Q_CLAYLDRF_10-1	PF00005,PF00664 PTHR24223:SF21,PTHR24223 KOG0054 GO:0005524,GO:0016887,GO:0042626,GO:0006810,GO:0055085,GO:0016021 AT3G59140.1 ATMRP14,MRP14 multidrug resistance-associated protein 14
133	Glyma10g37160	44778100:59592500	14814400	YIELD	Q_CLAYLDRF_10-1	PF00664,PF00005 PTHR24223:SF21,PTHR24223 KOG0054 GO:0005524,GO:0016887,GO:0042626,GO:0006810,GO:0055085,GO:0016021 AT3G59140.1 ATMRP14,MRP14 multidrug resistance-associated protein 14
134	Glyma10g37180	44778100:59592500	14814400	YIELD	Q_CLAYLDRF_10-1	PF00282 PTHR11999,PTHR11999:SF4 KOG0629 GO:0016831,GO:0030170,GO:0019752 AT1G43710.1 emb1075 Pyridoxal phosphate (PLP)-dependent transferases superfamily protein
135	Glyma10g37200	44778100:59592500	14814400	YIELD	Q_CLAYLDRF_10-1	PF00704 3.2.1.14 K01183 GO:0004553,GO:0005975 AT5G24090.1 ATCHIA,CHIA chitinase A
136	Glyma10g37210	44778100:59592500	14814400	YIELD	Q_CLAYLDRF_10-1	PF00224,PF02887 PTHR11817 KOG2323 GO:0000287,GO:0004743,GO:0030955,GO:0006096 AT5G52920.1 PKP-BETA1,PKP1,PKP2 plastidic pyruvate kinase beta subunit 1
137	Glyma10g37220	44778100:59592500	14814400	YIELD	Q_CLAYLDRF_10-1	PF04821,PF05029 PTHR22940,PTHR22940:SF4 KOG1974 K03155 AT5G52910.1 ATIM timeless family protein
138	Glyma10g37230	44778100:59592500	14814400	YIELD	Q_CLAYLDRF_10-1	PF08263,PF00560 PTHR24420:SF474,PTHR24420 KOG0472 GO:0005515 AT2G34930.1 disease resistance family protein / LRR family protein
139	Glyma10g37250	44778100:59592500	14814400	YIELD	Q_CLAYLDRF_10-1	PF00560,PF08263 PTHR24420 KOG0472 GO:0005515 AT2G34930.1 disease resistance family protein / LRR family protein

140	Glyma10g37260	44778100:59592500	14814400	YIELD	Q_CLAYLDRF_10-1	PF00560,PF08263 PTHR24420,PTHR24420:SF473 KOG0472 GO:0005515 AT2G34930.1 disease resistance family protein / LRR family protein
141	Glyma10g37290	44778100:59592500	14814400	YIELD	Q_CLAYLDRF_10-1	PF00560,PF08263 PTHR24420 KOG0472 GO:0005515 AT2G34930.1 disease resistance family protein / LRR family protein
142	Glyma10g37300	44778100:59592500	14814400	YIELD	Q_CLAYLDRF_10-1	PF00560,PF08263 PTHR24420,PTHR24420:SF473 KOG4237 GO:0005515 AT2G34930.1 disease resistance family protein / LRR family protein
143	Glyma10g37310	44778100:59592500	14814400	YIELD	Q_CLAYLDRF_10-1	PF00400 PTHR13083 KOG1333 GO:0005515 AT3G49660.1 Transducin/WD40 repeat-like superfamily protein
144	Glyma10g37320	44778100:59592500	14814400	YIELD	Q_CLAYLDRF_10-1	PF08263,PF00560 PTHR24420,PTHR24420:SF473 KOG0472 GO:0005515 AT2G34930.1 disease resistance family protein / LRR family protein
145	Glyma10g37350	44778100:59592500	14814400	YIELD	Q_CLAYLDRF_10-1	PF02517 GO:0016020 AT5G60750.1 CAAX amino terminal protease family protein
146	Glyma10g37360	44778100:59592500	14814400	YIELD	Q_CLAYLDRF_10-1	AT5G52900.1
147	Glyma10g37370	44778100:59592500	14814400	YIELD	Q_CLAYLDRF_10-1	PF03595 GO:0055085,GO:0016021 AT5G24030.1 SLAH3 SLAC1 homologue 3
148	Glyma10g37380	44778100:59592500	14814400	YIELD	Q_CLAYLDRF_10-1	PF00004 PTHR23074:SF20,PTHR23074 KOG0737 GO:0005524,GO:0009378,GO:0006281,GO:0006310 AT5G52882.1 P-loop containing nucleoside triphosphate hydrolases superfamily protein
149	Glyma10g37390	44778100:59592500	14814400	YIELD	Q_CLAYLDRF_10-1	PF00646 GO:0005515 AT5G52880.1 F-box family protein
150	Glyma10g37400	44778100:59592500	14814400	YIELD	Q_CLAYLDRF_10-1	PF02453 PTHR10994:SF27,PTHR10994 KOG1792 GO:0005783 AT4G23630.1 BTI1,RTNLB1 VIRB2-interacting protein 1
151	Glyma10g37410	44778100:59592500	14814400	YIELD	Q_CLAYLDRF_10-1	AT5G52870.1
152	Glyma10g37420	44778100:59592500	14814400	YIELD	Q_CLAYLDRF_10-1	PF00005,PF01061 PTHR19241:SF103,PTHR19241 KOG0061 GO:0005524,GO:0016887,GO:0016020 AT5G52860.1 ABC-2 type transporter family protein
153	Glyma10g37430	44778100:59592500	14814400	YIELD	Q_CLAYLDRF_10-1	PF07690,PF03105 PTHR24003,PTHR24003:SF230 KOG2325 GO:0022857,GO:0055085,GO:0016021 AT4G22990.2 Major Facilitator Superfamily with SPX (SYG1/Pho81/XPR1) domain-containing protein
154	Glyma10g37450	44778100:59592500	14814400	YIELD	Q_CLAYLDRF_10-1	PF01535 PTHR24015,PTHR24015:SF131 AT5G52850.1 Pentatricopeptide repeat (PPR) superfamily protein
155	Glyma10g37460	44778100:59592500	14814400	YIELD	Q_CLAYLDRF_10-1	PF03106 GO:0003700,GO:0043565,GO:0006355 AT5G52830.1 ATWRKY27,WRKY27 WRKY DNA-binding protein 27
156	Glyma10g37470	44778100:59592500	14814400	YIELD	Q_CLAYLDRF_10-1	PF02423 PTHR13812:SF2,PTHR13812 KOG3007 AT5G52810.1 NAD(P)-binding Rossmann-fold superfamily protein
157	Glyma10g37480	44778100:59592500	14814400	YIELD	Q_CLAYLDRF_10-1	AT3G61920.1
158	Glyma10g37490	44778100:59592500	14814400	YIELD	Q_CLAYLDRF_10-1	PF00403 PTHR22814 KOG1603 GO:0046872,GO:0030001 AT1G01490.1 Heavy metal transport/detoxification superfamily protein
159	Glyma10g37500	44778100:59592500	14814400	YIELD	Q_CLAYLDRF_10-1	PF00403 PTHR22814 KOG1603 GO:0046872,GO:0030001 AT1G01490.1 Heavy metal transport/detoxification superfamily protein
160	Glyma10g37510	44778100:59592500	14814400	YIELD	Q_CLAYLDRF_10-1	PF00403 PTHR22814:SF56,PTHR22814 KOG1603 GO:0046872,GO:0030001 AT1G01490.1 Heavy metal transport/detoxification superfamily protein
161	Glyma10g37520	44778100:59592500	14814400	YIELD	Q_CLAYLDRF_10-1	PF00249 PTHR12802:SF30,PTHR12802 KOG0724 GO:0003677 AT5G52660.1 Homeodomain-like superfamily protein
162	Glyma10g37530	44778100:59592500	14814400	YIELD	Q_CLAYLDRF_10-1	PF00295 GO:0004650,GO:0005975 AT3G61490.1 Pectin lyase-like superfamily protein
163	Glyma10g37540	44778100:59592500	14814400	YIELD	Q_CLAYLDRF_10-1	PF00295 GO:0004650,GO:0005975 AT3G48950.1 Pectin lyase-like superfamily protein
164	Glyma10g37560	44778100:59592500	14814400	YIELD	Q_CLAYLDRF_10-1	PF04116 GO:0005506,GO:0016491,GO:0006633,GO:0055114 AT4G25700.1 B1,BCH1,BETA-OHASE 1,chy1 beta-hydroxylase 1
165	Glyma10g37571	44778100:59592500	14814400	YIELD	Q_CLAYLDRF_10-1	AT4G25670.1
166	Glyma10g37580	44778100:59592500	14814400	YIELD	Q_CLAYLDRF_10-1	PF05903 PTHR12378 KOG0324 AT4G25680.1 PPPDE putative thiol peptidase family protein
167	Glyma10g37590	44778100:59592500	14814400	YIELD	Q_CLAYLDRF_10-1	PF11721,PF00069 PTHR24420:SF550,PTHR24420 KOG1187 GO:0004672,GO:0005524,GO:0006468 AT5G24010.1 Protein kinase superfamily protein
168	Glyma10g37600	44778100:59592500	14814400	YIELD	Q_CLAYLDRF_10-1	PF01794,PF08022,PF08030 PTHR11972,PTHR11972:SF3 KOG0039 GO:0005506,GO:0009055,GO:0016491,GO:0050660,GO:0016021,GO:0055114 AT5G23980.1 ATFRO4,FRO4 ferric reduction oxidase 4
169	Glyma10g37610	44778100:59592500	14814400	YIELD	Q_CLAYLDRF_10-1	PF01794,PF08030,PF08022 PTHR11972:SF3,PTHR11972 GO:0005506,GO:0009055,GO:0016491,GO:0050660,GO:0016021,GO:0055114 AT1G01580.1 ATFRO2,FRD1,FRO2 ferric reduction oxidase 2

170	Glyma10g37620	44778100:59592500	14814400	YIELD	Q_CLAYLDRF_10-1	
171	Glyma10g37630	44778100:59592500	14814400	YIELD	Q_CLAYLDRF_10-1	
172	Glyma10g37640	44778100:59592500	14814400	YIELD	Q_CLAYLDRF_10-1	PF03514 AT5G52510.1 SCL8 SCARECROW-like 8
173	Glyma10g37660	44778100:59592500	14814400	YIELD	Q_CLAYLDRF_10-1	PF01554 PTHR11206:SF49,PTHR11206 KOG1347 GO:0015238,GO:0015297,GO:0006855,GO:0055085,GO:0016020 AT4G25640.2 ATDTX35,DTX35,FFT detoxifying efflux carrier 35
174	Glyma10g37690	44778100:59592500	14814400	YIELD	Q_CLAYLDRF_10-1	PF02416 GO:0008565,GO:0015031 AT5G52440.1 HCF106 Bacterial sec-independent translocation protein mttA/Hcf106
175	Glyma10g37700	44778100:59592500	14814400	YIELD	Q_CLAYLDRF_10-1	AT5G52430.1 hydroxyproline-rich glycoprotein family protein
176	Glyma10g37710	44778100:59592500	14814400	YIELD	Q_CLAYLDRF_10-1	PF03600 PTHR10283:SF40,PTHR10283 KOG2639 GO:0015105,GO:0016021,GO:0015137,GO:0015746,GO:0055085,GO:0015385,GO:0006814 AT1G02260.1 Divalent ion symporter
177	Glyma10g37720	44778100:59592500	14814400	YIELD	Q_CLAYLDRF_10-1	PF01479,PF00163 PTHR11831,PTHR11831:SF1 KOG4655 K14560 GO:0019843,GO:0005622,GO:0003723 AT5G15750.1 Alpha-L RNA-binding motif/Ribosomal protein S4 family protein
178	Glyma10g37730	44778100:59592500	14814400	YIELD	Q_CLAYLDRF_10-1	PF00069 PTHR24361:SF208,PTHR24361 KOG0198 GO:0004672,GO:0005524,GO:0006468 AT1G63700.1 EMB71,MAPKKK4,YDA Protein kinase superfamily protein
179	Glyma10g37740	44778100:59592500	14814400	YIELD	Q_CLAYLDRF_10-1	
180	Glyma10g37750	44778100:59592500	14814400	YIELD	Q_CLAYLDRF_10-1	PF00106 PTHR24320 KOG1208 GO:0016491,GO:0008152,GO:0003824,GO:0050662,GO:0044237 AT4G23430.2 NAD(P)-binding Rossmann-fold superfamily protein
181	Glyma10g37760	44778100:59592500	14814400	YIELD	Q_CLAYLDRF_10-1	PF00106 PTHR24320 KOG1208 GO:0016491,GO:0008152,GO:0003824,GO:0050662,GO:0044237 AT4G23420.1 NAD(P)-binding Rossmann-fold superfamily protein
182	Glyma10g37770	44778100:59592500	14814400	YIELD	Q_CLAYLDRF_10-1	AT5G52420.1
183	Glyma10g37780	44778100:59592500	14814400	YIELD	Q_CLAYLDRF_10-1	AT4G25610.1 C2H2-like zinc finger protein
184	Glyma10g37810	44778100:59592500	14814400	YIELD	Q_CLAYLDRF_10-1	AT3G49055.1
185	Glyma10g37820	44778100:59592500	14814400	YIELD	Q_CLAYLDRF_10-1	PF03893,PF01764 PTHR21493,PTHR21493:SF1 KOG2088 GO:0004806,GO:0006629,GO:0004091,GO:0016042 AT3G49050.1 alpha/beta-Hydrolases superfamily protein
186	Glyma10g37840	44778100:59592500	14814400	YIELD	Q_CLAYLDRF_10-1	PF01158 PTHR10114 KOG3452 K02920 GO:0003735,GO:0006412,GO:0005622,GO:0005840 AT5G02450.1 Ribosomal protein L36e family protein
187	Glyma10g37860	44778100:59592500	14814400	YIELD	Q_CLAYLDRF_10-1	PF02042,PF00564 GO:0005515 AT1G64530.1 Plant regulator RWP-RK family protein
188	Glyma10g37870	44778100:59592500	14814400	YIELD	Q_CLAYLDRF_10-1	PF04258 PTHR12174:SF22,PTHR12174 KOG2443 3.4.23.- K09598 GO:0004190,GO:0016021 AT4G33410.1 ATSPPL1,SPPL1 SIGNAL PEPTIDE PEPTIDASE-LIKE 1
189	Glyma10g37890	44778100:59592500	14814400	YIELD	Q_CLAYLDRF_10-1	PF03283 PTHR21562,PTHR21562:SF1 KOG4287 AT5G23870.3 Pectinacetylesterase family protein
190	Glyma10g37900	44778100:59592500	14814400	YIELD	Q_CLAYLDRF_10-1	PF00082,PF12580 PTHR10795:SF2,PTHR10795 KOG1114 3.4.14.10 K01280 GO:0004252,GO:0006508 AT4G20850.1 TPP2 tripeptidyl peptidase ii
191	Glyma10g37910	44778100:59592500	14814400	YIELD	Q_CLAYLDRF_10-1	PF00067 PTHR24282,PTHR24282:SF174 KOG0157 GO:0005506,GO:0009055,GO:0016705,GO:0020037,GO:0055114 AT5G52400.1 CYP715A1 cytochrome P450, family 715, subfamily A, polypeptide 1
192	Glyma10g37920	44778100:59592500	14814400	YIELD	Q_CLAYLDRF_10-1	PF00067 PTHR24282,PTHR24282:SF174 KOG0157 GO:0005506,GO:0009055,GO:0016705,GO:0020037,GO:0055114 AT5G52400.1 CYP715A1 cytochrome P450, family 715, subfamily A, polypeptide 1
193	Glyma10g38770	44778100:59592500	14814400	YIELD	Q_CLAYLDRF_10-1	PF02170,PF08699,PF02171 PTHR22891 KOG1041 K11596 GO:0005515 AT5G43810.1 AGO10,PNH,ZLL Stabilizer of iron transporter SufD / Polynucleotidyl transferase
194	Glyma10g38790	44778100:59592500	14814400	YIELD	Q_CLAYLDRF_10-1	AT1G02205.3 CER1 Fatty acid hydroxylase superfamily
195	Glyma10g38810	44778100:59592500	14814400	YIELD	Q_CLAYLDRF_10-1	PF00069,PF00560 PTHR24359,PTHR24359:SF89 KOG0192 GO:0004672,GO:0005524,GO:0006468,GO:0005515 AT1G04210.1 Leucine-rich repeat protein kinase family protein
196	Glyma10g38820	44778100:59592500	14814400	YIELD	Q_CLAYLDRF_10-1	AT2G32980.1
197	Glyma10g38830	44778100:59592500	14814400	YIELD	Q_CLAYLDRF_10-1	PF08423 PTHR22942,PTHR22942:SF13 KOG1434 K10872 GO:0003697,GO:0005524,GO:0006281,GO:0009432 AT3G22880.1 ARLIM15,ATDMC1,DMC1 DNA repair (Rad51) family protein
198	Glyma10g38840	44778100:59592500	14814400	YIELD	Q_CLAYLDRF_10-1	PF12504,PF12481 PTHR11772 AT3G22850.1 Aluminium induced protein with YGL and LRDR motifs
199	Glyma10g38861	44778100:59592500	14814400	YIELD	Q_CLAYLDRF_10-1	PF00069,PF02138,PF00400 PTHR13743 KOG1786 GO:0004672,GO:0005524,GO:0006468,GO:0005515 AT5G18525.1 protein serine/threonine kinases;protein tyrosine kinases;ATP binding;protein kinases

200	Glyma10g38880	44778100:59592500	14814400	YIELD	Q_CLAYLDRF_10-1	PF01105 PTHR22811:SF32,PTHR22811 KOG1692 GO:0006810,GO:0016021 AT3G22845.1 emp24/gp25L/p24 family/GOLD family protein
201	Glyma10g38890	44778100:59592500	14814400	YIELD	Q_CLAYLDRF_10-1	PF00248 PTHR11732:SF12,PTHR11732 KOG1575 GO:0055114,GO:0016491 AT1G60710.1 ATB2 NAD(P)-linked oxidoreductase superfamily protein
202	Glyma10g38900	44778100:59592500	14814400	YIELD	Q_CLAYLDRF_10-1	PF00248 PTHR11732:SF12,PTHR11732 KOG1575 GO:0055114,GO:0016491 AT1G60710.1 ATB2 NAD(P)-linked oxidoreductase superfamily protein
203	Glyma10g38910	44778100:59592500	14814400	YIELD	Q_CLAYLDRF_10-1	PF00504 PTHR14154,PTHR14154:SF1 AT3G22840.1 ELIP,ELIP1 Chlorophyll A-B binding family protein
204	Glyma10g38920	44778100:59592500	14814400	YIELD	Q_CLAYLDRF_10-1	PF09742 PTHR12895,PTHR12895:SF1 KOG2225 AT1G04200.1
205	Glyma10g38930	44778100:59592500	14814400	YIELD	Q_CLAYLDRF_10-1	PF00447 PTHR10015,PTHR10015:SF7 KOG0627 GO:0003700,GO:0043565,GO:0006355,GO:0005634 AT3G22830.1 AT-HSFA6B,HSFA6B heat shock transcription factor A6B
206	Glyma10g38940	44778100:59592500	14814400	YIELD	Q_CLAYLDRF_10-1	PF03079 PTHR23418 KOG2107 1.13.11.53,1.13.11.54 K08967 GO:0010309,GO:0055114 AT4G14710.1 ATARD2 RmlC-like cupins superfamily protein
207	Glyma10g38950	44778100:59592500	14814400	YIELD	Q_CLAYLDRF_10-1	PF12204 AT4G38225.3
208	Glyma10g38960	44778100:59592500	14814400	YIELD	Q_CLAYLDRF_10-1	PF03079 PTHR23418 KOG2107 GO:0006355,GO:0010309,GO:0055114 AT5G43850.1 ARD4,ATARD4 RmlC-like cupins superfamily protein
209	Glyma10g38970	44778100:59592500	14814400	YIELD	Q_CLAYLDRF_10-1	PF09425,PF06200 GO:0005515 AT4G14720.1 PPD2,TIFY4B TIFY domain/Divergent CCT motif family protein
210	Glyma10g38980	44778100:59592500	14814400	YIELD	Q_CLAYLDRF_10-1	AT4G14723.1
211	Glyma10g38990	44778100:59592500	14814400	YIELD	Q_CLAYLDRF_10-1	PF05703,PF08458 PTHR24730:SF7,PTHR24730 AT3G22810.1 Plant protein of unknown function (DUF828) with plant pleckstrin homology-like region
212	Glyma10g39010	44778100:59592500	14814400	YIELD	Q_CLAYLDRF_10-1	AT4G14746.1
213	Glyma10g39020	44778100:59592500	14814400	YIELD	Q_CLAYLDRF_10-1	PF00097 AT2G15580.1 RING/U-box superfamily protein
214	Glyma10g39030	44778100:59592500	14814400	YIELD	Q_CLAYLDRF_10-1	PF00612 PTHR10699,PTHR10699:SF9 GO:0005515 AT4G14750.1 IQD19 IQ-domain 19
215	Glyma10g39050	44778100:59592500	14814400	YIELD	Q_CLAYLDRF_10-1	PF01015 PTHR11830 KOG1628 K02984 GO:0003735,GO:0006412,GO:0005622,GO:0005840 AT3G04840.1 Ribosomal protein S3Ae
216	Glyma10g39060	44778100:59592500	14814400	YIELD	Q_CLAYLDRF_10-1	PF00583 GO:0008080,GO:0008152 AT2G32020.1 Acyl-CoA N-acyltransferases (NAT) superfamily protein
217	Glyma10g39070	44778100:59592500	14814400	YIELD	Q_CLAYLDRF_10-1	PF07765 PTHR13140:SF97,PTHR13140 KOG0962 AT3G22790.1 Kinase interacting (KIP1-like) family protein
218	Glyma10g39080	44778100:59592500	14814400	YIELD	Q_CLAYLDRF_10-1	PF03638 PTHR12446:SF4,PTHR12446 KOG1171 AT4G14770.1 ATTCX2,TCX2 TESMIN/TSO1-like CXC 2
219	Glyma10g39100	44778100:59592500	14814400	YIELD	Q_CLAYLDRF_10-1	PF07540,PF03914 PTHR14428:SF5,PTHR14428 KOG2153 AT1G79150.1 binding
220	Glyma10g39110	44778100:59592500	14814400	YIELD	Q_CLAYLDRF_10-1	PF02469 PTHR10900:SF23,PTHR10900 AT5G60490.1 FLA12 FASCICLIN-like arabinogalactan-protein 12
221	Glyma10g39121	44778100:59592500	14814400	YIELD	Q_CLAYLDRF_10-1	PF01301 PTHR23421,PTHR23421:SF31 GO:0004553,GO:0005975,GO:0004565,GO:0009341 AT4G26140.1 BGAL12 beta-galactosidase 12
222	Glyma10g39130	44778100:59592500	14814400	YIELD	Q_CLAYLDRF_10-1	PF03372 PTHR11200:SF29,PTHR11200 KOG0565 AT2G32010.1 CVL1 CVP2 like 1
223	Glyma10g39150	44778100:59592500	14814400	YIELD	Q_CLAYLDRF_10-1	PF00190 GO:0045735 AT3G22640.1 PAP85 cupin family protein
224	Glyma10g39170	44778100:59592500	14814400	YIELD	Q_CLAYLDRF_10-1	PF00190 GO:0045735 AT3G22640.1 PAP85 cupin family protein
225	Glyma10g39190	44778100:59592500	14814400	YIELD	Q_CLAYLDRF_10-1	PF00234 AT3G22620.1 Bifunctional inhibitor/lipid-transfer protein/seed storage 2S albumin superfamily protein
226	Glyma10g39200	44778100:59592500	14814400	YIELD	Q_CLAYLDRF_10-1	AT3G22600.1 Bifunctional inhibitor/lipid-transfer protein/seed storage 2S albumin superfamily protein
227	Glyma10g39210	44778100:59592500	14814400	YIELD	Q_CLAYLDRF_10-1	PF04570 AT3G22550.1 Protein of unknown function (DUF581)
228	Glyma10g39220	44778100:59592500	14814400	YIELD	Q_CLAYLDRF_10-1	PF03092 PTHR24003:SF372,PTHR24003 KOG3574 GO:0055085,GO:0016021 AT2G32040.1 Major facilitator superfamily protein
229	Glyma10g39230	44778100:59592500	14814400	YIELD	Q_CLAYLDRF_10-1	PF07911 AT3G22540.1 Protein of unknown function (DUF1677)
230	Glyma10g39240	44778100:59592500	14814400	YIELD	Q_CLAYLDRF_10-1	PF04622 GO:0000247,GO:0006696,GO:0005783 AT1G05440.1 C-8 sterol isomerases
231	Glyma10g39250	44778100:59592500	14814400	YIELD	Q_CLAYLDRF_10-1	AT3G22530.1
232	Glyma10g39270	44778100:59592500	14814400	YIELD	Q_CLAYLDRF_10-1	PF04927 AT3G22490.1 Seed maturation protein

233	Glyma10g39290	44778100:59592500	14814400	YIELD	Q_CLAYLDRF_10-1	PF01535 PTHR24015:SF62,PTHR24015 AT4G14850.1 LOI1,MEF11 Pentatricopeptide repeat (PPR) superfamily protein
234	Glyma10g39320	44778100:59592500	14814400	YIELD	Q_CLAYLDRF_10-1	PF00291 PTHR10314,PTHR10314:SF8 KOG1252 GO:0003824,GO:0030170,GO:0008152 AT4G14880.1 ATCYS-3A,CYTACS1,OASA1,OLD3 O-acetylserine (thiol) lyase (OAS-TL) isoform A1
235	Glyma10g39330	44778100:59592500	14814400	YIELD	Q_CLAYLDRF_10-1	PF07899 AT3G22440.1 FRIGIDA-like protein
236	Glyma10g39350	44778100:59592500	14814400	YIELD	Q_CLAYLDRF_10-1	
237	Glyma10g39370	44778100:59592500	14814400	YIELD	Q_CLAYLDRF_10-1	PF00475 PTHR23133,PTHR23133:SF2 GO:0004424,GO:0000105 AT3G22425.2 HISN5A,IGPD imidazoleglycerol-phosphate dehydratase
238	Glyma10g39390	44778100:59592500	14814400	YIELD	Q_CLAYLDRF_10-1	PF00069 PTHR13902:SF38,PTHR13902 KOG0584 GO:0004672,GO:0005524,GO:0006468 AT3G04910.1 ATWNK1,WNK1,ZIK4 with no lysine (K) kinase 1
239	Glyma11g13800	9136860:11605270	2468410	WHOLE-PLANT	Pl ht 20-1	PF00232 PTHR10353 KOG0626 3.2.1.21 K01188 GO:0004553,GO:0005975 AT5G44640.1 BGLU13 beta glucosidase 13
240	Glyma11g13810	9136860:11605270	2468410	WHOLE-PLANT	Pl ht 20-1	PF00232 PTHR10353 KOG0626 GO:0004553,GO:0005975 AT2G44480.1 BGLU17 beta glucosidase 17
241	Glyma11g13820	9136860:11605270	2468410	WHOLE-PLANT	Pl ht 20-1	PF00232 PTHR10353 KOG0626 GO:0004553,GO:0005975 AT5G44640.1 BGLU13 beta glucosidase 13
242	Glyma11g13830	9136860:11605270	2468410	WHOLE-PLANT	Pl ht 20-1	PF00232 PTHR10353 KOG0626 GO:0004553,GO:0005975 AT5G44640.1 BGLU13 beta glucosidase 13
243	Glyma11g13850	9136860:11605270	2468410	WHOLE-PLANT	Pl ht 20-1	PF00232 PTHR10353 KOG0626 GO:0004553,GO:0005975 AT2G44480.1 BGLU17 beta glucosidase 17
244	Glyma11g13870	9136860:11605270	2468410	WHOLE-PLANT	Pl ht 20-1	PF00305,PF01477 PTHR11771:SF2,PTHR11771 GO:0016702,GO:0046872,GO:0055114,GO:0005515 AT3G45140.1 ATLOX2,LOX2 lipoxygenase 2
245	Glyma11g13880	9136860:11605270	2468410	WHOLE-PLANT	Pl ht 20-1	PF01477,PF00305 PTHR11771:SF2,PTHR11771 1.13.11.12 K00454 GO:0016702,GO:0046872,GO:0055114,GO:0005515 AT3G45140.1 ATLOX2,LOX2 lipoxygenase 2
246	Glyma11g13890	9136860:11605270	2468410	WHOLE-PLANT	Pl ht 20-1	PF00168,PF08372 PTHR10024:SF97,PTHR10024 GO:0005515 AT3G57880.1 Calcium-dependent lipid-binding (CaLB domain) plant phosphoribosyltransferase family protein
247	Glyma11g13920	9136860:11605270	2468410	WHOLE-PLANT	Pl ht 20-1	AT5G12900.1
248	Glyma12g17360	16548000:17927000	1379000	OTHER-SEED	Daidzein 2-2	PF00954,PF01453,PF07714,PF08276 PTHR24420,PTHR24420:SF703 KOG1187 GO:0004672,GO:0005524,GO:0006468,GO:0048544,GO:0005529 AT4G27290.1 S-locus lectin protein kinase family protein
249	Glyma12g17390	16548000:17927000	1379000	OTHER-SEED	Daidzein 2-2	PF01397,PF03936 GO:0010333,GO:0016829,GO:0008152,GO:0000287 AT5G23960.2 ATTPS21,TPS21 terpene synthase 21
250	Glyma12g17450	16548000:17927000	1379000	OTHER-SEED	Daidzein 2-2	PF00954,PF07714,PF08276,PF01453 PTHR24420:SF703,PTHR24420 KOG1187 GO:0004672,GO:0005524,GO:0006468,GO:0048544,GO:0005529 AT4G27290.1 S-locus lectin protein kinase family protein
251	Glyma12g20890	22379200:25629700	3250500	YIELD	Q_COLYLDRF_12	PF07714,PF00954,PF08276,PF01453 PTHR24420:SF703,PTHR24420 KOG1187 GO:0004672,GO:0005524,GO:0006468,GO:0048544,GO:0005529 AT4G27290.1 S-locus lectin protein kinase family protein
252	Glyma12g20960	22379200:25629700	3250500	YIELD	Q_COLYLDRF_12	PTHR10366:SF35,PTHR10366 AT3G53520.4 ATUXS1,UXS1 UDP-glucuronic acid decarboxylase 1
253	Glyma12g21030	22379200:25629700	3250500	YIELD	Q_COLYLDRF_12	PF00954,PF08276,PF01453,PF07714 PTHR24420,PTHR24420:SF703 KOG1187 GO:0004672,GO:0005524,GO:0006468,GO:0048544,GO:0005529 AT4G27290.1 S-locus lectin protein kinase family protein
254	Glyma12g21040	22379200:25629700	3250500	YIELD	Q_COLYLDRF_12	PF00954,PF00069,PF08276 PTHR24420,PTHR24420:SF703 KOG1187 GO:0004672,GO:0005524,GO:0006468,GO:0048544 AT4G27290.1 S-locus lectin protein kinase family protein
255	Glyma12g21070	22379200:25629700	3250500	YIELD	Q_COLYLDRF_12	PTHR10782:SF4,PTHR10782 AT5G60410.2 ATSIZ1,SIZ1 DNA-binding protein with MIZ/SP-RING zinc finger, PHD-finger and SAP domain
256	Glyma12g21090	22379200:25629700	3250500	YIELD	Q_COLYLDRF_12	PF00954,PF00069,PF11883,PF08276,PF01453 PTHR24420,PTHR24420:SF703 KOG1187 GO:0004672,GO:0005524,GO:0006468,GO:0048544,GO:0005529,GO:0004674 AT4G27290.1 S-locus lectin protein kinase family protein
257	Glyma12g21110	22379200:25629700	3250500	YIELD	Q_COLYLDRF_12	PF08276,PF01453,PF00954,PF07714 PTHR24420,PTHR24420:SF703 KOG1187 GO:0004672,GO:0005524,GO:0006468,GO:0048544,GO:0005529 AT4G27290.1 S-locus lectin protein kinase family protein

258	Glyma12g21130	22379200:25629700	3250500	YIELD	Q_COLYLDRF_12	PF01612 PTHR13620 GO:0003676,GO:0008408,GO:0006139,GO:0005622 AT2G36110.1 Polynucleotidyl transferase, ribonuclease H-like superfamily protein
259	Glyma12g21140	22379200:25629700	3250500	YIELD	Q_COLYLDRF_12	PF00954,PF07714,PF08276,PF01453 PTHR24420,PTHR24420:SF703 KOG1187 GO:0004672,GO:0005524,GO:0006468,GO:0048544,GO:0005529 AT4G27290.1 S-locus lectin protein kinase family protein
260	Glyma12g21150	22379200:25629700	3250500	YIELD	Q_COLYLDRF_12	PTHR24093:SF73,PTHR24093 AT5G57110.1 ACA8,AT-ACA8 autoinhibited Ca2+ -ATPase, isoform 8
261	Glyma12g21640	22379200:25629700	3250500	YIELD	Q_COLYLDRF_12	PF00954,PF08276,PF01453,PF07714 PTHR24420,PTHR24420:SF432 KOG1187 GO:0004672,GO:0005524,GO:0006468,GO:0048544,GO:0005529 AT1G65790.1 ARK1,RK1 receptor kinase 1
262	Glyma12g21920	22379200:25629700	3250500	YIELD	Q_COLYLDRF_12	PTHR10891,PTHR10891:SF148 AT4G27280.1 Calcium-binding EF-hand family protein
263	Glyma12g22160	22379200:25629700	3250500	YIELD	Q_COLYLDRF_12	PF00582 GO:0006950 AT1G11360.1 Adenine nucleotide alpha hydrolases-like superfamily protein
264	Glyma12g22220	22379200:25629700	3250500	YIELD	Q_COLYLDRF_12	AT1G49430.1 LACS2,LRD2 long-chain acyl-CoA synthetase 2
265	Glyma12g22230	22379200:25629700	3250500	YIELD	Q_COLYLDRF_12	PF00067 PTHR24286,PTHR24286:SF25 GO:0005506,GO:0009055,GO:0016705,GO:0020037,GO:0055114 AT3G19270.1 CYP707A4 cytochrome P450, family 707, subfamily A, polypeptide 4
266	Glyma12g22240	22379200:25629700	3250500	YIELD	Q_COLYLDRF_12	PF02148,PF00443,PF00627 PTHR24006,PTHR24006:SF36 KOG0944 3.1.2.15 K11836 GO:0004221,GO:0006511,GO:0005515,GO:0008270 AT3G20630.1 ATUBP14,PER1,TTN6,UBP14 ubiquitin-specific protease 14
267	Glyma12g22280	22379200:25629700	3250500	YIELD	Q_COLYLDRF_12	PF00609,PF00781 PTHR11255,PTHR11255:SF1 GO:0004143,GO:0007205 AT2G20900.2 ATDGK5,DGK5 diacylglycerol kinase 5
268	Glyma12g22290	22379200:25629700	3250500	YIELD	Q_COLYLDRF_12	PF01535 PTHR24015 AT1G16480.1 Tetratricopeptide repeat (TPR)-like superfamily protein
269	Glyma12g22300	22379200:25629700	3250500	YIELD	Q_COLYLDRF_12	PF00612,PF00063 PTHR13140,PTHR13140:SF37 KOG0160 GO:0003774,GO:0005524,GO:0016459,GO:0005515 AT5G54280.2 ATM2,ATM4,ATMYOS1 myosin 2
270	Glyma12g22880	22379200:25629700	3250500	YIELD	Q_COLYLDRF_12	PF02365 GO:0003677,GO:0006355 AT4G27410.2 ANAC072,RD26 NAC (No Apical Meristem) domain transcriptional regulator superfamily protein
271	Glyma12g22960	22379200:25629700	3250500	YIELD	Q_COLYLDRF_12	PF03398 PTHR12161:SF9,PTHR12161 AT2G14830.1 Regulator of Vps4 activity in the MVB pathway protein
272	Glyma12g23000	22379200:25629700	3250500	YIELD	Q_COLYLDRF_12	PF03358 KOG3135 AT4G27270.1 Quinone reductase family protein
273	Glyma12g23010	22379200:25629700	3250500	YIELD	Q_COLYLDRF_12	AT5G49570.1 AtPNG1,PNG1 peptide-N-glycanase 1
274	Glyma12g23080	22379200:25629700	3250500	YIELD	Q_COLYLDRF_12	PF03018 PTHR21495:SF10,PTHR21495 AT4G13580.1 Disease resistance-responsive (dirigent-like protein) family protein
275	Glyma12g23110	22379200:25629700	3250500	YIELD	Q_COLYLDRF_12	PF00400 PTHR14221 KOG0283 GO:0005515 AT5G54200.1 Transducin/WD40 repeat-like superfamily protein
276	Glyma12g23150	22379200:25629700	3250500	YIELD	Q_COLYLDRF_12	PF12481,PF12504 PTHR11772 AT4G27450.1 Aluminium induced protein with YGL and LRDR motifs
277	Glyma12g23180	22379200:25629700	3250500	YIELD	Q_COLYLDRF_12	PF04515 PTHR12385,PTHR12385:SF5 KOG1362 AT3G15380.1 Plasma-membrane choline transporter family protein
278	Glyma12g23200	22379200:25629700	3250500	YIELD	Q_COLYLDRF_12	PF01357,PF03330 AT3G15370.1 ATEXP12,ATEXPA12,ATHEXP ALPHA 1.24,EXP12,EXPA12 expansin 12
279	Glyma12g23220	22379200:25629700	3250500	YIELD	Q_COLYLDRF_12	PF04389 PTHR10404 GO:0008233,GO:0006508 AT3G54720.1 AMP1,COP2,HPT,MFO1,PT Peptidase M28 family protein
280	Glyma12g23230	22379200:25629700	3250500	YIELD	Q_COLYLDRF_12	PF01805,PF04818 PTHR12323 GO:0003723,GO:0006396 AT4G31200.1 SWAP (Suppressor-of-White-APricot)/surp RNA-binding domain-containing protein
281	Glyma13g00780	0:2239890	2239890	WHOLE-PLANT	Node num 1-5	PF01263 PTHR11122 KOG1594 GO:0016853,GO:0005975 AT5G57330.1 Galactose mutarotase-like superfamily protein
282	Glyma13g00790	0:2239890	2239890	WHOLE-PLANT	Node num 1-5	PF00141 1.11.1.7 K00430 GO:0004601,GO:0020037,GO:0006979,GO:0055114 AT2G18980.1 Peroxidase superfamily protein
283	Glyma13g00800	0:2239890	2239890	WHOLE-PLANT	Node num 1-5	AT5G57340.1
284	Glyma13g00820	0:2239890	2239890	WHOLE-PLANT	Node num 1-5	PF07466 AT5G57345.1
285	Glyma13g00840	0:2239890	2239890	WHOLE-PLANT	Node num 1-5	PF00690,PF00122,PF00702 PTHR24093,PTHR24093:SF61 KOG0205 3.6.3.6 K01535 GO:0000166,GO:0046872,GO:0003824,GO:0008152,GO:0016020,GO:0015662,GO:0006812,GO:0006754 AT4G30190.1 AHA2,HA2,PMA2 H(+)-ATPase 2
286	Glyma13g00850	0:2239890	2239890	WHOLE-PLANT	Node num 1-5	PF01040 PTHR11048,PTHR11048:SF1 GO:0004659,GO:0016021 AT2G18950.1 ATHPT,HPT1,TPT1,VTE2 homogentisate phytyltransferase 1

287	Glyma13g00860	0:2239890	2239890	WHOLE-PLANT	Node num 1-5	PF00646,PF07646,PF00989,PF01344 PTHR23244,PTHR23244:SF9 KOG4693 K12115 GO:0005515,GO:0006355 AT5G57360.1 ADO1,FKL2,LKP1,ZTL Galactose oxidase/kelch repeat superfamily protein
288	Glyma13g00870	0:2239890	2239890	WHOLE-PLANT	Node num 1-5	AT2G18910.1 hydroxyproline-rich glycoprotein family protein
289	Glyma13g00880	0:2239890	2239890	WHOLE-PLANT	Node num 1-5	PF00400 PTHR22847:SF195,PTHR22847 KOG1963 GO:0005515 AT2G18900.1 Transducin/WD40 repeat-like superfamily protein
290	Glyma13g00890	0:2239890	2239890	WHOLE-PLANT	Node num 1-5	PF00069 PTHR24420:SF548,PTHR24420 KOG1187 GO:0004672,GO:0005524,GO:0006468 AT2G18890.1 Protein kinase superfamily protein
291	Glyma13g00900	0:2239890	2239890	WHOLE-PLANT	Node num 1-5	PF08648 KOG3263 K12846 AT5G57370.1
292	Glyma13g00910	0:2239890	2239890	WHOLE-PLANT	Node num 1-5	PF00101 GO:0016984,GO:0015977 AT5G38420.1 Ribulose bisphosphate carboxylase (small chain) family protein
293	Glyma13g00920	0:2239890	2239890	WHOLE-PLANT	Node num 1-5	PF00041,PF07227 PTHR21736:SF13,PTHR21736 GO:0005515 AT4G30200.3 VEL1 vernalization5/VIN3-like
294	Glyma13g00950	0:2239890	2239890	WHOLE-PLANT	Node num 1-5	PF00847 PTHR11795:SF332,PTHR11795 K09285 GO:0003700,GO:0006355 AT5G57390.1 AIL5,CHO1,EMK AINTEGUMENTA-like 5
295	Glyma13g00960	0:2239890	2239890	WHOLE-PLANT	Node num 1-5	PF01336,PF08784 PTHR13989,PTHR13989:SF7 KOG3108 K10739 GO:0003676 AT3G02920.1 ATRPA32B,RPA32B Replication protein A, subunit RPA32
296	Glyma13g00970	0:2239890	2239890	WHOLE-PLANT	Node num 1-5	AT4G25850.2 ORP4B OSBP(oxysterol binding protein)-related protein 4B
297	Glyma13g00980	0:2239890	2239890	WHOLE-PLANT	Node num 1-5	PF11559 PTHR21736:SF19,PTHR21736 AT5G57410.3 Afadin/alpha-actinin-binding protein
298	Glyma13g00990	0:2239890	2239890	WHOLE-PLANT	Node num 1-5	PF00667,PF00175,PF00258 PTHR19384:SF17,PTHR19384 KOG1158 1.6.2.4 K00327 GO:0016491,GO:0055114,GO:0010181 AT4G30210.2 AR2,ATR2 P450 reductase 2
299	Glyma13g01000	0:2239890	2239890	WHOLE-PLANT	Node num 1-5	PF00702 PTHR18901 KOG2914 GO:0003824,GO:0008152 AT5G57440.1 GPP2,GS1 Haloacid dehalogenase-like hydrolase (HAD) superfamily protein
300	Glyma13g01020	0:2239890	2239890	WHOLE-PLANT	Node num 1-5	PF00004 PTHR23070:SF1,PTHR23070 KOG0743 GO:0005524 AT5G57480.1 P-loop containing nucleoside triphosphate hydrolases superfamily protein
301	Glyma13g01030	0:2239890	2239890	WHOLE-PLANT	Node num 1-5	PF04893 PTHR21236,PTHR21236:SF1 KOG2946 GO:0016020 AT2G18840.1 Integral membrane Yip1 family protein
302	Glyma13g01040	0:2239890	2239890	WHOLE-PLANT	Node num 1-5	PF01459 PTHR11743,PTHR11743:SF14 KOG3126 GO:0008308,GO:0006820,GO:0044070,GO:0055085,GO:0005741 AT5G57490.1 ATVDAC4,VDAC4 voltage dependent anion channel 4
303	Glyma13g01050	0:2239890	2239890	WHOLE-PLANT	Node num 1-5	PF03807,PF02153 GO:0004665,GO:0008977,GO:0006571,GO:0055114,GO:0004616,GO:0006098,GO:0016491 AT5G34930.1 arogenate dehydrogenase
304	Glyma13g01060	0:2239890	2239890	WHOLE-PLANT	Node num 1-5	PF01762 PTHR11214,PTHR11214:SF3 KOG2287 GO:0008378,GO:0006486,GO:0016020 AT5G57500.1 Galactosyltransferase family protein
305	Glyma13g01070	0:2239890	2239890	WHOLE-PLANT	Node num 1-5	AT5G57510.1
306	Glyma13g01080	0:2239890	2239890	WHOLE-PLANT	Node num 1-5	PF00501 PTHR24096,PTHR24096:SF43 KOG1176 GO:0003824,GO:0008152 AT3G21240.1 4CL2,AT4CL2 4-coumarate:CoA ligase 2
307	Glyma13g01090	0:2239890	2239890	WHOLE-PLANT	Node num 1-5	PTHR10042:SF14,PTHR10042 AT5G57520.1 ATZFP2,ZFP2 zinc finger protein 2
308	Glyma13g01100	0:2239890	2239890	WHOLE-PLANT	Node num 1-5	PF04535 PTHR11615:SF33,PTHR11615 AT4G25830.1 Uncharacterised protein family (UPF0497)
309	Glyma13g01110	0:2239890	2239890	WHOLE-PLANT	Node num 1-5	PF06955,PF00722 PTHR10963:SF14,PTHR10963 2.4.1.207 K08235 GO:0004553,GO:0005975,GO:0016762,GO:0006073,GO:0005618,GO:0048046 AT4G25810.1 XTH23,XTR6 xyloglucan endotransglycosylase 6
310	Glyma13g01120	0:2239890	2239890	WHOLE-PLANT	Node num 1-5	PF06955,PF00722 PTHR10963,PTHR10963:SF14 2.4.1.207 K08235 GO:0004553,GO:0005975,GO:0016762,GO:0006073,GO:0005618,GO:0048046 AT4G25810.1 XTH23,XTR6 xyloglucan endotransglycosylase 6
311	Glyma13g01140	0:2239890	2239890	WHOLE-PLANT	Node num 1-5	PF00722,PF06955 PTHR10963:SF14,PTHR10963 2.4.1.207 K08235 GO:0004553,GO:0005975,GO:0016762,GO:0006073,GO:0005618,GO:0048046 AT5G57560.1 TCH4,XTH22 Xyloglucan endotransglucosylase/hydrolase family protein

312	Glyma13g01150	0:2239890	2239890	WHOLE-PLANT	Node num 1-5	PF00722,PF06955 PTHR10963,PTHR10963:SF14 2.4.1.207 K14504 GO:0004553,GO:0005975,GO:0016762,GO:0006073,GO:0005618,GO:0048046 AT5G57560.1 TCH4,XTH22 Xyloglucan endotransglucosylase/hydrolase family protein
313	Glyma13g01160	0:2239890	2239890	WHOLE-PLANT	Node num 1-5	PF07887 K02183 AT5G57580.1 Calmodulin-binding protein
314	Glyma13g01170	0:2239890	2239890	WHOLE-PLANT	Node num 1-5	PF00202 PTHR11986:SF4,PTHR11986 KOG1401 2.6.1.62 K00833 GO:0008483,GO:0030170 AT5G57590.1 BIO1 adenosylmethionine-8-amino-7-oxononanoate transaminases
315	Glyma13g01180	0:2239890	2239890	WHOLE-PLANT	Node num 1-5	PF00370,PF02782 PTHR10196:SF8,PTHR10196 KOG2517 GO:0016773,GO:0005975 AT4G30310.2 FGGY family of carbohydrate kinase
316	Glyma13g01190	0:2239890	2239890	WHOLE-PLANT	Node num 1-5	PF07714,PF00564 PTHR23257,PTHR23257:SF68 KOG0192 GO:0004672,GO:0005524,GO:0006468,GO:0005515 AT5G57610.1 Protein kinase superfamily protein with octicosapeptide/Phox/Bem1p domain
317	Glyma13g01200	0:2239890	2239890	WHOLE-PLANT	Node num 1-5	PF00249 PTHR10641 KOG0048 K09422 GO:0003677 AT5G57620.1 AtMYB36,MYB36 myb domain protein 36
318	Glyma13g01220	0:2239890	2239890	WHOLE-PLANT	Node num 1-5	PF00201 PTHR11926,PTHR11926:SF15 KOG1192 2.4.1.91 K10757 GO:0016758,GO:0008152 AT5G17050.1 UGT78D2 UDP-glucosyl transferase 78D2
319	Glyma13g01230	0:2239890	2239890	WHOLE-PLANT	Node num 1-5	PF00188 PTHR10334,PTHR10334:SF7 KOG3017 AT4G30320.1 CAP (Cysteine-rich secretory proteins, Antigen 5, and Pathogenesis-related 1 protein) superfamily
320	Glyma13g01250	0:2239890	2239890	WHOLE-PLANT	Node num 1-5	PF00188 PTHR10334:SF7,PTHR10334 AT4G25780.1 CAP (Cysteine-rich secretory proteins, Antigen 5, and Pathogenesis-related 1 protein) superfamily protein
321	Glyma13g01260	0:2239890	2239890	WHOLE-PLANT	Node num 1-5	AT5G57655.2 xylose isomerase family protein
322	Glyma13g01270	0:2239890	2239890	WHOLE-PLANT	Node num 1-5	PF00025 PTHR11711 KOG0070 K07942 GO:0005525,GO:0007264,GO:0004871,GO:0019001,GO:0007186,GO:0005634,GO:0005737,GO:0005622 AT2G24765.1 ARF3,ARL1,ATARL1 ADP-ribosylation factor 3
323	Glyma13g01280	0:2239890	2239890	WHOLE-PLANT	Node num 1-5	PF02779,PF02780 PTHR11624,PTHR11624:SF20 KOG0524 GO:0003824,GO:0008152 AT4G15560.1 CLA,CLA1,DEF,DXPS2,DXS Deoxyxylulose-5-phosphate synthase
324	Glyma13g01290	0:2239890	2239890	WHOLE-PLANT	Node num 1-5	PF00643,PF06203 KOG1601 GO:0008270,GO:0005622,GO:0005515 AT5G57660.1 ATCOL5,COL5 CONSTANS-like 5
325	Glyma13g01300	0:2239890	2239890	WHOLE-PLANT	Node num 1-5	PF00069,PF00582 PTHR24420:SF791,PTHR24420 KOG1187 GO:0004672,GO:0005524,GO:0006468,GO:0006950 AT5G57670.2 Protein kinase superfamily protein
326	Glyma13g01320	0:2239890	2239890	WHOLE-PLANT	Node num 1-5	PF01423 PTHR11193 KOG1774 K11097 AT2G18740.1 Small nuclear ribonucleoprotein family protein
327	Glyma13g01330	0:2239890	2239890	WHOLE-PLANT	Node num 1-5	PF00060,PF00497 PTHR18966,PTHR18966:SF6 GO:0004970,GO:0005234,GO:0016020,GO:0005215,GO:0006810,GO:0030288 AT5G11210.1 ATGLR2.5,GLR2.5 glutamate receptor 2.5
328	Glyma13g01360	0:2239890	2239890	WHOLE-PLANT	Node num 1-5	PF00609,PF00781 PTHR11255:SF32,PTHR11255 GO:0004143,GO:0007205 AT4G30340.1 ATDGK7,DGK7 diacylglycerol kinase 7
329	Glyma13g01370	0:2239890	2239890	WHOLE-PLANT	Node num 1-5	PF01980 PTHR12818 KOG2942 AT4G28020.1
330	Glyma13g01380	0:2239890	2239890	WHOLE-PLANT	Node num 1-5	PF02012 AT5G57700.1 BNR/Asp-box repeat family protein
331	Glyma13g01390	0:2239890	2239890	WHOLE-PLANT	Node num 1-5	AT5G57700.3 BNR/Asp-box repeat family protein
332	Glyma13g01400	0:2239890	2239890	WHOLE-PLANT	Node num 1-5	PTHR11638,PTHR11638:SF63 KOG1051 AT5G57710.1 Double Clp-N motif-containing P-loop nucleoside triphosphate hydrolases superfamily protein
333	Glyma13g17100	15523600:22497400	6973800	YIELD	Q_COLYLDRF_13	PF07911 AT5G20670.1 Protein of unknown function (DUF1677)
334	Glyma13g17130	15523600:22497400	6973800	YIELD	Q_COLYLDRF_13	PF00092 PTHR10338,PTHR10338:SF8 GO:0005515 AT1G19110.1 inter-alpha-trypsin inhibitor heavy chain-related
335	Glyma13g17140	15523600:22497400	6973800	YIELD	Q_COLYLDRF_13	PF06172 K09705 AT1G19130.1
336	Glyma13g17150	15523600:22497400	6973800	YIELD	Q_COLYLDRF_13	PF08511 PTHR21427,PTHR21427:SF2 KOG2969 AT1G19140.1
337	Glyma13g17160	15523600:22497400	6973800	YIELD	Q_COLYLDRF_13	PF00069,PF00560,PF08263 PTHR24420,PTHR24420:SF823 KOG1187 GO:0004672,GO:0005524,GO:0006468,GO:0005515 AT3G42880.1 Leucine-rich repeat protein kinase family protein
338	Glyma13g17170	15523600:22497400	6973800	YIELD	Q_COLYLDRF_13	PF00295 GO:0004650,GO:0005975 AT1G19170.1 Pectin lyase-like superfamily protein

339	Glyma13g17180	15523600:22497400	6973800	YIELD	Q_COLYLDRF_13	PF09425,PF06200 K13464 GO:0005515 AT1G19180.1 JAZ1,TIFY10A jasmonate-zim-domain protein 1
340	Glyma13g17190	15523600:22497400	6973800	YIELD	Q_COLYLDRF_13	
341	Glyma13g17200	15523600:22497400	6973800	YIELD	Q_COLYLDRF_13	PF00076 PTHR24012,PTHR24012:SF210 KOG0148 GO:0003676 AT1G17370.1 UBP1B oligouridylate binding protein 1B
342	Glyma13g17220	15523600:22497400	6973800	YIELD	Q_COLYLDRF_13	PF04570 AT1G74940.1 Protein of unknown function (DUF581)
343	Glyma13g17240	15523600:22497400	6973800	YIELD	Q_COLYLDRF_13	PF02140,PF01301,PF02837 PTHR23421,PTHR23421:SF35 KOG0496 GO:0004553,GO:0005975,GO:0005529,GO:0004565,GO:0009341 AT5G20710.1 BGAL7 beta-galactosidase 7
344	Glyma13g17250	15523600:22497400	6973800	YIELD	Q_COLYLDRF_13	PF00847 GO:0003700,GO:0006355 AT1G19210.1 Integrase-type DNA-binding superfamily protein
345	Glyma13g17260	15523600:22497400	6973800	YIELD	Q_COLYLDRF_13	PF00166 PTHR10772,PTHR10772:SF0 KOG1641 GO:0006457,GO:0005737 AT5G20720.1 ATCPN21,CHCPN10,CPN10,CPN20,CPN21 chaperonin 20
346	Glyma13g17270	15523600:22497400	6973800	YIELD	Q_COLYLDRF_13	PF06507,PF02362,PF02309 KOG4725 K14486 GO:0006355,GO:0005634,GO:0003677,GO:0009725 AT5G20730.2 ARF7,BIP,IAA21,IAA23,IAA25,MSG1,NPH4,TIR5 Transcriptional factor B3 family protein / auxin-responsive factor AUX/IAA-related
347	Glyma13g17290	15523600:22497400	6973800	YIELD	Q_COLYLDRF_13	PF00109,PF02801 PTHR11712:SF52,PTHR11712 KOG1394 2.3.1.179 K09458 GO:0009058,GO:0003824 AT1G74960.1 ATKAS2,FAB1,KAS2 fatty acid biosynthesis 1
348	Glyma13g17300	15523600:22497400	6973800	YIELD	Q_COLYLDRF_13	PF08161 PTHR21576,PTHR21576:SF2 KOG1248 AT2G34357.1 ARM repeat superfamily protein
349	Glyma13g17320	15523600:22497400	6973800	YIELD	Q_COLYLDRF_13	PF00005 PTHR24221 GO:0005524,GO:0016887 AT3G28390.1 PGP18 P-glycoprotein 18
350	Glyma13g17330	15523600:22497400	6973800	YIELD	Q_COLYLDRF_13	AT5G20790.1
351	Glyma13g17340	15523600:22497400	6973800	YIELD	Q_COLYLDRF_13	PF00743 PTHR23023,PTHR23023:SF18 KOG1399 GO:0004499,GO:0050660,GO:0050661,GO:0055114,GO:0016491 AT1G19250.1 FMO1 flavin-dependent monooxygenase 1
352	Glyma13g17350	15523600:22497400	6973800	YIELD	Q_COLYLDRF_13	PF00578 PTHR10681:SF5,PTHR10681 KOG0855 1.11.1.15 K03564 GO:0016209,GO:0016491,GO:0055114 AT3G26060.1 ATPRX Q Thioredoxin superfamily protein
353	Glyma13g17360	15523600:22497400	6973800	YIELD	Q_COLYLDRF_13	AT1G19240.1
354	Glyma13g17370	15523600:22497400	6973800	YIELD	Q_COLYLDRF_13	PF00111 PTHR23426:SF1,PTHR23426 KOG3309 GO:0009055,GO:0051536 AT4G21090.1 ATMFDX2,MFDX2 MITOCHONDRIAL FERREDOXIN 2
355	Glyma13g17380	15523600:22497400	6973800	YIELD	Q_COLYLDRF_13	PF02519 K14488 AT3G43120.1 SAUR-like auxin-responsive protein family
356	Glyma13g17390	15523600:22497400	6973800	YIELD	Q_COLYLDRF_13	PF01095 PTHR22931,PTHR22931:SF5 GO:0030599,GO:0042545,GO:0005618 AT5G61680.1 Pectin lyase-like superfamily protein
357	Glyma13g17400	15523600:22497400	6973800	YIELD	Q_COLYLDRF_13	PF02519 AT5G20820.1 SAUR-like auxin-responsive protein family
358	Glyma13g17421	15523600:22497400	6973800	YIELD	Q_COLYLDRF_13	PF00862,PF00534 PTHR12526,PTHR12526:SF27 KOG0853 2.4.1.13 K00695 GO:0009058,GO:0005985 AT3G43190.1 ATSUS4,SUS4 sucrose synthase 4
359	Glyma13g17440	15523600:22497400	6973800	YIELD	Q_COLYLDRF_13	PF00225,PF11995 PTHR24115,PTHR24115:SF323 KOG0242 GO:0003777,GO:0005524,GO:0007018 AT3G43210.1 ATNACK2,NACK2,TES ATP binding microtubule motor family protein
360	Glyma13g17450	15523600:22497400	6973800	YIELD	Q_COLYLDRF_13	PF03399 PTHR13022 KOG3252 GO:0006413,GO:0005634,GO:0003743 AT4G33250.1 ATTIF3K1,EIF3K,TIF3K1 eukaryotic translation initiation factor 3K
361	Glyma13g17490	15523600:22497400	6973800	YIELD	Q_COLYLDRF_13	PF02383 PTHR11200,PTHR11200:SF9 KOG1888 GO:0042578 AT3G43220.1 Phosphoinositide phosphatase family protein
362	Glyma13g17500	15523600:22497400	6973800	YIELD	Q_COLYLDRF_13	PF04366,PF01363 PTHR15629 KOG1843 GO:0046872 AT3G43230.1 RING/FYVE/PHD-type zinc finger family protein
363	Glyma13g17510	15523600:22497400	6973800	YIELD	Q_COLYLDRF_13	PF01504,PF00118,PF01363 PTHR11353:SF14,PTHR11353 KOG0230 2.7.1.150 K00921 GO:0005524,GO:0044267,GO:0046872,GO:0016307,GO:0046488 AT3G14270.1 FAB1B phosphatidylinositol-4-phosphate 5-kinase family protein
364	Glyma13g17530	15523600:22497400	6973800	YIELD	Q_COLYLDRF_13	PF08423 PTHR22942:SF12,PTHR22942 KOG1433 K04482 GO:0003697,GO:0005524,GO:0006281,GO:0009432 AT5G20850.1 ATRAD51,RAD51 RAS associated with diabetes protein 51
365	Glyma13g17540	15523600:22497400	6973800	YIELD	Q_COLYLDRF_13	PF04043 GO:0004857,GO:0030599 AT2G47340.1 Plant invertase/pectin methylesterase inhibitor superfamily protein
366	Glyma13g17550	15523600:22497400	6973800	YIELD	Q_COLYLDRF_13	PF04043,PF01095 PTHR22931:SF5,PTHR22931 GO:0030599,GO:0042545,GO:0005618,GO:0004857 AT2G26450.1 Plant invertase/pectin methylesterase inhibitor superfamily

367	Glyma13g17570	15523600:22497400	6973800	YIELD	Q_COLYLDRF_13	PF01095,PF04043 PTHR22931:SF5,PTHR22931 3.1.1.11 K01051 GO:0030599,GO:0042545,GO:0005618,GO:0004857 AT3G43270.1 Plant invertase/pectin methylesterase inhibitor superfamily
368	Glyma13g17580	15523600:22497400	6973800	YIELD	Q_COLYLDRF_13	PF04455,PF05222,PF03435,PF01262 PTHR11133:SF3,PTHR11133 KOG0172 GO:0016491,GO:0055114 AT4G33150.1 LKR,LKR/SDH,SDH lysine-ketoglutarate reductase/saccharopine dehydrogenase bifunctional enzyme
369	Glyma13g17600	15523600:22497400	6973800	YIELD	Q_COLYLDRF_13	PF07983,PF00332 PTHR16631 GO:0004553,GO:0005975 AT5G20870.1 O-Glycosyl hydrolases family 17 protein
370	Glyma13g17610	15523600:22497400	6973800	YIELD	Q_COLYLDRF_13	PF09324,PF01369 PTHR10663,PTHR10663:SF56 KOG0929 K13462 GO:0005086,GO:0032012,GO:0005622 AT3G43300.1 ATMIN7,BEN1 HOPM interactor 7
371	Glyma13g17620	15523600:22497400	6973800	YIELD	Q_COLYLDRF_13	PF00097 PTHR22764,PTHR22764:SF31 AT3G43430.1 RING/U-box superfamily protein
372	Glyma13g17630	15523600:22497400	6973800	YIELD	Q_COLYLDRF_13	PF01363 PTHR13856:SF64,PTHR13856 GO:0046872 AT2G26210.1 Ankyrin repeat family protein
373	Glyma13g17640	15523600:22497400	6973800	YIELD	Q_COLYLDRF_13	PF09425,PF06200 GO:0005515 AT5G20900.1 JAZ12,TIFY3B jasmonate-zim-domain protein 12
374	Glyma13g17650	15523600:22497400	6973800	YIELD	Q_COLYLDRF_13	PTHR13343 KOG3374 AT2G04690.1 Pyridoxamine 5\'-phosphate oxidase family protein
375	Glyma13g17660	15523600:22497400	6973800	YIELD	Q_COLYLDRF_13	PF03647 PTHR12668 KOG4267 GO:0016020 AT3G43520.1 Transmembrane proteins 14C
376	Glyma13g17670	15523600:22497400	6973800	YIELD	Q_COLYLDRF_13	PF00999 PTHR16254 KOG1650 GO:0015299,GO:0006812,GO:0055085,GO:0016021 AT3G17630.1 ATCHX19,CHX19 cation/H+ exchanger 19
377	Glyma13g37040	15523600:22497400	6973800	YIELD	Q_COLYLDRF_14	AT2G20585.3 NFD6 nuclear fusion defective 6
378	Glyma14g14220	10874400:14120960	3246560	LEAF-STEM	Lf lgth 10-4	PF00234 AT2G45180.1 Bifunctional inhibitor/lipid-transfer protein/seed storage 2S albumin superfamily protein
379	Glyma14g14230	10874400:14120960	3246560	LEAF-STEM	Lf lgth 10-4	
380	Glyma14g14260	10874400:14120960	3246560	LEAF-STEM	Lf lgth 10-4	PF10536 PTHR11668:SF94,PTHR11668 AT2G25010.1 Aminotransferase-like, plant mobile domain family protein
381	Glyma14g14270	10874400:14120960	3246560	LEAF-STEM	Lf lgth 10-4	PF00156 PTHR11776 KOG1712 2.4.2.7 K00759 GO:0009116 AT5G11160.1 APT5 adenine phosphoribosyltransferase 5
382	Glyma14g14290	10874400:14120960	3246560	LEAF-STEM	Lf lgth 10-4	AT4G32190.1 Myosin heavy chain-related protein
383	Glyma14g14320	12692710:14485410	1792700	LEAF-STEM	Stm str 1-3	PF07714 PTHR22967,PTHR22967:SF2 KOG0198 GO:0004672,GO:0005524,GO:0006468 AT4G32250.1 Protein kinase superfamily protein
384	Glyma14g14350	12692710:14485410	1792700	LEAF-STEM	Stm str 1-3	PTHR14942 AT4G32270.1 Ubiquitin-like superfamily protein
385	Glyma14g14360	12692710:14485410	1792700	LEAF-STEM	Stm str 1-3	PF03151 PTHR11132 KOG1444 GO:0055085 AT4G32272.1 Nucleotide/sugar transporter family protein
386	Glyma14g14370	12692710:14485410	1792700	LEAF-STEM	Stm str 1-3	PF02485 GO:0008375,GO:0016020 AT5G25330.1 Core-2/I-branching beta-1,6-N-acetylglucosaminyltransferase family protein
387	Glyma14g14380	12692710:14485410	1792700	LEAF-STEM	Stm str 1-3	AT3G11760.1
388	Glyma14g14390	12692710:14485410	1792700	LEAF-STEM	Stm str 1-3	PF00069,PF01453 PTHR24420,PTHR24420:SF926 KOG1187 GO:0004672,GO:0005524,GO:0006468,GO:0005529 AT4G32300.1 SD2-5 S-domain-2 5
389	Glyma14g14400	12692710:14485410	1792700	LEAF-STEM	Stm str 1-3	PF06886 AT4G32330.1 TPX2 (targeting protein for Xklp2) protein family
390	Glyma14g14410	12692710:14485410	1792700	LEAF-STEM	Stm str 1-3	PF00560,PF00646 PTHR23125,PTHR23125:SF106 KOG1947 K14515 GO:0005515 AT2G25490.1 EBF1,FBL6 EIN3-binding F box protein 1
391	Glyma14g14750	14223400:21906400	7683000	LEAF-STEM	Lf lgth 10-3	PF03638 PTHR12446:SF4,PTHR12446 AT3G22780.1 ATTSO1,TSO1 Tesmin/TSO1-like CXC domain-containing protein
392	Glyma14g14970	14223400:21906400	7683000	LEAF-STEM	Lf lgth 10-3	PF00160 PTHR11071 KOG0879 GO:0003755,GO:0006457 AT3G63400.1 Cyclophilin-like peptidyl-prolyl cis-trans isomerase family protein
393	Glyma14g14990	14223400:21906400	7683000	LEAF-STEM	Lf lgth 10-3	PF02770 PTHR10909,PTHR10909:SF11 GO:0003995,GO:0055114 AT2G35690.1 ACX5 acyl-CoA oxidase 5
394	Glyma14g15040	14223400:21906400	7683000	LEAF-STEM	Lf lgth 10-3	PF08698 PTHR21686:SF3,PTHR21686 KOG3100 AT5G30495.1 Fcf2 pre-rRNA processing protein
395	Glyma14g15070	14223400:21906400	7683000	LEAF-STEM	Lf lgth 10-3	PF01490 PTHR22950,PTHR22950:SF9 KOG1303 AT2G41190.1 Transmembrane amino acid transporter family protein
396	Glyma14g15080	14223400:21906400	7683000	LEAF-STEM	Lf lgth 10-3	PF08698 PTHR21686,PTHR21686:SF3 AT1G54770.1 Fcf2 pre-rRNA processing protein
397	Glyma14g15120	14223400:21906400	7683000	LEAF-STEM	Lf lgth 10-3	PF04862 AT5G11420.1 Protein of unknown function, DUF642
398	Glyma14g17190	14223400:21906400	7683000	LEAF-STEM	Lf lgth 10-3	AT4G31360.1 selenium binding

399	Glyma14g17200	14223400:21906400	7683000	LEAF-STEM	Lf lgth 10-3	PF02469 AT2G24450.1 FLA3 FASCICLIN-like arabinogalactan protein 3 precursor
400	Glyma15g06360	3124420:6737400	3612980	YIELD	Flr num 1-7	PF00013 PTHR10288,PTHR10288:SF53 KOG2190 GO:0003723 AT5G64390.1 HEN4 RNA-binding KH domain-containing protein
401	Glyma15g06380	3124420:6737400	3612980	YIELD	Flr num 1-7	PF01031,PF00350,PF02212 PTHR11566:SF12,PTHR11566 KOG0446 3.6.5.5 K01528 GO:0003924,GO:0005525 AT4G33650.1 ADL2,DRP3A dynamin-related protein 3A
402	Glyma15g06390	3124420:6737400	3612980	YIELD	Flr num 1-7	PF00201 PTHR11926:SF71,PTHR11926 KOG1192 GO:0016758,GO:0008152 AT5G17030.1 UGT78D3 UDP-glucosyl transferase 78D3
403	Glyma15g06400	3124420:6737400	3612980	YIELD	Flr num 1-7	PF00171 PTHR11699,PTHR11699:SF27 KOG2450 1.2.1.27 K00140 GO:0016491,GO:0008152,GO:0055114,GO:0004491 AT2G14170.1 ALDH6B2 aldehyde dehydrogenase 6B2
404	Glyma15g06410	3124420:6737400	3612980	YIELD	Flr num 1-7	PF01535 PTHR24015,PTHR24015:SF182 AT4G31070.1 Tetratricopeptide repeat (TPR)-like superfamily protein
405	Glyma15g06420	3124420:6737400	3612980	YIELD	Flr num 1-7	PF04576 AT1G18265.1 Protein of unknown function, DUF593
406	Glyma15g06430	3124420:6737400	3612980	YIELD	Flr num 1-7	PF00139,PF00069 PTHR24420,PTHR24420:SF438 KOG1187 GO:0004672,GO:0005524,GO:0006468,GO:0005488 AT5G10530.1 Concanavalin A-like lectin protein kinase family protein
407	Glyma15g06450	3124420:6737400	3612980	YIELD	Flr num 1-7	PF00400 PTHR18359 KOG2055 K14553 GO:0005515 AT5G14050.1 Transducin/WD40 repeat-like superfamily protein
408	Glyma15g06460	3124420:6737400	3612980	YIELD	Flr num 1-7	PF08240,PF00107 PTHR11695:SF285,PTHR11695 KOG0023 GO:0008270,GO:0016491,GO:0055114 AT1G72680.1 ATCAD1,CAD1 cinnamyl-alcohol dehydrogenase
409	Glyma15g06470	3124420:6737400	3612980	YIELD	Flr num 1-7	PF00319 PTHR11945:SF19,PTHR11945 GO:0003677,GO:0046983,GO:0043565,GO:0006355,GO:0005634,GO:0003700 AT2G14210.1 AGL44,ANR1 AGAMOUS-like 44
410	Glyma15g06491	3124420:6737400	3612980	YIELD	Flr num 1-7	PF01486 PTHR11945:SF96,PTHR11945 GO:0003700,GO:0006355,GO:0005634 AT4G37940.1 AGL21 AGAMOUS-like 21
411	Glyma15g06510	3124420:6737400	3612980	YIELD	Flr num 1-7	PF03169 PTHR22601 KOG2262 GO:0055085 AT5G64410.1 ATOPT4,OPT4 oligopeptide transporter 4
412	Glyma15g06520	3124420:6737400	3612980	YIELD	Flr num 1-7	PF03169 PTHR22601 KOG2262 GO:0055085 AT5G64410.1 ATOPT4,OPT4 oligopeptide transporter 4
413	Glyma15g06530	3124420:6737400	3612980	YIELD	Flr num 1-7	PF00012 PTHR19375:SF1,PTHR19375 KOG0102 K03283 GO:0000902,GO:0005524 AT5G09590.1 HSC70-5,MTHSC70-2 mitochondrial HSO70 2
414	Glyma15g06540	3124420:6737400	3612980	YIELD	Flr num 1-7	PF01544 PTHR21535:SF5,PTHR21535 GO:0046873,GO:0030001,GO:0055085,GO:0016020 AT2G04305.1 Magnesium transporter CorA-like family protein
415	Glyma15g06550	3124420:6737400	3612980	YIELD	Flr num 1-7	PF04931 PTHR13213,PTHR13213:SF1 KOG1926 GO:0003677,GO:0003887,GO:0006351 AT5G64420.1 DNA polymerase V family
416	Glyma15g06560	3124420:6737400	3612980	YIELD	Flr num 1-7	PF00439 PTHR22880:SF31,PTHR22880 KOG1474 GO:0005515 AT5G65630.1 GTE7 global transcription factor group E7
417	Glyma15g06570	3124420:6737400	3612980	YIELD	Flr num 1-7	PF00439 PTHR22880:SF31,PTHR22880 KOG1474 GO:0005515 AT1G06230.1 GTE4 global transcription factor group E4
418	Glyma15g06590	3124420:6737400	3612980	YIELD	Flr num 1-7	PF00564 GO:0005515 AT5G64430.1 Octicosapeptide/Phox/Bem1p family protein
419	Glyma15g06600	3124420:6737400	3612980	YIELD	Flr num 1-7	PF01425 PTHR11895,PTHR11895:SF7 KOG1211 GO:0016884 AT5G64440.1 AtFAAH,FAAH fatty acid amide hydrolase
420	Glyma15g06611	3124420:6737400	3612980	YIELD	Flr num 1-7	AT5G64460.1 Phosphoglycerate mutase family protein
421	Glyma15g06620	3124420:6737400	3612980	YIELD	Flr num 1-7	AT5G64480.1
422	Glyma15g06630	3124420:6737400	3612980	YIELD	Flr num 1-7	PTHR12170,PTHR12170:SF3 KOG2817 AT4G37880.1 LisH/CRA/RING-U-box domains-containing protein
423	Glyma15g06650	3124420:6737400	3612980	YIELD	Flr num 1-7	PF01641 PTHR10173:SF0,PTHR10173 KOG0856 GO:0008113,GO:0055114 AT4G21860.1 MSRB2 methionine sulfoxide reductase B 2
424	Glyma15g06670	3124420:6737400	3612980	YIELD	Flr num 1-7	PF00719 PTHR10286:SF0,PTHR10286 KOG1626 3.6.1.1 K01507 GO:0000287,GO:0004427,GO:0006796,GO:0005737 AT5G09650.1 AtPPa6,PPa6 pyrophosphorylase 6
425	Glyma15g06680	3124420:6737400	3612980	YIELD	Flr num 1-7	PF00010 PTHR12565 GO:0030528,GO:0006355 AT4G37850.1 basic helix-loop-helix (bHLH) DNA-binding superfamily protein
426	Glyma15g06710	3124420:6737400	3612980	YIELD	Flr num 1-7	PF00561 PTHR10992:SF12,PTHR10992 KOG1454 3.4.11.5 K01259 AT2G14260.1 PIP proline iminopeptidase
427	Glyma15g06721	3124420:6737400	3612980	YIELD	Flr num 1-7	AT4G17000.1

428	Glyma15g06730	3124420:6737400	3612980	YIELD	Flr num 1-7	PF01266,PF08491 PTHR10835 KOG1298 1.14.99.7 K00511 GO:0009055,GO:0016491,GO:0055114,GO:0050660,GO:0008033,GO:0004506,GO:0016021 AT1G58440.1 SQE1,XF1 FAD/NAD(P)-binding oxidoreductase family protein
429	Glyma15g06740	3124420:6737400	3612980	YIELD	Flr num 1-7	AT4G33690.1
430	Glyma15g06750	3124420:6737400	3612980	YIELD	Flr num 1-7	PF01595 PTHR12064 KOG2118 AT4G33700.1 CBS domain-containing protein with a domain of unknown function (DUF21)
431	Glyma15g06760	3124420:6737400	3612980	YIELD	Flr num 1-7	PF07021 AT3G49720.1
432	Glyma15g06770	3124420:6737400	3612980	YIELD	Flr num 1-7	PF00188 PTHR10334 KOG3017 AT2G19990.1 PR-1-LIKE pathogenesis-related protein-1-like
433	Glyma15g06780	3124420:6737400	3612980	YIELD	Flr num 1-7	PF00188 PTHR10334 KOG3017 AT2G14580.1 ATPRB1,PRB1 basic pathogenesis-related protein 1
434	Glyma15g06790	3124420:6737400	3612980	YIELD	Flr num 1-7	PF00188 PTHR10334 KOG3017 AT2G14580.1 ATPRB1,PRB1 basic pathogenesis-related protein 1
435	Glyma15g06830	3124420:6737400	3612980	YIELD	Flr num 1-7	PF00188 PTHR10334 AT3G19690.1 CAP (Cysteine-rich secretory proteins, Antigen 5, and Pathogenesis-related 1 protein) superfamily protein
436	Glyma15g06840	3124420:6737400	3612980	YIELD	Flr num 1-7	PF00188 PTHR10334 AT3G19690.1 CAP (Cysteine-rich secretory proteins, Antigen 5, and Pathogenesis-related 1 protein) superfamily protein
437	Glyma15g06860	3124420:6737400	3612980	YIELD	Flr num 1-7	PF00010 PTHR23042,PTHR23042:SF20 GO:0030528,GO:0006355 AT5G54680.1 bHLH105,ILR3 basic helix-loop-helix (bHLH) DNA-binding superfamily protein
438	Glyma15g06870	3124420:6737400	3612980	YIELD	Flr num 1-7	PF00085 PTHR10438:SF12,PTHR10438 KOG0907 GO:0045454,GO:0015035,GO:0009055,GO:0006662 AT1G11530.1 ATCXXS1,CXXS1 C-terminal cysteine residue is changed to a serine 1
439	Glyma15g06880	3124420:6737400	3612980	YIELD	Flr num 1-7	PF00225 PTHR24115,PTHR24115:SF170 KOG0239 K10405 GO:0003777,GO:0005524,GO:0007018 AT4G21270.1 ATK1,KATA,KATAP kinesin 1
440	Glyma15g06890	3124420:6737400	3612980	YIELD	Flr num 1-7	PF04504 AT1G11510.1 DNA-binding storekeeper protein-related transcriptional regulator
441	Glyma15g06900	3124420:6737400	3612980	YIELD	Flr num 1-7	PF03737 K02553 AT5G56260.1 Ribonuclease E inhibitor RraA/Dimethylmenaquinone methyltransferase
442	Glyma15g06910	3124420:6737400	3612980	YIELD	Flr num 1-7	PF06749 AT4G21310.1 Protein of unknown function (DUF1218)
443	Glyma15g06920	3124420:6737400	3612980	YIELD	Flr num 1-7	AT4G27240.1 zinc finger (C2H2 type) family protein
444	Glyma15g06930	3124420:6737400	3612980	YIELD	Flr num 1-7	AT1G79660.1
445	Glyma15g06941	3124420:6737400	3612980	YIELD	Flr num 1-7	PF03000 GO:0004871,GO:0009416 AT3G22104.1 Phototropic-responsive NPH3 family protein
446	Glyma15g06950	3124420:6737400	3612980	YIELD	Flr num 1-7	PF00010 PTHR11514,PTHR11514:SF7 GO:0030528,GO:0006355 AT4G21330.1 DYT1 basic helix-loop-helix (bHLH) DNA-binding superfamily protein
447	Glyma15g11260	6107000:22655000	16548000	LEAF-STEM	Lflt shape 8-8	PTHR22764:SF49,PTHR22764 AT5G22000.1 RHF2A RING-H2 group F2A
448	Glyma15g11270	6107000:22655000	16548000	LEAF-STEM	Lflt shape 8-8	PF00892,PF03151 PTHR11132 KOG1441 GO:0016020,GO:0055085 AT1G61800.1 ATGPT2,GPT2 glucose-6-phosphate/phosphate translocator 2
449	Glyma15g11290	6107000:22655000	16548000	LEAF-STEM	Lflt shape 8-8	PF00232 PTHR10353 KOG0626 3.2.1.21 K05350 GO:0004553,GO:0005975 AT4G21760.1 BGLU47 beta-glucosidase 47
450	Glyma15g11300	6107000:22655000	16548000	LEAF-STEM	Lflt shape 8-8	PF00849 PTHR10436:SF42,PTHR10436 KOG1919 GO:0003723,GO:0009982,GO:0001522,GO:0009451 AT4G21770.1 Pseudouridine synthase family protein
451	Glyma15g11310	6107000:22655000	16548000	LEAF-STEM	Lflt shape 8-8	PF00962 PTHR11409,PTHR11409:SF21 KOG1097 3.5.4.4 K01488 GO:0019239,GO:0009168 AT4G04880.1 adenosine/AMP deaminase family protein
452	Glyma15g11320	6107000:22655000	16548000	LEAF-STEM	Lflt shape 8-8	
453	Glyma15g11330	6107000:22655000	16548000	LEAF-STEM	Lflt shape 8-8	PF00069 PTHR24420:SF700,PTHR24420 KOG1187 GO:0004672,GO:0005524,GO:0006468 AT3G20530.1 Protein kinase superfamily protein
454	Glyma15g11340	6107000:22655000	16548000	LEAF-STEM	Lflt shape 8-8	PF01535 PTHR24015:SF20,PTHR24015 AT1G61870.1 PPR336 pentatricopeptide repeat 336
455	Glyma15g11350	6107000:22655000	16548000	LEAF-STEM	Lflt shape 8-8	PF04511 PTHR11009 KOG0858 K13989 AT4G04860.1 DER2.2 DERLIN-2.2
456	Glyma15g11360	6107000:22655000	16548000	LEAF-STEM	Lflt shape 8-8	PF00011 PTHR11527,PTHR11527:SF14 AT4G21870.1 HSP20-like chaperones superfamily protein
457	Glyma15g11370	6107000:22655000	16548000	LEAF-STEM	Lflt shape 8-8	PF01753 GO:0008270 AT4G21890.1
458	Glyma15g11380	6107000:22655000	16548000	LEAF-STEM	Lflt shape 8-8	PF00076 PTHR24012:SF67,PTHR24012 KOG0148 GO:0003676 AT1G11650.2 ATRBP45B,RBP45B RNA-binding (RRM/RBD/RNP motifs) family protein

459	Glyma15g14890	6107000:22655000	16548000	LEAF-STEM	Lflt shape 8-8	AT3G17100.1 sequence-specific DNA binding transcription factors
460	Glyma15g14900	6107000:22655000	16548000	LEAF-STEM	Lflt shape 8-8	PF00481 PTHR13832:SF97,PTHR13832 KOG0700 GO:0003824 AT3G17090.1 Protein phosphatase 2C family protein
461	Glyma15g14930	6107000:22655000	16548000	LEAF-STEM	Lflt shape 8-8	PF00657 PTHR22835,PTHR22835:SF39 GO:0016788,GO:0006629 AT2G23540.1 GDSL-like Lipase/Acylhydrolase superfamily protein
462	Glyma15g14950	6107000:22655000	16548000	LEAF-STEM	Lflt shape 8-8	PF00657 PTHR22835:SF39,PTHR22835 GO:0016788,GO:0006629 AT2G23540.1 GDSL-like Lipase/Acylhydrolase superfamily protein
463	Glyma15g14970	6107000:22655000	16548000	LEAF-STEM	Lflt shape 8-8	KOG3450 AT3G06610.1 DNA-binding enhancer protein-related
464	Glyma15g14980	6107000:22655000	16548000	LEAF-STEM	Lflt shape 8-8	PF01590,PF00989,PF00360,PF00512,PF08446,PF02518 PTHR24423 K12121 GO:0008020,GO:0006355,GO:0009584,GO:0018298,GO:0000155,GO:0007165,GO:0016020,GO:0005515,GO:0005524 AT2G18790.1 HY3,OOP1,PHYB phytochrome B
465	Glyma15g15010	6107000:22655000	16548000	LEAF-STEM	Lflt shape 8-8	PF00285,PF00549 PTHR26330 KOG1254 GO:0046912,GO:0044262,GO:0003824,GO:0008152 AT5G49460.1 ACLB-2 ATP citrate lyase subunit B 2
466	Glyma15g15020	6107000:22655000	16548000	LEAF-STEM	Lflt shape 8-8	PF00285,PF00549 PTHR26330 KOG1254 2.3.3.8 K01648 GO:0046912,GO:0044262,GO:0003824,GO:0008152 AT3G06650.1 ACLB-1 ATP-citrate lyase B-1
467	Glyma16g09930	8471000:12411000	3940000	REPROD-PERIOD	Fflr 9-3	PF04564 PTHR26224,PTHR26224:SF27 KOG4642 GO:0004842,GO:0016567,GO:0000151 AT3G07370.1 ATCHIP,CHIP carboxyl terminus of HSC70-interacting protein
468	Glyma16g10340	8471000:12411000	3940000	REPROD-PERIOD	Fflr 9-3	PF01582,PF00931 PTHR11017,PTHR11017:SF130 GO:0043531,GO:0005515,GO:0007165,GO:0005622,GO:0045087,GO:0031224,GO:0004888,GO:0006915,GO:0005524 AT5G36930.2 Disease resistance protein (TIR-NBS-LRR class) family
469	Glyma19g02430	0:21124310	21124310	YIELD	Pod num 1-9	PF07847 PTHR22966 KOG4281 GO:0047800,GO:0055114 AT5G39890.1 Protein of unknown function (DUF1637)
470	Glyma19g02440	0:21124310	21124310	YIELD	Pod num 1-9	PF03106 GO:0003700,GO:0043565,GO:0006355 AT5G15130.1 ATWRKY72,WRKY72 WRKY DNA-binding protein 72
471	Glyma19g02450	0:21124310	21124310	YIELD	Pod num 1-9	PF01263 PTHR10091 KOG1604 GO:0016853,GO:0005975,GO:0019318 AT3G47800.1 Galactose mutarotase-like superfamily protein
472	Glyma19g02790	0:21124310	21124310	YIELD	Pod num 1-9	PF08574 AT2G30280.1 DMS4,RDM4 RNA-directed DNA methylation 4
473	Glyma19g02810	0:21124310	21124310	YIELD	Pod num 1-9	PF03330,PF01357 AT2G03090.1 ATEXP15,ATEXPA15,ATHEXP ALPHA 1.3,EXP15,EXPA15 expansin A15
474	Glyma19g02840	0:21124310	21124310	YIELD	Pod num 1-9	PF00013 PTHR10288:SF53,PTHR10288 KOG2190 GO:0003723 AT5G15270.1 RNA-binding KH domain-containing protein
475	Glyma19g04340	0:21124310	21124310	YIELD	Pod num 1-9	PF00097 PTHR22766,PTHR22766:SF20 KOG3039 AT5G15790.1 RING/U-box superfamily protein
476	Glyma19g04390	0:21124310	21124310	YIELD	Pod num 1-9	PF00005 PTHR19241:SF95,PTHR19241 GO:0005524,GO:0016887 AT1G15520.1 ABCG40,ATABCG40,ATPDR12,PDR12 pleiotropic drug resistance 12
477	Glyma19g04410	0:21124310	21124310	YIELD	Pod num 1-9	PF01190 AT5G15780.1 Pollen Ole e 1 allergen and extensin family protein
478	Glyma19g04430	0:21124310	21124310	YIELD	Pod num 1-9	PF01190 AT5G15780.1 Pollen Ole e 1 allergen and extensin family protein
479	Glyma19g04450	0:21124310	21124310	YIELD	Pod num 1-9	PF00230 PTHR19139,PTHR19139:SF30 KOG0223 GO:0005215,GO:0006810,GO:0016020 AT3G16240.1 AQP1,ATTIP2;1,DELTA-TIP,DELTA-TIP1,TIP2;1 delta tonoplast integral protein
480	Glyma19g04470	0:21124310	21124310	YIELD	Pod num 1-9	PF01190 AT5G15780.1 Pollen Ole e 1 allergen and extensin family protein
481	Glyma19g04540	0:21124310	21124310	YIELD	Pod num 1-9	PF01190 AT5G15780.1 Pollen Ole e 1 allergen and extensin family protein
482	Glyma19g04570	0:21124310	21124310	YIELD	Pod num 1-9	PF00201 PTHR11926:SF15,PTHR11926 KOG1192 GO:0016758,GO:0008152 AT1G22340.1 AtUGT85A7,UGT85A7 UDP-glucosyl transferase 85A7
483	Glyma19g04610	0:21124310	21124310	YIELD	Pod num 1-9	PF00201 PTHR11926:SF15,PTHR11926 KOG1192 GO:0016758,GO:0008152 AT1G22340.1 AtUGT85A7,UGT85A7 UDP-glucosyl transferase 85A7
484	Glyma19g04810	0:21124310	21124310	YIELD	Pod num 1-9	PF01612,PF00476 PTHR10133 GO:0003677,GO:0003887,GO:0006260,GO:0003676,GO:0008408,GO:0006139,GO:0005622 AT1G50840.1 POLGAMMA2 polymerase gamma 2
485	Glyma19g04820	0:21124310	21124310	YIELD	Pod num 1-9	PF10250 AT5G15740.1 O-fucosyltransferase family protein
486	Glyma19g04853	0:21124310	21124310	YIELD	Pod num 1-9	PF00153 PTHR24089:SF45,PTHR24089 AT5G42130.1 Mitochondrial substrate carrier family protein
487	Glyma19g04870	0:21124310	21124310	YIELD	Pod num 1-9	PF00069 PTHR24420,PTHR24420:SF741 KOG1187 GO:0004672,GO:0005524,GO:0006468 AT5G15730.1 Protein kinase superfamily protein

488	Glyma19g04890	0:21124310	21124310	YIELD	Pod num 1-9	PF00657 PTHR22835:SF39,PTHR22835 GO:0016788,GO:0006629 AT5G15720.1 GLIP7 GDSL-motif lipase 7
489	Glyma19g04900	0:21124310	21124310	YIELD	Pod num 1-9	PF00326 PTHR12277,PTHR12277:SF8 KOG1552 GO:0008236,GO:0006508 AT3G30380.2 alpha/beta-Hydrolases superfamily protein
490	Glyma19g05700	6081390:7517520	1436130	WHOLE-PLANT	Pl ht 22-1	PF03005 PTHR13533:SF3,PTHR13533 AT5G15900.1 TBL19 TRICHOME BIREFRINGENCE-LIKE 19
491	Glyma19g05740	6081390:7517520	1436130	WHOLE-PLANT	Pl ht 22-1	PF03005 PTHR13533,PTHR13533:SF3 AT5G15900.1 TBL19 TRICHOME BIREFRINGENCE-LIKE 19
492	Glyma19g05760	6081390:7517520	1436130	WHOLE-PLANT	Pl ht 22-1	PF03005 PTHR13533:SF3,PTHR13533 AT5G15900.1 TBL19 TRICHOME BIREFRINGENCE-LIKE 19
493	Glyma20g10920	15332510:16035800	703290	YIELD	Flr num 1-12	PF07714 PTHR24420:SF897,PTHR24420 KOG1187 GO:0004672,GO:0005524,GO:0006468 AT1G14370.1 APK2A,PBL2 protein kinase 2A
494	Glyma20g10960	15332510:16035800	703290	YIELD	Flr num 1-12	PF00069 PTHR24056,PTHR24056:SF77 KOG0600 2.7.11.22 K08819 GO:0004672,GO:0005524,GO:0006468 AT5G10270.1 CDKC;1 cyclin-dependent kinase C;1
495	Glyma20g11040	15332510:16035800	703290	YIELD	Flr num 1-12	PF00249 PTHR10641,PTHR10641:SF30 KOG0048 GO:0003677 AT5G06100.2 ATMYB33,MYB33 myb domain protein 33
496	Glyma20g11150	15332510:16035800	703290	YIELD	Flr num 1-12	PF05221 GO:0004013,GO:0006730 AT4G13940.4 ATSAHH1,EMB1395,HOG1,MEE58,SAHH1 S-adenosyl-L-homocysteine hydrolase

Supplementary Table 10. Gene list (270 genes) of the artificial selection region harboring QTL associated with major traits.

No	Gene	Start:End	Size	Trait	QTLs	Annotation
1	Glyma03g26060	**3699336:4200251**	500915	YIELD	Q_COLYLDIR_5-2	PF02298 GO:0005507,GO:0009055 AT2G32300.1 UCC1 uclacyanin 1
2	Glyma03g26060	**3699336:4200251**	500915	YIELD	Q_COLYLDIR_5-2	PF02298 GO:0005507,GO:0009055 AT1G72230.1 Cupredoxin superfamily protein
3	Glyma03g26080	**3699336:4200251**	500915	YIELD	Q_COLYLDIR_5-2	PF00573 PTHR10746 KOG1624 GO:0003735,GO:0006412,GO:0005840 AT1G07320.1 RPL4 ribosomal protein L4
4	Glyma03g26540	**3699336:4200251**	500915	YIELD	Q_COLYLDIR_5-2	AT4G37930.1 SHM1,SHMT1,STM serine transhydroxymethyltransferase 1
5	Glyma03g26550	**3699336:4200251**	500915	YIELD	Q_COLYLDIR_5-2	AT3G20810.2 2-oxoglutarate (2OG) and Fe(II)-dependent oxygenase superfamily protein
6	Glyma03g26580	**3699336:4200251**	500915	YIELD	Q_COLYLDIR_5-2	PF00179 PTHR24067:SF8,PTHR24067 KOG0896 GO:0016881 AT3G52560.1 MMZ4,UEV1D,UEV1D-4 ubiquitin E2 variant 1D-4
7	Glyma03g26590	**3699336:4200251**	500915	YIELD	Q_COLYLDIR_5-2	PF00106 PTHR24322 KOG0725 GO:0016491,GO:0008152 AT3G51680.1 NAD(P)-binding Rossmann-fold superfamily protein
8	Glyma03g30640	**3699336:4200251**	500915	YIELD	Q_COLYLDIR_5-2	PF03171 KOG4176 GO:0016491,GO:0016706,GO:0055114 AT4G02940.1 oxidoreductase, 2OG-Fe(II) oxygenase family protein
9	Glyma03g30650	**3699336:4200251**	500915	YIELD	Q_COLYLDIR_5-2	
10	Glyma03g30660	**3699336:4200251**	500915	YIELD	Q_COLYLDIR_5-2	AT2G48090.1
11	Glyma03g30673	**3699336:4200251**	500915	YIELD	Q_COLYLDIR_5-2	AT2G42190.1
12	Glyma03g30686	**3699336:4200251**	500915	YIELD	Q_COLYLDIR_5-2	PF00225 PTHR24115:SF176,PTHR24115 GO:0003777,GO:0005524,GO:0007018 AT2G21380.1 Kinesin motor family protein
13	Glyma03g30700	**3699336:4200251**	500915	YIELD	Q_COLYLDIR_5-2	
14	Glyma05g04580	**3183520:4168520**	985000	WHOLE-PLANT	Pl ht 24-3	PF00635 PTHR10809,PTHR10809:SF6 KOG0439 GO:0005198 AT2G45140.1 PVA12 plant VAP homolog 12
15	Glyma05g04590	**3183520:4168520**	985000	WHOLE-PLANT	Pl ht 24-3	PF00026 PTHR13683:SF98,PTHR13683 KOG1339 GO:0004190,GO:0006508 AT4G16563.1 Eukaryotic aspartyl protease family protein
16	Glyma05g04600	**3183520:4168520**	985000	WHOLE-PLANT	Pl ht 24-3	PF01596 PTHR11006:SF2,PTHR11006 KOG1501 2.1.1.- K11438 GO:0008171,GO:0008168 AT4G16570.1 ATPRMT7,PRMT7 protein arginine methyltransferase 7
17	Glyma07g04090	**2758000:4925000**	2167000	OTHER-SEED	Daidzein 2-3	PF08381 PTHR22870:SF36,PTHR22870 AT3G14000.1 ATBRXL2,BRX-LIKE2 DZC (Disease resistance/zinc finger/chromosome condensation-like region) domain containing protein
18	Glyma07g04100	**2758000:4925000**	2167000	OTHER-SEED	Daidzein 2-3	PF06972 PTHR12758,PTHR12758:SF13 AT3G13990.2 Kinase-related protein of unknown function (DUF1296)
19	Glyma07g11470	**6698000:9850000**	3152000	WHOLE-PLANT	Pl ht 19-6	PF00069 PTHR24055,PTHR24055:SF69 KOG0660 GO:0004672,GO:0005524,GO:0006468,GO:0016773,GO:0009103,GO:0016020 AT3G18040.1 MPK9 MAP kinase 9
20	Glyma07g11495	**6698000:9850000**	3152000	WHOLE-PLANT	Pl ht 19-6	PF00641 PTHR23111,PTHR23111:SF11 KOG4198 GO:0008270,GO:0005622 AT1G48570.1 zinc finger (Ran-binding) family protein
21	Glyma07g11520	**6698000:9850000**	3152000	WHOLE-PLANT	Pl ht 19-6	PF00043,PF00462 PTHR12782 KOG3029 GO:0009055,GO:0015035,GO:0045454 AT5G42150.1 Glutathione S-transferase family protein
22	Glyma07g11540	**6698000:9850000**	3152000	WHOLE-PLANT	Pl ht 19-6	PF00168 PTHR26357,PTHR26357:SF5 KOG1030 GO:0005515 AT3G17980.1 Calcium-dependent lipid-binding (CaLB domain) family protein
23	Glyma07g11550	**6698000:9850000**	3152000	WHOLE-PLANT	Pl ht 19-6	PF03547 K13947 GO:0055085,GO:0016021 AT1G73590.1 ATPIN1,PIN1 Auxin efflux carrier family protein
24	Glyma07g11560	**6698000:9850000**	3152000	WHOLE-PLANT	Pl ht 19-6	PF08060,PF08156,PF01798 PTHR10894 KOG2573 K14564 AT1G56110.1 NOP56 homolog of nucleolar protein NOP56
25	Glyma07g11580	**6698000:9850000**	3152000	WHOLE-PLANT	Pl ht 19-6	PF08241 PTHR10108,PTHR10108:SF328 KOG1269 2.1.1.103 K05929 GO:0004719,GO:0006464,GO:0008168,GO:0008610,GO:0008757,GO:0009312,GO:0009877,GO:0008152 AT3G18000.1 NMT1,PEAMT,XPL1 S-adenosyl-L-methionine-dependent methyltransferases superfamily protein

26	Glyma07g11590	**6698000:9850000**	3152000	WHOLE-PLANT	Pl ht 19-6	PF03643 PTHR12233,PTHR12233:SF2 KOG2717 GO:0007034,GO:0030904 AT1G48550.1 Vacuolar protein sorting-associated protein 26
27	Glyma07g11600	**6698000:9850000**	3152000	WHOLE-PLANT	Pl ht 19-6	PF01263 PTHR10091 KOG1604 GO:0016853,GO:0005975,GO:0019318 AT3G17940.1 Galactose mutarotase-like superfamily protein
28	Glyma07g11630	**6698000:9850000**	3152000	WHOLE-PLANT	Pl ht 19-6	AT3G17950.1
29	Glyma07g11650	**7683000:11623000**	3940000	WHOLE-PLANT	Cnpy ht 2-1	PF02104 PTHR23427,PTHR23427:SF2 KOG1563 GO:0016020 AT3G17910.1 SURF1 Surfeit locus 1 cytochrome c oxidase biogenesis
30	Glyma07g11660	**7683000:11623000**	3940000	WHOLE-PLANT	Cnpy ht 2-1	PF00234 AT1G73550.1 Bifunctional inhibitor/lipid-transfer protein/seed storage 2S albumin superfamily protein
31	Glyma07g11670	**7683000:11623000**	3940000	WHOLE-PLANT	Cnpy ht 2-1	PF00069 PTHR24356:SF87,PTHR24356 KOG0606 GO:0004672,GO:0005524,GO:0006468 AT3G17850.1 Protein kinase superfamily protein
32	Glyma07g11680	**7683000:11623000**	3940000	WHOLE-PLANT	Cnpy ht 2-1	PF00560,PF08263,PF00069 PTHR24420,PTHR24420:SF537 KOG1187 GO:0004672,GO:0005524,GO:0006468,GO:0005515 AT1G48480.1 RKL1 receptor-like kinase 1
33	Glyma07g11690	**7683000:11623000**	3940000	WHOLE-PLANT	Cnpy ht 2-1	PF00684,PF00226,PF01556 PTHR24076:SF80,PTHR24076 KOG0715 GO:0031072,GO:0051082,GO:0006457 AT3G17830.1 Molecular chaperone Hsp40/DnaJ family protein
34	Glyma07g11700	**7683000:11623000**	3940000	WHOLE-PLANT	Cnpy ht 2-1	PF03168 PTHR19957:SF11,PTHR19957 AT2G35980.1 ATNHL10,NHL10,YLS9 Late embryogenesis abundant (LEA) hydroxyproline-rich glycoprotein family
35	Glyma07g11710	**7683000:11623000**	3940000	WHOLE-PLANT	Cnpy ht 2-1	PF03168 PTHR19957:SF11,PTHR19957 AT2G35980.1 ATNHL10,NHL10,YLS9 Late embryogenesis abundant (LEA) hydroxyproline-rich glycoprotein family
36	Glyma07g11720	**7683000:11623000**	3940000	WHOLE-PLANT	Cnpy ht 2-1	PF03168 PTHR19957:SF11,PTHR19957 AT2G35980.1 ATNHL10,NHL10,YLS9 Late embryogenesis abundant (LEA) hydroxyproline-rich glycoprotein family
37	Glyma09g06250	**3370670:8049420**	4678750	OTHER-SEED	Sd cadmium 1-1	PF00702,PF00690,PF00122 PTHR24093:SF61,PTHR24093 KOG0205 3.6.3.6 K01535 GO:0000166,GO:0046872,GO:0003824,GO:0008152,GO:0016020,GO:0015662,GO:0006812,GO:0006754 AT4G30190.1 AHA2,HA2,PMA2 H()-ATPase 2
38	Glyma09g06260	**3370670:8049420**	4678750	OTHER-SEED	Sd cadmium 1-1	PF01582,PF00931 PTHR11017:SF20,PTHR11017 KOG4658 GO:0043531,GO:0005515,GO:0007165,GO:0005622,GO:0045087,GO:0031224,GO:0004888,GO:0006915,GO:0005524 AT5G17680.1 disease resistance protein (TIR-NBS-LRR class), putative
39	Glyma09g06290	**3370670:8049420**	4678750	OTHER-SEED	Sd cadmium 1-1	AT4G30180.1 sequence-specific DNA binding transcription factors;transcription regulators
40	Glyma09g06300	**3370670:8049420**	4678750	OTHER-SEED	Sd cadmium 1-1	PF01106 PTHR11178 KOG2358 GO:0005506,GO:0051536,GO:0016226 AT4G25910.1 ATCNFU3,NFU3 NFU domain protein 3
41	Glyma09g06310	**3370670:8049420**	4678750	OTHER-SEED	Sd cadmium 1-1	AT5G57340.1
42	Glyma09g06320	**3370670:8049420**	4678750	OTHER-SEED	Sd cadmium 1-1	PTHR23303:SF5,PTHR23303 AT3G62360.1 Carbohydrate-binding-like fold
43	Glyma09g06330	**3370670:8049420**	4678750	OTHER-SEED	Sd cadmium 1-1	PF07725,PF01582,PF00931 PTHR11017:SF20,PTHR11017 KOG4658 GO:0043531,GO:0005515,GO:0007165,GO:0005622,GO:0045087,GO:0031224,GO:0004888,GO:0006915,GO:0005524 AT5G41540.1 Disease resistance protein (TIR-NBS-LRR class) family
44	Glyma09g07240	**3370670:8049420**	4678750	OTHER-SEED	Sd cadmium 1-1	AT1G22770.1 FB,GI gigantea protein (GI)
45	Glyma09g07250	**3370670:8049420**	4678750	OTHER-SEED	Sd cadmium 1-1	PF01535 PTHR24015 AT1G12700.1 ATP binding;nucleic acid binding;helicases
46	Glyma09g07270	**3370670:8049420**	4678750	OTHER-SEED	Sd cadmium 1-1	
47	Glyma09g07290	**3370670:8049420**	4678750	OTHER-SEED	Sd cadmium 1-1	PF01535 PTHR24015 AT1G12700.1 ATP binding;nucleic acid binding;helicases
48	Glyma09g07310	**3370670:8049420**	4678750	OTHER-SEED	Sd cadmium 1-1	PF00504 PTHR21649 GO:0016020,GO:0009765 AT1G29930.1 AB140,CAB1,CAB140,LHCB1.3 chlorophyll A/B binding protein 1
49	Glyma09g07320	**3370670:8049420**	4678750	OTHER-SEED	Sd cadmium 1-1	PF04864,PF04863 PTHR11751,PTHR11751:SF162 GO:0016740,GO:0030170,GO:0009058,GO:0016846 AT1G34060.1 Pyridoxal phosphate (PLP)-dependent transferases superfamily protein
50	Glyma09g07330	**3370670:8049420**	4678750	OTHER-SEED	Sd cadmium 1-1	PF04863,PF04864 PTHR11751,PTHR11751:SF162 GO:0016846 AT1G34060.1 Pyridoxal phosphate (PLP)-dependent transferases superfamily protein
51	Glyma09g07340	**3370670:8049420**	4678750	OTHER-SEED	Sd cadmium 1-1	PF04864,PF04863 PTHR11751,PTHR11751:SF162 GO:0016740,GO:0030170,GO:0009058,GO:0016846 AT1G34060.1 Pyridoxal phosphate (PLP)-dependent transferases superfamily protein
52	Glyma09g07360	**3370670:8049420**	4678750	OTHER-SEED	Sd cadmium 1-1	PF01565,PF09265 PTHR13878,PTHR13878:SF19 KOG1231 1.5.99.12 K00279 GO:0008762,GO:0016491,GO:0050660,GO:0055114,GO:0019139,GO:0009690 AT5G56970.1 ATCKX3,CKX3 cytokinin oxidase 3

53	Glyma09g07400	**3370670:8049420**	4678750	OTHER-SEED	Sd cadmium 1-1	PF00154 PTHR22942,PTHR22942:SF1 KOG1433 K03553 GO:0003697,GO:0005524,GO:0006281,GO:0009432 AT2G19490.1 recA DNA recombination family protein
54	Glyma09g07410	**3370670:8049420**	4678750	OTHER-SEED	Sd cadmium 1-1	PF00956 PTHR11875:SF7,PTHR11875 KOG1507 GO:0006334,GO:0005634 AT5G56950.1 NAP1;3,NFA03,NFA3 nucleosome assembly protein 1;3
55	Glyma09g07420	**3370670:8049420**	4678750	OTHER-SEED	Sd cadmium 1-1	PF00886 PTHR12919:SF5,PTHR12919 KOG3419 GO:0003735,GO:0006412,GO:0005622,GO:0005840 AT5G56940.1 Ribosomal protein S16 family protein
56	Glyma09g07430	**3370670:8049420**	4678750	OTHER-SEED	Sd cadmium 1-1	PF01975 GO:0016787 AT1G72880.1 Survival protein SurE-like phosphatase/nucleotidase
57	Glyma09g07440	**3370670:8049420**	4678750	OTHER-SEED	Sd cadmium 1-1	PF12313,PF11900,PF00651 K14508 GO:0005515 AT1G64280.1 ATNPR1,NIM1,NPR1,SAI1 regulatory protein (NPR1)
58	Glyma09g07450	**3370670:8049420**	4678750	OTHER-SEED	Sd cadmium 1-1	AT1G72690.1
59	Glyma09g07470	**3370670:8049420**	4678750	OTHER-SEED	Sd cadmium 1-1	AT4G33050.3 EDA39 calmodulin-binding family protein
60	Glyma09g07490	**3370670:8049420**	4678750	OTHER-SEED	Sd cadmium 1-1	PF00069 PTHR11909:SF18,PTHR11909 KOG1163 2.7.11.1 K02218 GO:0004672,GO:0005524,GO:0006468 AT4G26100.1 CK1,CKL1 casein kinase 1
61	Glyma09g07520	**3370670:8049420**	4678750	OTHER-SEED	Sd cadmium 1-1	PF03062 PTHR10408 KOG0380 2.3.1.20,2.3.1.75,2.3.1.76 K11155 AT2G19450.1 ABX45,AS11,ATDGAT,DGAT1,RDS1,TAG1 membrane bound O-acyl transferase (MBOAT) family
62	Glyma09g07900	**3370670:8049420**	4678750	OTHER-SEED	Sd cadmium 1-1	PF00924 KOG4629 GO:0055085,GO:0016020 AT5G12080.1 ATMSL10,MSL10 mechanosensitive channel of small conductance-like 10
63	Glyma09g07920	**3370670:8049420**	4678750	OTHER-SEED	Sd cadmium 1-1	PF03357 PTHR10476:SF4,PTHR10476 KOG3230 K12191 GO:0015031 AT2G06530.1 VPS2.1 SNF7 family protein
64	Glyma09g07930	**3370670:8049420**	4678750	OTHER-SEED	Sd cadmium 1-1	PF08381 PTHR22870:SF36,PTHR22870 AT5G20540.1 ATBRXL4,BRX-LIKE4,BRXL4 BREVIS RADIX-like 4
65	Glyma09g11460	**10047000:11623000**	1576000	POD	Pod mat 5-3	PF01241 GO:0015979,GO:0009522,GO:0016020 AT1G30380.1 PSAK photosystem I subunit K
66	Glyma09g11480	**10047000:11623000**	1576000	POD	Pod mat 5-3	PF04050,PF02854 PTHR12839 KOG2051 K14327 GO:0005515,GO:0016070 AT2G39260.1 binding;RNA binding
67	Glyma09g11500	**10047000:11623000**	1576000	POD	Pod mat 5-3	
68	Glyma09g11510	**10047000:11623000**	1576000	POD	Pod mat 5-3	PF01535 PTHR24015:SF145,PTHR24015 AT4G21300.1 Tetratricopeptide repeat (TPR)-like superfamily protein
69	Glyma10g15910	**11504800:20885940**	9381140	POD	Pod num 2-2	PF00756 PTHR10061 KOG3101 3.1.2.12 K01070 GO:0008236,GO:0006508,GO:0018738,GO:0016023,GO:0004091 AT2G41530.1 ATSFGH,SFGH S-formylglutathione hydrolase
70	Glyma10g15950	**11504800:20885940**	9381140	POD	Pod num 2-2	PF00011 PTHR11527,PTHR11527:SF44 AT2G27140.1 HSP20-like chaperones superfamily protein
71	Glyma10g28060	**19798500:39380300**	19581800	YIELD	Q_COLYLDIR_10	AT5G03050.1
72	Glyma10g28070	**19798500:39380300**	19581800	YIELD	Q_COLYLDIR_10	AT3G62010.1
73	Glyma10g28080	**19798500:39380300**	19581800	YIELD	Q_COLYLDIR_10	PF01399 PTHR15350,PTHR15350:SF2 KOG2753 GO:0005515 AT5G15610.2 Proteasome component (PCI) domain protein
74	Glyma10g28100	**19798500:39380300**	19581800	YIELD	Q_COLYLDIR_10	PF00098,PF00271,PF08152,PF00270 PTHR24031:SF161,PTHR24031 KOG0331 GO:0003676,GO:0008270,GO:0005524,GO:0008026,GO:0004386,GO:0003723,GO:0005634 AT5G26742.1 emb1138 DEAD box RNA helicase (RH3)
75	Glyma10g28110	**19798500:39380300**	19581800	YIELD	Q_COLYLDIR_10	PF03619 PTHR23423:SF2,PTHR23423 KOG2641 AT3G05940.1 Protein of unknown function (DUF300)
76	Glyma10g28130	**19798500:39380300**	19581800	YIELD	Q_COLYLDIR_10	AT5G26731.1
77	Glyma10g28140	**19798500:39380300**	19581800	YIELD	Q_COLYLDIR_10	AT5G64860.1 DPE1 disproportionating enzyme
78	Glyma10g28150	**19798500:39380300**	19581800	YIELD	Q_COLYLDIR_10	
79	Glyma10g28170	**19798500:39380300**	19581800	YIELD	Q_COLYLDIR_10	PF01590,PF00512,PF08446,PF02518,PF00989,PF00360 K12120 GO:0008020,GO:0006355,GO:0009584,GO:0018298,GO:0000155,GO:0007165,GO:0016020,GO:0005515,GO:0005524 AT1G09570.1 FHY2,FRE1,HY8,PHYA phytochrome A
80	Glyma10g28180	**19798500:39380300**	19581800	YIELD	Q_COLYLDIR_10	PF03950,PF00749,PF00043 PTHR10119:SF16,PTHR10119 KOG1147 6.1.1.17 K01885 GO:0000166,GO:0005524,GO:0016876,GO:0043039,GO:0005737,GO:0004812,GO:0006418,GO:0006412 AT5G26710.1 Glutamyl/glutaminyl-tRNA synthetase, class Ic

81	Glyma10g28190	**19798500:39380300**	19581800	YIELD	Q_COLYLDIR_10	PF00190 GO:0045735 AT3G62020.1 GLP10 germin-like protein 10
82	Glyma10g28530	**19798500:39380300**	19581800	YIELD	Q_COLYLDIR_10	PF00069 PTHR24057 KOG0658 2.7.1.- K00924 GO:0004672,GO:0005524,GO:0006468 AT5G26751.1 ATSK11,SK 11 shaggy-related kinase 11
83	Glyma10g28540	**19798500:39380300**	19581800	YIELD	Q_COLYLDIR_10	PF00642,PF06220 PTHR16465 GO:0003676,GO:0008270 AT5G26749.1 C2H2 and C2HC zinc fingers superfamily protein
84	Glyma10g28560	**19798500:39380300**	19581800	YIELD	Q_COLYLDIR_10	PF01470 PTHR23402 KOG4755 GO:0006508 AT1G56700.1 Peptidase C15, pyroglutamyl peptidase I-like
85	Glyma10g28600	**19798500:39380300**	19581800	YIELD	Q_COLYLDIR_10	PTHR12847 KOG2355 K12608 AT5G02270.1 ATNAP9,NAP9 non-intrinsic ABC protein 9
86	Glyma10g28610	**19798500:39380300**	19581800	YIELD	Q_COLYLDIR_10	PF01501 PTHR11183,PTHR11183:SF2 GO:0016757 AT2G47180.1 AtGolS1,GolS1 galactinol synthase 1
87	Glyma10g28620	**19798500:39380300**	19581800	YIELD	Q_COLYLDIR_10	PF05832 PTHR13019 KOG3195 GO:0016021 AT1G09330.1
88	Glyma10g28640	**19798500:39380300**	19581800	YIELD	Q_COLYLDIR_10	PF04853 AT1G56560.1 Plant neutral invertase family protein
89	Glyma10g28650	**19798500:39380300**	19581800	YIELD	Q_COLYLDIR_10	PTHR10870 GO:0008853,GO:0006281,GO:0005634,GO:0003684 AT4G17760.1 damaged DNA binding;exodeoxyribonuclease IIIs
90	Glyma10g28680	**19798500:39380300**	19581800	YIELD	Q_COLYLDIR_10	PF04398 AT1G09310.1 Protein of unknown function, DUF538
91	Glyma10g28690	**19798500:39380300**	19581800	YIELD	Q_COLYLDIR_10	PF00557,PF05195 PTHR10804,PTHR10804:SF8 KOG2414 3.4.11.9 K01262 GO:0009987,GO:0004177,GO:0030145 AT1G09300.1 Metallopeptidase M24 family protein
92	Glyma10g28700	**19798500:39380300**	19581800	YIELD	Q_COLYLDIR_10	PF03959 PTHR18838,PTHR18838:SF15 KOG2551 GO:0016787 AT1G09280.1
93	Glyma10g28710	**19798500:39380300**	19581800	YIELD	Q_COLYLDIR_10	
94	Glyma10g28730	**19798500:39380300**	19581800	YIELD	Q_COLYLDIR_10	PF09597 AT5G26800.1
95	Glyma10g28740	**19798500:39380300**	19581800	YIELD	Q_COLYLDIR_10	AT1G09250.1 basic helix-loop-helix (bHLH) DNA-binding superfamily protein
96	Glyma10g28760	**19798500:39380300**	19581800	YIELD	Q_COLYLDIR_10	PF06963 PTHR11660:SF1,PTHR11660 KOG2601 GO:0005381,GO:0034755,GO:0016021 AT5G26820.1 ATIREG3,IREG3,MAR1,RTS3 iron-regulated protein 3
97	Glyma10g28800	**19798500:39380300**	19581800	YIELD	Q_COLYLDIR_10	PF00515 PTHR22904:SF95,PTHR22904 GO:0005515 AT1G56440.1 Tetratricopeptide repeat (TPR)-like superfamily protein
98	Glyma10g28810	**19798500:39380300**	19581800	YIELD	Q_COLYLDIR_10	PTHR12444 KOG1877 AT5G26850.1 Uncharacterized protein
99	Glyma10g28820	**19798500:39380300**	19581800	YIELD	Q_COLYLDIR_10	PF03790,PF00046,PF03789,PF03791 PTHR11850:SF45,PTHR11850 KOG0773 GO:0003700,GO:0043565,GO:0006355,GO:0003677,GO:0005634 AT1G23380.1 KNAT6,KNAT6L,KNAT6S KNOTTED1-like homeobox gene 6
100	Glyma10g28830	**19798500:39380300**	19581800	YIELD	Q_COLYLDIR_10	AT1G56423.1
101	Glyma10g28840	**19798500:39380300**	19581800	YIELD	Q_COLYLDIR_10	PF00004,PF02190,PF05362 PTHR10046,PTHR10046:SF23 KOG2004 3.4.21.- K08675 GO:0005524,GO:0004176,GO:0006508,GO:0004252,GO:0009378,GO:0006281,GO:0006310,GO:00168 87 AT5G26860.1 LON1,LON_ARA_ARA lon protease 1
102	Glyma10g28850	**19798500:39380300**	19581800	YIELD	Q_COLYLDIR_10	AT2G24100.1
103	Glyma10g28860	**19798500:39380300**	19581800	YIELD	Q_COLYLDIR_10	AT1G56420.1
104	Glyma10g28880	**19798500:39380300**	19581800	YIELD	Q_COLYLDIR_10	PF03030 GO:0004427,GO:0009678,GO:0015992,GO:0016020 AT1G15690.1 ATAVP3,AtVHP1;1,AVP-3,AVP1 Inorganic H pyrophosphatase family protein
105	Glyma10g28890	**19798500:39380300**	19581800	YIELD	Q_COLYLDIR_10	PF00262 PTHR11073 KOG0674 K08057 GO:0005509 AT1G09210.1 AtCRT1b,CRT1b calreticulin 1b
106	Glyma10g28900	**19798500:39380300**	19581800	YIELD	Q_COLYLDIR_10	PF00582 GO:0006950 AT3G62550.1 Adenine nucleotide alpha hydrolases-like superfamily protein
107	Glyma10g28910	**19798500:39380300**	19581800	YIELD	Q_COLYLDIR_10	PF00025 PTHR11711,PTHR11711:SF12 KOG0077 3.6.5.- K07953 GO:0005525,GO:0007264,GO:0004871,GO:0019001,GO:0007186,GO:0005622,GO:0006886 AT4G02080.1 ASAR1,ATSAR2,ATSARA1C,SAR2 secretion-associated RAS super family 2
108	Glyma10g28930	**19798500:39380300**	19581800	YIELD	Q_COLYLDIR_10	PF01535 PTHR24015,PTHR24015:SF76 AT1G09190.1 Tetratricopeptide repeat (TPR)-like superfamily protein
109	Glyma10g28950	**19798500:39380300**	19581800	YIELD	Q_COLYLDIR_10	PTHR21726,PTHR21726:SF12 AT5G26910.1
110	Glyma10g28960	**19798500:39380300**	19581800	YIELD	Q_COLYLDIR_10	PF00270 PTHR13710:SF13,PTHR13710 KOG0331 GO:0003676,GO:0005524,GO:0008026,GO:0006310 AT3G05740.1 RECQI1 RECQ helicase l1

111	Glyma10g28970	**19798500:39380300**	19581800	YIELD	Q_COLYLDIR_10	PF12023 AT3G05725.1 Protein of unknown function (DUF3511)
112	Glyma10g28980	**19798500:39380300**	19581800	YIELD	Q_COLYLDIR_10	PF01612 PTHR13620 KOG2207 GO:0003676,GO:0008408,GO:0006139,GO:0005622 AT1G56310.1 Polynucleotidyl transferase, ribonuclease H-like superfamily protein
113	Glyma10g28990	**19798500:39380300**	19581800	YIELD	Q_COLYLDIR_10	PF07887 AT4G25800.1 Calmodulin-binding protein
114	Glyma10g29000	**19798500:39380300**	19581800	YIELD	Q_COLYLDIR_10	PF00514,PF01749 PTHR23316 KOG0166 GO:0005515,GO:0008565,GO:0006606,GO:0005634,GO:0005737,GO:0044183,GO:0006886,GO:0005643 AT3G06720.1 AIMP ALPHA,AT-IMP,ATKAP ALPHA,IMPA-1,IMPA1 importin alpha isoform 1
115	Glyma10g29010	**19798500:39380300**	19581800	YIELD	Q_COLYLDIR_10	PF00929 PTHR13058,PTHR13058:SF10 KOG4793 AT5G26940.1 Polynucleotidyl transferase, ribonuclease H-like superfamily protein
116	Glyma10g29020	**19798500:39380300**	19581800	YIELD	Q_COLYLDIR_10	PF00804,PF05739 PTHR19957,PTHR19957:SF57 KOG0809 GO:0016020,GO:0005515 AT3G05710.2 ATSYP43,SYP43 syntaxin of plants 43
117	Glyma10g29030	**19798500:39380300**	19581800	YIELD	Q_COLYLDIR_10	PF05605 AT3G05700.1 Drought-responsive family protein
118	Glyma10g29040	**19798500:39380300**	19581800	YIELD	Q_COLYLDIR_10	PF00628 PTHR15242 KOG1493 GO:0005515 AT3G05670.1 RING/U-box protein
119	Glyma10g29050	**19798500:39380300**	19581800	YIELD	Q_COLYLDIR_10	PF00225,PF00307 PTHR24115,PTHR24115:SF135 KOG0239 GO:0003777,GO:0005524,GO:0007018,GO:0005515 AT5G27000.1 ATK4,KATD kinesin 4
120	Glyma10g29060	**19798500:39380300**	19581800	YIELD	Q_COLYLDIR_10	PF00481 PTHR13832:SF148,PTHR13832 KOG0698 GO:0003824 AT1G09160.1 Protein phosphatase 2C family protein
121	Glyma10g29070	**19798500:39380300**	19581800	YIELD	Q_COLYLDIR_10	PF00436 PTHR10302 KOG1653 GO:0003697,GO:0006260 AT4G11060.1 MTSSB mitochondrially targeted single-stranded DNA binding protein
122	Glyma10g29080	**19798500:39380300**	19581800	YIELD	Q_COLYLDIR_10	PF03371 PTHR23142 KOG2889 AT2G40650.1 PRP38 family protein
123	Glyma10g29090	**19798500:39380300**	19581800	YIELD	Q_COLYLDIR_10	PF00400 PTHR22847,PTHR22847:SF52 KOG0266 GO:0005515 AT5G27030.1 TPR3 TOPLESS-related 3
124	Glyma10g29100	**19798500:39380300**	19581800	YIELD	Q_COLYLDIR_10	PF00481 PTHR13832:SF94,PTHR13832 KOG0698 GO:0003824 AT5G27930.1 Protein phosphatase 2C family protein
125	Glyma10g29110	**19798500:39380300**	19581800	YIELD	Q_COLYLDIR_10	PTHR18896,PTHR18896:SF2 AT3G05630.1 PDLZ2,PLDP2,PLDZETA2 phospholipase D P2
126	Glyma10g29120	**19798500:39380300**	19581800	YIELD	Q_COLYLDIR_10	PTHR23083,PTHR23083:SF219 AT3G05625.1 Tetratricopeptide repeat (TPR)-like superfamily protein
127	Glyma10g29130	**19798500:39380300**	19581800	YIELD	Q_COLYLDIR_10	PF00646 GO:0005515 AT1G09155.1 AtPP2-B15,PP2-B15 phloem protein 2-B15
128	Glyma10g29140	**19798500:39380300**	19581800	YIELD	Q_COLYLDIR_10	PF07173 AT1G56230.1 Protein of unknown function (DUF1399)
129	Glyma10g29150	**19798500:39380300**	19581800	YIELD	Q_COLYLDIR_10	PF04043,PF01095 PTHR22931,PTHR22931:SF5 GO:0030599,GO:0042545,GO:0005618,GO:0004857 AT4G02330.1 ATPMEPCRB Plant invertase/pectin methylesterase inhibitor superfamily
130	Glyma10g29160	**19798500:39380300**	19581800	YIELD	Q_COLYLDIR_10	PF01095,PF04043 PTHR22931,PTHR22931:SF5 GO:0030599,GO:0042545,GO:0005618,GO:0004857 AT3G05610.1 Plant invertase/pectin methylesterase inhibitor superfamily
131	Glyma10g29170	**19798500:39380300**	19581800	YIELD	Q_COLYLDIR_10	PF00828 PTHR10934 KOG1714 GO:0006412,GO:0005840,GO:0005622,GO:0003735 AT3G05590.1 RPL18 ribosomal protein L18
132	Glyma10g29180	**19798500:39380300**	19581800	YIELD	Q_COLYLDIR_10	PF03024 AT5G27830.1
133	Glyma10g29730	**19798500:39380300**	19581800	YIELD	Q_COLYLDIR_10	PF03834 PTHR12749 KOG2841 K10849 GO:0003684,GO:0004519,GO:0006281,GO:0005634 AT3G05210.1 ERCC1,UVR7 nucleotide repair protein, putative
134	Glyma10g29750	**19798500:39380300**	19581800	YIELD	Q_COLYLDIR_10	PF00097 PTHR22764,PTHR22764:SF21 AT3G05200.1 ATL6 RING/U-box superfamily protein
135	Glyma10g29760	**19798500:39380300**	19581800	YIELD	Q_COLYLDIR_10	PF00010 PTHR11534:SF7,PTHR11534 KOG4029 GO:0030528,GO:0006355 AT1G72210.1 basic helix-loop-helix (bHLH) DNA-binding superfamily protein
136	Glyma10g29770	**19798500:39380300**	19581800	YIELD	Q_COLYLDIR_10	PF01063 PTHR11825,PTHR11825:SF13 KOG0975 GO:0003824,GO:0008152 AT5G27410.2 D-aminoacid aminotransferase-like PLP-dependent enzymes superfamily protein
137	Glyma10g30220	**19798500:39380300**	19581800	YIELD	Q_COLYLDIR_10	PF01593 PTHR10742,PTHR10742:SF6 KOG0029 1.14.99.30 K00514 GO:0016491,GO:0055114 AT3G04870.1 PDE181,SPC1,ZDS zeta-carotene desaturase
138	Glyma10g30230	**19798500:39380300**	19581800	YIELD	Q_COLYLDIR_10	PF10184 AT3G04890.1 Uncharacterized conserved protein (DUF2358)
139	Glyma10g30240	**19798500:39380300**	19581800	YIELD	Q_COLYLDIR_10	PF05910 AT5G11000.1 Plant protein of unknown function (DUF868)
140	Glyma10g30250	**19798500:39380300**	19581800	YIELD	Q_COLYLDIR_10	PF00036 AT1G54530.1 Calcium-binding EF hand family protein
141	Glyma10g30260	**19798500:39380300**	19581800	YIELD	Q_COLYLDIR_10	PF05910 AT5G28150.1 Plant protein of unknown function (DUF868)

142	Glyma10g30270	**19798500:39380300**	19581800	YIELD	Q_COLYLDIR_10	PF03096 PTHR11034,PTHR11034:SF3 KOG2931 AT5G56750.1 NDL1 N-MYC downregulated-like 1
143	Glyma10g38170	**44778100:59592500**	14814400	YIELD	Q_CLAYLDRF_10-1	
144	Glyma10g38190	**44778100:59592500**	14814400	YIELD	Q_CLAYLDRF_10-1	PF04979 PTHR12398:SF5,PTHR12398 GO:0004864,GO:0009966,GO:0043666 AT5G52200.1 phosphoprotein phosphatase inhibitors
145	Glyma10g38200	**44778100:59592500**	14814400	YIELD	Q_CLAYLDRF_10-1	AT5G52190.1 Sugar isomerase (SIS) family protein
146	Glyma10g38220	**44778100:59592500**	14814400	YIELD	Q_CLAYLDRF_10-1	
147	Glyma10g38230	**44778100:59592500**	14814400	YIELD	Q_CLAYLDRF_10-1	PF10268 AT5G52180.1
148	Glyma10g38240	**44778100:59592500**	14814400	YIELD	Q_CLAYLDRF_10-1	PF00447 PTHR10015,PTHR10015:SF7 GO:0003700,GO:0043565,GO:0006355,GO:0005634 AT5G62020.1 AT-HSFB2A,HSFB2A heat shock transcription factor B2A
149	Glyma10g38270	**44778100:59592500**	14814400	YIELD	Q_CLAYLDRF_10-1	PF00023 PTHR24136,PTHR24136:SF158 KOG0502 GO:0005515 AT5G07270.1 XBAT33 XB3 ortholog 3 in Arabidopsis thaliana
150	Glyma12g16940	**16548000:17927000**	1379000	OTHER-SEED	Daidzein 2-2	PF01397,PF03936 GO:0010333,GO:0016829,GO:0008152,GO:0000287 AT2G24210.1 TPS10 terpene synthase 10
151	Glyma12g17240	**16548000:17927000**	1379000	OTHER-SEED	Daidzein 2-2	PF02542 GO:0008685,GO:0016114 AT1G63970.1 ISPF,MECPS isoprenoid F
152	Glyma12g17510	**16548000:17927000**	1379000	OTHER-SEED	Daidzein 2-2	PF03321 K14487 AT5G54510.1 DFL1,GH3.6 Auxin-responsive GH3 family protein
153	Glyma12g17550	**16548000:17927000**	1379000	OTHER-SEED	Daidzein 2-2	PF05207,PF00226 GO:0031072 AT4G10130.1 DNAJ heat shock N-terminal domain-containing protein
154	Glyma12g17560	**16548000:17927000**	1379000	OTHER-SEED	Daidzein 2-2	PF05093 PTHR13273,PTHR13273:SF5 KOG4020 AT5G18400.2 Cytokine-induced anti-apoptosis inhibitor 1, Fe-S biogenesis
155	Glyma12g17580	**16548000:17927000**	1379000	OTHER-SEED	Daidzein 2-2	
156	Glyma12g17630	**16548000:17927000**	1379000	OTHER-SEED	Daidzein 2-2	AT3G20640.1 basic helix-loop-helix (bHLH) DNA-binding superfamily protein
157	Glyma12g17660	**16548000:17927000**	1379000	OTHER-SEED	Daidzein 2-2	PF00810 PTHR10585,PTHR10585:SF10 GO:0046923,GO:0006621,GO:0016021 AT1G19970.1 ER lumen protein retaining receptor family protein
158	Glyma12g17680	**16548000:17927000**	1379000	OTHER-SEED	Daidzein 2-2	PF03358 KOG3135 GO:0010181,GO:0016491,GO:0009055,GO:0050662 AT5G54500.1 FQR1 flavodoxin-like quinone reductase 1
159	Glyma13g00200	**0:2239890**	2239890	WHOLE-PLANT	Node num 1-5	PF00320 PTHR10071 GO:0003700,GO:0008270,GO:0043565,GO:0006355 AT5G56860.1 GATA21,GNC GATA type zinc finger transcription factor family protein
160	Glyma13g00230	**0:2239890**	2239890	WHOLE-PLANT	Node num 1-5	PF01399 PTHR12732 KOG2688 GO:0005515 AT2G19560.1 EER5 proteasome family protein
161	Glyma13g00240	**0:2239890**	2239890	WHOLE-PLANT	Node num 1-5	
162	Glyma13g00280	**0:2239890**	2239890	WHOLE-PLANT	Node num 1-5	PF00722,PF06955 PTHR10963,PTHR10963:SF14 2.4.1.207 K08235 GO:0004553,GO:0005975,GO:0016762,GO:0006073,GO:0005618,GO:0048046 AT4G25810.1 XTH23,XTR6 xyloglucan endotransglycosylase 6
163	Glyma13g00300	**0:2239890**	2239890	WHOLE-PLANT	Node num 1-5	PF03005 PTHR13533,PTHR13533:SF3 AT5G20590.1 TBL5 TRICHOME BIREFRINGENCE-LIKE 5
164	Glyma13g00310	**0:2239890**	2239890	WHOLE-PLANT	Node num 1-5	PF02183,PF00046 PTHR24326,PTHR24326:SF218 GO:0003700,GO:0043565,GO:0006355,GO:0003677,GO:0005634 AT5G06710.1 HAT14 homeobox from Arabidopsis thaliana
165	Glyma13g00320	**0:2239890**	2239890	WHOLE-PLANT	Node num 1-5	
166	Glyma13g00350	**0:2239890**	2239890	WHOLE-PLANT	Node num 1-5	PF05915 PTHR15664 KOG4753 AT4G29850.1 Eukaryotic protein of unknown function (DUF872)
167	Glyma13g00360	**0:2239890**	2239890	WHOLE-PLANT	Node num 1-5	PF09753 AT3G55600.1 Membrane fusion protein Use1
168	Glyma13g00370	**0:2239890**	2239890	WHOLE-PLANT	Node num 1-5	PF00069 PTHR24420:SF531,PTHR24420 KOG1187 GO:0004672,GO:0005524,GO:0006468 AT2G17220.1 Protein kinase superfamily protein
169	Glyma13g00380	**0:2239890**	2239890	WHOLE-PLANT	Node num 1-5	PF10533,PF03106 GO:0003700,GO:0043565,GO:0006355 AT4G31550.1 ATWRKY11,WRKY11 WRKY DNA-binding protein 11
170	Glyma13g00390	**0:2239890**	2239890	WHOLE-PLANT	Node num 1-5	PF03547 K13947 GO:0055085,GO:0016021 AT5G57090.1 AGR,AGR1,ATPIN2,EIR1,PIN2,WAV6 Auxin efflux carrier family protein
171	Glyma13g00430	**0:2239890**	2239890	WHOLE-PLANT	Node num 1-5	PF05022 PTHR23216 AT5G57120.1
172	Glyma13g00440	**0:2239890**	2239890	WHOLE-PLANT	Node num 1-5	AT5G57123.1

173	Glyma13g00450	**0:2239890**	2239890	WHOLE-PLANT	Node num 1-5	PF12146 PTHR11614,PTHR11614:SF20 KOG1455 AT1G52760.1 LysoPL2 lysophospholipase 2
174	Glyma13g00460	**0:2239890**	2239890	WHOLE-PLANT	Node num 1-5	PF02861 PTHR11638:SF2,PTHR11638 KOG1051 GO:0019538 AT4G29920.1 Double Clp-N motif-containing P-loop nucleoside triphosphate hydrolases superfamily
175	Glyma13g00470	**0:2239890**	2239890	WHOLE-PLANT	Node num 1-5	PF01532 PTHR11742:SF6,PTHR11742 KOG2204 GO:0004571,GO:0005509,GO:0016020 AT1G51590.1 MANIB,MNS1 alpha-mannosidase 1
176	Glyma13g00480	**0:2239890**	2239890	WHOLE-PLANT	Node num 1-5	PF00010 PTHR11514:SF7,PTHR11514 GO:0030528,GO:0006355 AT5G57150.2 basic helix-loop-helix (bHLH) DNA-binding superfamily protein
177	Glyma13g00490	**0:2239890**	2239890	WHOLE-PLANT	Node num 1-5	PF10253 PTHR13621:SF3,PTHR13621 KOG3903 AT2G19270.1
178	Glyma13g00500	**0:2239890**	2239890	WHOLE-PLANT	Node num 1-5	PF01187 PTHR11954:SF3,PTHR11954 KOG1759 AT5G57170.1 Tautomerase/MIF superfamily protein
179	Glyma13g00510	**0:2239890**	2239890	WHOLE-PLANT	Node num 1-5	PF00013 PTHR10288,PTHR10288:SF53 KOG2190 GO:0003723 AT4G26000.1 PEP RNA-binding KH domain-containing protein
180	Glyma13g00520	**0:2239890**	2239890	WHOLE-PLANT	Node num 1-5	PF06203 GO:0005515 AT5G57180.2 CIA2 chloroplast import apparatus 2
181	Glyma13g00530	**0:2239890**	2239890	WHOLE-PLANT	Node num 1-5	PF00383 PTHR11079 KOG1018 GO:0008270,GO:0016787 AT1G48175.1 emb2191 Cytidine/deoxycytidylate deaminase family protein
182	Glyma13g00540	**0:2239890**	2239890	WHOLE-PLANT	Node num 1-5	PF00628,PF00046 PTHR12628:SF11,PTHR12628 GO:0003700,GO:0043565,GO:0006355,GO:0005515 AT4G29940.1 PRHA pathogenesis related homeodomain protein A
183	Glyma13g00550	**0:2239890**	2239890	WHOLE-PLANT	Node num 1-5	PF07651 PTHR22951,PTHR22951:SF1 KOG0251 GO:0005543 AT4G25940.1 ENTH/ANTH/VHS superfamily protein
184	Glyma13g00560	**0:2239890**	2239890	WHOLE-PLANT	Node num 1-5	AT1G78890.1
185	Glyma13g00570	**0:2239890**	2239890	WHOLE-PLANT	Node num 1-5	PF00566 PTHR22957:SF24,PTHR22957 KOG1091 GO:0005097,GO:0032313,GO:0005622 AT4G29950.1 Ypt/Rab-GAP domain of gyp1p superfamily protein
186	Glyma13g00580	**0:2239890**	2239890	WHOLE-PLANT	Node num 1-5	PF05922,PF00082,PF06280,PF02225 PTHR10795,PTHR10795:SF17 KOG1153 GO:0004252,GO:0006508,GO:0042802,GO:0043086,GO:0005618,GO:0016020 AT4G30020.1 PA-domain containing subtilase family protein
187	Glyma13g00590	**0:2239890**	2239890	WHOLE-PLANT	Node num 1-5	PF01237 PTHR10972:SF27,PTHR10972 KOG2210 AT4G25850.1 ORP4B OSBP(oxysterol binding protein)-related protein 4B
188	Glyma13g00595	**0:2239890**	2239890	WHOLE-PLANT	Node num 1-5	PF01237 PTHR10972 KOG2210 AT4G25850.2 ORP4B OSBP(oxysterol binding protein)-related protein 4B
189	Glyma13g00600	**0:2239890**	2239890	WHOLE-PLANT	Node num 1-5	AT5G66580.1
190	Glyma13g00610	**0:2239890**	2239890	WHOLE-PLANT	Node num 1-5	PF02485 GO:0008375,GO:0016020 AT2G19160.1 Core-2/I-branching beta-1,6-N-acetylglucosaminyltransferase family protein
191	Glyma13g00630	**0:2239890**	2239890	WHOLE-PLANT	Node num 1-5	PF00702,PF00122 PTHR24093,PTHR24093:SF31 KOG0207 3.6.3.3,3.6.3.5 K01534 GO:0000166,GO:0046872,GO:0003824,GO:0008152 AT4G30110.1 ATHMA2,HMA2 heavy metal atpase 2
192	Glyma13g00640	**0:2239890**	2239890	WHOLE-PLANT	Node num 1-5	PTHR11132 AT3G11320.1 Nucleotide-sugar transporter family protein
193	Glyma13g08460	**8668000:10441000**	1773000	OTHER-SEED	Glycitein 2-3	PF06017 GO:0003774,GO:0016459 AT5G53310.1 myosin heavy chain-related
194	Glyma14g03490	**1544480:2474320**	929840	YIELD	Flr num 1-6	PF02458 GO:0016747 AT1G32910.1 HXXXD-type acyl-transferase family protein
195	Glyma14g03500	**1544480:2474320**	929840	YIELD	Flr num 1-6	PF00494 KOG1459 GO:0016740,GO:0009058 AT5G17230.1 PSY PHYTOENE SYNTHASE
196	Glyma14g03510	**1544480:2474320**	929840	YIELD	Flr num 1-6	PF11900,PF00651,PF12313 KOG0512 GO:0005515 AT5G45110.1 ATNPR3,NPR3 NPR1-like protein 3
197	Glyma14g03520	**1544480:2474320**	929840	YIELD	Flr num 1-6	PF03798 PTHR13439 KOG4561 GO:0016021 AT4G19645.1 TRAM, LAG1 and CLN8 (TLC) lipid-sensing domain containing
198	Glyma14g03550	**1544480:2474320**	929840	YIELD	Flr num 1-6	PF00400 PTHR22838 KOG0293 GO:0005515 AT5G08560.1 transducin family protein / WD-40 repeat family protein
199	Glyma14g03560	**1544480:2474320**	929840	YIELD	Flr num 1-6	PF01789 GO:0005509,GO:0015979,GO:0009523,GO:0009654,GO:0019898 AT1G06680.1 OE23,OEE2,PSBP-1,PSII-P photosystem II subunit P-1
200	Glyma14g03570	**1544480:2474320**	929840	YIELD	Flr num 1-6	PF02453 PTHR10994:SF27,PTHR10994 KOG1792 GO:0005783 AT3G10260.3 Reticulon family protein
201	Glyma14g03580	**1544480:2474320**	929840	YIELD	Flr num 1-6	
202	Glyma14g03590	**1544480:2474320**	929840	YIELD	Flr num 1-6	PF09753 KOG2678 AT3G55600.1 Membrane fusion protein Use1

203	Glyma14g03600	**1544480:2474320**	929840	YIELD	Flr num 1-6	PF00010 PTHR12565,PTHR12565:SF7 GO:0030528,GO:0006355 AT2G43010.2 PIF4,SRL2 phytochrome interacting factor 4
204	Glyma14g03610	**1544480:2474320**	929840	YIELD	Flr num 1-6	PF01593 PTHR10742:SF30,PTHR10742 KOG0029 GO:0009055,GO:0016491,GO:0055114,GO:0050660,GO:0008033 AT2G43020.1 ATPAO2,PAO2 polyamine oxidase 2
205	Glyma14g03620	**1544480:2474320**	929840	YIELD	Flr num 1-6	PF01554 PTHR11206:SF47,PTHR11206 KOG1347 GO:0015238,GO:0015297,GO:0006855,GO:0055085,GO:0016020 AT3G59030.1 ATTT12,TT12 MATE efflux family protein
206	Glyma14g03630	**1544480:2474320**	929840	YIELD	Flr num 1-6	KOG0978 AT2G34780.1 EMB1611,MEE22 maternal effect embryo arrest 22
207	Glyma14g03640	**1544480:2474320**	929840	YIELD	Flr num 1-6	PF01535 PTHR24015 AT5G64320.1 Pentatricopeptide repeat (PPR) superfamily protein
208	Glyma14g03650	**1544480:2474320**	929840	YIELD	Flr num 1-6	PF02309,PF06507,PF02362 GO:0006355,GO:0005634,GO:0003677,GO:0009725 AT1G30330.2 ARF6 auxin response factor 6
209	Glyma14g03660	**1544480:2474320**	929840	YIELD	Flr num 1-6	PF00234 AT4G33355.1 Bifunctional inhibitor/lipid-transfer protein/seed storage 2S albumin superfamily protein
210	Glyma14g03710	**1544480:2474320**	929840	YIELD	Flr num 1-6	PF00295 GO:0004650,GO:0005975 AT4G23820.1 Pectin lyase-like superfamily protein
211	Glyma14g03730	**1544480:2474320**	929840	YIELD	Flr num 1-6	AT4G33355.1 Bifunctional inhibitor/lipid-transfer protein/seed storage 2S albumin superfamily protein
212	Glyma14g03740	**1544480:2474320**	929840	YIELD	Flr num 1-6	PTHR24015:SF11,PTHR24015 AT3G59300.1 Pentatricopeptide repeat (PPR) superfamily protein
213	Glyma14g13480	**10874400:14120960**	3246560	LEAF-STEM	Lf lgth 10-4	PF00155 PTHR11879 GO:0016740,GO:0030170,GO:0009058,GO:0016769,GO:0008483,GO:0006520 AT4G31990.3 AAT3,ASP5,ATAAT1 aspartate aminotransferase 5
214	Glyma14g13490	**10874400:14120960**	3246560	LEAF-STEM	Lf lgth 10-4	PF00069 PTHR24420:SF449,PTHR24420 KOG1187 GO:0004672,GO:0005524,GO:0006468 AT2G25220.1 Protein kinase superfamily protein
215	Glyma14g13520	**10874400:14120960**	3246560	LEAF-STEM	Lf lgth 10-4	PF08626 PTHR21512,PTHR21512:SF5 AT5G11040.1 AtTRS120,TRS120 TRS120
216	Glyma14g13780	**10874400:14120960**	3246560	LEAF-STEM	Lf lgth 10-4	AT5G25265.1
217	Glyma14g14140	**10874400:14120960**	3246560	LEAF-STEM	Lf lgth 10-4	
218	Glyma14g14210	**10874400:14120960**	3246560	LEAF-STEM	Lf lgth 10-4	
219	Glyma14g17640	**14223400:21906400**	7683000	LEAF-STEM	Lf lgth 10-3	AT5G39380.1 Plant calmodulin-binding protein-related
220	Glyma14g17650	**14223400:21906400**	7683000	LEAF-STEM	Lf lgth 10-3	PF01535,PF00571 PTHR24015 GO:0005515 AT5G10690.1 pentatricopeptide (PPR) repeat-containing protein / CBS domain-containing protein
221	Glyma14g17660	**14223400:21906400**	7683000	LEAF-STEM	Lf lgth 10-3	AT5G10695.1
222	Glyma15g14260	**6107000:22655000**	16548000	LEAF-STEM	Lflt shape 8-8	AT1G19980.1 cytomatrix protein-related
223	Glyma15g14280	**6107000:22655000**	16548000	LEAF-STEM	Lflt shape 8-8	PF00542 PTHR11809,PTHR11809:SF6 KOG1715 GO:0003735,GO:0006412,GO:0005622,GO:0005840 AT3G06040.1 Ribosomal protein L12/ ATP-dependent Clp protease adaptor protein
224	Glyma15g14290	**6107000:22655000**	16548000	LEAF-STEM	Lflt shape 8-8	PF03092 PTHR24003,PTHR24003:SF252 KOG2615 AT1G64890.1 Major facilitator superfamily protein
225	Glyma15g14320	**6107000:22655000**	16548000	LEAF-STEM	Lflt shape 8-8	PF00538,PF00249 PTHR21717 GO:0003677,GO:0006334,GO:0000786,GO:0005634 AT1G49950.1 ATTRB1,TRB1 telomere repeat binding factor 1
226	Glyma15g14330	**6107000:22655000**	16548000	LEAF-STEM	Lflt shape 8-8	PF00067 PTHR24286:SF24,PTHR24286 KOG0157 1.14.13.79 K04123 GO:0005506,GO:0009055,GO:0016705,GO:0020037,GO:0055114 AT2G32440.1 ATKAO2,CYP88A4,KAO2 ent-kaurenoic acid hydroxylase 2
227	Glyma15g14340	**6107000:22655000**	16548000	LEAF-STEM	Lflt shape 8-8	PF11955 AT2G39120.1 Ubiquitin carboxyl-terminal hydrolase family protein
228	Glyma15g14350	**6107000:22655000**	16548000	LEAF-STEM	Lflt shape 8-8	PF00191 PTHR10502:SF10,PTHR10502 KOG0819 GO:0005509,GO:0005544 AT5G12380.1 ANNAT8 annexin 8
229	Glyma15g14360	**6107000:22655000**	16548000	LEAF-STEM	Lflt shape 8-8	PF00106 PTHR24322:SF18,PTHR24322 KOG1611 GO:0016491,GO:0008152 AT4G20760.1 NAD(P)-binding Rossmann-fold superfamily protein
230	Glyma15g14371	**6107000:22655000**	16548000	LEAF-STEM	Lflt shape 8-8	PF03106 GO:0003700,GO:0043565,GO:0006355 AT1G30650.1 AR411,ATWRKY14,WRKY14 WRKY DNA-binding protein 14
231	Glyma15g14380	**6107000:22655000**	16548000	LEAF-STEM	Lflt shape 8-8	PF00501 PTHR24095,PTHR24095:SF26 GO:0003824,GO:0008152 AT3G16910.1 AAE7,ACN1 acyl-activating enzyme 7

232	Glyma15g14390	**6107000:22655000**	16548000	LEAF-STEM	Lflt shape 8-8	PF00069 PTHR24056:SF109,PTHR24056 KOG0594 2.7.11.22 K02087 GO:0004672,GO:0005524,GO:0006468 AT3G48750.1 CDC2,CDC2A,CDC2AAT,CDK2,CDKA1,CDKA;1 cell division control 2
233	Glyma15g14420	**6107000:22655000**	16548000	LEAF-STEM	Lflt shape 8-8	AT1G53645.1 hydroxyproline-rich glycoprotein family protein
234	Glyma15g14433	**6107000:22655000**	16548000	LEAF-STEM	Lflt shape 8-8	PF01370 PTHR10366,PTHR10366:SF39 KOG1371 5.1.3.5 K12448 GO:0016491,GO:0008152,GO:0003854,GO:0016616,GO:0006694,GO:0055114,GO:0003824,GO:0050662,GO:0044237,GO:0009058,GO:0008831,GO:0045226,GO:0006012,GO:0003978 AT1G30620.1 HSR8,MUR4,UXE1 NAD(P)-binding Rossmann-fold superfamily protein
235	Glyma15g14633	**6107000:22655000**	16548000	LEAF-STEM	Lflt shape 8-8	PF03171 PTHR10209 GO:0016491,GO:0016706,GO:0055114 AT1G47990.1 ATGA2OX4,GA2OX4 gibberellin 2-oxidase 4
236	Glyma15g14660	**6107000:22655000**	16548000	LEAF-STEM	Lflt shape 8-8	AT2G43630.1
237	Glyma15g14690	**6107000:22655000**	16548000	LEAF-STEM	Lflt shape 8-8	AT5G49610.1 F-box family protein
238	Glyma15g14700	**6107000:22655000**	16548000	LEAF-STEM	Lflt shape 8-8	PF00226 PTHR24078 KOG0720 GO:0031072 AT5G49580.1 Chaperone DnaJ-domain superfamily protein
239	Glyma15g14710	**6107000:22655000**	16548000	LEAF-STEM	Lflt shape 8-8	PF01250 KOG4708 GO:0003735,GO:0019843,GO:0006412,GO:0005840 AT3G17170.1 RFC3 Translation elongation factor EF1B/ribosomal protein S6 family protein
240	Glyma15g14720	**6107000:22655000**	16548000	LEAF-STEM	Lflt shape 8-8	AT1G47980.1
241	Glyma15g23270	**6107000:22655000**	16548000	LEAF-STEM	Lflt shape 8-8	PF01655 PTHR23413 GO:0003735,GO:0006412,GO:0005622,GO:0005840 AT4G18100.1 Ribosomal protein L32e
242	Glyma16g08990	**8471000:12411000**	3940000	REPROD-PERIOD	Fflr 9-3	PF04185 GO:0016788 AT3G03530.1 NPC4 non-specific phospholipase C4
243	Glyma16g09020	**8471000:12411000**	3940000	REPROD-PERIOD	Fflr 9-3	PF00044,PF02800 PTHR10836 KOG0657 1.2.1.12 K00134 GO:0016620,GO:0055114 AT1G16300.1 GAPCP-2 glyceraldehyde-3-phosphate dehydrogenase of plastid 2
244	Glyma18g07345	**8471000:12411000**	3940000	REPROD-PERIOD	Fflr 9-3	PTHR11132 AT1G07290.1 GONST2 golgi nucleotide sugar transporter 2
245	Glyma18g14670	**13545720:19024290**	5478570	REPROD-PERIOD	Reprod 5-2	PF00271,PF00270 PTHR24031,PTHR24031:SF161 KOG0331 GO:0003676,GO:0005524,GO:0008026,GO:0004386 AT3G22330.1 ATRH53,PMH2 putative mitochondrial RNA helicase 2
246	Glyma18g14680	**13545720:19024290**	5478570	REPROD-PERIOD	Reprod 5-2	PF00069,PF00560,PF08263 PTHR24420:SF516,PTHR24420 KOG1187 GO:0004672,GO:0005524,GO:0006468,GO:0005515 AT4G20270.1 BAM3 Leucine-rich receptor-like protein kinase family protein
247	Glyma18g14730	**13545720:19024290**	5478570	REPROD-PERIOD	Reprod 5-2	
248	Glyma18g14740	**13545720:19024290**	5478570	REPROD-PERIOD	Reprod 5-2	PF00266 PTHR21152,PTHR21152:SF7 KOG2862 2.6.1.44,2.6.1.45,2.6.1.51 K00830 GO:0008152 AT2G13360.1 AGT,AGT1,SGAT alanine:glyoxylate aminotransferase
249	Glyma18g14750	**13545720:19024290**	5478570	REPROD-PERIOD	Reprod 5-2	PF00170 PTHR13301,PTHR13301:SF21 GO:0003700,GO:0043565,GO:0046983,GO:0006355 AT5G11260.1 HY5,TED 5 Basic-leucine zipper (bZIP) transcription factor family protein
250	Glyma18g14760	**13545720:19024290**	5478570	REPROD-PERIOD	Reprod 5-2	
251	Glyma18g14770	**13545720:19024290**	5478570	REPROD-PERIOD	Reprod 5-2	PF02854,PF02847 PTHR18034,PTHR18034:SF3 KOG2140 K13100 GO:0005515,GO:0016070 AT1G80930.1 MIF4G domain-containing protein / MA3 domain-containing protein
252	Glyma18g14790	**13545720:19024290**	5478570	REPROD-PERIOD	Reprod 5-2	
253	Glyma18g14810	**13545720:19024290**	5478570	REPROD-PERIOD	Reprod 5-2	PF01582,PF07725,PF00931 PTHR11017,PTHR11017:SF20 KOG4658 GO:0043531,GO:0005515,GO:0007165,GO:0005622,GO:0045087,GO:0031224,GO:0004888,GO:0006915,GO:0005524 AT5G17680.1 disease resistance protein (TIR-NBS-LRR class), putative
254	Glyma19g04890	**0:21124310**	21124310	YIELD	Pod num 1-9	PF00657 PTHR22835:SF39,PTHR22835 GO:0016788,GO:0006629 AT5G15720.1 GLIP7 GDSL-motif lipase 7
255	Glyma19g04900	**0:21124310**	21124310	YIELD	Pod num 1-9	PF00326 PTHR12277,PTHR12277:SF8 KOG1552 GO:0008236,GO:0006508 AT3G30380.2 alpha/beta-Hydrolases superfamily protein
256	Glyma19g04910	**0:21124310**	21124310	YIELD	Pod num 1-9	PF06391 PTHR12683,PTHR12683:SF3 K10842 GO:0007049,GO:0005634 AT4G30820.1 cyclin-dependent kinase-activating kinase assembly factor-related / CDK-activating kinase
257	Glyma19g04920	**0:21124310**	21124310	YIELD	Pod num 1-9	PF10046 PTHR10668:SF1,PTHR10668 KOG4559 AT5G49550.1
258	Glyma19g04990	**0:21124310**	21124310	YIELD	Pod num 1-9	PF01490 PTHR22950 KOG1305 AT5G38820.1 Transmembrane amino acid transporter family protein

259	Glyma19g05000	**0:21124310**	21124310	YIELD	Pod num 1-9	PF01490 PTHR22950 KOG1305 AT5G38820.1 Transmembrane amino acid transporter family protein
260	Glyma19g05020	**0:21124310**	21124310	YIELD	Pod num 1-9	PF01747 PTHR24799:SF2,PTHR24799 KOG0636 GO:0004781,GO:0000103 AT3G22890.1 APS1 ATP sulfurylase 1
261	Glyma19g05030	**0:21124310**	21124310	YIELD	Pod num 1-9	PF00261 AT5G11390.1 WIT1 WPP domain-interacting protein 1
262	Glyma19g05040	**0:21124310**	21124310	YIELD	Pod num 1-9	PTHR22763 AT3G02340.1 RING/U-box superfamily protein
263	Glyma19g05050	**0:21124310**	21124310	YIELD	Pod num 1-9	PF00170 PTHR22952:SF60,PTHR22952 GO:0003700,GO:0043565,GO:0046983,GO:0006355 AT3G30530.1 ATBZIP42,bZIP42 basic leucine-zipper 42
264	Glyma19g05060	**0:21124310**	21124310	YIELD	Pod num 1-9	PF01501 PTHR13778:SF3,PTHR13778 2.4.1.43 K13648 GO:0016757 AT3G02350.1 GAUT9 galacturonosyltransferase 9
265	Glyma19g05080	**0:21124310**	21124310	YIELD	Pod num 1-9	PF00249 PTHR10641 KOG0048 GO:0003677 AT3G08500.1 AtMYB83,MYB83 myb domain protein 83
266	Glyma19g05120	**0:21124310**	21124310	YIELD	Pod num 1-9	PF00393,PF03446 PTHR11811 KOG2653 1.1.1.44 K00033 GO:0004616,GO:0050661,GO:0006098,GO:0055114 AT3G02360.1 6-phosphogluconate dehydrogenase family protein
267	Glyma19g05130	**0:21124310**	21124310	YIELD	Pod num 1-9	PTHR11926:SF15,PTHR11926 GO:0016758,GO:0008152 AT1G78270.1 AtUGT85A4,UGT85A4 UDP-glucosyl transferase 85A4
268	Glyma19g05140	**0:21124310**	21124310	YIELD	Pod num 1-9	PF00689,PF00702,PF00122,PF00690 PTHR24093,PTHR24093:SF73 KOG0204 3.6.3.8 K01537 GO:0000166,GO:0046872,GO:0003824,GO:0008152,GO:0016020,GO:0015662,GO:0006812,GO:0006754 AT3G22910.1 ATPase E1-E2 type family protein /
269	Glyma19g05150	**0:21124310**	21124310	YIELD	Pod num 1-9	AT5G57685.1 ATGDU3,GDU3,LSB1 glutamine dumper 3
270	Glyma19g05170	**0:21124310**	21124310	YIELD	Pod num 1-9	PF00643,PF06203 KOG1601 GO:0008270,GO:0005622,GO:0005515 AT5G15840.1 CO,FG B-box type zinc finger protein with CCT domain

Supplementary Table 11. Candidate genes associated with maturity and growth habit in soybean.

Category	Gene ID	Name	Reference
**Maturity**	Glyma06g23040	***E1***	Watanabe et al. 2009; Tsubokura et al 2013; Langewisch et al 2014
	Glyma10g36600	***E2***	
	Glyma19g41210	***E3***	
	Glyma20g22160	***E4***	
Growth type (Determinate and Indeterminate)	Glyma19g37890	*DT1*	Tian et al 2010
	Glyma03g35250	*DT1-A/TFL1*	
	Glyma10g08340	*DT2*	
	Glyma13g22030	*DT2-A*	

Supplementary Table 12. **Allelic variation for maturity genes in soybean**. (a) Allelic variation for maturity and determinate/indeterminate growth loci. (b) Details of major maturity genes used for haplogroup analysis.

(a)

Seq Id	MG	Region/ Country	Growth	Type	*E1, e1-as, e1-fs, e1-nl*	*E2, e2*	*E3, e3, E3-Short*	*E4, e4-kes, e4-SORE1,*	*DT1, dt1-1(R62S), dt1-2 (P113L), dt1-3 (R130K), dt1-4 (R166W)*	*DT2, dt2*
HN105	0	Sweden	Determinate	Elite	*e1-nl*	*e2/E2**	*e3*	*E4*	*dt1-4*	*dt2*
HN037	I	Japan	Determinate	Elite	*E1*	*e2/E2**	*e3*	*E4*	*dt1-4*	*DT2*
HN011	I	Japan	Determinate	Land Race	*e1-as*	*E2*	*e3*	*E4*	*dt1-1*	*DT2*
HN010	II	China	Non-determinate	Land Race	*E1*	*E2*	*E3*	*E4*	*DT-1*	*DT2*
HN060	II	China	Non-determinate	Land Race	*E1*	*E2*	*E3*	*E4*	*DT-1*	*DT2*
HN062	II	China	Semi-determinate	Elite	*E1*	*E2*	*E3*	*E4*	*DT-1*	*DT2*
HN063	II	China	Non-determinate	Wild	*E1*	*E2**	*E3*	*E4**	*DT-1*	*DT2*
HN056	II	China	Non-determinate	Elite	*E1*	*e2/E2**	*E3*	*E4*	*DT-1*	*dt2*
HN054	II	Russian Federation	Non-determinate	Elite	*e1-as*	*E2**	*E3*	*E4*	*DT-1*	*DT2*
HN064	II	Former Serbia	Non-determinate	Elite	*e1-as*	*e2/E2**	*E3*	*E4*	*DT-1*	*dt2*
HN070	II	U.S.	Non-determinate	Elite	*e1-as*	*e2/E2**	*E3*	*E4*	*DT-1*	*dt2*
HN057	II	China	Non-determinate	Elite	*e1-as*	*E2*	*e3*	*E4*	*DT-1*	*DT2*
HN106	II	Japan	Non-determinate	Land Race	*E1*	*E2*	*E3*	*E4*	*DT-1*	*DT2*
HN005	III	China	Non-determinate	Land Race	*E1*	*E2*	*E3*	*E4*	*dt1-1*	*dt2*
HN008	III	China	Non-determinate	Land Race	*E1*	*E2*	*E3*	*E4*	*DT-1*	*dt2*
HN015	III	China	Non-determinate	Land Race	*E1*	*E2*	*E3*	*E4*	*DT-1*	*dt2*
HN018	III	China	Semi-determinate	Land Race	*E1*	*E2*	*E3*	*E4*	*dt1-1*	*dt2*
HN020	III	China	Non-determinate	Land Race	*E1*	*E2*	*E3*	*E4*	*DT-1*	*dt2*
HN024	III	China	Non-determinate	Land Race	*E1*	*E2*	*E3*	*E4*	*DT-1*	*dt2*
HN026	III	North Korea	Non-determinate	Land Race	*E1*	*E2*	*E3*	*E4*	*DT-1*	*dt2*
HN068	III	North Korea	Non-determinate	Land Race	*E1*	*E2*	*E3*	*E4*	*DT-1*	*DT2*
HN072	III	China	Non-determinate	Land Race	*E1*	*E2*	*E3*	*E4*	*DT-1*	*DT2*
HN081	III	China	Non-determinate	Elite	*E1*	*E2**	*E3*	*E4*	*DT-1*	*dt2*
HN097	III	China	Determinate	Land Race	*E1*	*e2**	*e3*	*E4*	*dt1-1*	*DT2*
HN051	III	South Korea	Non-determinate	Land Race	*E1*	*E2**	*e3*	*E4*	*DT-1*	*DT2*
HN034	III	Japan	Non-determinate	Land Race	*E1*	*E2*	*E3-Short*	*E4*	*DT-1*	*DT2*
HN031	III	China	Non-determinate	Land Race	*E1*	*E2**	*E3-Short*	*E4*	*dt1-4*	*DT2*
HN059	III	China	Determinate	Elite	*E1*	*e2/E2**	*E3-Short*	*E4*	*dt1-4*	*dt2*
HN067	III	China	Non-determinate	Land Race	*E1*	*e2/E2**	*E3-Short*	*E4*	*DT-1*	*DT2*
HN079	III	China	Non-determinate	Land Race	*E1*	*e2/E2**	*E3-Short*	*E4*	*DT-1*	*DT2*
HN084	III	China	Semi-determinate	Elite	*E1*	*e2/E2**	*E3-Short*	*E4*	*DT-1*	*DT2*
HN086	III	China	Semi-determinate	Elite	*E1*	*e2/E2**	*E3-Short*	*E4*	*dt1-1*	*DT2*
HN107	III	Japan	Determinate	Elite	*E1*	*e2/E2**	*E3-Short*	*E4*	*dt1-4*	*dt2*

HN029	III	U.S.	N/A	Elite	*e1-as*	*E2*	*E3*	*E4*	*DT-1*	*DT2*
HN030	III	U.S.	Non-determinate	Elite	*e1-as*	*E2*	*E3*	*E4*	*DT-1*	*DT2*
HN033	III	North Korea	Non-determinate	Elite	*e1-as*	*e2*	*E3*	*E4*	*DT-1*	*DT2*
HN065	III	U.S.	Non-determinate	Elite	*e1-as*	*E2*	*E3*	*E4*	*DT-1*	*DT2*
HN066	III	U.S.	Non-determinate	Elite	*e1-as*	*E2*	*E3*	*E4*	*DT-1*	*DT2*
HN073	III	U.S.	Non-determinate	Elite	*e1-as*	*E2*	*E3*	*E4*	*DT-1*	*dt2*
HN087	III	U.S.	Non-determinate	Elite	*e1-as*	*E2*	*E3*	*E4*	*DT-1*	*DT2*
HN088	III	N/A	Non-determinate	Elite	*e1-as*	*E2*	*E3*	*E4*	*DT-1*	*DT2*
HN092	III	U.S.	Non-determinate	Elite	*e1-as*	*E2*	*E3*	*E4*	*DT-1*	*DT2*
HN032	III	Japan	Non-determinate	Land Race	*e1-as*	*E2**	*E3*	*E4*	*DT-1*	*DT2*
HN038	IV	Japan	Determinate	Elite	*E1*	*e2/E2**	*e3*	*E4*	*dt1-4*	*DT2*
HN002	IV	China	Determinate	Land Race	*E1*	*E2*	*E3*	*E4*	*dt1-1*	*dt2*
HN003	IV	China	Non-determinate	Land Race	*E1*	*E2*	*E3*	*E4*	*dt1-1*	*DT2*
HN004	IV	China	Non-determinate	Land Race	*E1*	*E2*	*E3*	*E4*	*DT-1*	*dt2*
HN017	IV	China	Semi-determinate	Land Race	*E1*	*E2*	*E3*	*E4*	*dt1-1*	*dt2*
HN019	IV	China	Determinate	Land Race	*E1*	*E2*	*E3*	*E4*	*dt1-1*	*DT2*
HN021	IV	Japan	Non-determinate	Land Race	*E1*	*E2*	*E3*	*E4*	*DT-1*	*dt2*
HN047	IV	China	Non-determinate	Land Race	*E1*	*E2*	*E3*	*E4*	*DT-1*	*dt2*
HN055	IV	China	Semi-determinate	Land Race	*E1*	*E2*	*E3*	*E4*	*dt1-1*	*dt2*
HN069	IV	China	Non-determinate	Land Race	*E1*	*E2*	*E3*	*E4*	*DT-1*	*dt2*
HN012	IV	China	Non-determinate	Land Race	*E1*	*E2**	*E3*	*E4*	*DT-1*	*dt2*
HN016	IV	China	Non-determinate	Land Race	*E1*	*E2**	*E3*	*E4*	*DT-1*	*DT2*
HN022	IV	China	Non-determinate	Land Race	*E1*	*E2**	*E3*	*E4*	*DT-1*	*dt2*
HN025	IV	China	Non-determinate	Land Race	*E1*	*E2**	*E3*	*E4*	*DT-1*	*dt2*
HN039	IV	Japan	N/A	Wild	*E1*	*E2**	*E3*	*E4**	*DT-1*	*DT2*
HN040	IV	Japan	N/A	Wild	*E1*	*E2**	*E3*	*E4**	*DT-1*	*dt2*
HN045	IV	South Korea	N/A	Wild	*E1*	*E2**	*E3*	*E4**	*DT-1*	*DT2*
HN046	IV	South Korea	N/A	Wild	*E1*	*E2**	*E3*	*E4**	*DT-1*	*dt2*
HN052	IV	South Korea	N/A	Wild	*E1*	*E2**	*E3*	*E4**	*DT-1*	*DT2*
HN053	IV	South Korea	N/A	Wild	*E1*	*E2**	*E3*	*E4**	*DT-1*	*dt2*
HN058	IV	China	Semi-determinate	Land Race	*E1*	*E2**	*E3*	*E4*	*dt1-1, dt1-4*	*DT2*
HN076	IV	China	Non-determinate	Land Race	*E1*	*E2**	*E3*	*E4*	*DT-1*	*dt2*
HN007	IV	South Korea	Non-determinate	Land Race	*E1*	*E2**	*e3*	*E4*	*DT-1*	*dt2*
HN023	IV	South Korea	Non-determinate	Land Race	*E1*	*E2**	*e3*	*E4*	*DT-1*	*DT2*
HN094	IV	North Korea	Determinate	Elite	*E1*	*e2**	*e3*	*E4*	*dt1-4*	*DT2*
HN095	IV	North Korea	Determinate	Elite	*E1*	*e2**	*e3*	*E4*	*dt1-4*	*DT2*
HN006	IV	South Korea	Determinate	Elite	*E1*	*e2/E2**	*e3*	*E4*	*dt1-4*	*DT2*
HN096	IV	North Korea	Non-determinate	Elite	*E1*	*E2*	*e3*	*E4*	*DT-1*	*DT2*
HN009	IV	China	Non-determinate	Land Race	*E1*	*E2**	*E3-Short*	*E4*	*DT-1*	*DT2*
HN049	IV	South Korea	Determinate	Elite	*E1*	*e2/E2**	*E3-Short*	*E4*	*dt1-4*	*DT2*
HN078	IV	China	Non-determinate	Land Race	*E1*	*e2/E2**	*E3-Short*	*E4*	*DT-1*	*dt2*
HN082	IV	China	Non-determinate	Elite	*E1*	*e2/E2**	*E3-Short*	*E4*	*DT-1*	*dt2*
HN083	IV	China	Semi-determinate	Elite	*E1*	*e2/E2**	*E3-Short*	*E4*	*DT-1*	*DT2*
HN085	IV	China	Non-determinate	Elite	*E1*	*e2/E2**	*E3-Short*	*E4*	*DT-1*	*DT2*

HN091	IV	China	Semi-determinate	Elite	*E1*	*e2/E2**	*E3-Short*	*E4*	*DT-1*	*DT2*
HN099	IV	U.S.	Non-determinate	Elite	*e1-as*	*E2*	*E3*	*E4*	*DT-1*	*DT2*
HN102	IV	U.S.	Non-determinate	Elite	*e1-as*	*E2*	*E3*	*E4*	*DT-1*	*DT2*
HN089	IV, V	South Korea	Determinate	Elite	*E1*	*e2/E2**	*E3-Short*	*E4*	*dt1-4*	*DT2*
HN013	IV,V	U.S.	Determinate	Elite	*E1*	*E2*	*E3*	*E4*	*dt1-4*	*DT2*
HN027	N/A	U.S.	Non-determinate	Elite	*e1-as*	*E2*	*E3*	*E4*	*DT-1*	*DT2*
HN035	V	South Korea	Determinate	Elite	*E1*	*E2*	*e3*	*E4*	*dt1-4*	*dt2*
HN041	V	South Korea	Determinate	Elite	*E1*	*E2**	*e3*	*E4*	*dt1-4*	*dt2*
HN042	V	South Korea	Determinate	Elite	*E1*	*E2**	*e3*	*E4*	*dt1-4*	*DT2*
HN043	V	South Korea	Semi-determinate	Elite	*E1*	*E2**	*e3*	*E4*	*dt1-4*	*DT2*
HN044	V	South Korea	Semi-determinate	Elite	*E1*	*E2**	*e3*	*E4*	*dt1-4*	*DT2*
HN028	V	U.S.	Determinate	Elite	*E1*	*E2*	*E3*	*E4*	*dt1-4*	*dt2*
HN001	V	U.S.	Determinate	Elite	*E1*	*E2**	*E3*	*E4*	*dt1-4*	*DT2*
HN061	V	Nepal	Non-determinate	Elite	*E1*	*E2**	*E3*	*E4*	*dt1-4*	*dt2*
HN075	V	China	Non-determinate	Land Race	*E1*	*E2**	*E3*	*E4*	*DT-1*	*dt2*
HN077	V	China	Non-determinate	Land Race	*E1*	*E2**	*E3*	*E4*	*DT-1*	*dt2*
HN074	V	China	Non-determinate	Elite	*E1*	*e2/E2**	*E3*	*E4*	*DT-1*	*DT2*
HN080	V	China	Semi-determinate	Land Race	*E1*	*e2/E2**	*E3*	*E4*	*dt1-1*	*dt2*
HN048	V	South Korea	Non-determinate	Elite	*E1*	*E2**	*e3*	*E4*	*DT-1*	*dt2*
HN093	V	North Korea	Determinate	Elite	*E1*	*E2**	*e3*	*E4*	*dt1-4*	*DT2*
HN014	V	U.S.	Determinate	Elite	*E1*	*E2*	*E3-Short*	*E4*	*dt1-4*	*DT2*
HN098	V	Vietnam	Non-determinate	Elite	*E1*	*E2**	*E3-Short*	*E4*	*DT-1*	*DT2*
HN104	VI	N/A	Non-determinate	Land Race	*E1*	*E2**	*E3*	*E4*	*DT-1*	*DT2*
HN103	VI	U.S.	N/A	Elite	*E1*	*E2**	*e3*	*E4*	*dt1-4*	*DT2*
HN050	VI	Japan	Determinate	Elite	*E1*	*e2/E2**	*E3-Short*	*E4*	*dt1-4*	*DT2*
HN071	VII	U.S.	Determinate	Elite	*E1*	*E2**	*E3*	*E4*	*dt1-4*	*DT2*
HN090	VII	China	Determinate	Elite	*E1*	*E2**	*E3-Short*	*E4*	*dt1-4*	*DT2*
HN101	VII	N/A	Non-determinate	Elite	*e1-as*	*E2*	*E3*	*E4*	*DT-1*	*DT2*
HN100	VIII	U.S.	Determinate	Elite	*E1*	*E2*	*E3*	*E4*	*dt1-4*	*DT2*

(b)

Gene	Glyma	Chromosome coordinates	Known Allele	Polymorphism type/ Position	Amino acid change	Found in current study
E1	Glyma06g23040	20006928 -20007814	E1	SNP (Gm06:20007173)	R15T	Yes
			e1-as	-	-	Yes
			e1-fs	Indel: A (Gm06:20007178)	Frameshift	No
			e1-nl	Gene deletion	Null Protein	Yes
E2	Glyma10g36600	44716804–44738165	e2	SNP (Gm10:44732850)	K521*	Yes
			E2*	SNP (Gm10:44727337)	I220V	Yes
E3	Glyma19g41210	47512505–47519937	E3 (Short)		No change	Yes

				2.6 Kb deletion in intron 3 (Gm19: 47516697-47519330)		
			e3	15.5 Kb deletion (Gm19: 47531605-47516603)	Frameshift	Yes
			e3-ft3	SNP (Gm19: 47516339)	G1050R	No
E4	Glyma20g22160	32087661–32093266	E4*	SNP (Gm20: 32088844)	L151S	Yes
			e4 (SORE-1)	Retrotransposome insertion (Gm20: 32089086)	Frameshift	No
			e4-oto	Indel (Gm20: 32089731)	Frameshift	No
			e4-tsu	Indel (Gm20: 32091045)	Frameshift	No
			e4-kam	Indel Gm20: 32091473	Frameshift	No
			e4-kes	Indel (Gm20: 32091662)	Frameshift	No
DT1	Glyma19g37890	44979743–44981385	dt1-1	SNP (Gm19: 44981190)	R62S	Yes
			dt1-2	SNP (Gm19: 44980245)	P113L	No
			dt1-3	SNP (Gm19: 44980194)	R130K	No
			dt1-4	SNP (Gm19: 44980087)	R166W	Yes
DT2	Glyma10g08340	7175357 - 7177049	dt2	SNP (Gm10: 7176981)	T06K	Yes

1. Locus names were used or modified from published data (Langewisch et al 2014)
2. E2* Non_Syn_SNP I220V for E2 locus is not reported previously
3. E4* SNP <L151S> for E4 locus is not reported previously

Supplementary Table 13. Total oil and protein content in 106 soybean lines

Type	Germplasm identifier	Total oil (%)	Total protein (%)
Wild	HN031	12.56	38.55
	HN032	20.51	41.26
	HN039	10.88	39.80
	HN040	10.44	49.18
	HN045	9.84	45.85
	HN046	5.76	42.14
	HN051	14.05	43.51
	HN052	10.56	37.43
	HN053	12.16	45.02
	HN063	19.93	41.11
Elite	HN001	21.88	36.56
	HN006	17.1	39.68
	HN013	21.11	39.43
	HN014	21.14	39.41
	HN027	21.28	40.80
	HN028	20.77	42.32
	HN029	22.26	39.05
	HN030	21.32	41.71
	HN033	18.99	42.07
	HN035	16.09	48.75
	HN037	18.54	39.03
	HN041	12.43	47.86
	HN042	10.65	44.12
	HN043	17	45.23
	HN044	14.32	44.26
	HN048	20.02	47.82
	HN049	15.78	38.79
	HN050	22.99	37.14
	HN054	21.8	41.25
	HN056	18.06	40.51
	HN057	21.04	44.15
	HN059	22.76	46.24
	HN061	22.02	40.97
	HN062	21.09	41.36
	HN064	22.88	41.20
	HN065	20.37	43.94
	HN066	21.19	41.19
	HN070	24.21	40.88
	HN071	19.01	43.06

	HN073	23.64	41.79
	HN074	18.69	42.06
	HN083	19.93	40.01
	HN084	18.79	43.29
	HN085	21.71	40.47
	HN086	19.69	44.33
	HN087	20.45	42.08
	HN088	22.37	40.70
	HN089	17.46	40.11
	HN090	14.24	42.24
	HN091	17.98	38.68
	HN092	22.4	41.28
	HN094	17.99	46.50
	HN095	17.46	42.41
	HN096	20	44.46
	HN098	15.56	47.70
	HN099	21.02	39.74
	HN100	19.5	42.64
	HN101	20.98	38.63
	HN102	21.23	42.30
	HN103	20.84	41.60
	HN105	18.29	46.13
	HN107	18.52	39.17
Land Race	HN002	17.99	41.05
	HN003	15.33	42.60
	HN004	17.12	39.65
	HN005	18.76	40.50
	HN007	20.06	43.37
	HN008	17.37	38.81
	HN009	20.29	39.60
	HN010	18.42	40.17
	HN011	19.3	41.46
	HN012	16.05	30.39
	HN015	17.65	39.19
	HN016	15.6	39.20
	HN017	18.76	38.89
	HN018	17.12	39.27
	HN020	18.32	40.57
	HN021	17.52	39.21
	HN022	16.17	41.52
	HN023	18.26	44.54
	HN024	18.85	40.91
	HN025	15.5	38.16
	HN026	20.26	42.92
	HN034	11.88	37.90
	HN047	16.79	48.23

	HN055	19.88	40.76
	HN058	19.04	39.71
	HN060	14.69	39.81
	HN067	20.76	41.02
	HN068	19.38	39.17
	HN069	18.51	40.37
	HN072	23.59	41.72
	HN075	16.03	41.52
	HN076	14.23	37.33
	HN077	19.68	40.77
	HN078	20.37	40.63
	HN079	20.52	47.13
	HN080	17.25	37.49
	HN097	16.53	47.16
	HN104	20.11	43.57
	HN106	18.56	42.21

Supplementary Table 14. Copy number variation on genes associated with oil traits in soybean

	HN070 VS HN046			HN070 VS HN063			HN046 VS HN063		
	HN070	HN046	Gene number	HN070	HN063	Gene number	HN046	HN063	Gene number
Copy number increase in individual 1	+	-	33	+	-	39	+	-	49
	+	=	260	+	=	296	+	=	352
	=	-	1372	=	-	1008	=	-	823
Summary			1665			1343			1224
Copy number increas in individual 2	-	+	19	-	+	17	-	+	17
	-	=	485	-	=	467	-	=	1193
	=	+	322	=	+	292	=	+	310
Summary			826			776			1520
Total			2491			2119			2744

+ for more copy number compared with the reference, - for less copy number, = for no variation. HN070: highest oil, HN046: lowest oil, HN063: wild soybean

Supplementary Table 15. Copy number variations in total protein trait related genes

	HN040 VS HN012			HN040 VS HN063			HN012 VS HN063		
	HN040	HN012	Gene number	HN040	HN063	Gene number	HN012	HN063	Gene number
Copy number increase in individual 1	+	-	24	+	-	20	+	-	20
	+	=	262	+	=	296	+	=	343
	=	-	1408	=	-	889	=	-	847
Summary			1694			1205			1210
Copy number increase in individual 2	-	+	32	-	+	26	-	+	18
	-	=	987	-	=	1006	-	=	1385
	=	+	310	=	+	289	=	+	288
Summary			1329			1321			1691
Total			3023			2526			2901

+ for more copy number compared with the reference, - for less copy number, = for no variation. HN040: highest protein HN012: lowest protein HN063: wild

Supplementary Figure 1. Missing rate of SNPs for 106 soybean germplasm sequences. Proportion of 106 individuals with missing genotype at each SNP loci was calculated. Distribution of the missing genotype proportions was shown in this figure. Majority of the SNPs had low missing rate.

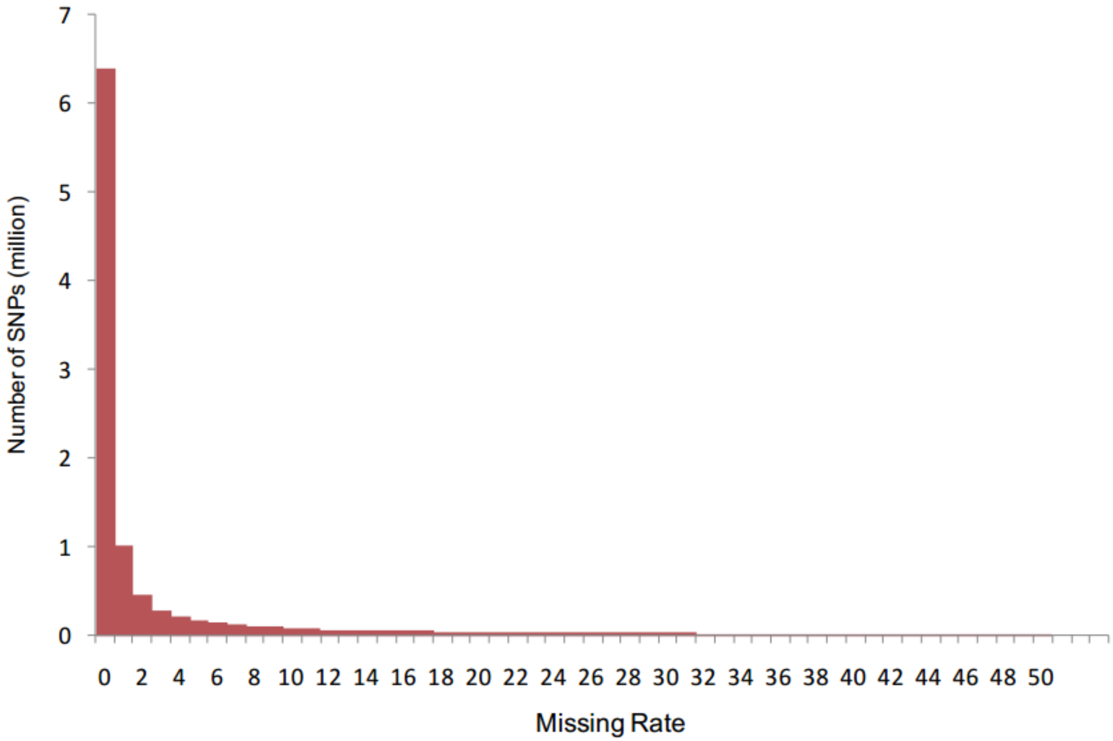

Supplementary Figure 2. Distribution rate of indels. Distribution rate of small insertion and deletions (indels) in different genomic regions. All identified indels are shorter than or equal to 5 bp.

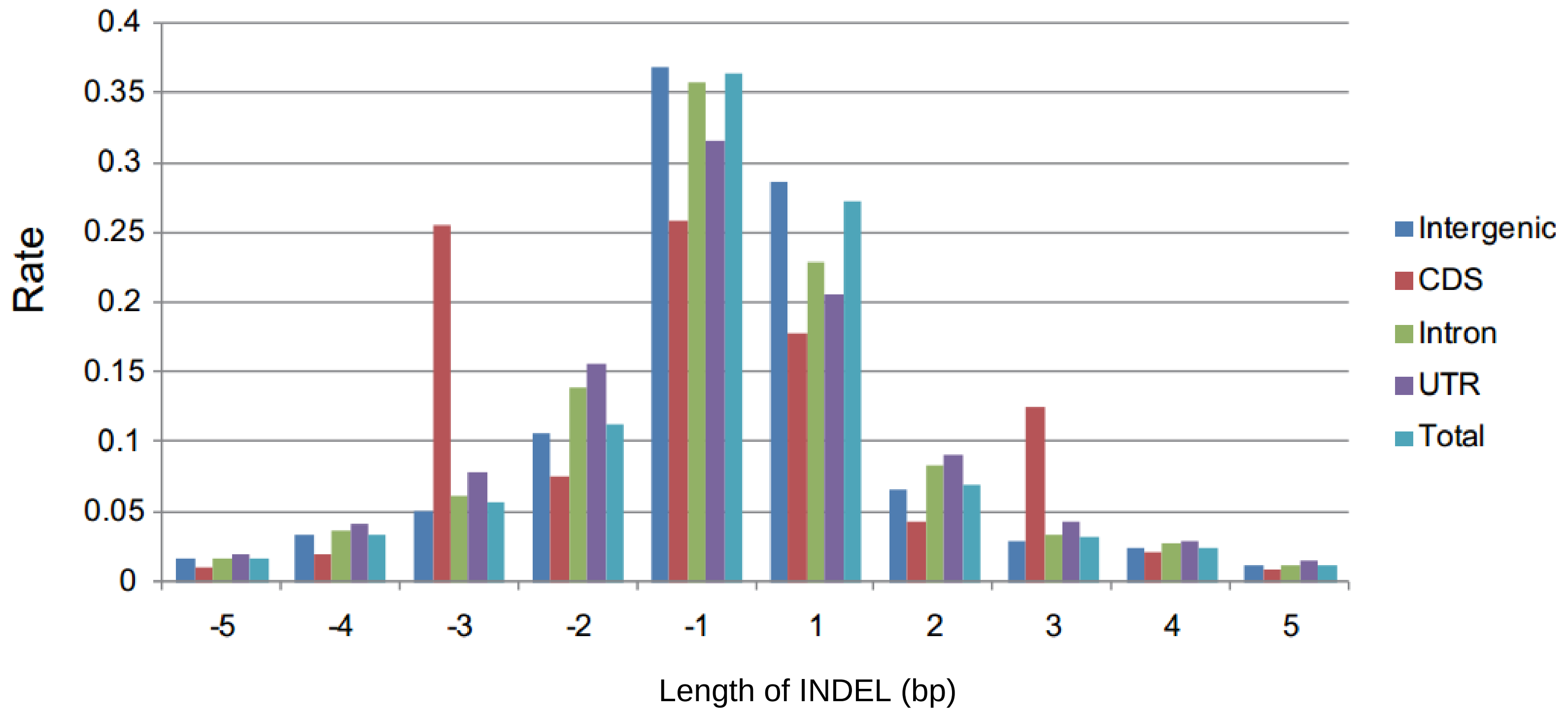

Supplementary Figure 3. Gene ontology (GO) analysis of large effect SNPs genes within biological process, cellular component and molecular function. a. Gene ontology (GO) of each Large effect SNPs genes were obtained by the annotation of gene functions using GO database. The blue bar is the ratio of certain category of genes number in the whole genome to the whole category gene numbers. **b.** GO enrichment analysis of large effect SNPs genes were calculated by Cytoscape 2.8 using the plugins BiNGO-2.44.

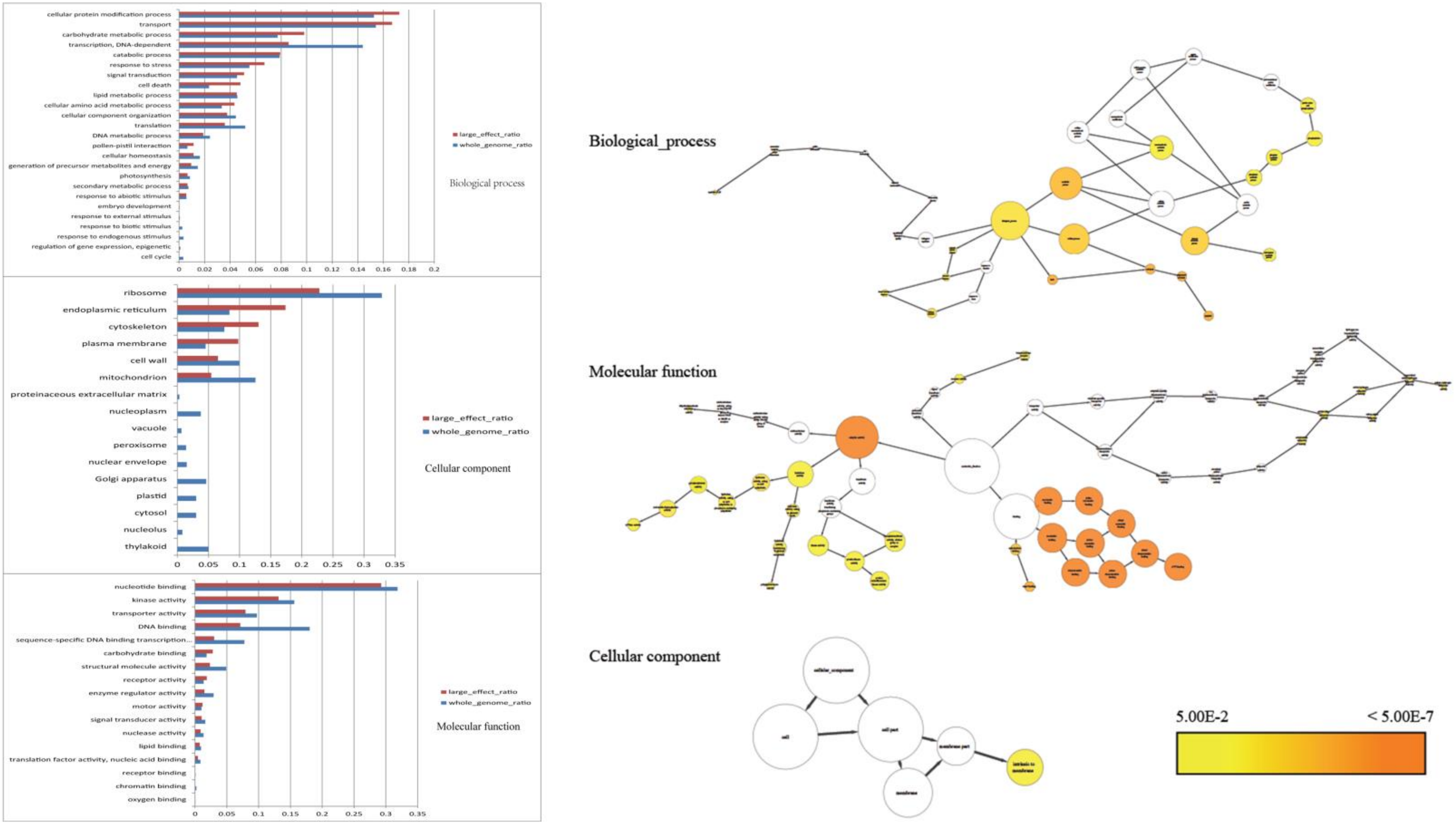

Supplementary Figure 4. Gene ontology (GO) analysis of Present/Absent Variation (PAV) genes within biological process, cellular component and molecular function. Gene ontology (GO) of each PAV genes were obtained by the annotation of gene functions using GO database.

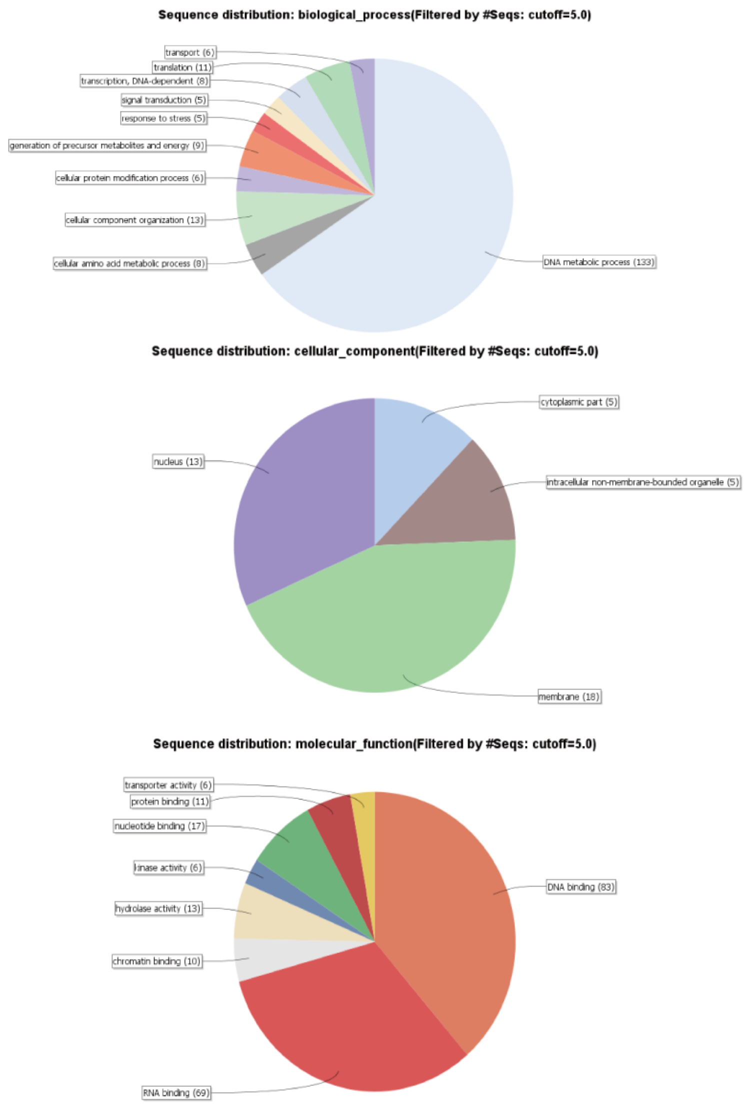

Supplementary Figure 5. Bayesian clustering analysis of all individual (STRUCTURE, K=3; K=4; K=5; and K=6).

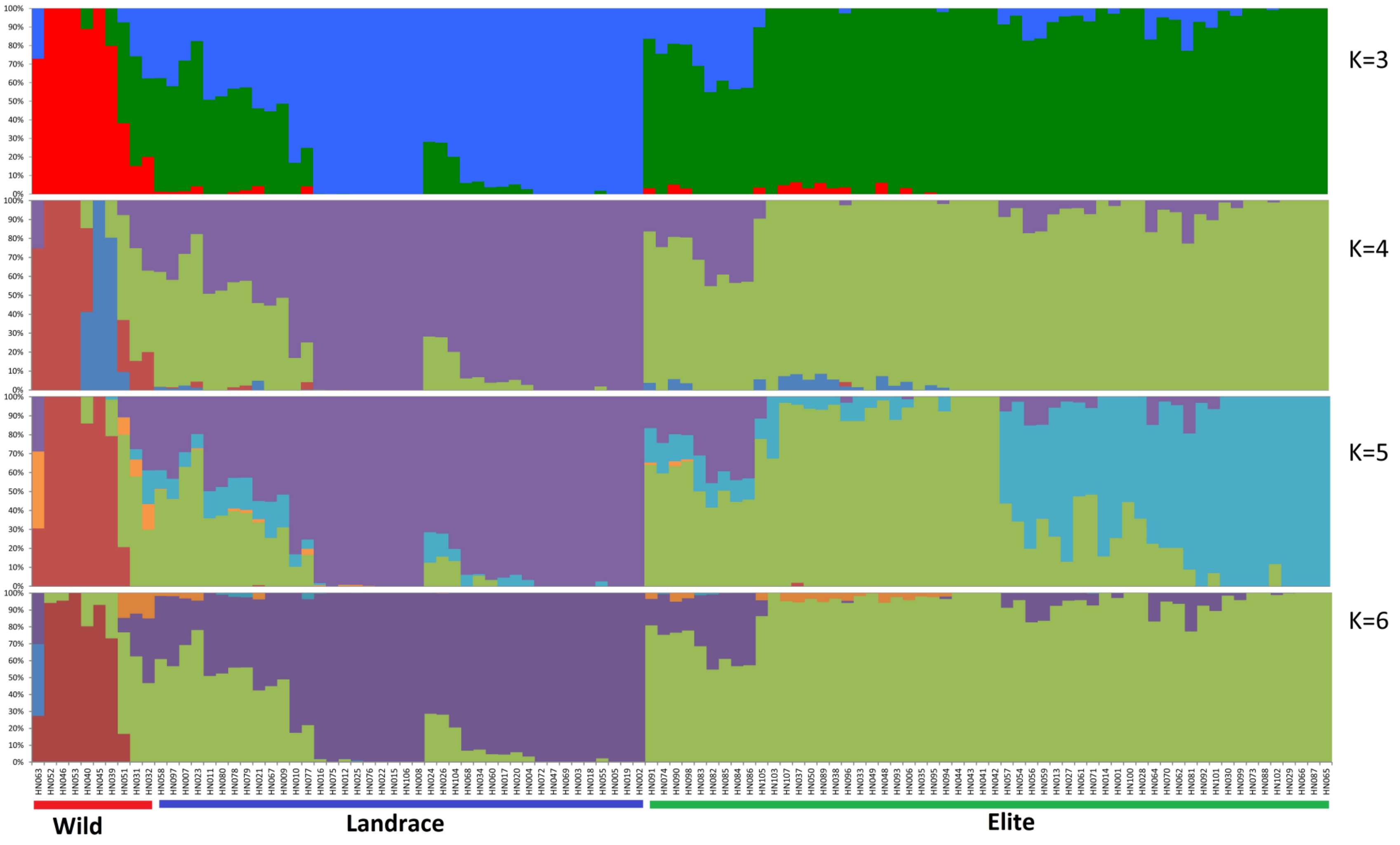

Each line is represented by a vertical bar, and the length of each colored segment in each vertical bar represents the proportion contributed by the population.

Supplementary Figure 6. Overlap of (a) Total SNPs and (b) non-synonymous SNPs in the three soybean gene pools (wild, landrace and elite cultivar).

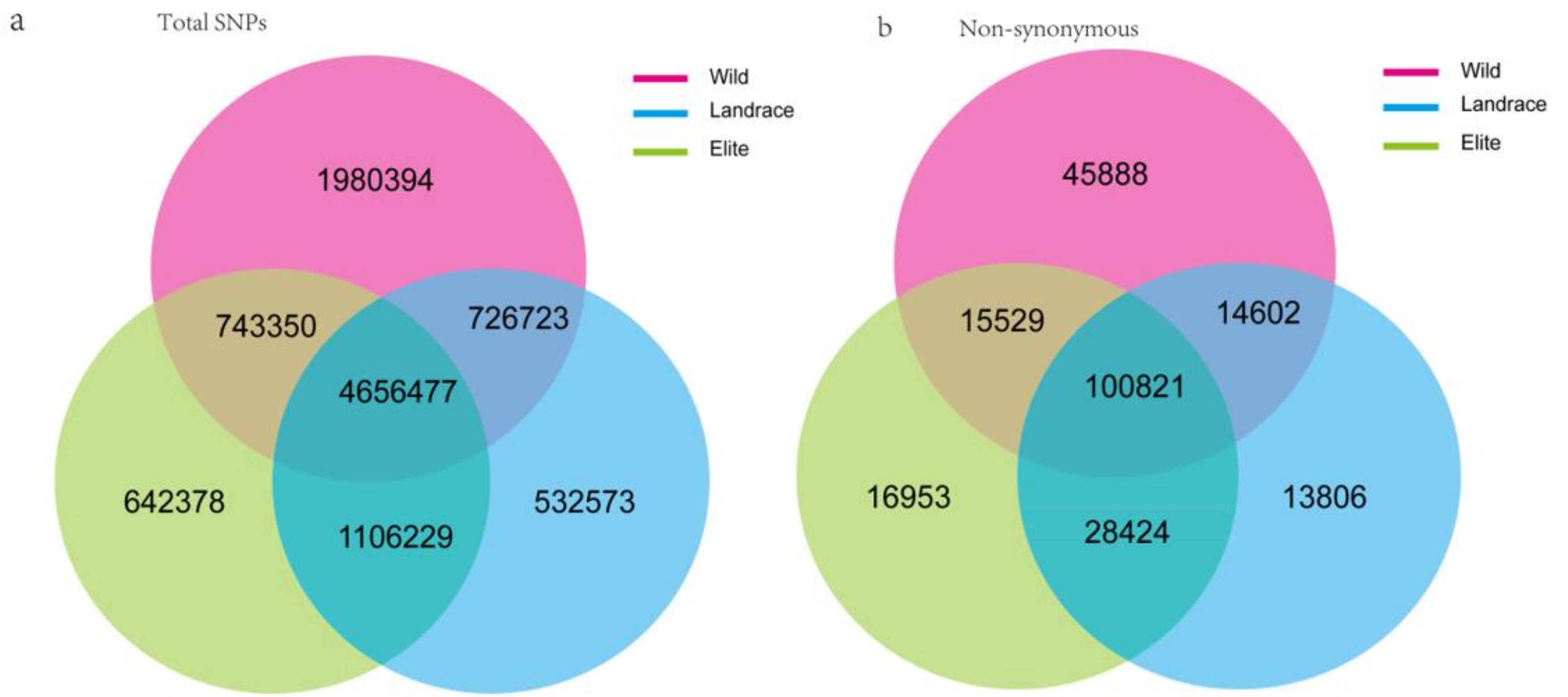

Supplementary Figure 7. Comparison of the diversity (θπ) levels across genic and non-genic regions. Coding regions displayed lower diversity levels relative to intron and UTR regions. The genic regions include both coding and non-coding regions of the genes (mRNA, CDS, UTR and intron), in comparison to the genic and non-genic regions genome-wide.

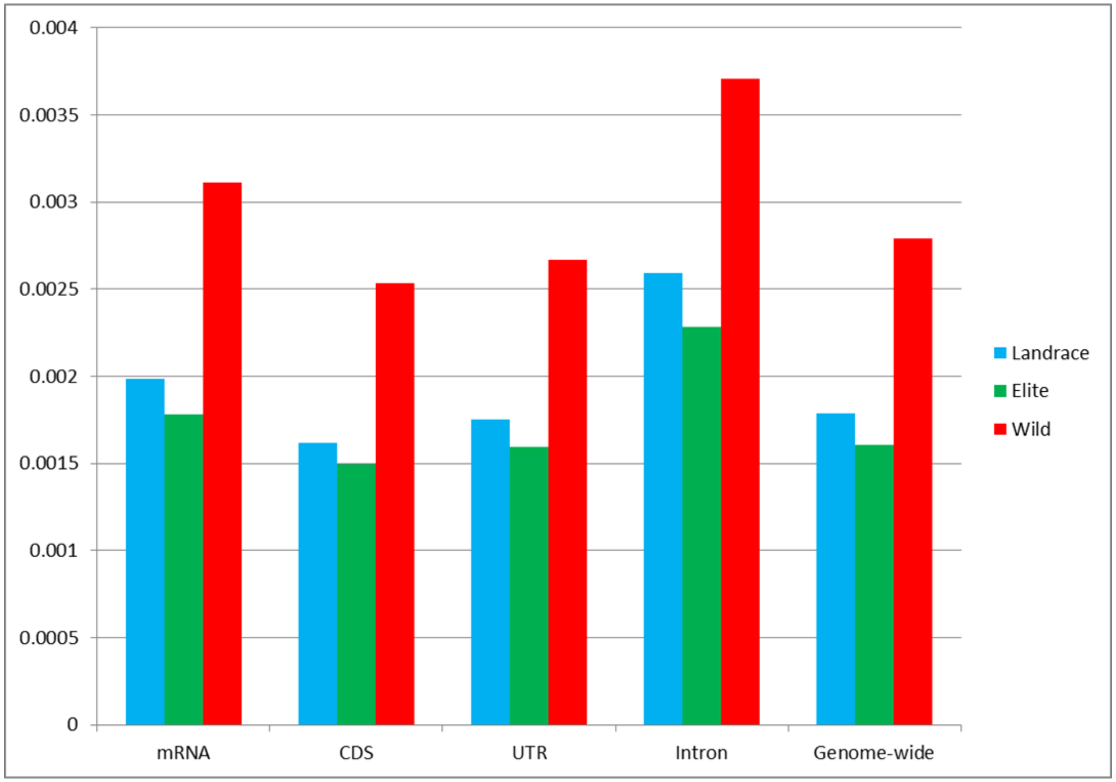

Supplementary Figure 8. Distribution of Genomic diversity (θ_π) for wild (red), landrace (blue), elite soybeans (green) and cultivated (orange) in 20 soybean chromosomes.

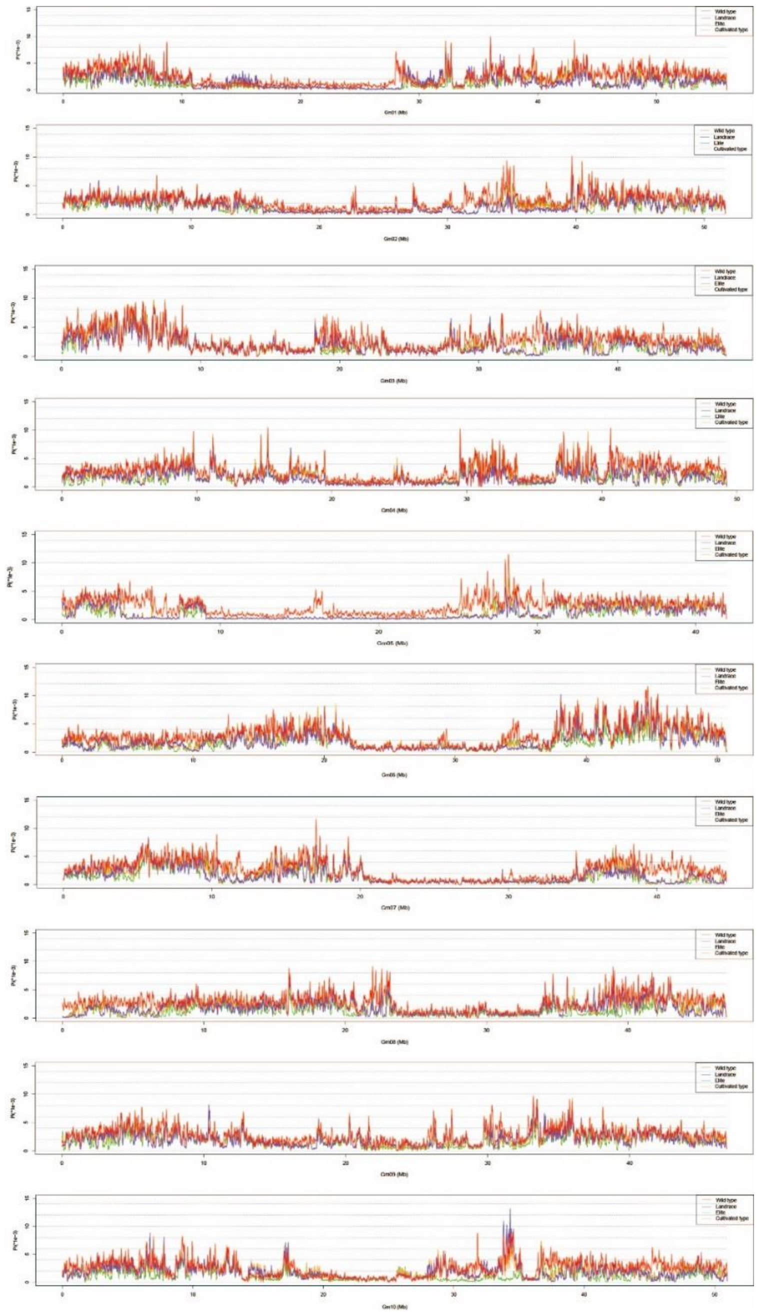

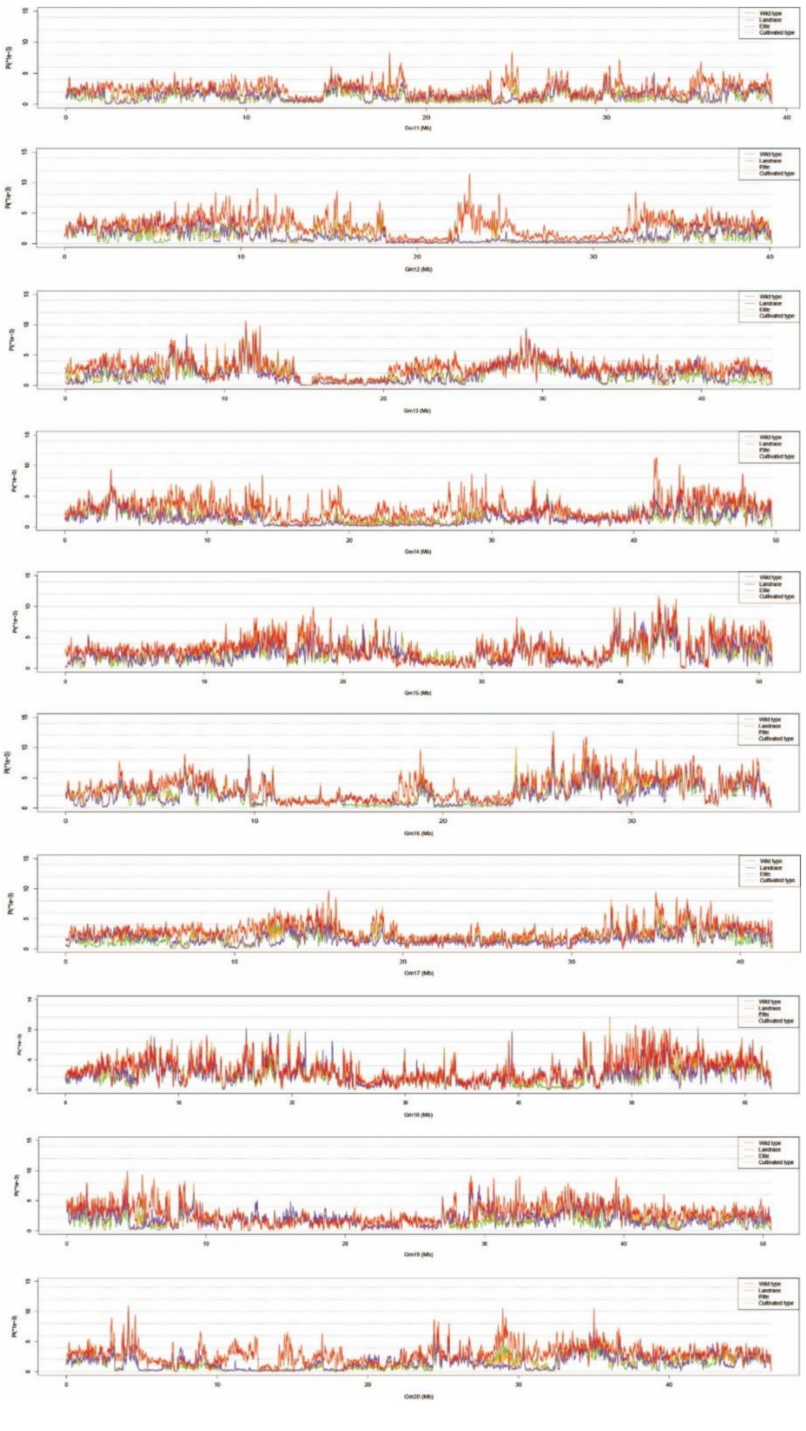
Wild type
Landrace
Elite
Cultivated type
Gm11 (Mb)
Gm12 (Mb)
Gm13 (Mb)
Gm14 (Mb)
Gm15 (Mb)
Gm16 (Mb)
Gm17 (Mb)
Gm18 (Mb)
Gm19 (Mb)
Gm20 (Mb)

Supplementary Figure 9. Frequency distribution of Genomic diversity (θ_π) and Divergence index value (*Fst*). Both *Fst* and θ_π between wild, landrace and elite soybean was calculated.

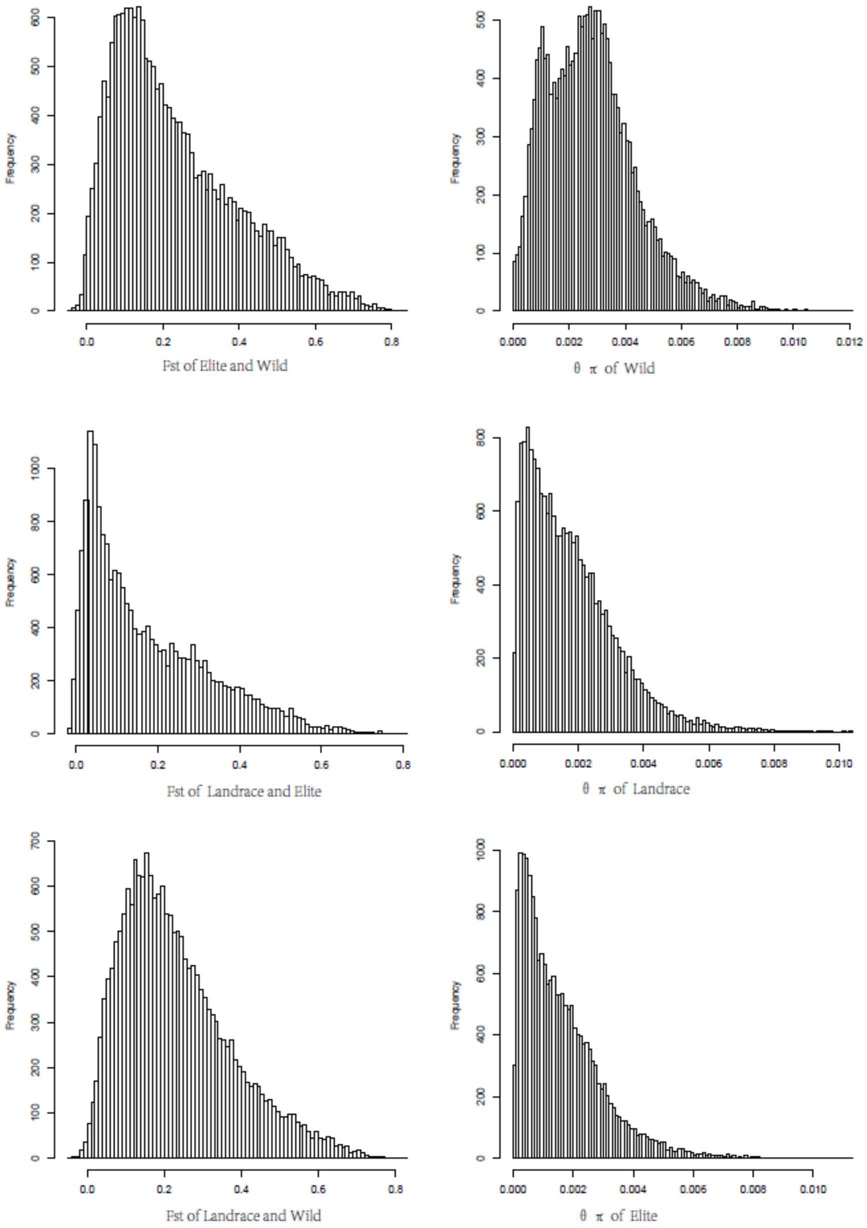

Supplementary Figure 10 a-e. Haplotype analysis of Maturity genes. A- ***E1*****, B-*****E2*****, C-*****E3*****, D-*****E4*****, E-*****Dt1*****.** SNPviz software was used to draw pictorial clusters. Base position identical to reference (W82) are white, black – different, gray-missing data.

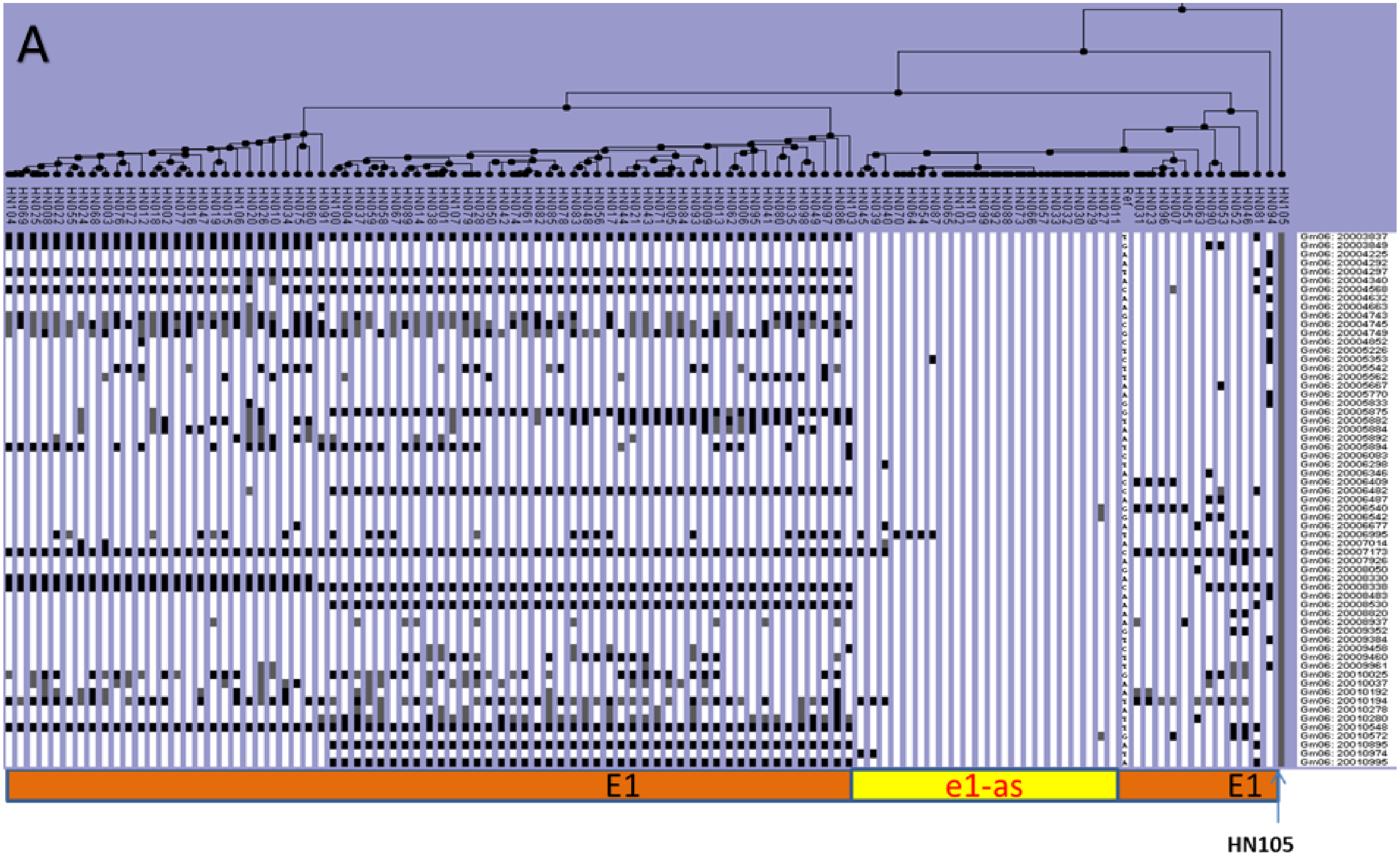

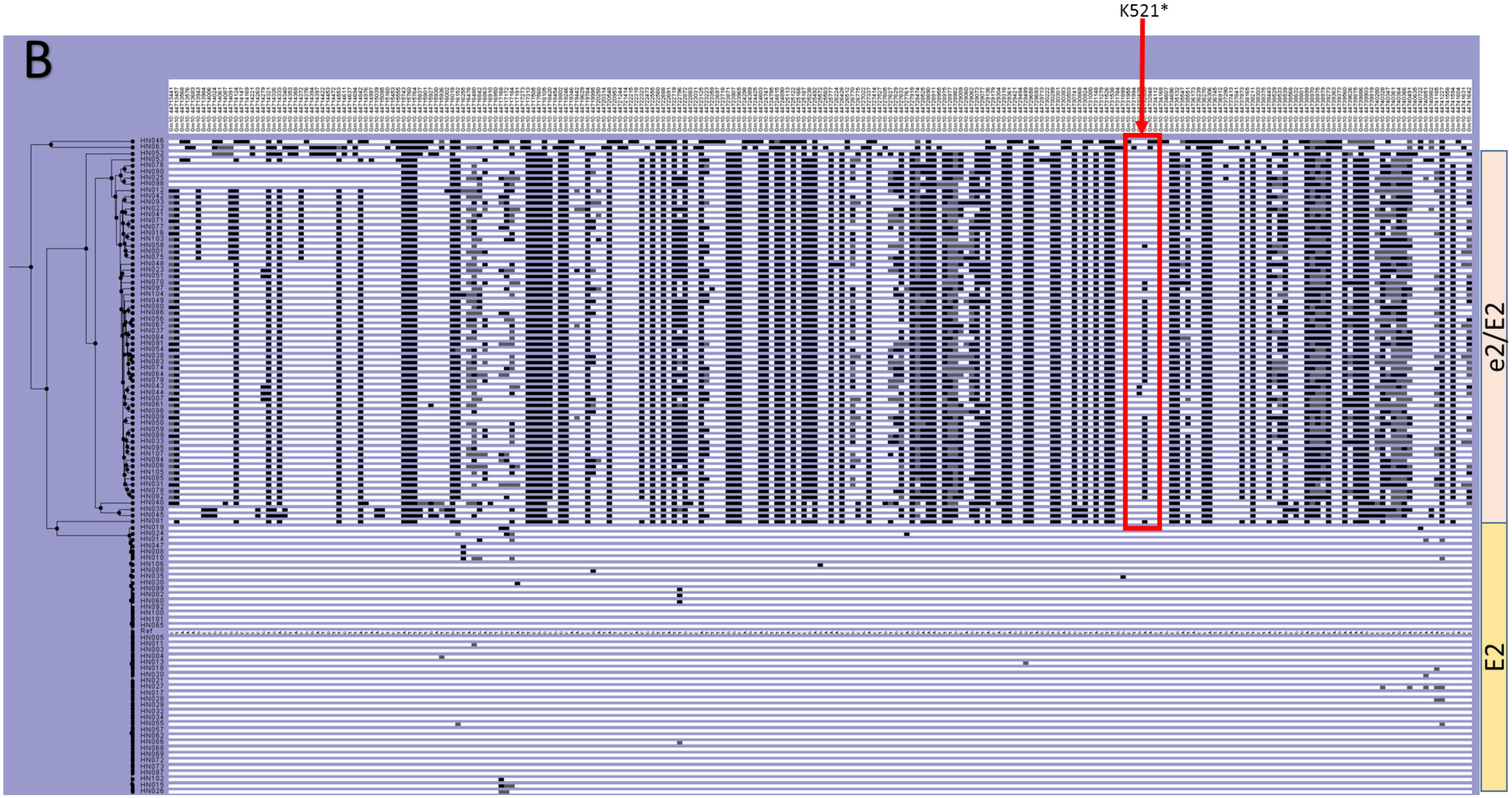

K521*
B
e2/E2
E2

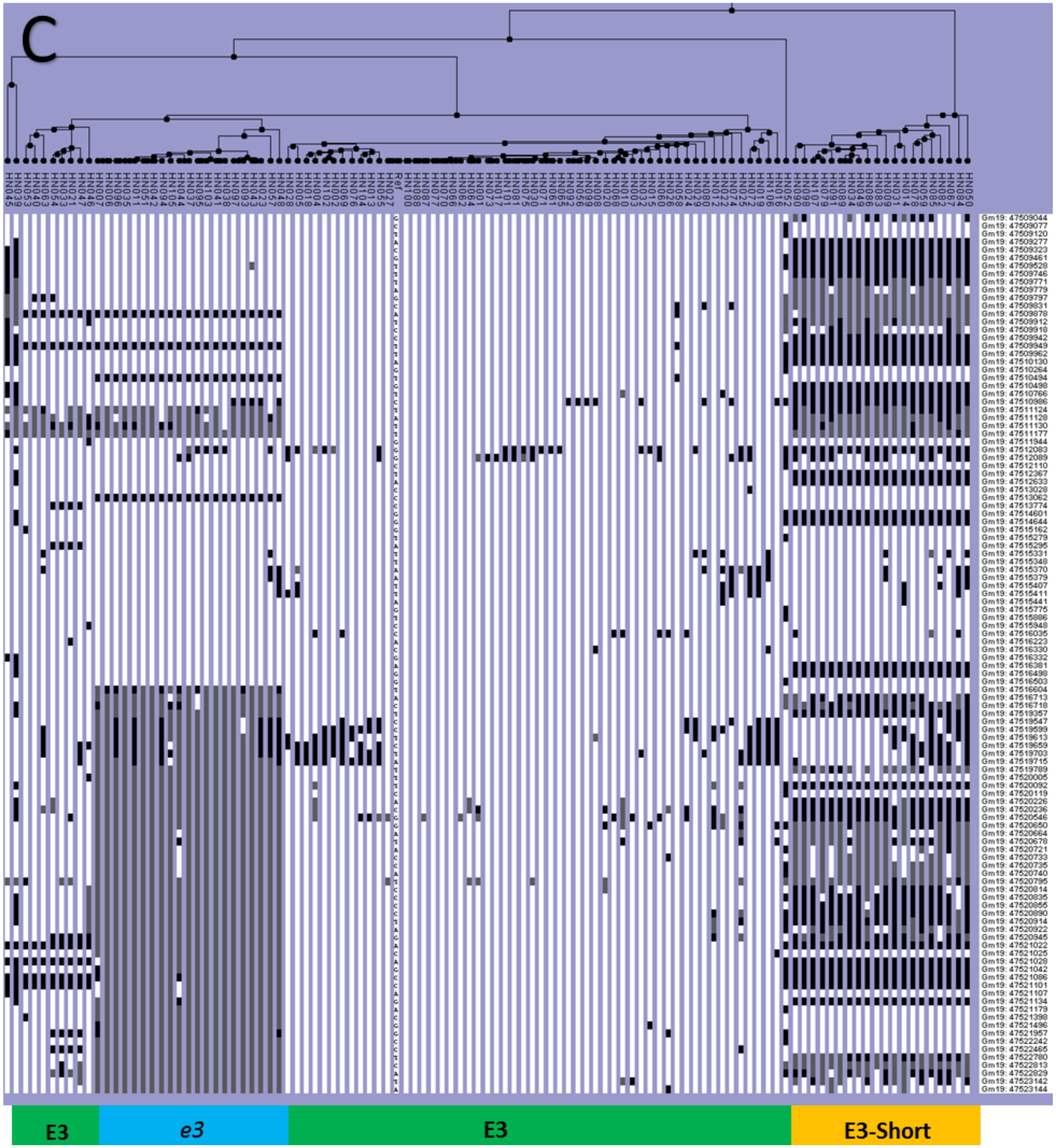
C
E3
e3
E3
E3-Short

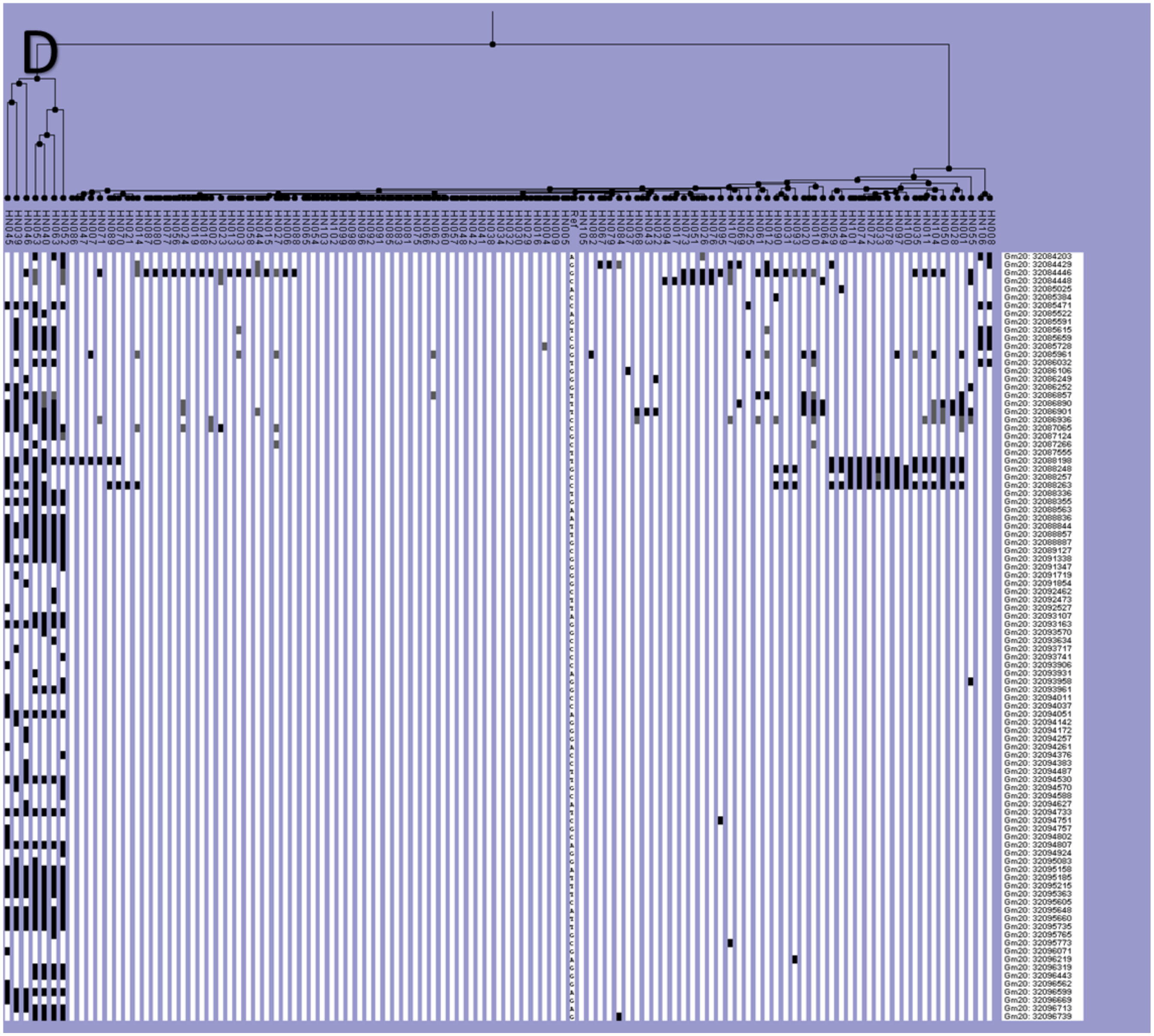
D

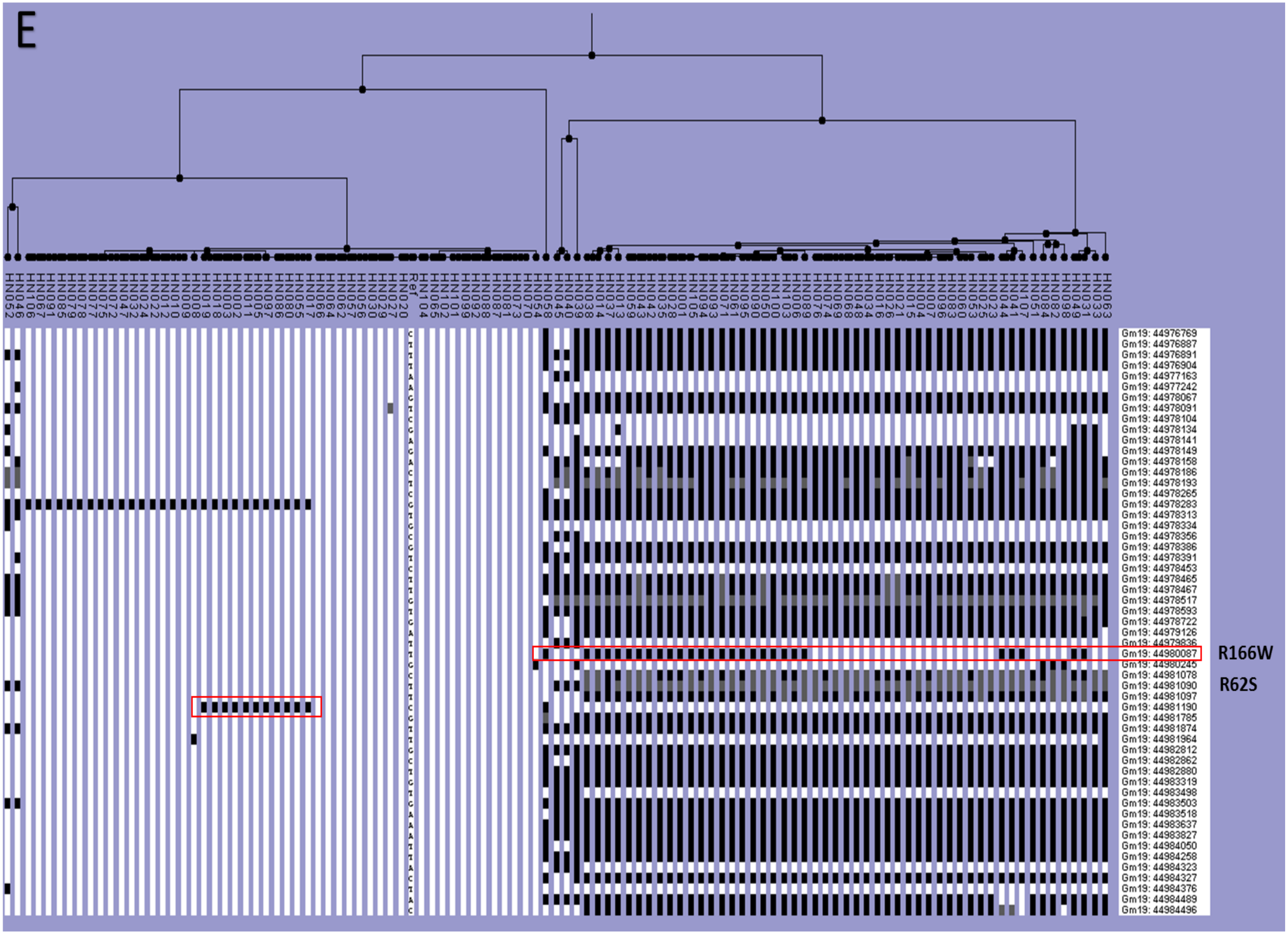
E
Gm19: 44976769
Gm19: 44976887
Gm19: 44976891
Gm19: 44976904
Gm19: 44977163
Gm19: 44977242
Gm19: 44978067
Gm19: 44978091
Gm19: 44978104
Gm19: 44978134
Gm19: 44978141
Gm19: 44978149
Gm19: 44978158
Gm19: 44978186
Gm19: 44978193
Gm19: 44978265
Gm19: 44978283
Gm19: 44978313
Gm19: 44978334
Gm19: 44978356
Gm19: 44978386
Gm19: 44978391
Gm19: 44978453
Gm19: 44978465
Gm19: 44978467
Gm19: 44978517
Gm19: 44978593
Gm19: 44978722
Gm19: 44979126
Gm19: 44979836
Gm19: 44980087
Gm19: 44980245
Gm19: 44981078
Gm19: 44981090
Gm19: 44981097
Gm19: 44981190
Gm19: 44981785
Gm19: 44981874
Gm19: 44981964
Gm19: 44982812
Gm19: 44982862
Gm19: 44982880
Gm19: 44983319
Gm19: 44983498
Gm19: 44983503
Gm19: 44983518
Gm19: 44983637
Gm19: 44983827
Gm19: 44984050
Gm19: 44984258
Gm19: 44984323
Gm19: 44984327
Gm19: 44984376
Gm19: 44984489
Gm19: 44984496
R166W
R62S

Supplementary Figure 11a. **Diversity and allelic variation of lipid biosynthesis genes**. Example showing the allelic variation of lipid biosynthesis related genes associated with a QTL controlling oil content. (a) The *Fst* between wild, landrace and elite soybean; (b) Predicted gene models in the genomic block on Chromosome 13 (4-89-11.49 Mb); (c) Comparative analysis of amino acid polymorphism in the gene *Glyma13g04571.1*, a negative regulator of lipid biosynthesis.

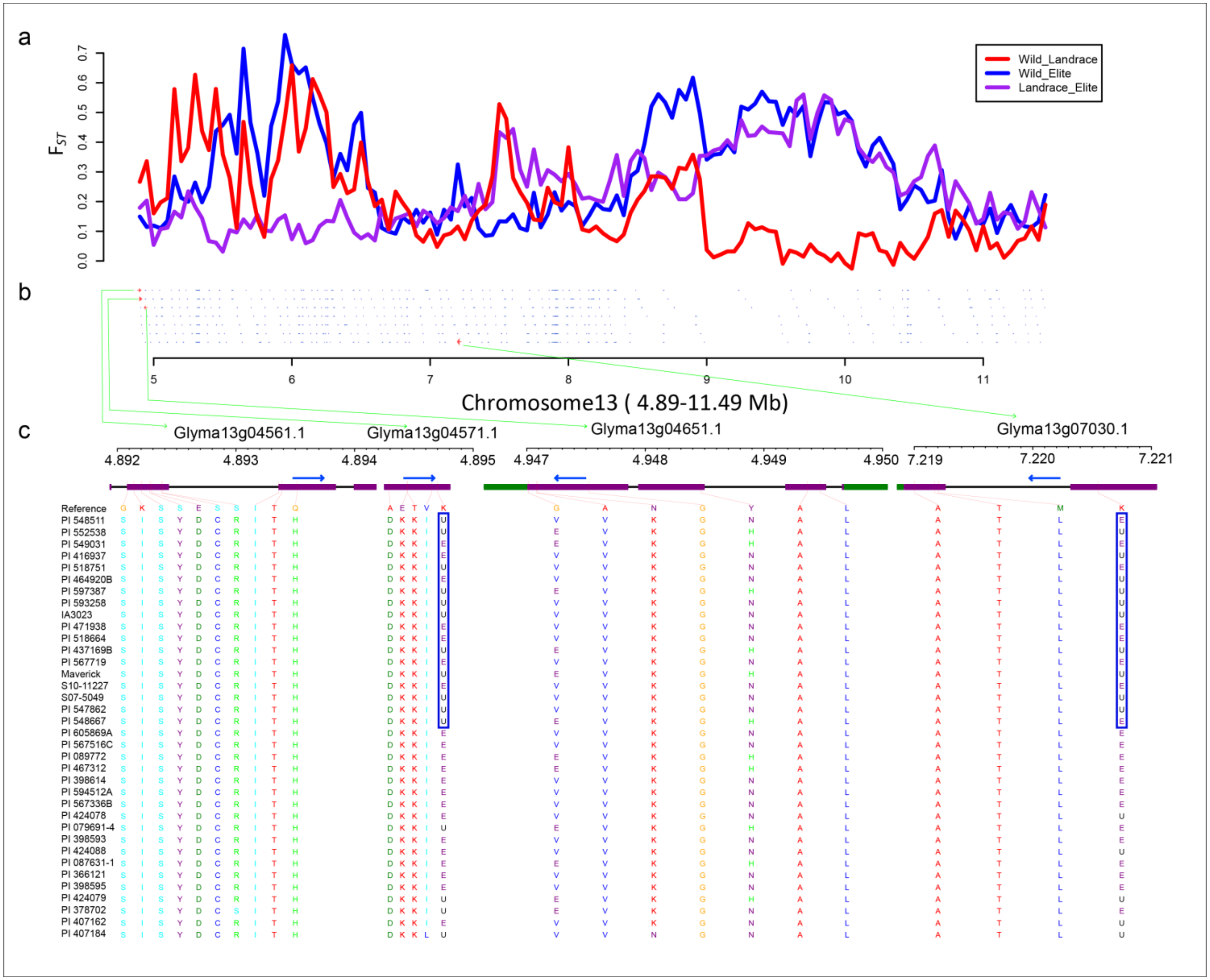

Supplementary Figure 11b. **Diversity and allelic variation of genes associated with protein content.** Example showing the allelic variation of seed protein related genes associated with a QTL controlling protein content. (a) The *Fst* between wild, landrace and elite soybean; (b) The genomic block on chromosome 20 (24.5 to 32.9 Mbp, 8.1 Mbp) is defined as major protein QTL for protein content. Predicted gene models in this genomic region; (c) Comparative analysis of amino acid polymorphism in the gene HSP, *Glyma20g19680*; Ammonium transporter, *Glyma20g21030*; ethylene receptor, *Glyma20g21780* associated with protein content.

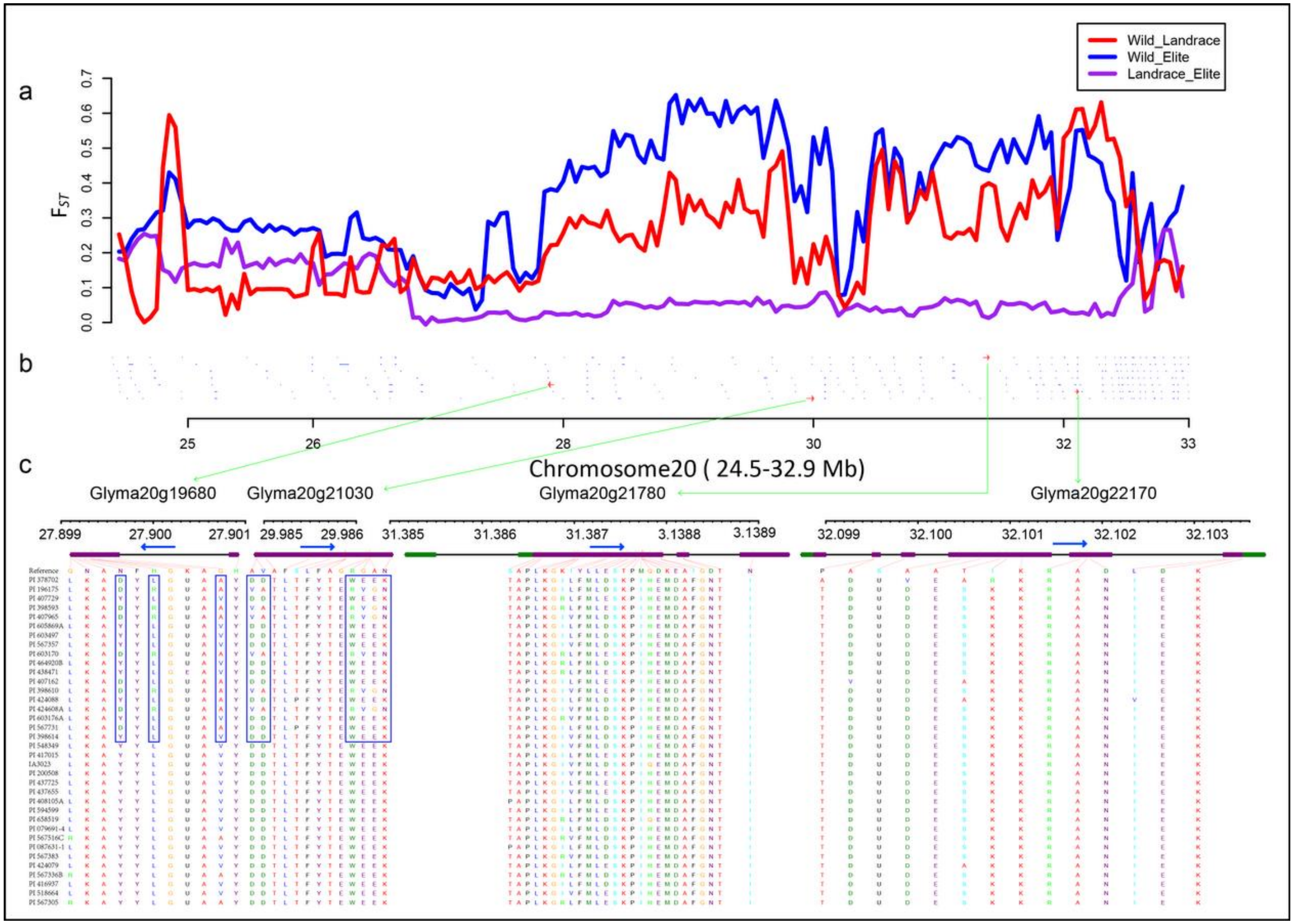

Supplementary Figure 12a-b. Gene Structure variation in 106 lines. a. Phylogenetic clustering of *CHX1* locus (gene + 2 kb promoter region) preceded three major clades. Samples were grouped by their sequence identity to the reference (W 82). W05 and C08 were used for comparing salt-tolerant and salt sensitive genotypes, respectively[23]. Known indicator lines are marked with arrows. **b.** *CHX1* gene architecture in 106 lines. Dotted lines indicating the exon position. Red lines indicating that this region is deleted in group 1 (G1). G2 and G1 group indicates the sensitive while G3 indicates salt tolerant group. However, no deletion was tested in the exon at the G1 group.

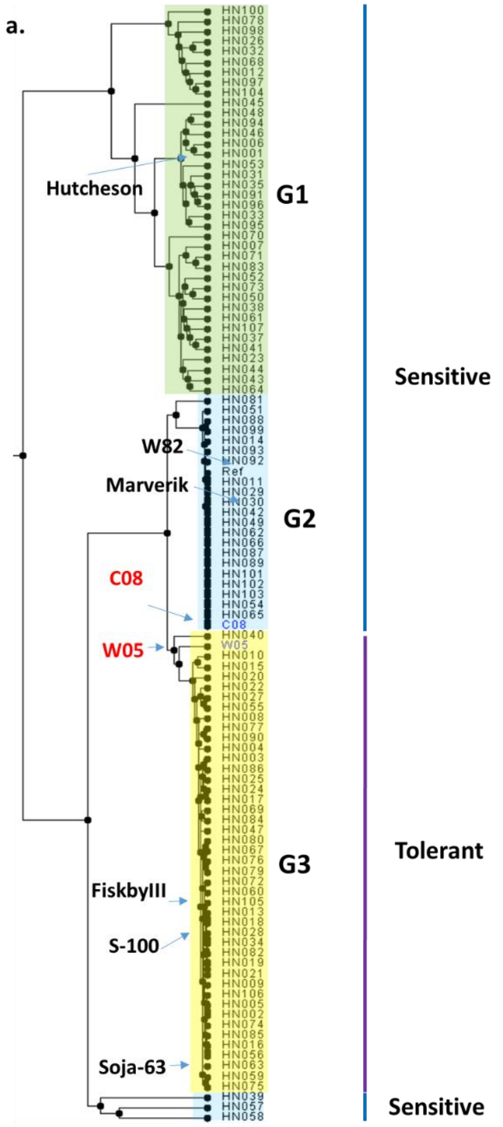

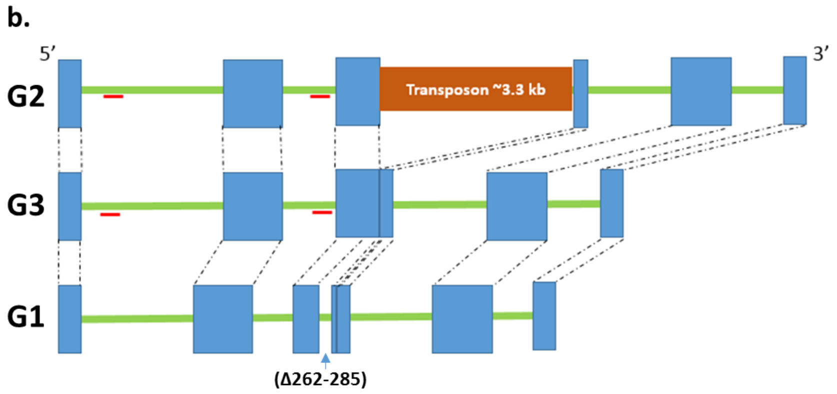

Supplement: Supplementary Information [file srep23598-s1.pdf]
